# Disordered gut microbiota and alterations in metabolic patterns are associated with atrial fibrillation

**DOI:** 10.1093/gigascience/giz058

**Published:** 2019-05-30

**Authors:** Kun Zuo, Jing Li, Kuibao Li, Chaowei Hu, Yuanfeng Gao, Mulei Chen, Roumu Hu, Ye Liu, Hongjie Chi, Hongjiang Wang, Yanwen Qin, Xiaoyan Liu, Shichao Li, Jun Cai, Jiuchang Zhong, Xinchun Yang

**Affiliations:** 1 Heart Center & Beijing Key Laboratory of Hypertension, Beijing Chaoyang Hospital, Capital Medical University, Beijing 100020, China; 2 The Key Laboratory of Upper Airway Dysfunction-related Cardiovascular Diseases, Beijing An Zhen Hospital, Capital Medical University, Beijing Institute of Heart, Lung and Blood Vessel Diseases, Beijing 100029, China; 3 Medical Research Center, Beijing Chaoyang Hospital, Capital Medical University, Beijing 100020, China; 4 Hypertension Center, Fuwai Hospital, State Key Laboratory of Cardiovascular Disease of China, National Center for Cardiovascular Diseases of China, Chinese Academy of Medical Sciences and Peking Union Medical College, Beijing 100037, China

**Keywords:** atrial fibrillation, gut microbiota, metagenome, metabolism

## Abstract

**Background:**

With the establishment of the heart-gut axis concept, accumulating studies suggest that the gut microbiome plays an important role in the pathogenesis of cardiovascular diseases. Yet, little evidence has been reported in characterizing the gut microbiota shift in atrial fibrillation.

**Methods:**

We include the result of the global alterations that occur in the intestinal microbiota in a cohort of 50 patients with atrial fibrillation and 50 matched controls based on a strategy of metagenomic and metabolomic analyses.

**Results:**

The alterations include a dramatic elevation in microbial diversity and a specific perturbation of gut microbiota composition. Overgrowth of *Ruminococcus*, *Streptococcus*, and *Enterococcus*, as well as reduction of *Faecalibacterium*, *Alistipes*, *Oscillibacter*, and *Bilophila* were detected in patients with atrial fibrillation. A gut microbial function imbalance and correlated metabolic pattern changes were observed with atrial fibrillation in both fecal and serum samples. The differential gut microbiome signatures could be used to identify patients with atrial fibrillation.

**Conclusions:**

Our findings characterize the disordered gut microbiota and microbial metabolite profiles in atrial fibrillation. Further research could determine whether intervention strategies targeting intestinal microbiome composition might be useful to counteract the progression of atrial fibrillation.

## Background

Atrial fibrillation (AF), an abnormal heart rhythm characterized by rapid and irregular beating of the atria, is the most common arrhythmia, with heavy global burdens, intensifying disability, and morbidity. In Europe and the USA, 1 in 4 middle-aged adults will experience AF [1, 2]. AF is prevalent in ∼3% of adults at the age of 20 years or older [3], with greater prevalence in older persons and in patients with conditions such as hypertension (HTN), heart failure, obesity, or type 2 diabetes mellitus (T2DM) [4]. AF is independently associated with a 2-fold increased risk of all-cause mortality in women and a 1.5-fold increase in men [5] and has become a significant contributor to cardiovascular events leading to cardiac death worldwide. Currently, ideal preventive and therapeutic strategies to counteract the progression of AF remain sparse. The heterogeneity of the underlying atrial substrate, extent of atrial fibrosis, and the discrepancies among inter-individual electrophysiological characteristics contribute to unpredictable responses to drug or ablation therapy [6]. It is essential to embrace AF prevention as a priority, focusing not only on rate, rhythm control, or stroke prevention but also considering AF as a concomitant factor of adverse atrial remodeling rather than a solitary disease. Therefore, efforts to identify the pathological mechanisms of AF are warranted. Various genetic mutations have been identified to be associated with AF [7], and environmental or unhealthy lifestyle factors are also believed to contribute to the development of AF [8]. It is worth noting that AF risk factors or contributors, such as HTN, T2DM, and obesity, have been linked to dietary intake that possibly contributes to alterations in the composition of the gut microbiota [8–11].

Recently, more investigators have focused on the role of the gut microbiome (GM), which has been identified as an essential factor affecting human health [9–14]. Dysbiotic GM has been reported in multiple diseases, such as T2DM [10], obesity [11], HTN [9], atherosclerotic cardiovascular disease (ACVD) [15], liver cirrhosis [12], colorectal adenoma-carcinoma [13], rheumatoid arthritis [14], irritable bowel syndrome [16], and anxiety and depression [17], and shown to activate the immune system [18], eliciting chronic diseases. As the understanding of the relationship between the intestinal microbiome and diseases has deepened, possible underlying mechanisms have been proposed. For example, emerging evidence suggests that through immune system and metabolic alterations, gut microbiota disequilibrium could induce obesity, HTN, and T2DM, traditional cardiac risk factors that play an essential role during atrial remodeling in the development of AF [8, 19]. However, data demonstrating a correlation between AF and the intestinal microbiome are still lacking. To our knowledge, studies of gut microbiota and AF have been few. Information regarding the impact of microbial metabolites is also incomplete. A gut microbial−dependent metabolite, trimethylamine *N*-oxide (TMAO), which is positively correlated with cardiovascular disease (CVD) in humans, is proatherogenic and could increase the instability of atrial electrophysiology [20]. However, it remains unclear whether circulating TMAO levels derived from the intrinsic microbiome can reach the ganglionated plexi and create local concentrations sufficient to result in comparable arrhythmogenic effects. In addition, recent studies have shown that gut-derived lipopolysaccharide is predictive for major adverse cardiovascular events in patients with AF [21]. Furthermore, microbiome-derived free fatty acids, such as palmitic and adrenic acid, might have potential influences on arrhythmogenesis [22, 23].

These seminal studies provided the first clues indicating a possible interaction between gut microbiota and AF. They encouraged us to identify direct evidence of gut bacteria alterations in patients with AF and evaluate the possible contribution of gut dysbiosis to aberrant metabolic patterns that accelerate the progression of AF. We performed metagenomic sequencing analyses of stool samples from patients with AF to outline the potential compositional and functional alterations of the GM. In addition, to expose the relationship between disordered GM and altered metabolomic profiles in AF, we aimed to construct a microbiota-dependent discrimination index for distinguishing AF, thus providing a comprehensive understanding of gut microbiota dysbiosis in the progression of AF. This work is fundamental for further studies to reveal the causal relationship and explore preventative measures for postponing AF progression.

## Results

### Baseline characteristics of the study cohort

We enrolled 100 Chinese participants comprising 50 patients with nonvalvular AF and 50 individuals as matched controls. AF was diagnosed using an electrocardiogram and defined as the absence of P waves, replaced by disorganized electrical activity and irregular R–R intervals due to irregular conduction of impulses to the ventricles [24]. To adjust for the effect of HTN on gut microbiota composition, we selected 50 samples from our previous gut microbiota work matched for a history of HTN [9]. None of the participants had heart failure, coronary heart disease, structural heart disease, inflammatory bowel diseases, irritable bowel syndrome, autoimmune diseases, liver diseases, renal diseases, or cancer. Patients who had used antibiotics or probiotics in the past month were excluded. The clinical characteristics of all participants are presented in Table 1. There was no significant difference between patients with AF and controls in terms of body mass index, creatinine, total bilirubin, or glutamic-pyruvic transaminase level. Most of the patients were elderly, with 70% >60 years old. For the control group, there were more males than females, with males accounting for 82%. Although the total cholesterol serum levels were much lower in patients with AF, these clinical indices were all within the normal range.

**Table 1:** Baseline clinical characteristics of the study cohort

Characteristic	AF	Control	*P*-value
Number	50	50	
Age, years	66 (57.00, 71.25)	55 (50.50, 57.50)	<0.001
Male/female sex	32/18	41/9	0.043
Body mass index	26.46 (23.79, 28.64)	24.77 (22.79, 27.62)	0.112
HTN	27	27	
T2DM	12	0	
**Total cholesterol**	4.13 ± 1.05	4.82 ± 0.96	0.001
Triglyceride	1.29 (1.02, 1.88)	1.06 (0.77, 1.80)	0.084
LDL cholesterol	2.45 (1.58, 2.93)	2.3 (1.96, 2.86)	0.872
Fasting blood glucose	4.95 (4.50, 5.83)	5.12 (4.56, 5.55)	0.883
Creatinine	68.5 (60.48, 79.35)	70 (60, 89.5)	0.533
Uric acid	321.5 (278, 389.75)	333 (264.5, 384)	0.927
Total bilirubin	14 (10.08, 19.5)	14.7 (11.59, 19.75)	0.431
Glutamic-pyruvic transaminase	19 (13.75, 28.5)	19 (12, 25)	0.185
Drug use			
Angiotensin-converting enzyme inhibitors	7	0	
Angiotensin receptor blockers	4	0	
β receptor blockers	8	0	
Statins	4	0	
Aspirin	2	0	
Amiodarone	10	0	
Dimethyl biguanide	6	0	
Oral anticoagulation therapy	13	0	0

Data are presented as mean ± standard deviation or median (interquartile range), as appropriate.

### Elevated microbiota richness and altered community types in the gut of patients with AF

**Figure 1:**
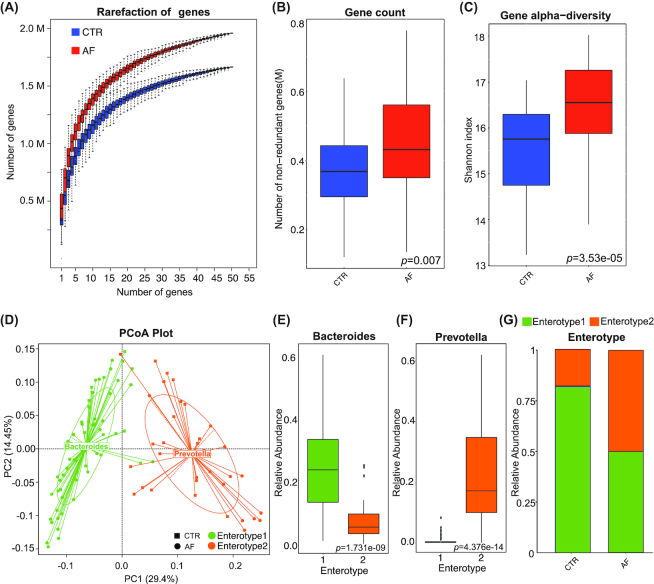
Elevated microbiota richness and altered community types in patients with AF. A, Rarefaction curves for gene number, which were calculated after 50 iterations of random sampling with replacement in control (CTR; n=50) and AF (n=50). X-axis is the number of samples and Y-axis means number of genes. The rarefaction curve is near smooth when the sequencing data are great enough with few new genes undetected and the present sample size has met the need of this study. B and C, Gene count (B) and α-diversity (Shannon index) (C) based on the genera profile in the AF and control cohorts. Boxes represent the interquartile ranges, and lines inside the boxes denote medians. Gut microbial richness (gene count) and diversity in the AF group were much higher (*P*=0.007, control vs AF, for gene count; *P*=3.53e−05, control vs AF, for α-diversity; Kruskal-Wallis test). D, 100 samples are clustered into enterotype 1 (green) and enterotype 2 (orange) by principal component analysis (PCA) of Jensen-Shannon divergence values at the genus level. The major contributor in the 2 enterotypes is *Bacteroides* and *Prevotella*, respectively. E and F, Relative abundances of the top genera in each enterotype, *Bacteroides* in enterotype 1 (E), *Prevotella* in enterotype 2 (F). Boxes represent the interquartile ranges, lines inside the boxes denote medians, and circles are outliers. *P*=1.731e−09 and *P*=4.376e−14, respectively; Wilcoxon rank sum test. G, The percentage of control and AF samples distributed in enterotype 1 and enterotype 2. A dysbiosis of enterotype distribution by AF conditions was revealed. There were 50% controls in enterotype 1, 50% controls in enterotype 2; 82% AF participants in enterotype 1, 18% AF participants in enterotype 2. *P*=0.001, control vs AF; Fisher's exact test, PCoA: principal coordinate analysis.

Whole-metagenome shotgun sequencing of the 100 stool samples from our study cohort was performed. A total of 612.84 Gb high-quality sequencing reads were generated (6.13 ± 0.96 million reads per sample on average) (Additional files 1: Table S1). Rarefaction analyses, performed as we previously described [9], showed that the curves approached saturation in each group and with a significantly increased gene number in the microbiomes of patients with AF (Fig. 1A). We also compared the gene count, within-sample diversity (Shannon index), and 3 other ecological parameters, including Chao richness, Pielou evenness, and Firmicutes/Bacteroidetes ratio between controls and patients with AF. Consistently, gut microbial richness (gene count) and diversity in the AF group were much higher (*P* = 0.007 for gene count, Fig. 1B; *P* = 3.53e−05 for Shannon index, Fig. 1C; *P* = 7.162e−05 for Firmicutes/Bacteroidetes ratio, Additional files 2: Fig. S1a; *P* = 0.007633 for Chao richness, Fig. S1b; *P* = 4.262e−06 for Pielou evenness, Fig. S1c). The elevated richness of genes or genera observed in our cohort may suggest the overgrowth of a variety of harmful bacteria in patients with AF.

To investigate the shift of gut microbiota community structure during the AF state, microbial enterotype features were examined using the partitioning around medoids clustering method. The 100 samples were divided into 2 clusters by principal coordinate analysis based on the Jensen-Shannon divergence (Fig. 1D). Enterotype 1 was dominated by *Bacteroides* as the most enriched genus, and *Prevotella* was the core in enterotype 2 (*P* = 1.731 e−09 and *P* = 4.376 e−14, respectively; Wilcoxon rank sum test, Fig. 1E and F). Both enterotypes have been previously reported in HTN, T2DM, colorectal cancer, and irritable bowel syndrome [9, 10, 13, 16]. There were 12 other significantly increased genera in enterotype 1, including *Blautia*, *Coprobacillus*, *Dorea*, *Enterococcus*, *Streptococcus*, and *Veillonella* (Additional files 3: Fig. S2). Interestingly, there was a dysbiosis of enterotype distribution by AF conditions. For the control group, the percentage of samples in both enterotypes was the same (50% in enterotype 1, 50% in enterotype 2), whereas a higher percentage of patients with AF were found to be distributed in enterotype 1 (82%), and less in enterotype 2 (*P* = 0.001, AF vs control; Fisher's exact test; Fig. 1G). Furthermore, a similar difference in enterotype distribution at the species level was also found, although no significantly different species were found between enterotypes (Additional files 4: Fig. S3). Therefore, a morbid state of AF is associated with imbalanced gut microbial communities, with a tendency towards the enterotype dominated by *Bacteroides* and away from the *Prevotella*-prominent enterotype.

### Taxonomic profile of AF-associated gut microbiota

To compare the taxonomic profile of gut microbiota in patients with AF with those in healthy individuals, we accessed the GM abundances and phylogenetic profiles at the genus level. Genes were aligned to the nonredundant (nr) database using DIAMOND61 (Version 0.7.9.58) and annotated to taxonomic groups (Additional files 5: Fig. S4). The relative abundance of gut microbes was calculated by summing the abundance of genes as listed in Additional files 6 and 7: Tables S2 and S3. The state of disease significantly separated the participants with AF from those without AF in principal component analysis (PCA) or in non-metric dimensional scaling (NMDS) analysis at the genus level (Additional files 5: Fig. S4a and b). The 35 most abundant genera in patients with AF and healthy controls are shown in Additional files 5: Fig. S4c.

**Figure 2:**
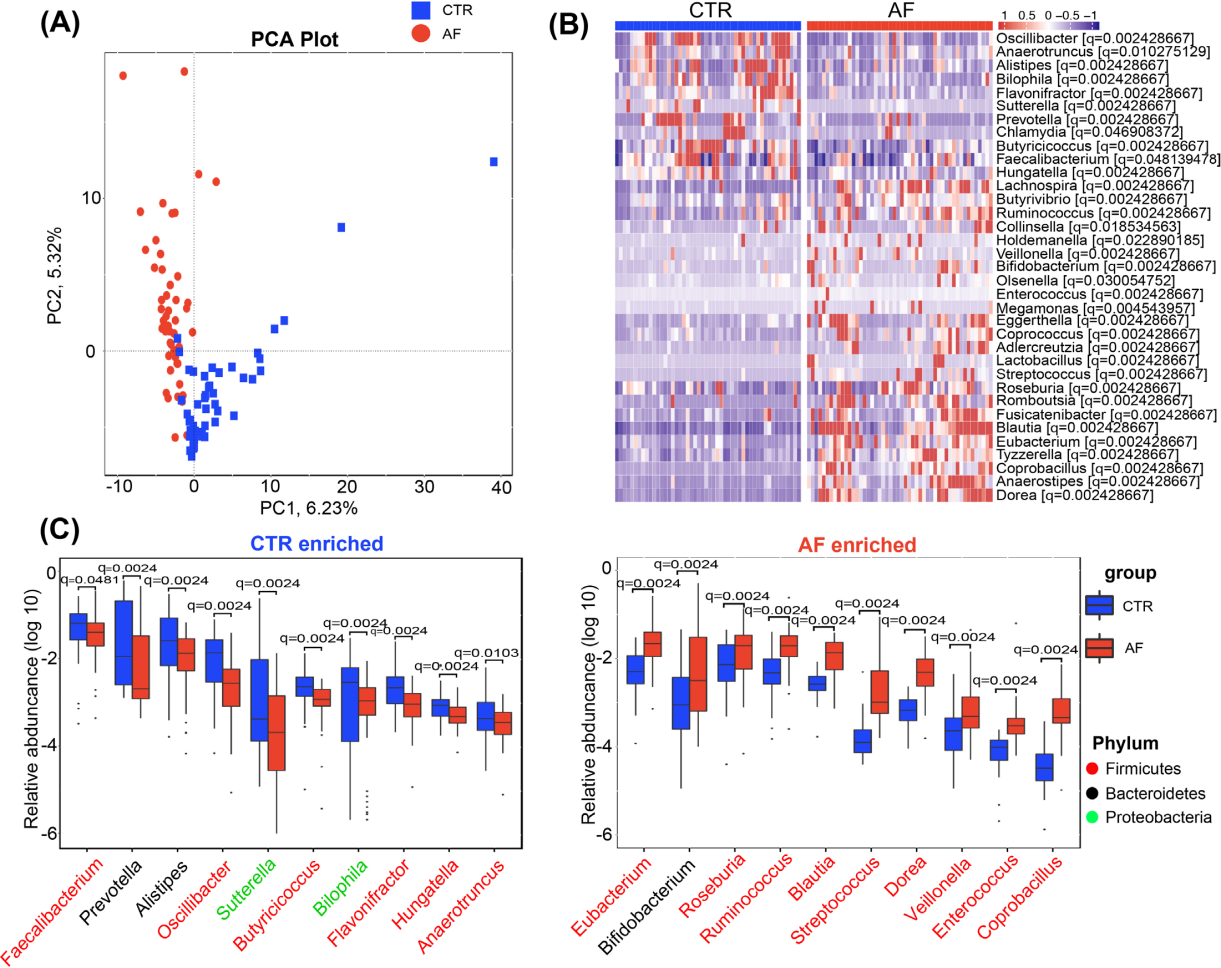
Genera strikingly different across groups. A, PCA based on abundances of the microbes showed that the structures of gut microbiota in AF were significantly different from controls (CTR) at the genus level. B, Relative abundance of the top 35 most different genera across groups at the criteria of *q* value <0.05 (comparisons presented in the square brackets); Wilcoxon rank sum test. The abundance profiles are transformed into *z* scores by subtracting the average abundance and dividing the standard deviation of all samples. *z* score is negative (shown in blue) when the row abundance is lower than the mean and red when the row abundance is higher than the mean. C, The box plot shows the relative abundance of the top 10 genera enriched in controls and AF participants. Boxes represent the interquartile ranges, lines inside the boxes denote medians, circles are outliers, and *q* (adjusted *P*) value is shown at the top of the box; Wilcoxon rank sum test.

Overall, 574 genera were dramatically different in control and AF participants (*P* < 0.05, *P* values were tested using the Wilcoxon rank sum test and corrected for multiple testing with the Benjamini and Hochberg method [12]; Additional files 8: Table S4). Consistent results were also obtained when the PCA analysis was performed on the basis of the genera or species differentially enriched across groups (*P* < 0.05, analysis of similarities, genus: Fig. 2A, species: Additional files 9: Fig. S5a). The top 10 different gut bacteria that dominated in AF participants or controls at the genus level are shown in Fig. 2C and D. In patients with AF, the proportions of *Streptococcus*, *Enterococcus, Blautia*, *Dorea*, *Veillonella*, and *Coprobacillus* were much higher than in controls (Fig. 2C), in agreement with our previous observations that they were more abundant in the AF-correlated enterotype (enterotype 1). In addition to *Eubacterium, Bifidobacterium*,and*Roseburia*, *Ruminococcus* were also overexpressed in individuals with AF (Fig. 2C). *Ruminococcus* is known to possess a pro-inflammatory property, which has been implicated in the development of inflammatory bowel disease [25–27]. Transplantation of *Ruminococcus* into germ-free mice has been reported to enhance the levels of interferon-γ, interleukin 17, and interleukin 22 [26]. *Streptococcus*, recognized as a morbific oral bacterial genus, has also been demonstrated to be elevated in HTN [9], congestive heart failure (CHF) [28], and ACVD [15, 29]. Furthermore, *Veillonella*, a gram-negative anaerobic coccus, was suggested to be inversely correlated with cardiovascular protective metabolites such as niacin, cinnamic acid, and orotic acid [30]. In addition, *Enterococcus* is known to produce cytolysin, a toxin that causes rupture of a variety of target membranes, including bacterial cells, erythrocytes, and other mammalian cells [31].

Of the top 10 different species in the AF group shown in Fig. S5c, *Escherichia coli*, a potentially pathogenic bacterial species, was the most abundant and may be correlated with the progression of AF. *Eubacterium rectale* is a main representative of Firmicutes and a kind of conditioned pathogen, which can ferment the metabolic products of glucose (such as formic acid, acetic acid, and butyric acid) as well as proteins, thereby inhibiting the proliferation of other beneficial bacteria in the intestines and decreasing the production of catabolic enzymes of glycan [32]. Furthermore, species enriched in the AF group, including *Bifidobacterium longum*and*Collinsella aerofaciens*, were more abundant in patients with metastatic melanoma [33]. Meanwhile, *Faecalibacterium prausnitzii* [34], the butyrate-producing bacterial species, was found decreased in the AF group. These results showed the imbalanced structure of the intestinal flora, reduced probiotic species, and increased quantity of harmful bacteria in patients with AF. It is speculated that these clusters of conditioned pathogens accumulated in the gut might influence AF susceptibility.

Moreover, *Faecalibacterium*, *Prevotella*, *Alistipes*, *Oscillibacter* (genus level), and *Sutterella* were dramatically decreased in the patients with AF compared with controls, and a similar shift was found for *Butyricicoccus, Flavonifractor*, and *Bilophila* (Fig. 2C). In addition, we also identified a dramatic decline of species such as *F. prausnitzii*, *Oscillibacter sp*.(species level), and also *Firmicutes bacterium* (species level) in the patients with AF (Fig. S5c). *F. prausnitzii* is a butyrate-producing commensal bacterium with anti-inflammatory properties, and its deficiency may aggravate chronic inflammation, leading to ulcerative colitis, Crohn's disease, obesity, asthma, and major depressive disorder [35–38]. *Alistipes* is a common member of the human intestinal microbiota, capable of producing short-chain fatty acids from amino acids, such as succinic and acetic acids [39]. The enrichment of *Oscillibacter sp*. and *Alistipes* was previously reported to be essential for maintaining balanced gut microbes to protect against HTN [9], CHF [28], and ACVD [15]. In addition, *Bilophila* is found in normal flora in human feces [40] and *Flavonifractor* was enriched in the feces of non-obese participants [41].

Considering the difference of baseline characteristics, including sex, age, T2DM diagnosis, and total cholesterol levels between the 2 groups, we questioned whether the alterations of GM observed in patients with AF were mediated by these clinical factors [10, 42, 43]. A PCA plot was performed to assess the contribution of these factors, and the results showed that it failed to distinguish patients with AF into a separate group based on these factors, indicating the negligible impact of sex, age, T2DM, or total cholesterol on our results (*P* > 0.05, analysis of similarities, Additional files 10: Fig. S6).

Additionally, medication is a key factor that can alter the GM as shown in previous studies [43, 44]. Therefore, the effects of statin and dimethyl biguanide use were further analyzed by PCA plots to assess the possible influence of drug consumption on GM in patients with AF. As indicated above, there were 4 patients with AF taking statins and 6 taking dimethyl biguanide. The PCA at the genus level failed to separate the patients with AF into different clusters based on the use of statins or dimethyl biguanide (*P* > 0.05, analysis of similarities; Additional files 10: Fig. S6e). These findings based on the taxonomic profile of gut microbiota supported our hypothesis that there is serious dysbiosis of the gut bacteria under the AF state, which may play a crucial role in the pathology of atrial remodeling and the formation of an arrhythmogenic substrate.

### AF state is identifiable by the gut co-abundance group

At the gene level, there were 121,145 genes differentially enriched in patients with AF versus the controls (Additional files 11: Table S5). These genes were further clustered into co-abundance gene groups (CAGs) as we described previously [9], which generated 15,289 distinct CAGs (Additional files 12–15: Tables S6–S9). The confidence of taxonomic annotation, confidence of individual CAG assignment, and distribution of CAG size (number of genes) is shown in Fig. S7 (Additional files 16). A total of 477 CAGs were assigned to known bacterial genera based on the tracer genes, with ≥80% of the genes mapped to the reference genome at an identity >85%. The CAGs were then compared with those of the controls, yielding 240 CAGs specifically enriched in AF (Additional files 13: Table S7). A cluster of CAGs containing *Prevotella*, along with anti-inflammatory CAGs such as *Faecalibacterium*, were more abundant in the healthy controls (Additional files 17: Fig. S8). In contrast, the AF-enriched CAGs formed a cluster originating from proinflammatory *Ruminococcus*, *Dorea*, *Eubacterium*, and *Bacteroides*, some microbes enriched in CVD [9, 15, 28].

**Figure 3:**
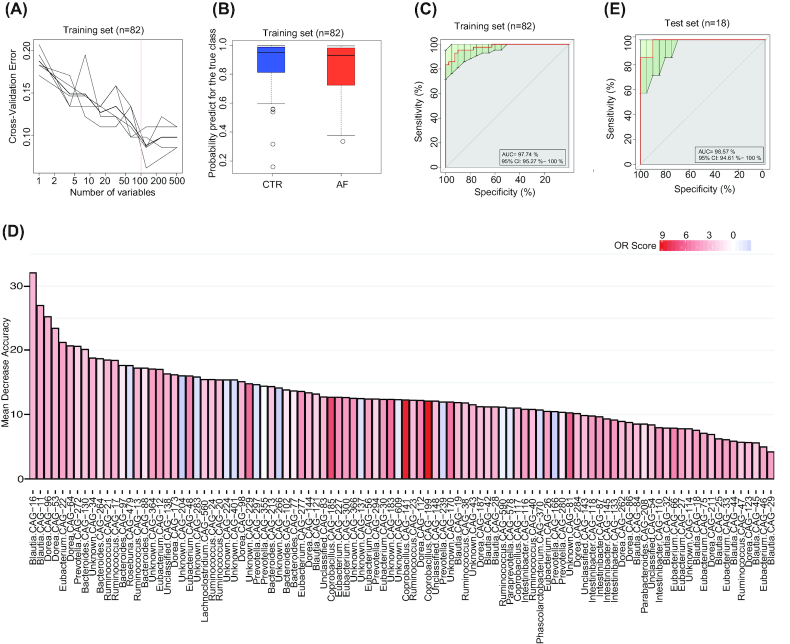
Gut CAGs distinguish AF from controls. A, The random forest disease classifier. The model was trained using the relative abundance of the CAGs in the controls and AF samples as variables. In the training set (n = 82), the distribution of 5 trials of 10-fold cross-validation error in random forest classification of AF is plotted vs the number of CAGs used in each trial. The vertical red line indicates the number of CAGs in the optimal set with the lowest cross-validation error. B, Box-and-whisker plot for the probability of AF in the cross-validation training set according to the model in A. Either the control or AF group showed a high probability for predicting the true class in the training set. C, Receiver-operating characteristic curve (ROC) for the training set. The area under the receiver operating curve (AUC) is 97.74%, and the green area indicates the 95% CI, 95.27–100%. D. The top 30 different CAGs distinguish AF from control based on the random forest model using explanatory variables of CAGs. E, ROC for the test set (n = 18). The AUC is 98.57%, and the green area indicates the 95% CI, 94.61–100%.

On the basis of the clusters of microbial CAG gene markers specific to AF, we aimed to further delineate the features of AF-associated GM and investigate the clinical value of the intestinal microbiome for distinguishing AF. Therefore, we performed a random forest disease classifier using the relative CAGs abundances as variables. With 5, 10, 20, 50, 70, and 100 CAG marker variables, the classification error remained low and relatively stable (Additional files 18: Fig. S9, Fig. 3A). The box-and-whisker plot for the probability of AF in the cross-validation training set showed that either the control or AF group showed a high probability for predicting the true class in the training set (n = 82) (Fig. 3B). As shown in Fig. 3C, the area under the receiver operating curve (AUC) was 97.74% (95% confidence interval [CI], 95.27–100%) in the training set, suggesting that patients with AF could be effectively distinguished from the controls. Consistently, the AUC for distinguishing AF from the controls was 98.57% (95% CI, 94.61–100%) in the testing set (n = 18). The CAGs that originated from *Blautia*, *Dorea*, *Eubacterium*, *Prevotella*, *Bacteroides*, *Ruminococcus*, and *Lachnospiraceae* contributed the most to discriminating AF from controls (Fig. 3D). These CAGs were significantly correlated with each other. The abundances of bacteria enriched in controls were inversely correlated with the AF group, and clustered together into a complicated network (Additional files 17: Fig. S8). So far, we have constructed a microbiota-dependent discrimination model for AF detection, and thus the values of dysbiotic GM under the AF condition should be further emphasized and uncovered.

### Aberrant microbial functions in AF populations

**Figure 4:**
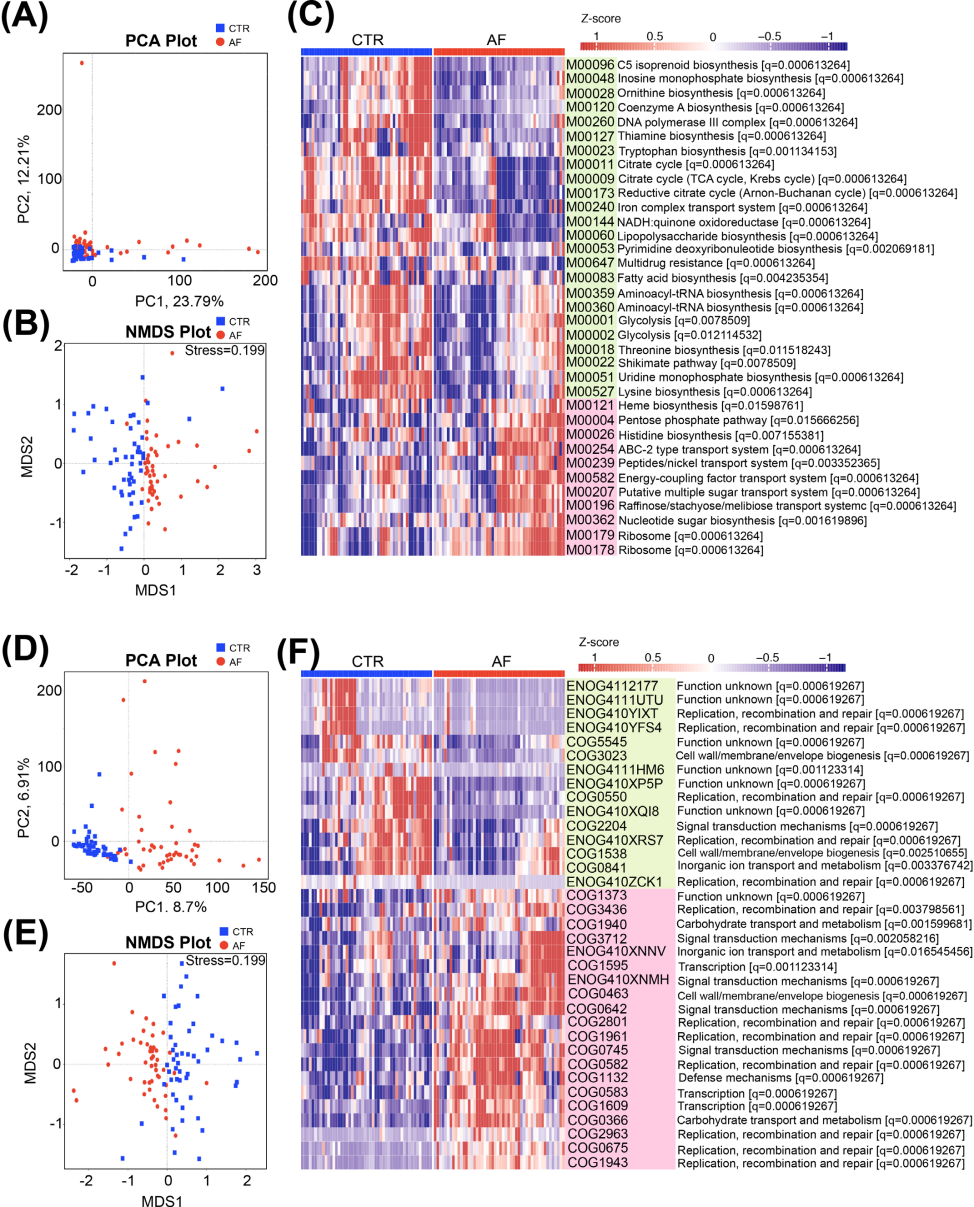
Microbial gene function annotation in AF. A and B, PCA (A) and NMDS (B) based on the relative abundance of KEGG orthology groups in 100 samples showed a significant difference between AF and control (CTR). C, The meanabundance of KEGG modules differentially enriched in the GM of controls and patients with AF. The relative abundance profiles were transformed into *z* scores by subtracting the mean abundance and dividing the standard deviation of all samples. The *z* score is negative (shown in blue) when the row abundance is lower than the mean, and red when the row abundance is higher than the mean. Overall, 24 modules enriched in control and 11 modules overrepresented in AF are shown in green and pink, respectively. The physiological effect of KEGG modules and *q* value are demonstrated on the right; Wilcoxon rank sum test. D and E, PCA (D) and NMDS (E) based on the relative abundance of eggNOG orthologs in 100 samples showed a significant difference between AF and control. F, The mean abundance of eggNOG orthologs differentially enriched in control and AF group. The relative abundance profiles were transformed into *z* scores by subtracting the mean abundance and dividing the standard deviation of all samples. The*z* score is negative (shown in blue) when the row abundance is lower than the mean, and red when the row abundance is higher than the mean. Overall, 15 eggNOGs enriched in control and 20 eggNOGs overrepresented in AF are shown in green and pink, respectively. The potential function of eggNOGs and *q* value are demonstrated on the right; Wilcoxon rank sum test.

The KEGG and evolutionary genealogy of genes: Non-supervised Orthologous Groups (EggNOG) databases were used in the present study to access the gut microbial gene functions as described previously [45, 46] (Additional files 19–21: Tables S10–S12). The AF and control groups could be separated clearly from each other by both PCA and NMDS, suggesting a significant difference of microbial functions between patients with AF and controls (*P* < 0.001, analysis of similarities, Fig. 4A, B, D, E). There were 35 KEGG modules differentially enriched among the 2 groups (adjusted *P* < 0.05, Wilcoxon rank sum test, Fig. 4C), of which, 24 modules that were decreased in the AF group were implicated in the biosynthesis of fatty acids and aminoacyl–transfer RNA (tRNA). Furthermore, genes for the iron complex transport system, nucleotide sugar biosynthesis, citrate cycle, and glycolysis were also reduced in patients with AF. These metabolic functions produce metabolites necessary for maintaining human health, and some have been indicated to be deficient in patients with HTN [9], CHF [28], or liver cirrhosis [12]. Eleven KEGG modules such as histidine biosynthesis, putative multiple sugar transport system, heme biosynthesis (glutamate to protoheme/siroheme), and the pentose phosphate pathway were found to be significantly elevated in the AF group. They were also increased in patients with colorectal adenoma-carcinoma [13], rheumatoid arthritis, T2DM, obesity, ACVD, and cirrhosis [15]. Moreover, some EggNOG orthologs enriched in the control group participate in maintaining normal human cellular functions, such as DNA replication, recombination, and repair and cell wall/membrane/envelope biogenesis. Other identified EggNOG orthologs that are enhanced in patients with AF function in signal transduction mechanisms such as carbohydrate transport and metabolism. Furthermore, we performed correlation analysis between CAGs and KEGG modules and eggNOGs (Additional files 22: Fig. S10). AF-deficient CAGs were positively correlated with some basic functions necessary for life-sustaining activities such as aminoacyl–tRNA biosynthesis and the citrate cycle. Considering these findings, the abnormal microbial functions that result from disordered GM composition in AF populations may directly lead to imbalances in metabolic profiles, resulting in disease development.

### Alterations in gut and serum metabolomics in AF

**Figure 5:**
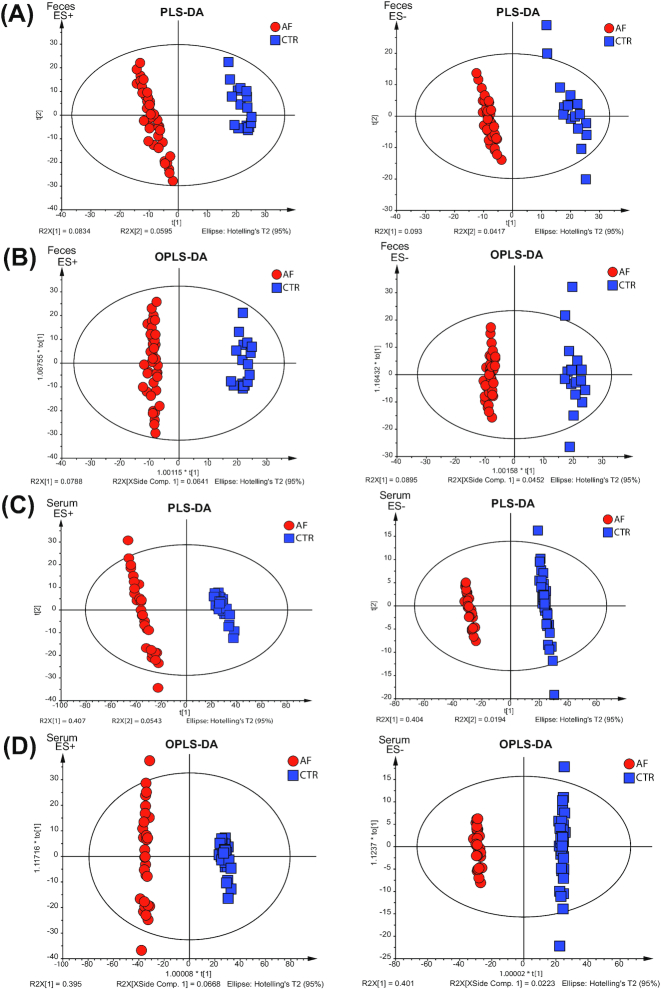
Distinguished metabolic patterns between AF and control (CTR). A. Partial least-squares discriminant analysis (PLS-DA) score plots based on the metabolic profiles in feces samples from the control and AF groups in ES+ and ES−. A clear separation between patients with AF and healthy controls was obtained under both ES+ and ES− modes. B. Score scatter plots of orthogonal PLS-DA (OPLS-SA) comparing the feces metabolic differences identify the separation between AF and control in ES+ and ES−. C, PLS-DA score plots based on the metabolic profiles in serum samples from control and AF group in ES+ and ES−, which successfully classify the 2 groups. D. Score scatter plots of OPLS-DA comparing the serum metabolic differences identify the separation between AF and control in ES+ and ES−.

Mammalian metabolism is thought to be greatly influenced by interaction with the intestinal microflora community. To explore how the host metabolic pattern alterations were affected by the gut microbiota dysbiosis in patients with AF, serum and fecal samples were collected and analyzed by high-throughput liquid chromatography–mass spectrometry (LC-MS) in both positive ion mode (ES+) and negative ion mode (ES−). A subset of 65 participants (36 controls and 29 patients with AF) from the present study were enrolled in the serum metabolic study and 59 (17 controls and 42 patients with AF) were enrolled in the feces study (Additional files 23 and 24: Tables S13 and 14). For serum, 2,548 features at ESI+ ion mode and 1,733 features at ESI− ion mode were detected. And for feces, 2,547 features at ESI+ ion mode and 1,894 features at ESI− ion mode were tested in this experiment. The partial least-squares discriminant analysis (PLS-DA) and the orthogonal partial least-squares discriminant analysis (OPLS-DA) were plotted to reveal the global metabolic changes between patients with AF and controls. For the fecal samples, a clear separation between patients with AF and healthy controls was obtained under both ES+ and ES− modes (Fig. 5A and B). The serum data recapitulated the distinction, successfully classifying the AF and control groups with PLS-DA and OPLS-DA methods (Fig. 5C and D).

Significant differentially enriched metabolites were identified on the basis of the variable importance in the projection threshold > 1 and the *P* < 0.05 and were further matched in the Metlin database. Overall, 96 serum metabolites, 46 elevated and 50 decreased, were detected in patients with AF as compared to controls (Additional files 25: Fig. S11). For the stool samples, 63 metabolites, 15 increased and 48 down-regulated, differentiated patients with AF from healthy controls (Additional files 26: Fig. S12).

**Figure 6:**
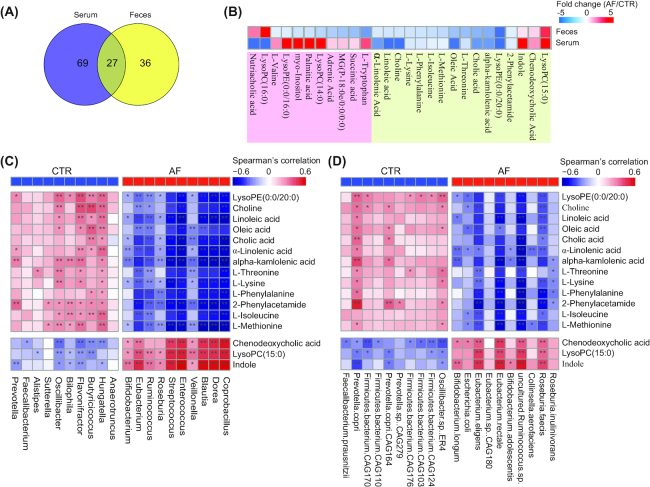
Aberrant metabolic patterns related to AF. A, Venn diagram shows the number of altered metabolites shared between serum (purple) and feces (yellow). The overlap shows that there were 27 endogenous compounds concurrently identified in both feces and serum. B, Heat map of fold change (AF/control [CTR]) of 27 compounds that were altered in both serum and stool samples of patients with AF. The fold change was transformed into *t*-scores, and the *t*-score is negative (shown in blue) when the compound showed a decline tendency in the AF group. Compounds that increased or decreased simultaneously (n = 16) or were unsynchronized (n = 11) in feces and serum are shown in green and pink, respectively. C and D, The relationship between 16 endogenous metabolites and the top 10 altered genera (C) and species (D) in AF. The 16 metabolites that were increased or decreased simultaneously in feces and serum are shown in light red and light blue, respectively. Considering the circulating metabolites that played a role during the process of GM-mediated responses, the serum data of metabonomic profiling were used in Spearman's correlation analysis. Red indicates a negative correlation, and blue, a positive correlation; single asterisk indicates *P* < 0.05, and double asterisk, *P* < 0.01. The enriched type of each genera and metabolic patterns was colored according to its direction of enrichment: blue, enriched in controls, and red, enriched in patients with AF.

Notably, 27 metabolites were altered in both serum and stool samples of patients with AF (Fig. 6A and B), 16 of which showed the same variation trend and were the focus of further investigation (Fig. 6B, Additional files 27: Table S15). These compositional changes identified AF-enriched compounds, such as chenodeoxycholic acid and lysophosphatidylcholine (lysoPC) (15:0). There were 14 metabolites with significantly decreased abundance in AF including cholic acid, oleic acid, linoleic acid (LA), and α-linolenic acid (ALA) (Fig. 6B). Chenodeoxycholic acid was able to activate the NLRP3 inflammasome in macrophages, which could primarily induce interleukin 1β and aggravates the inflammatory process and affects epithelial integrity by inducing the production of pro-inflammatory cytokines [47]. Cholic acid may influence cardiac electrophysiology, inhibiting the activity of cardiac myocytes, causing calcium overload, and leading to sudden fetal death, and hence might influence cardiac electrophysiology [48]. Furthermore, it has been reported that cholic acid could strongly reduce endoplasmic reticulum stress by inhibiting extracellular signal−regulated kinase signaling and endoplasmic reticulum stress−related activating transcription factor 4 [49]. A 20-year cohort study following >74,000 participants revealed that oleic acid consumption significantly relieved the risk for developing CVD [50]. Oleic acid prevents coronary heart disease by suppressing oxidative stress, mitigating cardiomyocyte cell damage [51]. Previous observational studies have reported that LA, the predominant μ-6 polyunsaturated fatty acid from vegetable oils and nuts, could reduce major risk factors for ACVD [52]. Increased LA intake is believed to reduce low-density lipoprotein (LDL) cholesterol, promote insulin sensitivity, and attenuate the risk of HTN [53]. These metabolic variations might aggravate or even promote the arrhythmogenic substrate aggravation in the left atrium during the pathological processes of AF.

Furthermore, some metabolites showed an increased tendency in serum but decreased in feces. These pathogenic substances might originate from a pathway other than gut microbes. For example, higher levels of circulating palmitic acid were associated with a higher risk of AF [22]. Circulating succinate, a metabolite produced by both microbiota and the host, was increased in HTN, ischemic heart disease, and T2DM [54]. Adrenic acid is an inflammation enhancer in non-alcoholic fatty liver disease [23].

To explore the association between aberrant metabolites and disordered gut microflora, we carried out a correlation analysis between the top 10 genera (Fig. 6C) and species (Fig. 6D) enriched in the AF or control groups and the 16 representative metabolites in serum or feces with similar variation tendencies. Consistently, LA and ALA, previously described as cardiovascular protectors, were negatively associated with generas such as *Flavonifractor*and*Hungatella* and species like *Prevotella copri*. ALA and LA were reported to prevent as well as terminate lysophosphatidylcholine- or acylcarnitine-induced arrhythmias [55]. The close relationship between microbes and metabolites indicates that the specific metabolites might be produced at least indirectly by corresponding gut microbes, which awaits further investigation.

Based on the significant correlation between the distinguishing metabolic features in AF and the disordered gut flora, it is possible that the gut microbiota dysbiosis induced disordered microbial functions, causing the deficiency of multiple cardiovascular-protective metabolites and thus increased susceptibility to AF.

## Discussion

In the present study we obtained seminal evidence delineating the features of AF-associated gut dysbiosis through the integration of metagenomic and metabolomic analyses. The individuals with AF exhibited significantly elevated richness and increased diversity of gut microbiota, and thus the overgrowth of bacteria may be key to the development and establishment of AF. The GM shift from an enterotype represented by *Prevotella* to *Bacteroides* further characterized an imbalanced intestinal microbial environment specific to AF. Gut bacteria such as *Faecalibacterium*, *Alistipes*, *Oscillibacter*, *Bilophila*, and *Flavonifractor* were substantially decreased in the intestinal tract in patients with AF. Inversely, *Ruminococcus*, *Streptococcus*, and *Enterococcus* were typically enriched in the AF-associated gut metagenomic composition. Metabolic profiles of both fecal and serum samples analyzed from patients with AF demonstrated significant alterations, which were correlated with gut microbiota dysbiosis. More importantly, a discriminant model based on bacterial signature profiles has been established and may have the potential to be used as biomarkers for AF in the future. It is therefore hypothesized that an increase of a specific group of gut flora may induce disordered metabolic activity of the GM, triggering the accumulation of bacterial metabolites in the circulation. This accumulation could negatively affect human health, perturbing the progression of AF, and may even play an important role in the establishment of AF. If future research confirms this hypothesis, intervention strategies targeting gut microbiota to improve the progression of AF could be pursued.

To our knowledge, the richness and diversity of the GM has been evaluated in multiple diseases, particularly in CVD, and variable findings were reported recently. In atherosclerotic disease, it was suggested that GM diversity is inversely associated with arterial stiffness in women [56], whereas a higher microbial richness and diversity in the systemic microbiome of the ST segment was correlated with elevated myocardial infarction event frequency [57]. Increased diversity of the GM was also observed in patients with stroke and transient ischemic attacks, and this dysbiosis was correlated with the severity of the disease [58]. Thus, the evaluated richness and diversity of the GM could reflect the imbalanced gut milieu, characterized by overgrowth of a variety of harmful bacteria and fewer commensal or beneficial genera. This is consistent with the present study.

A cluster of bacteria were significantly aggregated in the gut from patients with AF, including *Ruminococcus*, *Streptococcus*, and *Enterococcus*. The accrual of these microorganisms in the intestine may inhibit the growth of some bacteria that are enriched in healthy populations. For example, the decline of *Faecalibacterium*, *Alistipes*, *Oscillibacter*, *Bilophila*, and *Flavonifractor* often occurred in conjunction with changes in*Streptococcus* abundance [9, 15, 28]. It is worth noting that patients with AF shared the enrichment of numerous microbial flora, such as *Streptococcus*, *Dorea*, *Enterococcus*, and *Coprobacillus*, demonstrated in HTN [9], CHF [28], and ACVD [15]. Additionally, patients with CVDs often have decreased levels of *Faecalibacterium* and *Oscillibacter*, which are butyrate-producing species identified as important anti-inflammatory commensal bacteria [36, 59]. *Alistipes*, *Bilophila*, and *Butyricicoccus* also exhibited the same decreasing trend in AF and other CVDs, like HTN [9], CHF [28], and ACVD [15]. This group of bacterial strains is consistently altered in multiple CVDs and is therefore considered a guild emerging during the progression of disease. The aforementioned chronic CVDs might be a consequence of the imbalanced gut microbial composition associated with the establishment of this guild. Although the underlying mechanism remains largely unknown, several CVDs share some common pathophysiologic pathways, such as endothelial dysfunction [60]. Reestablishing the functionally active ecological populations as the primary ecosystem service providers is crucial to a healthier gut microbiota. Restoring the deficient gut microbes might alleviate or attenuate the disease phenotypes or progression. Targeted promotion of the gut ecosystem by individualized intervention may present a novel ecological approach for manipulating the gut microbiota to manage CVD and potentially other dysbiosis-related diseases [61].

Notably, GM of AF exhibited some unique features not displayed in other related diseases. For example, *Prevotella*, whose function is to encode superoxide reductase, phosphoadenosine phosphosulphate reductase, and favor the development of inflammation [62], showed a declined trend in AF, but overgrowth in HTN [9]. In addition, some flora that are decreased in HTN [9] exhibited a tendency to be increased in AF, CHF [28], and ACVD [15], such as *Ruminococcus, Enterococcus, Veillonella*,and *Coprococcus*. These contrasting phenomena may partly be explained by the complex and various factors involved in the pathophysiological process. To a certain extent, the generality and specificity of CVDs could be analyzed from the point of view of gut flora.

Metabolites derived by the gut microbiota, such as TMAO, have been confirmed to act on downstream cellular targets to improve or contribute to the pathogenesis of structural, metabolic, and functional cardiovascular remodeling [63]. Here, the present study revealed decreased levels of LA and ALA in patients with AF, which was consistent with the decreased function of GM in fatty acid biosynthesis. Notably, ALA/LA exerted protective effects through inhibition of reactive oxygen species generation, down-regulation of the activation of the p38 mitogen-activated protein kinase (MAPK) pathway, and the expression of transforming growth factor β1, which played a regulatory role in atrial fibrosis and contributed to the progression of AF [53]. Taken together, these findings highlight the potential and diverse physiological effects of GM-related metabolites during the progression of AF. Further studies are required to make clear the biological mechanism underlying these differential effects.

Promisingly, the microbiota-dependent discrimination model we built could distinguish AF from controls nicely based on the GM feature. Traditionally, AF can be further distinguished as paroxysmal and persistent AF on the basis of the presentation, duration, and spontaneous termination of AF episodes. The episodic pattern of paroxysmal AF is self-terminating, in most cases within 48 hours, while persistent AF is characterized as lasting longer than 7 days, including episodes that are terminated by cardioversion, with drugs, or by direct current cardioversion after ≥7 days [64]. Among our present AF cohort, there were 30 patients with paroxysmal AF and 20 patients with persistent AF. The types of AF may be partially determined by the varying extent of personalized electrical and structural remodeling in the atrial arrhythmogenic substrate. Additionally, they have different prognoses and responses to rhythm-controlling therapy and distinction between the types helps the physician and patient to make individualized therapeutic decisions [65]. Therefore, the classification of AF type based on the characteristics of gut microbiota might have clinical value, which will be explored in our future work.

Consideration of possible confounders and limitations is relevant to our study and can help to inform the design of future studies. Some of the patients with AF recruited in our cohort also had a diagnosis of HTN or T2DM. Isolated AF, driven by genetic factors, represents a minority of AF cases and the pathogenesis of AF may be an end stage of multiple metabolic and cardiovascular diseases [7, 8]. To reflect the real signature of clinical practice we did not exclude patients with comorbidities even though HTN and T2DM have been widely known to be connected with GM dysfunction. To evaluate the disordered patterns of GM resulting solely from AF, the HTN history in each group was matched individually to remove the HTN contribution. Separately, there were 12 patients with AF with T2DM, which was not adjusted between groups. We performed a PCA plot to assess the contribution of different baseline characteristics and found that PCA failed to distinguish patients with AF into separate groups based on these factors, indicating the negligible impact of sex, age, total cholesterol, or T2DM on our data. Therefore, the majority of the association of GM dysbiosis observed in the AF cohort was not mediated by HTN or T2DM. Second, although we excluded participants who used antibiotics or probiotics and confirmed the possible influences of drug use (dimethyl biguanide and statins) on gut microbiota, exercise and dietary information were not collected and corrected for in this study. Third, the conclusions drawn from our data were associations rather than causal relationships. Further studies such as gut microbiota transplantation and electrophysiological modulation testing AF inducibility are still needed. The present results provide preliminary clues and evidence for future investigations regarding the potential mechanisms between gut microbes and AF.

## Conclusions

The present study provides the first comprehensive description of the disordered patterns of gut microbiota and aberrant microbial-related metabolites in a cohort of patients with AF. These novel findings are fundamental for further studies exploring the causal relationship between AF and GM, but they are just the beginning. Extensive research is still needed to explore the clinical value of intervention strategies based on gut microbiota to improve AF conditions.

## Methods

### Study cohort

Fifty patients with nonvalvular AF were consecutively enrolled from Beijing Chaoyang Hospital and 50 individuals as matched controls were enrolled from Kailuan cohort who received biennial medical examination in Kailuan General Hospital [66]. Individuals with a history of heart failure, coronary heart disease, structural heart disease, comorbidities (inflammatory bowel diseases, irritable bowel syndrome, autoimmune diseases, liver diseases, renal diseases, or cancer), or use of antibiotics or probiotics in the past 1 month were excluded. Demographic and clinical characteristics were obtained by completing face-to-face surveys and checking hospital or medical examination records. Fifty samples from our previous work [9] regarding gut microbiota were selected by matching for the history of HTN, and the metagenomic sequencing data of 50 control stool samples from our previous study were used as controls in the present study. Among the 50 patients with AF included, fecal samples were available from each participant and used for metagenomic analyses. Metabolomic analyses were performed using serum samples from 8 patients with AF and 12 controls and stool samples from 8 patients with AF and 8 controls. The study conformed to the principles of the Declaration of Helsinki. The research protocol was approved by the ethics committee of Beijing Chaoyang Hospital and Kailuan General Hospital. All of the participants signed informed consent.

### Stool sample collection and DNA extraction

Fresh stool samples were collected from each participant, immediately frozen at −20°C, transported on ice to the laboratory, and then stored at −80°C. Bacterial DNA was extracted using TIANGEN kit (DP328, TIANGEN BIOTECH CO., Ltd, Beijing, China) at Novogene Bioinformatics Technology Co., Ltd (Beijing, China).

### Metagenomic sequencing, gene catalogue construction

Paired-end metagenomic sequencing was performed on the Illumina platform (insert size 300 bp, read length 150 bp) at the Novogene Bioinformatics Technology Co., Ltd. After quality control, the reads aligned to the human genome (alignment with Short Oligonucleotide Analysis Package 2 [SOAP2], Version 2.21, parameters: −s 135, −l 30, −v 7, −m 200, −x 400, RRID:SCR_005503) were removed and the remaining high-quality reads were used for further analysis. The assembly of reads was executed using SOAP denovo (Version 2.04, parameters: −d 1 −M 3 −R −u −F, RRID:SCR_010752). For each sample, we used a series of k-mer values (from 49 to 87) and chose the optimal one with the longest N50 value for the remaining scaffolds [12]. The clean data were mapped against scaffolds using SOAP2 (Version 2.21, parameters: −m 200 −x 400 −s 119, RRID:SCR_005503). Unused reads from each sample were assembled using the same parameters.

Gene prediction from the assembled contigs was performed using Meta GeneMark (prokaryotic GeneMark, hidden Markov model Version 2.10). A non-redundant gene catalogue was constructed with Cluster Database at High Identity with Tolerance (CD-HIT, Version 4.5.8, parameters: −G 0 −aS 0.9 −g 1 −d 0 −c 0.95.,RRID:SCR_007105) using a sequence identity cut-off of 0.95, with a minimum coverage cut-off of 0.9 for the shorter sequences. Reads were realigned to the gene catalogue with SOAP2 using parameters to determine the abundance of genes: −m 200 −x 400 −s 119. Only genes with ≥2 mapped reads were included. The gene abundance was calculated by counting the number of reads and normalizing by gene length.

### Analyses of genera richness and enterotypes

Rarefaction analysis was carried out to evaluate gene richness. Using R (Version 2.15.3, vegan package), the cohort was randomly sampled 100 times with replacement and the total number of identified genes from these samples was assessed.

Based on the genera profiles, we calculated the within-sample (α) diversity using the Shannon index to estimate the genera richness of the sample. A high α diversity denotes a high richness of genera within the sample.

By using the partitioning around medoids method based on relative abundance of genera, we analyzed the community types of each sample. As previously described [67], we estimated the optimal number of clusters using the CH index. Genera with a mean relative abundance ≥10^−4^ and present in ≥6 samples would be used in the analysis. The genera in enterotype 1 were clustered according to the Spearman's correlation between genera abundances, and their co-occurrence network was visualized using Cytoscape (Version 3.2.1, RRID:SCR_003032).

### Taxonomic assignment, annotation, and abundance profiling

Genes were aligned to the integrated nr database to assess the taxonomic assignment by using DIAMOND (Version 0.7.9.58, default parameters except that −k 50 −sensitive −e 0.00001, RRID:SCR_016071). To distinguish taxonomic groups, the significant matches for each gene, defined by e-values ≤10 × e-value of the top hit, were determined and the retained matches were used as previously described [68]. The taxonomical level of each gene was determined using the lowest common ancestor−based algorithm implemented with MEGAN (MEtaGenome ANalyzer, RRID:SCR_011942). The abundance of a taxonomic group was calculated by summing the abundance of genes annotated to a feature.

### CAGs and co-occurrence network

As previously described [69, 70], we compared the abundance of each gene across groups to identify the marker genes associated with AF. On the basis of their abundance variation across groups, these marker genes were clustered into groups [34]. CAGs were defined as clusters with >50 genes [9, 12, 70]. CAG abundance profiles were calculated on the basis of the average gene depth signal and weighted by gene length. Taxonomic assignment of the CAGs was performed on the basis of the taxonomy of tracer genes, as previously described [9, 10]. All genes from 1 CAG were aligned to the reference microbial genomes at the nucleotide level (by BLASTN) and the NCBI-nr database at the protein level (by BLASTP). The alignment hits were filtered by both the e-value (<1 × 10^–5^ at the nucleotide level and <1 × 10^–5^ at the protein level) and the alignment coverage (>70% of a query sequence). From the alignments with the reference microbial genomes, we obtained a list of well-mapped bacterial genomes for each CAG and ordered these bacterial genomes according to the proportion of genes that could be mapped onto the bacterial genome, as well as the average identity of the alignments. The species assignment required 90% of the genes in a CAG to match the species’ genome with 95% identity and 70% overlap of query. The CAG assignment to a genus required 80% of its genes to align to the genome with 85% identity in both DNA and protein sequences.

The enriched CAGs were identified and clustered according to Spearman's correlation, and the co-occurrence network was visualized by Cytoscape (Version 3.2.1; RRID:SCR_003032). Based on the abundance in the set of compared samples, an odds ratio score [69] was calculated for each CAG and for the comparative analysis between control and AF samples; the AF-associated CAGs were identified as AF-enriched (odds ratio > 2) or AF-depleted (odds ratio < 0.5).

### Functional annotation

Using DIAMOND (Version 0.7.9.58, default parameters except for –k 50 –sensitive –e 0.00001), all genes in the catalogue were aligned to the KEGG database (Release 73.1, with animal and plant genes removed) and to the eggNOG database (v4.5 via eggNOG-mapper with hidden Markov model search mode). Each protein was assigned to the KEGG and eggNOG orthologs using the highest scoring annotated hits containing ≥1 high-scoring segment pair scoring >60 hits. By summing the abundance of genes annotated to the same feature, the abundance of KEGG ortholog/module was calculated.

### Metabolomic analysis based on LC-MS

In preparation for extraction, 50-mg fecal samples were pipetted into centrifuge tubes (1.5 mL). The protein was precipitated with 800 μL of methanol and 10 μL of internal standard (2.9 mg/mL, DL-*o*-chlorophenylalanine) was added. The samples were ground at 65 kHz for 90 s and centrifuged at 12,000 rpm for 15 min at 4°C. Then 200 μL of the supernatant was transferred into a vial for further analysis. The serum samples were thawed at room temperature and 100 μL was pipetted into centrifuge tubes (1.5 mL) in preparation for extraction. The protein was precipitated with 300 μL of methanol, and 10 μL of internal standard (2.9 mg/mL, DL-*o*-chlorophenylalanine) was added. The samples were vortexed for 30 s and centrifuged at 12,000 rpm for 15 min at 4°C. Then 200 μL of the supernatant was transferred to a vial for further analysis. The fecal and serum metabolic profiles were performed on an LC-MS platform (Thermo Fisher Scientific, Ultimate 3000LC, Orbitrap Elite) using a Hypergod C18 (100 × 4.6 mm × 3 μm) column. The chromatographic separation conditions were as follows: column temperature, 40°C; flow rate, 0.3 mL/min; mobile phase A, water +0.1% formic acid; mobile phase B, acetonitrile +0.1% formic acid; injection volume, 4 mL; automatic injector temperature, 4°C.

For both fecal and serum samples the following conditions were used for the positive ion mode (ES+): heater temperature, 300°C; sheath gas flow rate, 45 arb (arbitrary units); auxiliary gas flow rate, 15 arb; sweep gas flow rate, 1 arb; spray voltage, 3.0 kV; capillary temperature, 350°C; S-lens RF level, 30%. The following conditions were used for negative ion mode (ES−): heater temperature, 300°C; sheath gas flow rate, 45 arb; auxiliary gas flow rate, 15 arb; sweep gas flow rate, 1 arb; spray voltage, 3.2 kV; capillary temperature, 350°C; S-lens RF level, 60%.

All metabolomic data were prepared for feature extraction and preprocessed with Compound Discoverer 2.0 software (Thermo Fisher Scientific). Data were normalized at the start. Considering that remarkable differences existed among various metabolites, some signals of metabolites with too high or low concentration might be covered up and failed to be identified as biomarkers. So, normalization, aiming to adjust the weight of different variables to decrease the gap of different signals, should be performed to bring the dimensions (e.g., mean and standard deviation) of all variables to a similar level and make the data more comparable. The calculation process was to normalize the peak area of each sample to 1,000,000 and divide the peak area of each ion by the total peak area of the sample and multiplied by 1,000,000. Data were then edited into a 2D data matrix by Excel 2010 software, using retention time (RT), compound molecular weight (compMW), observations (samples), and peak areas. Using SIMCA-P software (Umetrics AB, Umea, Sweden), a multivariate analysis was performed. Compounds were significantly distinguished between groups, identified by a variable influence on projection > 1 and *P* < 0.05 based on the peak areas. The exact molecular mass, ppm (<25), and tandem mass spectrometry value of these compounds was used to identify the metabolites related to the featured peak in the Metlin database [71]. Furthermore, we compared the mass spectrum. The score value that indicated the matching rate was calculated by Compound Discoverer 2.0 software (Thermo Fisher Scientific) with a maximum of 100. For metabolites detected in both ES+ and ES−, the data in the mode with the lower *P*-value was retained for further analysis.

### Statistical analysis

Quantitative demographic and clinical characteristic data with normal distributions were presented as mean and standard deviation, and the *t*-test was used for between-group comparisons. Quantitative data with non-normal distributions were presented as median (first quartile, third quartile), and the Wilcoxon rank sum test was performed for between-group comparisons. Qualitative data were presented as a percentage, and the χ^2^ test was used for between-group comparisons. All statistical tests were 2 sided, and *P* < 0.05 was regarded as significant. Statistical analyses were performed with SPSS, Version 22.0 (IBM Corp., Armonk, New York)

The Shannon index at the genus level was calculated with QIIME (Quantitative Insights Into Microbial Ecology, Version 1.7.0, RRID:SCR_008249). PCA was performed using the Facto MineR package in R software (Version 2.15.3), while principal coordinate analysis was performed by using the ade4 package, cluster packages, fpc packages, and cluster Sim package in R software (Version 2.15.3). Partial least-squares−based structural equation modeling analysis was conducted using the Smart-PLS 3 software. PLS-DA was carried out using the SIMCA-P software to cluster sample plots across groups.

Differential abundance of genes, genera, and KEGG orthology modules was tested on the basis of the Wilcoxon rank sum test, and *P* values were corrected for multiple testing with the Benjamini and Hochberg method. Genera with an average relative abundance ≥10^−4^ and presence in ≥6 participants were included in the analyses.

Based on the profiles of CAGs, the samples were randomly divided into training and test sets. A random forest classifier was trained on 80% of the data and tested on the remaining 20% of our data using the random forest package in R. We performed a 10-fold cross-validation within the training set to evaluate the performance of the predictive model and obtain more precise curves. The cross-validation error curves (average of 10 test sets each) from 5 trials of the 10-fold cross-validation were averaged. Variable importance was calculated for the random forest models using the full set of features determined by mean decrease in accuracy. At the lowest cross-validation error, the number of variables was 1,000. Therefore, the predictive model was constructed using the 1,000 most important variables, and the performance was assessed using ROC analysis. The 95% confidence interals for the ROC curves were calculated using the pROC R package. The performance of the smaller models was measured as the AUC when applied to the test set.

## Availability of supporting data and materials

The data supporting the results of this article have been deposited in the EMBL European Nucleotide Archive (ENA) under the BioProject accession code PRJEB28384. The metabolomics data are available at the NIH Common Fund's Data Repository and Coordinating Center website with Metabolomics Workbench Study ID: ST001168 (for fecal metabolomic analyses) and ST001169 (for serum metabolomic analyses). Other data further supporting this work are available in the *GigaScience* repository, GigaDB [72].

## Additional files

**Additional files 1**: Table S1, Data production of 100 samples in control and AF

**Additional files 2**: Fig. S1, Increased Firmicutes/Bacteroidetes ratio, Pielou evenness, and Chao richness in AF

**Additional files 3**: Fig. S2, Another 12 genera significantly enriched in enterotype 1

**Additional files 4**: Fig. S3, Enterotype analysis at the species level

**Additional files 5**: Fig. S4, Taxonomic annotation and abundance profiling

**Additional files 6**: Table S2, Relative abundance profile at the phylum level

**Additional files 7**: Table S3, Relative abundance profile at the genus level

**Additional files 8**: Table S4, Detailed information of differential genera

**Additional files 9**: Fig. S5, Species strikingly different across groups

**Additional files 10**: Fig. S6, Influence of baseline characteristics, including age, sex, T2DM, total cholesterol, and medication use on GM

**Additional files 11**: Table S5, Detailed information for 121,145 gene markers

**Additional files 12**: Table S6, Reference genomes for CAG taxonomy assignment

**Additional files 13**: Table S7, Detailed information of enriched CAGs in different groups

**Additional files 14**: Table S8, Detailed information of 477 CAGs

**Additional files 15**: Table S9, Spearman's correlation between enriched CAGs

**Additional files 16**: Fig. S7, Size distribution and taxonomic assignment of CAGs

**Additional files 17**: Fig. S8, The network of CAGs enriched in AF compared with controls

**Additional files 18**: Fig. S9, Gut CAGs (variables in 5, 10, 20, 50, 70) distinguish AF from controls

**Additional files 19**: Table S10, Detailed information of differential KEGG modules

**Additional files 20**: Table S11, Detailed information of differential KEGG orthologs

**Additional files 21**: Table S12, Detailed information of differential eggNOG family

**Additional files 22**: Fig. S10, Correlation between CAGs and altered function module

**Additional files 23**: Table S13, Clinical characteristics of participants in serum metabolism

**Additional files 24**: Table S14, Clinical characteristics of participants in fecal metabolism

**Additional files 25**: Fig. S11, Metabolites differentially enriched in AF and controls in serum

**Additional files 26**: Fig. S12, Metabolites differentially enriched in AF and controls in feces

**Additional files 27**: Table S15, Detailed information of 16 metabolites differently enriched across groups

**Additional files 28**: The computational code of step by step for bioinformatic analysis

## Abbreviations

ACVD: atherosclerotic cardiovascular disease; AF: atrial fibrillation; ALA: α-linolenic acid; arb: arbitrary unit; AUC: area under the receiver-operating curve; bp: base pairs; CAG: co-abundance gene group; CHF: congestive heart failure; CVD: cardiovascular disease; EggNOG: evolutionary genealogy of genes: Non-supervised Orthologous Groups; ES−: negative ion mode; ES+: positive ion mode; Gb: gigabase pairs; GM: gut microbiome; HTN: hypertension; KEGG: Kyoto Encyclopedia of Genes and Genomes; LA: linoleic acid; LC-MS: liquid chromatography–mass spectrometry; LDL: low-density lipoprotein; lysoPC: lysophosphatidylcholine; lysoPE: lysophosphatidylethanolamine; MG: monoglyceride; MAPK: mitogen-activated protein kinase; NCBI: National Center for Biotechnology Information; NIH: National Institutes of Health; NMDS: non-metric dimensional scaling; nr: nonredundant; OPLS-DA: orthogonal partial least-squares discriminant analysis; PLS-DA: partial least-squares discriminant analysis; PCA: principal component analysis; PC: principal component; PCoA: principal coordinate analysis; ROC: receiver-operating characteristic curve; RF: radio frequency; T2DM: type 2 diabetes mellitus; TMAO: trimethylamine *N*-oxide; tRNA: transfer RNA.

## Ethics approval and consent to participate

The research protocol was approved by the ethics committee of Beijing Chaoyang Hospital and Kailuan General Hospital. All of the participants signed informed consents.

## Competing interests

The authors declare that they have no competing interests.

## Supplementary Material

giz058_GIGA-D-18-00364_Original_Submission

giz058_GIGA-D-18-00364_Revision_1

giz058_GIGA-D-18-00364_Revision_2

giz058_Response_to_Reviewer_Comments_Original_Submission

giz058_Response_to_Reviewer_Comments_Revision_1

giz058_Reviewer_1_Report_Original_Submission -- Intawat Nookaew 11/20/2018 Reviewed

giz058_Reviewer_1_Report_Revision_1 -- Intawat Nookaew 4/9/2019 Reviewed

giz058_Reviewer_2_Report_Original_Submission -- Zeneng Wang 11/25/2018 Reviewed

giz058_Reviewer_2_Report_Revision_2 -- Zeneng Wang 3/23/2019 Reviewed

giz058_Reviewer_3_Report_Original_Submission -- Hein Min Tun 12/10/2018 Reviewed

giz058_Supplement_Files
