# Supplementary material for: Disordered gut microbiota and alterations in metabolic patterns are associated with atrial fibrillation
Source: Gigascience. 2019 May 30;8(6):giz058. doi: 10.1093/gigascience/giz058 (PMC6543127; doi:10.1093/gigascience/giz058)
Supplement: giz058_GIGA-D-18-00364_Original_Submission [file giz058_giga-d-18-00364_original_submission.pdf]

GigaScience

Disordered gut microbiota and alterations in metabolic patterns are associated with atrial fibrillation

--Manuscript Draft--

**Manuscript Number:**	GIGA-D-18-00364
**Full Title:**	Disordered gut microbiota and alterations in metabolic patterns are associated with atrial fibrillation
**Article Type:**	Research
**Funding Information:**	National Natural Science Foundation of China (81670214) — Dr. Xinchun Yang<br>National Natural Science Foundation of China (81500383) — Dr. Jing Li<br>National Natural Science Foundation of China (81870308) — Dr. Jing Li<br>National Natural Science Foundation of China (81770253) — Dr. Jiuchang Zhong<br>National Natural Science Foundation of China (81370362) — Dr. Jiuchang Zhong<br>Beijing Natural Science Foundation (7172080) — Dr. Xinchun Yang<br>Beijing Municipal Administration of Hospitals' Youth Programme (QML20170303) — Dr. Jing Li<br>1315 personnel training plan (CYMY-2017-03) — Dr. Xinchun Yang

Abstract:

Background: With the establishment of the heart-gut axis concept, accumulating studies suggest that the gut microbiome plays an important role in the genesis of cardiovascular diseases. Yet, little evidence has been reported characterizing the gut microbiota shift in atrial fibrillation.
Methods and Results: We include the first result of the global alterations that occur in the intestinal microbiota based on a strategy of metagenomic and metabolomic analyses in a cohort of 50 atrial fibrillation patients and 50 matched controls. These alterations include a dramatic elevation in microbial diversity, and a specific perturbation of gut microbiota composition. Overgrowths of Ruminococcus, Streptococcus and Enterococcus, and reductions of Faecalibacterium, Alistipes, Oscillibacter, and Bilophila were detected in atrial fibrillation patients. A gut microbial function imbalance and correlated metabolic pattern changes were observed with atrial fibrillation in both fecal and serum samples. The differential gut microbiome signatures could be used to identify atrial fibrillation patients.
Conclusion: Our findings characterize the disordered gut microbiota and microbial metabolite profiles in atrial fibrillation. Intervention strategies targeting microbiome composition to counteract the progression of atrial fibrillation are highly suggested.

Keywords: Atrial fibrillation, Gut microbiota, Metagenome, Metabolism

**Corresponding Author:**	Xinchun Yang<br><br>CHINA
**Corresponding Author Secondary Information:**	
**Corresponding Author's Institution:**	
**Corresponding Author's Secondary Institution:**	
**First Author:**	Kun Zuo

First Author Secondary Information:	
Order of Authors:	Kun Zuo
	Kuibao Li
	Chaowei Hu
	Yuanfeng Gao
	Mulei Chen
	Roumu Hu
	Ye Liu
	Hongjie Chi
	Hongjiang Wang
	Yanwen Qin
	Xiaoyan Liu
	Shichao Li
	Jiuchang Zhong
	Jun Cai
	Jing Li
	Xinchun Yang
Order of Authors Secondary Information:	
Additional Information:	
**Question**	**Response**
Are you submitting this manuscript to a special series or article collection?	No
**Experimental design and statistics**<br><br>Full details of the experimental design and statistical methods used should be given in the Methods section, as detailed in our Minimum Standards Reporting Checklist. Information essential to interpreting the data presented should be made available in the figure legends.<br><br>Have you included all the information requested in your manuscript?	Yes
**Resources**<br><br>A description of all resources used, including antibodies, cell lines, animals and software tools, with enough information to allow them to be uniquely identified, should be included in the	Yes

Methods section. Authors are strongly encouraged to cite Research Resource Identifiers (RRIDs) for antibodies, model organisms and tools, where possible.<br><br>Have you included the information requested as detailed in our Minimum Standards Reporting Checklist?	
**Availability of data and materials**<br><br>All datasets and code on which the conclusions of the paper rely must be either included in your submission or deposited in publicly available repositories (where available and ethically appropriate), referencing such data using a unique identifier in the references and in the “Availability of Data and Materials” section of your manuscript.<br><br>Have you have met the above requirement as detailed in our Minimum Standards Reporting Checklist?	Yes

Main Manuscript
Click here to download Manuscript article from Kun Zuo GS.docx

Disordered gut microbiota and alterations in metabolic patterns are associated with atrial fibrillation

Kun Zuo[1], Kuibao Li[1], Chaowei Hu[4], Yuanfeng Gao[1], Mulei Chen[1], Roumu Hu[1], Ye Liu[1], Hongjie Chi[1], Hongjiang Wang[1], Yanwen Qin[4], Xiaoyan Liu[2], Shichao Li[1], Jiuchang Zhong[1], Jun Cai[3], Jing Li[1*], Xinchun Yang[1*].

[1]*Heart Center & Beijing Key Laboratory of Hypertension, Beijing Chaoyang Hospital, Capital Medical University, Beijing 100020, China*

[2]*Medical Research Center, Beijing Chaoyang Hospital, Capital Medical University, Beijing 100020, China*

[3]*Hypertension Center, Fuwai Hospital, State Key Laboratory of Cardiovascular Disease of China, National Center for Cardiovascular Diseases of China, Chinese Academy of Medical Sciences and Peking Union Medical College, Beijing 100037, China*

[4]*The Key Laboratory of Upper Airway Dysfunction-related Cardiovascular Diseases, Beijing An Zhen Hospital, Capital Medical University, Beijing Institute of Heart, Lung and Blood Vessel Diseases, Beijing 100029, China*

Author email addresses

Kun Zuo, zuokun699@163.com

Kuibao Li, kuibaoli@126.com

Chaowei Hu, halohu123@sina.com

Yuanfeng Gao, gaoyuanwind1@163.com

Mulei Chen, cml68@sina.cn

Roumu Hu, roumuhu@126.com
Ye Liu, liuye8810@sina.com
Hongjie Chi, chihongjie@163.com
Hongjiang Wang, wanghongjiang@126.com
Yanwen Qin, qinyanwen@vip.126.com
Xiaoyan Liu, lxy-213@163.com
Shichao Li, lishichao3@sina.com
Jiuchang Zhong, jiuchangzhong@aliyun.com
Jun Cai, caijun@fuwaihospital.org
Jing Li, lijing11999@126.com
Xinchun Yang, yxc6229@163.com

***Correspondence to:**

Xinchun Yang, MD, PhD
Heart Center, Beijing ChaoYang Hospital, Capital Medical University,
Beijing Key Laboratory of Hypertension,
8th Gongtinanlu Rd, Chaoyang District, Beijing, China, 100020
Tel: 86-10-85231937
Fax: 86-10-85231937
E-mail: yxc6229@163.com

Jing Li, MD, PhD
Heart Center, Beijing ChaoYang Hospital, Capital Medical University,
Beijing Key Laboratory of Hypertension,

8th Gongtinanlu Rd, Chaoyang District, Beijing, China, 100020

Tel: 86-10-85231937

Fax: 86-10-85231937

E-mail: lijing11999@126.com

Abstract

Background: With the establishment of the heart-gut axis concept, accumulating studies suggest that the gut microbiome plays an important role in the genesis of cardiovascular diseases. Yet, little evidence has been reported characterizing the gut microbiota shift in atrial fibrillation.

Methods and Results: We include the first result of the global alterations that occur in the intestinal microbiota based on a strategy of metagenomic and metabolomic analyses in a cohort of 50 atrial fibrillation patients and 50 matched controls. These alterations include a dramatic elevation in microbial diversity, and a specific perturbation of gut microbiota composition. Overgrowths of *Ruminococcus*, *Streptococcus* and *Enterococcus*, and reductions of *Faecalibacterium*, *Alistipes*, *Oscillibacter*, and *Bilophila* were detected in atrial fibrillation patients. A gut microbial function imbalance and correlated metabolic pattern changes were observed with atrial fibrillation in both fecal and serum samples. The differential gut microbiome signatures could be used to identify atrial fibrillation patients.

Conclusion: Our findings characterize the disordered gut microbiota and microbial metabolite profiles in atrial fibrillation. Intervention strategies targeting microbiome composition to counteract the progression of atrial fibrillation are highly suggested.

Keywords: Atrial fibrillation, Gut microbiota, Metagenome, Metabolism

Background

Atrial Fibrillation (AF), an abnormal heart rhythm characterized by the rapid and irregular beating of the atria, is the most common arrhythmia with heavy global burdens, intensifying disability and morbidity. In Europe and the US, one in four middle-aged adults will develop AF [1,2]. AF is prevalent in approximately 3% of adults 20 years or older [3], with greater prevalence in older people [4] and in patients with conditions such as hypertension (HTN), heart failure, obesity and type two diabetes mellitus (T2DM) [5]. Decreased quality of life is common in AF patients and between 10–40% of AF patients are hospitalized each year [6]. AF is independently associated with a 2-fold increased risk of all-cause mortality in women and a 1.5-fold increase in men [7,8] and has become a significant contributor to cardiovascular events leading to cardiac death worldwide [9]. Currently, ideal preventive and therapeutic strategies to counteract the progression of AF remain sparse. The heterogeneity of underlying atrial substrate, extent of atrial fibrosis, and the discrepancies between inter-individual electrophysiological characteristics contribute to unpredictable responses to drug or ablation therapy [10]. For example, in pure focal AF patients without overlapping atrial fibrosis, triggers isolation could be considered as a curative treatment; however, it may not be enough for fibrotic atrial cardiomyopathy without stepwise individualized substrate modification. Therefore, efforts to identify the pathological mechanisms of AF are warranted. Various genetic mutations have been associated with AF [11] and environmental or unhealthy lifestyle factors are also believed to contribute to the

development of AF [12]. It is essential to embrace AF prevention as a priority, not only focusing on rate, rhythm controlling or stroke prevention but also considering AF as a concomitant factor of adverse atrial remodeling rather than a solitary disease [13]. It was worth noting that AF risk factors or contributors, such as HTN, T2DM and obesity have been linked to dietary intake that possibly contributes to alterations in the composition of the gut microbiota (dysbiosis) [12,14-16].

Recently, more investigators have focused on the role of the gut microbiome (GM), which has been identified as an essential factor affecting human health [14-19]. Dysbiotic GM has been reported in multiple diseases, such as T2DM [15], obesity [16], HTN [14], atherosclerotic cardiovascular disease [20], liver cirrhosis [17], colorectal adenoma-carcinoma [18], rheumatoid arthritis [19], irritable bowel syndrome [21], anxiety and depression [22], and shown to activate the immune system [23], eliciting chronic diseases. As the understanding of the relationship between intestinal microbiome and diseases has deepened, possible underlying mechanisms have been proposed. For example, emerging evidence suggests that through immune system and metabolic alterations, gut microbiota disequilibrium could induce obesity, HTN and T2DM, traditional cardiac risk factors that play essential roles during atrial remodeling in the development of AF [12, 24]. However, data demonstrating a correlation between AF and the intestinal microbiome are still lacking. To our knowledge, studies of gut microbiota and AF have been few in number and limited to canines. Information regarding the impact of microbial metabolites is also incomplete. A gut microbial-

dependent metabolite, trimethylamine-N-oxide (TMAO), which is positively correlated with cardiovascular disease in humans, is proatherogenic and could increase the instability of atrial electrophysiology [25]. However, it remains unclear whether circulating TMAO levels derived from the intrinsic microbiome can reach the ganglionated plexi and create local concentrations sufficient to result in comparable arrhythmogenic effects. In addition, recent studies have shown that gut-derived lipopolysaccharide (LPS) is predictive for major adverse cardiovascular events in AF patients [26]. Furthermore, microbiome-derived free fatty acids, such as palmitic (PA), stearic (SA) and oleic (OLA), might have potential influences on arrhythmogenesis [27].

These seminal studies provided the first clues indicating a possible interaction between gut microbiota and AF. They encouraged us to identify direct evidence of gut bacteria is alterations in patients with AF and evaluate the possible contribution of gut dysbiosis to aberrant metabolic patterns that accelerate the progression of AF. We performed metagenomic sequencing analyses of stool samples from patients with AF to outline the potential compositional and functional alterations of GM. In addition to exposing the relationship between disordered GM and altered metabolomic profiles in AF, we aimed to construct a microbiota-dependent discrimination index for distinguishing AF, thus providing a comprehensive understanding of gut microbiota dysbiosis in the progression of AF. This work is fundamental for further studies to

reveal the causal relationship and explore preventative measures for postponing AF progression.

Results

Baseline characteristics of the study cohort

We enrolled 100 Chinese participants consisting of 50 patients with nonvalvular AF and 50 individuals as matched controls (CTR). AF was diagnosed using an electrocardiogram and defined as the absence of P waves, replaced by disorganized electrical activity and irregular R–R intervals due to irregular conduction of impulses to the ventricles [33]. To adjust for the effect of HTN on gut microbiota composition, we selected 50 samples from our previous gut microbiota work matched for a history of HTN. None of the subjects had heart failure, coronary heart disease, structural heart disease, inflammatory bowel diseases, irritable bowel syndrome, autoimmune diseases, liver diseases, renal diseases or cancer. Patients who had used antibiotics or probiotics in the last month were excluded. Of note, some AF patients reported medication use including angiotensin converting enzyme inhibitors (n=7), angiotensin receptor blockers (n=4), β receptor blockers, statins (n=4), aspirin (n=2), amiodarone (n=10), dimethyl biguanide (DMBG) (n=6) and/or oral anticoagulation therapy (n=13). The clinical characteristics of all subjects are shown in Table 1. There was no significant difference between AF patients and controls in terms of body mass index, creatinine, total bilirubin or glutamic-pyruvic transaminase. Most of the patients were elderly, with

70% greater than 60 years old. For the control group, there were more males than females, with males accounting for 82%. Although the total cholesterol (TC) serum levels were much lower in patients with AF, these clinical indices were all within the normal range.

Elevated microbiota richness and altered community types in the gut of subjects with AF

Whole-metagenome shotgun sequencing of the 100 stool samples from our study cohort was performed. A total of 612.84 Gb high-quality sequencing reads were generated (6.13 ± 0.96 (s.d.) million reads per sample on average) (Additional files 1: Table S1). Rarefaction analyses, performed as we previous described [14], showed that the curves approached saturation in each group and with a significantly increased gene number in the microbiomes of patients with AF (Fig. 1a). We also compared the gene count, within-sample diversity (Shannon index) and 3 other ecological parameters, including Chao richness, Pielou evenness and Firmicutes/Bacteroidetes ratio (F/B ratio) among controls and AF patients. Consistently, gut microbial richness (gene count), diversity in AF group were much higher ($P = 0.007$ for gene count, Fig. 1b; $P = 3.53e-05$ for Shannon index, Fig. 1c; $P=7.162e-05$ for F/B ratio, Additional files 2: Fig. S1a; $P=0.007633$ for Chao richness, Fig. S1b; $P=4.262e-06$ for Pielou evenness, Fig. S1c). The elevated richness of genes or genera observed in our cohort may suggest the overgrowth of a variety of harmful bacteria in patients with AF.

To investigate the shift of gut microbiota community structure affected by AF state, microbial enterotype features were examined using the Partitioning Around Medoid clustering method. The 100 samples were divided into two clusters by Principal Coordinate Analysis (PCoA) based on the Jensen-Shannon divergence (Fig. 1d). Enterotype 1 was dominated by *Bacteroides* as the most enriched genus, and *Prevotella* was the core in enterotype 2 ($P = 1.730774e-09$ and $P = 4.376078e-14$, respectively; Wilcoxon rank sum test, Fig. 1e). Both enterotypes have been previously reported in HTN, T2DM, colorectal cancer and irritable bowel syndrome [14,15,18,21]. There were 12 other significantly increased genera in enterotype 1, including *Blautia*, *Coprobacillus*, *Dorea*, *Enterococcus*, *Streptococcus* and *Veillonella* (Additional files 3: Fig. S2). Interestingly, there was a dysbiosis of enterotype distribution by AF conditions. For the control group, the percentage of samples in both enterotypes was almost the same (50% in enterotype 1, 50% in enterotype 2), whereas a higher percentage of AF patients were found to be distributed in enterotype 1 (82%), and less in enterotype 2 ($P = 0.001$, AF vs CTR; Fisher's exact test; Fig. 1f). Therefore, a morbid state of AF is associated with imbalanced gut microbial communities, with a tendency towards the enterotype dominated by *Bacteroides* and away from the *Prevotella* prominent enterotype.

Taxonomic profile of AF-associated gut microbiota

In order to compare the taxonomic profile of gut microbiota in AF patients with those

in healthy individuals, we accessed the GM abundances and phylogenetic profiles at the genus level. Genes were aligned to the NR database using DIAMOND61 (Version 0.7.9.58) and annotated to taxonomic groups (Additional files 4: Fig. S3). The relative abundance of gut microbes was calculated by summing the abundance of genes as listed in Additional files 5-6: Table S2-S3. P values were tested using the Wilcoxon rank sum test and corrected for multiple testing with the Benjamin & Hochberg method [17]. The 35 most abundant genera in AF patients and healthy controls are shown in Fig. S3c. The state of disease significantly separated the subjects with AF or without AF in principal component analysis (PCA) analysis or in non-metric dimensional scaling (NMDS) analysis at the genus level. Overall, 574 genera were dramatically different in control and AF subjects ($P < 0.05$, Wilcoxon rank sum test, Additional files 7: Table S4). And consistent results were also obtained when the PCA analysis was performed based on the genera or species differentially enriched across groups ($P < 0.05$, Anosim, genus: Fig.2a, species: Additional files 8: Fig. S4a). The top 10 gut bacteria that dominated in AF or controls at the genus level are shown in Fig. 2c, d. In AF patients, the proportion of *Streptococcus*, *Enterococcus*, *Blautia*, *Dorea*, *Veillonella* and *Coprobacillus* were much higher than in controls (Fig. 2c), in agreement with our previous observations that they were more abundant in the AF-correlated enterotype (enterotype 1). In addition to *Eubacterium, Bifidobacterium and Roseburia*, *Ruminococcus* were also overexpressed in individuals with AF (Fig. 2c). *Ruminococcus* is known to possess a pro-inflammatory property, which was is implicated in the

development of inflammatory bowel disease [34-36]. Transplantation of *Ruminococcus* into germfree mice enhanced the levels of interforon-γ, interleukin-17 and interleukin-22 [35]. *Streptococcus*, recognized as a morbific oral bacteria, has also been demonstrated to be elevated in HTN [14], congestive heart failure (CHF) [37] and atherosclerotic cardiovascular disease (ACVD) [20,38]. Furthermore, *Veillonella*, a Gram-negative anaerobic coccus, was suggested to be inversely correlated with cardiovascular protective metabolites such as niacin, cinnamic acid and orotic acid [39]. In addition, *Enterococcus* is known to produce cytolysin, a toxin that causes rupture of a variety of target membranes, including bacterial cells, erythrocytes and other mammalian cells [40]. Of the top 10 species in AF group shown in Fig. S4c, *Escherichia coli*, a potentially pathogenic bacteria, was the most abundant and may be correlated with the progression of AF. It is speculated that these clusters of conditioned pathogens accumulated in the gut might antagonize AF susceptibility.

Moreover, *Faecalibacterium*, *Prevotella*, *Alistipes*, *Oscillibacter* and *Sutterella* were dramatically decreased in the AF patient group compared with controls and a similar shift was found for *Butyricicoccus, Flavonifractor* and *Bilophila* (Fig. 2d). In addition, we also identified a dramatic decline of species such as *Faecalibacterium prausnitzii*, *Oscillibacter sp.*, and also *Firmicutes bacterium* in the AF patient group (Fig. S4d). *Faecalibacterium prausnitzii* is a butyrate-producing commensal bacterium with anti-inflammatory properties and its deficiency may aggravate chronic inflammation, leading to ulcerative colitis, Crohn's disease, obesity, asthma and major

depressive disorder [41-44]. *Alistipes* is a common member of the human intestinal microbiota, capable of producing short chain fatty acids from amino acids, such as succinic and acetic acids [45]. The enrichment of *Oscillibacter sp.* and *Alistipes* were previously reported to be essential for maintaining balanced gut microbes protecting from HTN [14], CHF [37] and ACVD [20]. In addition, *Bilophila* is found in normal flora in human feces [46] and *Flavonifractor* was enriched in the feces of non-obese subjects [47].

Considering the difference in T2DM diagnosis and TC levels between the two groups, we questioned whether or not the alterations of GM observed in AF patients were mediated by T2DM/TC [15,48]. The partial least squares structural equation modelling (PLS-SEM) was applied to test if there was a mediation effect (indirect effect) of T2DM or TC during the shifts of GM observed in AF patients. It was found that the variance accounted for (VAF) scores, ratio of indirect-to-total effect which determines the proportion of the variance explained by the mediation process, for T2DM and TC were merely 3.39% and 2.85%, respectively (Additional files 9: Fig. S5a, b). Thus, the PLS-SEM indicated that although the T2DM and TC factors were different between groups, the contribution of T2DM and TC to the effect of the GM on AF was quite a small, with the majority of the effect not being mediated by these factors.

Additionally, the effects of statins and DMBG usage were further analyzed by PCA plots to assess the possible influence of drug consumption on GM in AF patients [49].

As indicated above, there were 4 AF patients taking statins and 6 taking DMBG. The PCA at the genus level failed to distinguished the AF patients into separate groups based on the usage of statins or DMBG, indicating a negligible impact of drug usage on our data ($P > 0.05$, Anosim, Additional files 10: Fig. S6). These findings based on the taxonomic profile of gut microbiota supported our hypothesis that there is serious dysbiosis of gut bacteria under AF state, which may play a crucial role in the pathology of atrial remodeling and the formation of an arrhythmogenic substrate.

AF state is identifiable by the gut co-abundance group

At the gene level, there were 121,145 genes differentially enriched in AF patients versus the controls (Additional files 11: Table S5). These genes were further clustered into co-abundance groups (CAGs) as we described previously [14] which generated 15,289 distinct CAGs (Additional files 12-15: Table S6-S9). The CAGs were then compared with the controls yielding 240 CAGs specifically enriched in AF (Additional files 13: Table S7). A cluster of CAGs containing *Prevotella*, along with anti-inflammatory CAGs such as *Faecalibacterium*, were more abundant in the healthy controls (Fig. 3). In contrast, the AF-enriched CAGs formed a cluster originated from proinflammatory *Ruminococcus*, *Dorea*, *Eubacterium*, and *Bacteroides*, some microbes enriched in CVD [14,20,37].

Based on the clusters of microbial CAG gene markers specific to AF, we aimed to further delineate the features of AF-associated GM and investigate the clinical values

of intestinal microbiome for distinguishing AF. Therefore, we performed a random forest disease classifier using the relative abundance of CAGs abundances as variables. With 5, 10, 20, 50, 70, 100 CAG marker variables, the classification error remained low and relatively stable (Additional files 16: Fig. S7, Fig. 4a). As shown in Fig. 4c, the area under receiver operating curve (AUC) was 97.74% (95% confidence interval (CI) of 95.27 %-100 %) in the training set (n=82) suggesting that subjects suffering from AF could be classified from the controls effectively. Consistently, the AUC for identifying AF from the controls was 98.57 (95% CI, 94.61%–100%) in the testing set (n=13). The CAGs that originated from *Blautia*, *Dorea*, *Eubacterium*, *Prevotella*, *Bacteroides*, *Ruminococcus* and *Lachnospiraceae* contributed the most to discriminating AF from controls (Fig. 4d). These CAGs were significantly correlated with each other. The abundances of bacteria enriched in controls were inversely correlated with AF group, and cluster together into a complicated network (Fig. 3). So far, we have constructed a microbiota-dependent discrimination model for AF detection, and thus the values of dysbiotic GM under AF condition should be further emphasized and uncovered.

Aberrant microbial functions in AF populations

The Kyoto Encyclopedia of Genes and Genomes (KEGG) and evolutionary genealogy of genes: Non-supervised Orthologous Groups (EggNOG) databases were utilized in the present study to access the gut microbial gene functions as described previously

[50,51] (Additional files 17-19: Table S10-S12). AF and control groups could be separated clearly from each other by both PCA and NMDS, suggesting significant differences of microbial functions in abundance between AF patients and controls ($P<0.001$, Anosim, Fig. 5a, b, d, e). There were thirty-five KEGG modules differentially enriched among the two groups (adjusted P value <0.05, Wilcoxon rank sum test, Fig. 5c), of which, twenty-four modules that were decreased in the AF group were implicated in the biosynthesis of fatty acid and aminoacyl-tRNA. Furthermore, genes for iron complex transport system, nucleotide sugar biosynthesis, citrate cycle and glycolysis were also reduced in AF patients. These metabolic functions produce metabolites necessary for maintaining human health and some have been indicated to be deficient in patients with HTN [14], CHF [37] or liver cirrhosis [17]. Eleven KEGG modules such as histidine biosynthesis, putative multiple sugar transport system, heme biosynthesis (glutamate to protoheme/siroheme) and pentose phosphate pathway were found to be significantly elevated in the AF group as well as in patients with colorectal adenoma-carcinoma [18], rheumatoid arthritis, T2DM, obesity, ACVD and cirrhosis [20]. Moreover, some EggNOG orthologs enriched in the control group participate in maintaining the normal human operations, such as DNA replication, recombination and repair and cell wall/membrane/envelope biogenesis. Other identified EggNOG orthologs, enhanced in AF patients, function in signal transduction mechanisms such as carbohydrate transport and metabolism. Considering these findings, the abnormal microbial functions that result from disordered GM composition in AF populations may

directly lead to imbalanced in metabolic profiles, resulting in disease development.

Alterations in gut and serum metabolomics in AF

Mammalian metabolism is thought to be greatly influenced by an interaction with the intestinal microflora community. To explore how the host metabolic pattern alterations were impacted by the gut microbiota dysbiosis in AF patients, serum and fecal samples were collected and analyzed by high-throughput liquid chromatography-mass spectrometry (LC/MS) in both positive ion mode (ES+) and negative ion mode (ES−). A subset of 26 subjects from the present study were enrolled in the serum metabolic study and 16 were enrolled in the feces study (Additional files 20-21, table S13-14). The partial least-squares discriminant analysis (PLS-DA) and the orthogonal partial least-squares discriminant analysis (OPLS-DA) were plotted to reveal the global metabolic changes between AF and controls. For the fecal samples, a clear separation between AF patients and healthy controls were obtained under both ES+ and ES- modes (Fig. 6 a, b). The serum data recapitulated the distinction, successfully classifying the AF and control groups with PLS-DA and OPLS-DA methods (Fig. 6 c, d).

Significant differentially enriched metabolites were identified based on the variable importance in the projection (VIP) threshold >1 and the p value < 0.05 and were further matched in the Metlin database. Overall, 113 serum metabolites, 100 elevated and 13 decreased, were detected in AF patients as compared to controls (Additional files 22: Fig. S8). For the stool samples, 76 metabolites, 18 increased and

58 down-regulated, differentiated AF patients from healthy controls (Additional files 23: Fig. S9). Notably, 29 metabolites were altered in both serum and stool samples of AF patients (Fig. 7a, b), 11 of which showed the same variation trend and were the focus of further investigation (Fig. 7b, Additional files 24: Table S15). These compositional changes identified AF-enriched compounds, such as alpha-hydroxyisovaleric acid and L-lysin. There were 7 metabolites with significantly decreased abundance in AF including SA, OLA, LA, cholic acid, palmitic acid, octadecanedioic acid, and L-homotryosione (Fig. 7b). It has been shown that lower level of circulating SA was responsible for a higher risk of AF [27]. Cholic acid may influence the cardiac electrophysiology, inhibiting the activity of cardiac myocytes, causing calcium overload and leading to sudden fetal death hence might influence the cardiac electrophysiology [47]. These metabolic variations might aggravate or even promote the arrhythmogenic substrate aggravation in the left atrium during the pathological processes of AF.

To explore the association between aberrant metabolites and disordered gut microflora, we carried out a correlation analysis between the top 10 genera enriched in AF or control groups and the 11 representative metabolites in serum or feces with similar variation tendencies (Fig. 7c). SA, which may be a crucial protective factor from AF, was negatively correlated with microflora, including *Streptococcus*, *Eubacterium*, *Enterococcus*, *Dorea*, *Coprobacillus*, and *Blautia*, which were enriched in AF patients. Moreover, OLA and LA, known as cardiovascular protectors, were negatively

associated with *Streptococcus Eubacterium, Enterococcus, Dorea, Coprobacillus,* and *Blautia*. Previous observational studies have reported that LA, the predominant n-6 PUFA from vegetable oils and nuts, could reduce major risk factors of ACVD [53]. Increased LA intake is believed to reduce LDL cholesterol, promote insulin sensitivity and attenuate the risk of HTN. A 20-year cohort study following more than 74,000 participants revealed that OLA consumption significantly relieved the risk for developing cardiovascular disease (CVD) [54]. OLA prevents coronary heart disease by suppressing oxidative stress, mitigating cardiomyocyte cell damage [55].

The distinguished metabolic features in AF were significantly correlated with the disordered gut floras and may be possible markers for AF. It was indicated that the gut microbiota dysbiosis induced disordered microbial functions, causing the deficiency of multiple cardiovascular-protective metabolites and thus increased susceptibility to AF.

Discussion

In the present study we obtained seminal evidence delineating the features of the AF-associated gut dysbiosis through the integration of metagenomic and metabolomic analyses. The AF individuals exhibited significantly elevated richness and increased diversity of gut microbiota and thus the overgrowth of bacteria may be key to the development and establishment of AF. The GM shift from an enterotype represented by *Prevotella* to *Bacteroides* further characterized an imbalanced intestinal microbial environment specific to AF. Gut bacteria such as *Faecalibacterium*, *Alistipes*,

Oscillibacter, *Bilophila* and *Flavonifractor* declined substantially in the intestinal tract from AF patients. Inversely, *Ruminococcus*, *Streptococcus* and *Enterococcus* were typically enriched in the AF-associated gut metagenomic composition. Metabolic profiles of both fecal and serum samples analyzed from AF patients demonstrated significant alterations, which were correlated with gut microbiota dysbiosis. More importantly, a discriminant model based on bacterial signature profiles has been established and may have the potential to be used as biomarkers for AF in the future. It is therefore hypothesized that an increase of a specific group of gut flora may induce disordered metabolic activity of GM, triggering the accumulation of bacterial metabolites in the circulation. This accumulation, where they could negatively affect human health perturbing the progression of AF and may even play an important role in the establishment of AF. Intervention strategies targeting gut microbiota to improve the progression of AF are strongly encouraged.

To our knowledge, the richness and diversity of GM has been evaluated in multiple diseases, particularly in CVD, and variable findings were reported recently. In atherosclerotic disease, it was suggested that GM diversity is inversely associated with arterial stiffness in women [56] whereas a higher microbial richness and diversity in the systemic microbiome of ST-segment correlated to elevated myocardial infarction events [57]. The increased diversity of GM was also observed in stroke and transient ischemic attack patients and this dysbiosis was correlated with the severity of the disease [58]. Thus, the evaluated richness and diversity of GM could reflect the

imbalanced gut milieu, characterized by overgrowth of a variety of harmful bacteria and fewer commensal or beneficial genera. This is consistent with the present study.

A cluster of bacteria significantly aggregated in the gut from AF patients, including *Ruminococcus*, *Streptococcus* and *Enterococcus*. The accrual of these microorganisms in the intestine may inhibit the growth of some bacteria enriched in healthy populations. For example, the decline of *Faecalibacterium*, *Alistipes*, *Oscillibacter*, *Bilophila* and *Flavonifractor* often occurred in conjunction with changes *Streptococcus* abundance [14,20,37]. It is worth noting that AF patients shared the enrichment of numerous microbial floras, such as *Streptococcus*, *Dorea*, *Enterococcus* and *Coprobacillus*, demonstrated in HTN [14], CHF [37] and ACVD [20]. Additionally, patients with cardiovascular diseases often have decreased levels of *Faecalibacterium* and *Oscillibacter*, which are butyrate-producing species identified as important anti-inflammatory commensal bacterium [42,59]. *Alistipes*, *Bilophila* and *Butyricicoccus* also exhibited the same decreasing trend in AF and other CVDs, like HTN [14], CHF [37] and ACVD [20]. This group of bacterial strains is consistently altered in multiple cardiovascular diseases and is therefore considered a guild emerging during the progression of disease. The chronic cardiovascular diseases mentioned above might be a consequence of the imbalanced gut microbial composition associated with the establishment of this guild. Although the underlying mechanism remains largely unknown, several CVDs share some common pathophysiologic pathways, such as endothelial dysfunction [60,61]. Reestablishing the functionally active ecological

populations as the primary ecosystem service providers is crucial to a healthier gut microbiota. Restoring the deficient gut microbe might alleviate or attenuate the disease phenotypes or progression. Targeted promotion of the gut ecosystem by individualized intertion may present a novel ecological approach for manipulating the gut microbiota to manage CVD and potentially other dysbiosis-related diseases [62].

Notably, GM of AF exhibited some unique features not displayed in other related diseases. For example, *prevotella*, whose function is to encode superoxide reductase, phosphoadenosine phosphosulphate reductase and favor the development of inflammation [63,64], showed a declined trend in AF, but overgrowth in HTN [14]. In addition, some flora decreased in HTN [14] exhibited a tendency to be increased in AF, CHF [37] and ACVD [20], such as *Ruminococcus*, *Enterococcus*, *Veillonella*, *Coprococcus* etc. These seemingly paradoxical phenomena may partly be explained by the complex and various factors involved in the pathophysiological process. To a certain extent, the generality and specificity of cardiovascular diseases could be analyzed from the point of view of gut flora.

Metabolites derived by the gut microbiota, such as TMAO, have been confirmed to act on downstream cellular targets to improve or contribute to the pathogenesis of structural, metabolic and functional cardiovascular remodeling [65]. Here, our present study revealed decreased levels of oleic acid (OLA), palmitic acid (PA), stearic acid (SA) and linoleic acid (LA) in AF patients, which was consistent with the decreased

function of GM in fatty acid biosynthesis. High serum concentrations of monounsaturated fatty acids (MUFAs), including OLA, have been noted in obese individuals, patients with metabolic syndrome and atherosclerotic patients with T2DM [66]. A recent study found that OLA could regulate atrial electrophysiological characteristics with calcium and sodium dysregulation, which may contribute to atrial arrhythmogenesis [67]. However, an atrial cell line from mice was used in this study, which may not be reflective of mature human atrial cells. Moreover, it was not clear whether OLA would have different effects on atrial electrophysiology under other concentrations or incubation periods. The actual effect of OLA as a human atrial substrate is extremely complex and therefore necessitates further investigation.

In addition, the risk of saturated fatty acids (SFAs) on cardiovascular disease is fairly controversial. A previous study aimed to determine the mechanisms underlying electrophysiological effects of palmitic (PA), stearic (SA) and oleic (OLA) free fatty acids (FFAs), found that SA could disrupt t-tubular architecture and remodel properties of membrane ionic currents in sheep atrial myocytes, with potential implications in arrhythmogenesis [68]. A higher risk of AF was demonstrated to be associated with higher levels of circulating PA but less SA by a prospective cohort study [27]. Taken together, these findings highlight potential and diverse physiological effects of GM-related metabolites during the progression of AF. Further studies are required to understand the biological mechanism underlying these differential effects.

Promisingly, the microbiota-dependent discrimination model we built could distinguish AF from controls nicely based on the GM feature. Traditionally, AF can be further distinguished as paroxysmal (PAF) and persistent (PeAF) AF based on the presentation, duration and spontaneous termination of AF episodes. The episodic pattern of PAF is self-terminating, in most cases within 48 hours, while peAF is characterized as lasting longer than 7 days, including episodes that are terminated by cardioversion, with drugs or by direct current cardioversion after 7 days or more [69]. Among our present AF cohort, there were 30 PAF patients and 20 peAF patients. The types of AF may be partially determined by the varying extent of personalized electrical and structural remodeling in atrial arrhythmogenic substrate. Additionally, they have different prognoses and responses to rhythm-controlling therapy and distinction between the types helps the physician and patient to make individualized therapeutic decisions [70]. Therefore, the classification of AF type based on the characteristics of gut microbiota might have more significant clinical value, which will be explored in our future work.

Consideration of possible confounders and limitations are of relevance to our study and help to inform the design of future studies. Some of the AF patients recruited in our cohort were also diagnosed with HTN or T2DM. Isolated AF, driven by genetic factors, represents a minority of AF cases and the pathogenesis of AF may be an end stage of multiple metabolic and cardiovascular diseases [11,12]. To reflect the real signature of clinical practice we did not exclude patients with comorbidities even though HTN and

T2DM have been widely known to be connected with GM dysfunction. To evaluate the disordered patterns of GM resulting solely from AF, the HTN history in each group was matched individually to remove the HTN contribution. Separately, there were 12 AF patients with T2DM which was not adjusted between groups. The PLS-SEM was used to assess the possible contribution of T2DM and we found that the indirect effect of the GM mediated by T2DM was statistically significant but of quite a small magnitude. Therefore, the majority effect of GM dysbiosis observed in the AF-associated cohort was not mediated by HTN or T2DM. Secondly, although we excluded subjects who used antibiotics or probiotics and confirmed the possible influences of drug consumption (DMBG and statins) on gut microbiota, exercise and dietary information were not collected and corrected in this study. Thirdly, the conclusions drawn from our data were associations rather than causal relationships. Further studies such as gut microbiota transplantation and electrophysiological modulation testing AF inducibility are still needed. The present results provided preliminary clues and evidence for future investigations regarding the potential mechanisms between gut microbes and AF.

Conclusions

The present study provides the first comprehensive description of the disordered patterns of gut microbiota and aberrant microbial-related metabolites in a cohort of AF patients. These novel findings are fundamental for further studies exploring the causal

relationship between AF and GM, but they are just the beginning. An extensive amount of research is still needed to explore the clinical values of intervention strategies based on gut microbiota to improve AF conditions.

Methods

Study cohort

50 patients with nonvalvular AF were consecutively enrolled from Beijing Chaoyang Hospital and 50 individuals as matched controls were enrolled from Kailuan cohort who received biennial medical examination in Kailuan General Hospital [28]. Individuals with a history of heart failure, coronary heart disease, structural heart disease, comorbidities (inflammatory bowel diseases, irritable bowel syndrome, autoimmune diseases, liver diseases, renal diseases or cancer) or use of antibiotics or probiotics in the last 1 month were excluded. Demographic and clinical characteristics were obtained by completing face-to-face surveys and checking hospital or medical examination records. 50 samples from our previous work [14] regarding gut microbiota were selected by matching for the history of hypertension and the metagenomic sequencing data of 50 control stool samples from our previous study were used as controls in the present study. Among the 50 AF patients included, fecal samples were available from each subject and used for metagenomic analyses. Metabolomic analyses were performed using serum samples from 8 AF patients and 12 controls and stool samples from 8 AF patients and 8 controls. The study conforms well to the principles

from the Declaration of Helsinki. The research protocol was approved by the ethics committee of Beijing Chaoyang Hospital and Kailuan General Hospital. All of the participants signed informed consents.

Stool sample collection and DNA extraction

Fresh stool samples were collected from each participant, immediately frozen at −20 °C, transported on ice to the laboratory and then stored at −80 °C. Bacterial DNA was extracted using TIANGEN kit from Novogene Bioinformatics Technology Co., Ltd.

Metagenomic sequencing, gene catalogue construction

Paired-end metagenomic sequencing was sequenced on the Illumina platform (insert size 300 bp, read length 150 bp) at the Novogene Bioinformatics Technology Co., Ltd. After quality control, the reads aligned to the human genome (alignment with SOAP2, Version 2.21, parameters: -s 135, -l 30, -v 7, -m 200, -x 400) were removed and the remaining high-quality reads were used for further analysis. The assembly of reads was executed using SOAP de novo (Version 2.04, parameters: -d 1 -M 3 -R -u -F). For each sample, we used a series of k-mer values (from 49 to 87) and chose the optimal one with the longest N50 value for the remaining scaffolds [17]. The clean data was mapped against scaffolds using SOAP2 (Version 2.21, parameters: -m 200 -x 400 -s 119). Unused reads from each sample were assembled using the same parameters.

Gene prediction from the assembled contigs was performed using MetaGeneMark (prokaryotic GeneMark.hmm version 2.10). A non-redundant gene catalogue was constructed with CD-HIT (version 4.5.8, parameters: -G 0 -aS 0.9 -g 1 -d 0 -c 0.95) using a sequence identity cut-off of 0.95, with a minimum coverage cut-off of 0.9 for the shorter sequences. Reads were realigned to the gene catalogue with SOAP2 using parameters to determine the abundance of genes: -m 200 -x 400 -s 119. Only genes with ≥2 mapped reads were decided included. The gene abundance was calculated by counting the number of reads and normalizing by gene length.

Analyses of genera richness and enterotypes

Rarefaction analysis was carried out to evaluate gene richness. Using R (Version 2.15.3, vegan package), the cohort was randomly sampled 100 times with replacement and the total number of identified genes from these samples was assessed.

Based on the genera profiles, we calculated the within-sample (α) diversity using the Shannon index to estimate the genera richness of the sample. A high α diversity denotes a high richness of genera within the sample.

By using the PAM method based on relative abundance of genera, we analyzed the community types of each sample. As previously described [29], we estimated the optimal number of clusters using the CH index. Genera with an average relative abundance $\geq 10^{-4}$ and present in at least six samples would be used in the analysis. The genera in enterotype 1 were clustered according to the Spearman's correlation between

genera abundances and their co-occurrence network was visualized using Cytoscape (Version 3.2.1).

Taxonomic assignment, annotation and abundance profiling

Genes were aligned to the integrated NR database to assess the taxonomic assignment by using DIAMOND (Version 0.7.9.58, default parameter except that -k 50 -sensitive -e 0.00001). To distinguish taxonomic groups, the significant matches for each gene, defined by e-values ≤10 × e-value of the top hit, were determined and the retained matches were used as previously described [30]. The taxonomical level of each gene was determined using the lowest common ancestor-based algorithm implemented with MEGAN. The abundance of a taxonomic group was calculated by summing the abundance of genes annotated to a feature.

Co-abundance gene groups (CAGs) and CAGs network of marker CAGs

As previously described [31,32], we compared the abundance of each gene across groups to identify the marker genes associated with AF. Based on their abundance variation across groups these marker genes were clustered into groups [34]. Co-abundance gene groups (CAGs) were defined as clusters with more than 50 genes. CAG abundance profiles were calculated based on the average gene depth signal and weighted by gene length. Taxonomic assignment of the CAGs was performed based on the taxonomy of tracer genes. The species assignment required 90% of the genes in a CAG to match with the species' genome with 95% identity and 70% overlap of query.

The CAG assignment to a genus required 80% of its genes to align to the genome with 85% identity in both DNA and protein sequences.

The enriched CAGs were identified and clustered according to Spearman's correlation and the co-occurrence network was visualized by Cytoscape (Version 3.2.1). Based on the abundance in the set of compared samples, an OR score was calculated for each CAG and for the comparative analysis between control and AF samples; the AF-associated CAGs were identified as AF-enriched (OR >2) or AF-depleted (OR <0.5).

Functional annotation

Using DIAMOND (Version 0.7.9.58, default parameter except that -k 50 –sensitive -e 0.00001), all genes in catalogue were aligned to the KEGG database (Release 73.1, with animal and plant genes removed) and to the eggNOG database (v4.5 via eggNOG-mapper with HMM search mode). Each protein was assigned to the KEGG and eggNOG orthologs using the highest scoring annotated hits containing at least one HSP scoring over 60 bits. By summing the abundance of genes annotated to the same feature, the abundance of KEGG orthologue/module was calculated.

Metabolomic analysis based on LC/MS

50 mg fecal samples were pipetted into centrifuge tubes (1.5 mL) in preparation for extraction. The protein was precipitated with 800 μL of methanol and 10 μL of internal

standard (2.9 mg/mL, DL-o-Chlorophenylalanine) was added. The samples were ground at 65 KHz for 90 s and centrifuged at 12000 rpm for 15 min at 4 °C. 200 μL of the supernatant was transferred into a vial for further analysis. The fecal metabolic profiles were performed on a LC/MS platform (Thermo, Ultimate 3000LC, Orbitrap Elite) using a Hypergod C18 (100 × 4.6 mm 3 μm) column. The chromatographic separation conditions were as follows: the column temperature, 40 °C; flow rate, 0.3 mL/min; mobile phase A, water +0.1% formic acid; mobile phase B, acetonitrile +0.1% formic acid; injection volume, 4 ml; automatic injector temperature, 4 °C.

The serum samples were thawed at room temperature and 100 μL was pipetted into centrifuge tubes (1.5 mL) in preparation for extraction. The protein was precipitated with 300 μL of methanol, and 10 μL of internal standard (2.9 mg/mL, DL-o-Chlorophenylalanine) was added. The samples were vortexed for 30 s and centrifuged at 12000 rpm for 15 min at 4 °C. 200 μL of the supernatant was transferred to a vial for further analysis. The serum metabolic profiles were performed on a LC/MS platform (Thermo, Ultimate 3000LC, Orbitrap Elite) by using a Hypergod C18 (100 × 4.6 mm 3 μm) column. The chromatographic separation conditions were as follows: column temperature, 40 °C; flow rate, 0.3 mL/min; mobile phase A, water +0.1% formic acid; mobile phase B, acetonitrile +0.1% formic acid; injection volume, 4 μL; automatic injector temperature, 4 °C.

For both fecal and serum samples the following conditions were used for the positive ion mode (ES+): heater temp, 300 °C; sheath gas flow rate, 45arb; aux gas flow rate, 15arb; sweep gas flow rate, 1arb; spray voltage, 3.0KV; capillary temp, 350 °C; S-lens RF level, 30%. The following conditions were used for negative ion mode (ES−): Heater temp, 300 °C; sheath gas flow rate, 45arb; aux gas flow rate, 15arb; sweep gas flow rate, 1arb; spray voltage, 3.2KV; capillary temp, 350 °C; S-lens RF level, 60%.

All metabolomic data were prepared for feature extraction and preprocessed with Compound Discoverer 2.0 software (Thermo). Data were then normalized and edited into a two-dimensional data matrix by excel 2010 software, using Retention time (RT), Compound Molecular Weight (compMW), Observations (samples) and peak areas. Using SIMCA-P software (Umetrics AB, Umea, Sweden), a multivariate Analysis (MVA) was performed. Compounds were significantly distinguished between groups, identified by a variable influence on projection (VIP) > 1 and p value < 0.05 based on the peak areas. The m/z value of these compounds was used to identify the metabolites related to the featured peak in the Metlin database. As for metabolites detected in both ES+ and ES−, the data in the mode with the higher VIP was retained for further analysis.

Statistical analysis

Quantitative demographic and clinical characteristic data with normal distributions were presented as mean ± standard deviation and the t-test was used for between group comparisons. Quantitative data with non-normal distributions were presented as

median (first quartile, third quartile) and the Wilcoxon rank sum test was performed for between group comparisons. Qualitative data were presented as a percentage and the Chi-square test was used for between group comparisons. All statistical tests were 2-sided and $p < 0.05$ was regarded as significant. Statistical analyses were performed with the SPSS version 22.0 (IBM Corp., Armonk, New York)

The Shannon index at the genera level was calculated with QIIME (Version 1.7.0). PCA was performed using the FactoMineR package in R software (Version 2.15.3) while PCoA was performed by using ade4 package, cluster packages, fpc packages, and clusterSim package in R software (Version 2.15.3). PLS-SEM analysis was conducted using the Smart-PLS 3 software. PLS-DA was carried out using the SIMCA-P software to cluster sample plots across groups.

Differential abundance of genes, genera and KO modules was tested based on the Wilcoxon rank sum test and P values were corrected for multiple testing with the Benjamin & Hochberg method. Genera with an average relative abundance $\geq 10^{-4}$ and presence in at least six subjects were included in the analyses.

Based on the profiles of CAGs, the samples were randomly divided into training and test sets. A random forest classifier was trained on 80% of the data and tested on the remaining 20% of our data using the random forest package in R. We performed a 10-fold cross-validation within the training set to evaluate the performance of the predictive model and obtain more precise curves. The cross-validation error curves

(average of 10 test sets each) from five trials of the 10-fold cross-validation were averaged. Variable importance was calculated for the random forest models using the full set of features determined by mean decrease in accuracy. At the lowest cross-validation error, the number of variables was 1000. Therefore, the predictive model was constructed using the 1000 most important variables, and the performance was assessed using ROC analysis. The 95% CIs for the ROC curves were calculated using the pROC R package. The performance of the smaller models was measured as the AUC when applied to the test set.

Data Availability

The data set supporting the results of this article has been deposited in the EMBL European Nucleotide Archive (ENA) under BioProject accession code PRJEB28384 [http://www.ebi.ac.uk/ena/data/view/PRJEB28384].

Acknowledgements

Not applicable.

Funding

This work was supported by the National Natural Science Foundation of China (81500383, 81670214, 81870308, 81770253, 81370362), the Beijing Natural Science Foundation (7172080), the Beijing Municipal Administration of Hospitals' Youth Programme (QML20170303), and the 1315 personnel training plan (CYMY-2017-03).

Author contributions

XCY, JL and KZ conceived the study, directed the project, designed the experiments, interpreted the results, and wrote the manuscript. YFG, MLC, RMH, YL, HJC, and HJW recruited, diagnosed, and collected the clinical details from the subjects. SCL and XYL collected the blood and feces samples from the subjects. KZ, JL, JC and KBL analyzed the data. XCY, JL, YQW, HCW, JC and JCZ revised the manuscript. All authors read and approved the final manuscript.

Ethics approval and consent to participate

The research protocol was approved by the ethics committee of Beijing Chaoyang Hospital and Kailuan General Hospital. All of the participants signed informed consents.

Consent for publication: Not applicable.

Conflict of interests: The authors declared no conflicts of interest to this work.

Table1. Baseline clinical characteristics of the study cohort.

	AF Group	Control Group	P value
**Number**	50	50	/
**Age, years**	66 (57, 71.25)	55 (50.5, 57.5)	<0.001
**Male/ Female**	32/18	41/9	0.043
**BMI**	26.46 (23.79, 28.64)	24.77 (22.79, 27.62)	0.112
**HTN**	27	27	/
**DM**	12	0	/
**TC**	4.13±1.05	4.82±0.96	0.001
**TG**	1.29 (1.02, 1.88)	1.06 (0.77, 1.80)	0.084
**LDL**	2.45 (1.58, 2.93)	2.3 (1.96, 2.86)	0.872
**FBG**	4.95 (4.50, 5.83)	5.12 (4.56, 5.55)	0.883
**Creatinine**	68.5 (60.48, 79.35)	70 (60, 89.5)	0.533
**UA**	321.5 (278, 389.75)	333 (264.5, 384)	0.927
**TBil**	14 (10.08, 19.5)	14.7 (11.59, 19.75)	0.431
**ALT**	19 (13.75, 28.5)	19 (12, 25)	0.185

Abbreviations: AF, atrial fibrillation; BMI, body mass index; HTN, hypertension; DM, diabetes mellitus; CHD, coronary heart disease; TC, total cholesterol; TG, triglyceride; LDL, low density lipoprotein; FBG, fasting blood glucose; UA, uric acid; TBil, total bilirubin; ALT, glutamic-pyruvic transaminase. IQR, interquartile range; Data are presented as mean± SD, or median (IQR), as appropriate.

Additional files

Additional files 1: Table S1, Data production of 100 samples in control and AF.

Additional files 2: Figure S1 Increased Firmicutes/Bacteroidetes ratio, Pielou evenness and Chao richness in AF.

Additional files 3: Figure S2 Another 12 genera significantly enriched in enterotype 1.

Additional files 4: Figure S3 Taxonomic annotation and abundance profiling.

Additional files 5: Table S2 Relative abundance profile at the phylum level.

Additional files 6: Table S3 Relative abundance profile at the genus level.

Additional files 7: Table S4: Detailed information of differential genera.

Additional files 8: Fig. S4 Species strikingly different across groups.

Additional files 9: Fig. S5 Mediation effect of T2DM/TC during the process from disordered gut microbiota to AF.

Additional files 10: Fig. S6 Insignificant influents of statins and metformin usage on GM.

Additional files 11: Table S5 Detailed information for 121145 gene markers.

Additional files 12: Table S6 Reference genomes for CAG's taxonomy assignment.

Additional files 13: Table S7 Detailed information of enriched CAGs in different groups.

Additional files 14: Table S8 Detailed information of 477 CAGs.

Additional files 15: Table S9 Spearman’s correlation between enriched CAGs.

Additional files 16: Fig. S7 Gut CAGs (variables in 5, 10, 20, 50, 70) classify AF from controls.

Additional files 17: Table S10 Detailed information of differential KEGG modules.

Additional files 18: Table S11 Detailed information of differential KEGG orthologues.

Additional files 19: Table S12 Detailed information of differential eggNOG family.

Additional files 20: Table S13 Clinical characteristics of participants in serum metabolism.

Additional files 21: Table S14 Clinical characteristics of participants in fecal metabolism.

Additional files 22: Fig. S8 Metabolites differentially enriched in AF and controls in serum.

Additional files 23: Fig. S9 Metabolites differentially enriched in AF and controls in feces.

Additional files 24: Table S15 Detailed information of 11 metabolites differently enriched across groups.

Figure Legends

Figure 1. Elevated microbiota richness and altered community types in AF patients.

a. Rarefaction curves for gene number which were calculated after 100 random sampling with replacement in control (n = 50) and AF (n = 50). The rarefaction curve is near smooth when the sequencing data are great enough with few new genes undetected.

b, c. Gene count and a-diversity (Shannon index) based on the genera profile in the AF and CTR cohorts. P=0.007, CTR vs AF; for gene count. P=3.53e-05, CTR vs AF; for α diversity; Kruskal-Wallis test.

d. 100 samples are clustered into enterotype 1 (green) and enterotype 2 (orange) by principal component analysis (PCA) of Jensen-Shannon divergence values at the genus level. The major contributor in the two enterotypes is Bacteroides and Prevotella, respectively.

e. Relative abundances of the top genera in each enterotype, Bacteroides in enterotype 1, Prevotella in enterotype 2. P = 1.730774e-09 and P = 4.376078e-14, respectively; Wilcoxon rank sum test.

f. The percentage of control and AF samples distributed in enterotype1 and enterotype2. 50% CTRs in enterotype1, 50% CTRs in enterotype2. 82% AFs in enterotype1, 18% AFs in enterotype2. P = 0.001, CTR vs AF; Fisher's exact test.

Figure S1. Increased Firmicutes/Bacteroidetes ratio, Pielou evenness and Chao richness in AF.

a. Firmicutes/Bacteroidetes ratio based on the phylum profile in the AF and CTR cohorts. P=7.162e-05, CTR vs AF; Kruskal-Wallis test.

b, c. Chao richness and Pielou evenness based on the genera profile in the AF and CTR cohorts. P=0.007633, CTR vs AF; for Chao richness. P=4.262e-06, CTR vs AF; for Pielou evenness; Kruskal-Wallis test.

Figure S2. Another 12 genera significantly enriched in enterotype 1.

Boxes represent the inter quartile ranges, lines inside the boxes denote medians and circles are outliers. +, adjust P value <0.01; ns, not significant; Wilcoxon rank sum test.

Figure S3. Taxonomic annotation and abundance profiling.

a, b. PCA and plot for non-metric dimensional scaling (NMDS) based on abundances of the microbes showed the structures of gut microbiota in AF were significantly different from controls.

c. Heatmap of genera enriched across controls and AF patients. The abundance profiles were transformed into Z scores by subtracting the average abundance and dividing the standard deviation of all samples. Z score is negative (shown in blue) when the row abundance is lower than the mean.

Figure 2. Genera strikingly different across groups.

a. PCA based on abundances of the microbes showed the structures of gut microbiota in AF were significantly different from controls in genus level.

b. Relative abundance of the top 35 most different genera across groups at the criteria of P value <0.05; Wilcoxon rank sum test. The abundance profiles are transformed into Z scores by subtracting the average abundance and dividing the standard deviation of all samples. Z score is negative (shown in blue) when the row abundance is lower than the mean.

c, d. The box plot shows the relative abundance of top 10 genera enriched in AFs and controls. Genera are colored according to the phylum, boxes represent the inter quartile ranges, lines inside the boxes denote medians and circles are outliers.

Figure S4. Species strikingly different across groups.

a. PCA based on abundances of the microbes showed the structures of gut microbiota in AF were significantly different from controls in species level.

b. Relative abundance of the top 35 most different species across groups at the criteria of P value <0.05; Wilcoxon rank sum test. The abundance profiles are transformed into Z scores by subtracting the average abundance and dividing the standard deviation of all samples. Z score is negative (shown in blue) when the row abundance is lower than the mean.

c, d. The box plot shows the relative abundance of top 10 species enriched in AF patients and controls. Species are colored according to the phylum, boxes represent the inter quartile ranges, lines inside the boxes denote medians and circles are outliers.

Figure S5. Mediation effect of T2DM/TC during the process from disordered gut microbiota to AF.

Mediation analysis of the association between top 35 most different genera and AF using partial least squares structural equation modelling. Path coefficients are denoted beside each path and indirect effect and variance accounted for (variance accounted for) score is denoted below each mediator (*P < 0.05; **P < 0.01; ***P < 0.001).

Figure S6. Insignificant influents of statins and metformin usage on GM.

PCA based on relative abundance of different genera in controls and AF patients with and without DMBG or statin usage showed no significantly different of the gut microbial structures.

Figure 3. The network of CAGs enriched in AF compared with controls.

CAGs are colored according to the taxonomic assignment as labeled, and the node size is scaled with the number of genes within the CAG. Edges between nodes denote Spearman correlation positive (red) or negative (blue).

Figure 4. Gut CAGs classify AF from controls.

a. Distribution of 5 trials of 10-fold cross-validation error in random forest classification of AF as the number of CAGs increases. The model was trained using relative abundance of the CAGs in the controls and AF samples. The black curve

indicates average of the five trials (grey lines), and the red line marks the number of CAGs in the optimal set.

b. Box-and-whisker plot for the probability of AF in the cross-validation training set according to the model in a.

c. Receiver operating curve (ROC) for the training set. The area under receiver operating curve (AUC) is 97.74% and 95% confidence interval (CI) is 95.27–100%.

d. The top 30 different CAGs distinguish AF from control based on the random forest model using explanatory variables of CAGs.

e. ROC for the test set. The AUC is 98.57% and 95% CI is 94.61–100%.

Figure S7. Gut CAGs (variables in 5, 10, 20, 50, 70) classify AF from controls.

(a-d, variable=5); e-h, variable=10; i-l, variable=50; m-p, variable=70.)

a, e, I, m. Distribution of 5 trials of 10-fold cross-validation error in random forest classification of AF as the number of CAGs increases.

b, f, j, n. Box-and-whisker plot for the probability of AF in the cross-validation training set.

c, g, k, o. ROC for the training set.

d, h, l, p. ROC for the test set.

Figure 5. Microbial gene functions annotation in AF.

a, b. PCA and NMDS based on the relative abundance of KEGG orthology groups in 100 samples showed significant difference between AF and CTR.

c. The average abundance of KEGG modules differentially enriched in control and AF gut microbiome. 24 modules enriched in control, and 11 modules overrepresented in AF are shown in green and pink, respectively. The physiological effect of KEGG modules are demonstrated on the right.

d, e. PCA and NMDS based on the relative abundance of eggNOG orthologues in 100 samples showed significant difference between AF and CTR either.

f. The average abundance of eggnog orthologues differentially enriched in control and AF. 15 eggNOGs enriched in control, and 20 eggNOGs overrepresented in AF are shown in green and pink, respectively. The potential function of eggNOGs are demonstrated on the right.

Figure 6. Distinguished metabolic patterns between AF and control.

a. Partial least squares-discriminant analysis (PLS-DA) score plots based on the metabolic profiles in feces samples from control and AF group in ES+ and ES−.

b. Score scatter plots of orthogonal PLS-DA (OPLS-SA) comparing the feces metabolic differences identify the separation between AF and control in ES+ and ES−.

c. PLS-DA score plots based on the metabolic profiles in serum samples from control and AF group in ES+ and ES−.

d. Score scatter plots of OPLS-DA comparing the serum metabolic differences identify the separation between AF and control in ES+ and ES−.

Figure S8. Metabolites differentially enriched in AF and controls in serum.

The abundance of 113 endogenous compounds varied in AF serum is transformed into Z scores in the heat map. Metabolites significantly changed in AF as compared to control at VIP >1.0 and P value (t test) <0.05 are identified

Figure S9. Metabolites differentially enriched in AF and controls in feces.

The abundance of 76 endogenous compounds varied in AF feces is transformed into Z scores in the heat map. Metabolites significantly changed in AF as compared to control at VIP >1.0 and P value (t test) <0.05 are identified.

Figure 7. Aberrant metabolic patterns related to AF

a. Venn diagrams demonstrated the number of altered metabolites shared between serum (purple) and feces (yellow). The overlap showed that there were 29 endogenous compounds concurrently identified in both feces and serum.

b. The heat map of fold/change (AF/CTR) of 29 compounds. The fold/change were transformed into t-scores, and t-score is negative (shown in blue) when the compound

showed a decline tendency in AF group. Compounds which increased or decreased simultaneously (n=11) or unsynchronized (n=18) in feces and serum were shown in green and pink, respectively.

c. The relationship between 11 endogenous metabolites and the 20 top altered genera in AF; Spearman's correlation analysis. Red, negative correlation; blue, positive correlation, *$p < 0.05$, +$p < 0.01$. The enriched type of each genera and metabolic patterns was colored according to its direction of enrichment. Blue, enriched in controls; red, enriched in AF patients.

References

1 Lloyd-Jones DM, Wang TJ, Leip EP, Larson MG, Levy D, Vasan RS, et al. Lifetime risk for development of atrial fibrillation: the Framingham Heart Study. Circulation. 2004; 110: 1042-1046.

2 Heeringa J, van der Kuip DA, Hofman A, Kors JA, van Herpen G, Stricker BH, et al. Prevalence, incidence and lifetime risk of atrial fibrillation: the Rotterdam study. Eur Heart J. 2006; 27: 949-953.

3 Haim M, Hoshen M, Reges O, Rabi Y, Balicer R, Leibowitz M. Prospective national study of the prevalence, incidence, management and outcome of a large contemporary cohort of patients with incident non-valvular atrial fibrillation. J Am Heart Assoc. 2015; 4: e001486.

4 Chugh SS, Havmoeller R, Narayanan K, Singh D, Rienstra M, Benjamin EJ, et al. Worldwide epidemiology of atrial fibrillation: a Global Burden of Disease 2010 Study. Circulation. 2014; 129: 837-847.

5 Oldgren J, Healey JS, Ezekowitz M, Commerford P, Avezum A, Pais P, et al. Variations in cause and management of atrial fibrillation in a prospective registry of 15,400 emergency department patients in 46 countries: the RE-LY Atrial Fibrillation Registry. Circulation. 2014; 129: 1568-1576.

6 Kotecha D, Holmes J, Krum H, Altman DG, Manzano L, Cleland JG, et al. Efficacy of beta blockers in patients with heart failure plus atrial fibrillation: an individual-patient data meta-analysis. Lancet. 2014; 384: 2235-2243.

7 Stewart S, Hart CL, Hole DJ, McMurray JJ. A population-based study of the long-term risks associated with atrial fibrillation: 20-year follow-up of the Renfrew/Paisley study. Am J Med. 2002; 113: 359-364.

8 Andersson T, Magnuson A, Bryngelsson IL, Frobert O, Henriksson KM, Edvardsson N, et al. All-cause mortality in 272,186 patients hospitalized with incident atrial fibrillation 1995-2008: a Swedish nationwide long-term case-control study. Eur Heart J. 2013; 34: 1061-1067.

9 Krahn AD, Manfreda J, Tate RB, Mathewson FA, Cuddy TE. The natural history of atrial fibrillation: incidence, risk factors, and prognosis in the Manitoba Follow-Up Study. Am J Med. 1995; 98: 476-484.

10 Marrouche NF, Wilber D, Hindricks G, Jais P, Akoum N, Marchlinski F, et al. Association of atrial tissue fibrosis identified by delayed enhancement MRI and atrial fibrillation catheter ablation: the DECAAF study. JAMA. 2014; 311: 498-506.

11 Fox CS, Parise H, D'Agostino RBSr, Lloyd-Jones DM, Vasan RS, Wang TJ, et al. Parental atrial fibrillation as a risk factor for atrial fibrillation in offspring. JAMA. 2004; 291: 2851-2855.

12 Du X, Dong J, Ma C. Is Atrial Fibrillation a Preventable Disease? J Am Coll Cardiol. 2017; 69: 1968-1982.

13 Bayes de Luna A, Baranchuk A, Martinez-Selles M, Platonov PG. Anticoagulation in patients at high risk of stroke without documented atrial fibrillation. Time for a paradigm shift? Ann Noninvasive Electrocardiol. 2017; 22.

14 Li J, Zhao F, Wang Y, Chen J, Tao J, Tian G, et al. Gut microbiota dysbiosis contributes to the development of hypertension. Microbiome. 2017; 5: 14.

15 Qin J, Li Y, Cai Z, Li S, Zhu J, Zhang F. et al. A metagenome-wide association study of gut microbiota in type 2 diabetes. Nature. 2012; 490: 55-60.

16 Chang CJ, Lin CS, Lu CC, Martel J, Ko YF, Ojcius DM, et al. Ganoderma lucidum reduces obesity in mice by modulating the composition of the gut microbiota. Nat Commun. 2015; 6: 7489.

17 Qin N, Yang F, Li A, Prifti E, Chen Y, Shao L, et al. Alterations of the human gut microbiome in liver cirrhosis. Nature. 2014; 513: 59-64.

18 Feng Q, Liang S, Jia H, Stadlmayr A, Tang L, Lan Z, et al. Gut microbiome development along the colorectal adenoma-carcinoma sequence. Nat Commun. 2015; 6: 6528.

19 Zhang X, Zhang D, Jia H, Feng Q, Wang D, Liang D, et al. The oral and gut microbiomes are perturbed in rheumatoid arthritis and partly normalized after treatment. Nat Med. 2015; 21: 895-905.

20 Jie Z, Xia H, Zhong SL, Feng Q, Li S, Liang S, et al. The gut microbiome in atherosclerotic cardiovascular disease. Nat Commun. 2017; 8: 845.

21 Su T, Liu R, Lee A, Long Y, Du L, Lai S, et al. Altered Intestinal Microbiota with Increased Abundance of Prevotella Is Associated with High Risk of Diarrhea-Predominant Irritable Bowel Syndrome. Gastroenterol Res Pract. 2018; 2018: 6961783.

22 Foster JA, McVey Neufeld KA. Gut-brain axis: how the microbiome influences anxiety and depression. Trends Neurosci. 2013; 36: 305-312.

23 Schirmer M, Smeekens SP, Vlamakis H, Jaeger M, Oosting M, Franzosa EA, et al. Linking the Human Gut Microbiome to Inflammatory Cytokine Production Capacity. Cell. 2016; 167: 1897.

24 Dzeshka MS, Shahid F, Shantsila A, Lip GYH. Hypertension and Atrial Fibrillation: An Intimate Association of Epidemiology, Pathophysiology, and Outcomes. Am J Hypertens. 2017; 30: 733-755.

25 Yu L, Meng G, Huang B, Zhou X, Stavrakis S, Wang M, et al. A potential relationship between gut microbes and atrial fibrillation: Trimethylamine N-oxide, a gut microbe-derived metabolite, facilitates the progression of atrial fibrillation. Int J Cardiol. 2018; 255: 92-98.

26 Pastori D, Carnevale R, Nocella C, Novo M, Santulli M, Cammisotto V, et al. Gut-Derived Serum Lipopolysaccharide is Associated With Enhanced Risk of Major Adverse Cardiovascular Events in Atrial Fibrillation: Effect of Adherence to Mediterranean Diet. J Am Heart Assoc. 2017; 6.

27 Fretts AM, Mozaffarian D, Siscovick DS, Djousse L, Heckbert SR, King IB, et al. Plasma phospholipid saturated fatty acids and incident atrial fibrillation: the Cardiovascular Health Study. J Am Heart Assoc. 2014; 3: e000889.

28 Wu S, Huang Z, Yang X, Zhou Y, Wang A, Chen L, et al. Prevalence of ideal cardiovascular health and its relationship with the 4-year cardiovascular events in a northern Chinese industrial city. Circ Cardiovasc Qual Outcomes. 2012; 5: 487-493.

29 Arumugam M, Raes J, Pelletier E, Le Paslier D, Yamada T, Mende DR, et al. Enterotypes of the human gut microbiome. Nature. 2011; 473: 174-180.

30 Qin J, Li R, Raes J, Arumugam M, Burgdorf KS, Manichanh C, et al. A human gut microbial gene catalogue established by metagenomic sequencing. Nature. 2010; 464: 59-65.

31 Greenblum S, Turnbaugh PJ, Borenstein E. Metagenomic systems biology of the human gut microbiome reveals topological shifts associated with obesity and inflammatory bowel disease. Proc Natl Acad Sci U S A. 2012; 109: 594-599.

32 Nielsen HB, Almeida M, Juncker AS, Rasmussen S, Li J, Sunagawa S, et al. Identification and assembly of genomes and genetic elements in complex metagenomic samples without using reference genomes. Nat Biotechnol. 2014; 32: 822-828.

33 Kirchhof P, Benussi S, Kotecha D, Ahlsson A, Atar D, Casadei B, et al. 2016 ESC Guidelines for the management of atrial fibrillation developed in collaboration with EACTS. Eur Heart J. 2016; 37: 2893-2962.

34 Joossens M, Huys G, Cnockaert M, De Preter V, Verbeke K, Rutgeerts P, et al. Dysbiosis of the faecal microbiota in patients with Crohn's disease and their unaffected relatives. Gut. 2011; 60: 631-637.

35 Hoffmann TW, Pham HP, Bridonneau C, Aubry C, Lamas B, Martin-Gallausiaux C, et al. Microorganisms linked to inflammatory bowel disease-associated dysbiosis differentially impact host physiology in gnotobiotic mice. ISME J. 2016; 10: 460-477.

36 Machiels K, Sabino J, Vandermosten L, Joossens M, Arijs I, de Bruyn M, et al. Specific members of the predominant gut microbiota predict pouchitis following colectomy and IPAA in UC. Gut. 2017; 66: 79-88.

37 Cui X, Ye L, Li J, Jin L, Wang W, Li S, et al. Metagenomic and metabolomic analyses unveil dysbiosis of gut microbiota in chronic heart failure patients. Sci Rep. 2018; 8: 635.

38 Hurst JR, Kasper KJ, Sule AN, McCormick JK. Streptococcal pharyngitis and rheumatic heart disease: the superantigen hypothesis revisited. Infect Genet Evol. 2018; 61: 160-175.

39 Brook, I. Veillonella infections in children. J Clin Microbiol. 1996; 34: 1283-1285.

40 Zheng JX, Wu Y, Lin ZW, Pu ZY, Yao WM, Chen Z, et al. Characteristics of and Virulence Factors Associated with Biofilm Formation in Clinical Enterococcus faecalis Isolates in China. Front Microbiol. 2017; 8: 2338.

41 Miquel S, Martin R, Rossi O, Bermudez-Humaran LG, Chatel JM, Sokol H, et al. Faecalibacterium prausnitzii and human intestinal health. Curr Opin Microbiol. 2013; 16: 255-261.

42 Sokol H, Pigneur B, Watterlot L, Lakhdari O, Bermudez-Humaran, LG, Gratadoux JJ, et al. Faecalibacterium prausnitzii is an anti-inflammatory commensal bacterium identified by gut microbiota analysis of Crohn disease patients. Proc Natl Acad Sci U S A. 2008; 105: 16731-16736.

43 Newton RJ, McLellan SL, Dila DK, Vineis JH, Morrison HG, Eren AM, et al. Sewage reflects the microbiomes of human populations. MBio. 2015; 6: e02574.

44 Jiang H, Ling Z, Zhang Y, Mao H, Ma Z, Yin Y, et al. Altered fecal microbiota composition in patients with major depressive disorder. Brain Behav Immun. 2015; 48: 186-194.

45 Nagai F, Morotomi M, Watanabe Y, Sakon H, Tanaka R. Alistipes indistinctus sp. nov. and Odoribacter laneus sp. nov., common members of the human intestinal microbiota isolated from faeces. Int J Syst Evol Microbiol. 2010; 60: 1296-1302.

46 Finegold S, Summanen P, Hunt Gerardo S, Baron E. Clinical importance of Bilophila wadsworthia. Eur J Clin Microbiol Infect Dis. 1992; 11: 1058-1063.

47 Kasai C, Sugimoto K, Moritani I, Tanaka J, Oya Y, Inoue H, et al. Comparison of the gut microbiota composition between obese and non-obese individuals in a Japanese population, as analyzed by terminal restriction fragment length polymorphism and next-generation sequencing. BMC Gastroenterol. 2015; 15: 100.

48 Fu J, Bonder MJ, Cenit MC, Tigchelaar EF, Maatman A, Dekens JA, et al. The Gut Microbiome Contributes to a Substantial Proportion of the Variation in Blood Lipids. Circ Res. 2015; 117: 817-824.

49 Zhernakova A, Kurilshikov A, Bonder MJ, Tigchelaar EF, Schirmer M, Vatanen T, et al. Population-based metagenomics analysis reveals markers for gut microbiome composition and diversity. Science. 2016; 352: 565-569.

50 Kanehisa M, Araki M, Goto S, Hattori M, Hirakawa M, Itoh M, et al. KEGG for linking genomes to life and the environment. Nucleic Acids Res. 2008; 36: D480-484.

51 Huerta-Cepas J, Szklarczyk D, Forslund K, Cook H, Heller D, Walter MC, et al. eggNOG 4.5: a hierarchical orthology framework with improved functional annotations for eukaryotic, prokaryotic and viral sequences. Nucleic Acids Res. 2016; 44: D286-293.

52 Gao H, Chen LJ, Luo QQ, Liu XX, Hu Y, Yu LL, et al. Effect of cholic acid on fetal cardiac myocytes in intrahepatic choliestasis of pregnancy. J Huazhong Univ Sci Technolog Med Sci. 2014; 34: 736-739.

53 Farvid MS, Ding M, Pan A, Sun Q, Chiuve SE, Steffen LM, et al. Dietary linoleic acid and risk of coronary heart disease: a systematic review and meta-analysis of prospective cohort studies. Circulation. 2014; 130: 1568-1578.

54 Fung TT, Rexrode KM, Mantzoros CS, Manson JE, Willett WC, Hu FB. Mediterranean diet and incidence of and mortality from coronary heart disease and stroke in women. Circulation. 2009; 119: 1093-1100.

55 Al-Shudiefat AA, Sharma AK, Bagchi AK, Dhingra S, Singal PK. Oleic acid mitigates TNF-alpha-induced oxidative stress in rat cardiomyocytes. Mol Cell Biochem. 2013; 372: 75-82.

56 Menni C, Lin C, Cecelja M, Mangino M, Matey-Hernandez ML, Keehn L, et al. Gut microbial diversity is associated with lower arterial stiffness in women. Eur Heart J. 2018; 39: 2390-2397.

57 Zhou X, Li J, Guo J, Geng B, Ji W, Zhao Q, et al. Gut-dependent microbial translocation induces inflammation and cardiovascular events after ST-elevation myocardial infarction. Microbiome. 2018; 6: 66.

58 Yin J, Liao SX, He Y, Wang S, Xia GH, Liu FT, et al. Dysbiosis of Gut Microbiota With Reduced Trimethylamine-N-Oxide Level in Patients With Large-Artery Atherosclerotic Stroke or Transient Ischemic Attack. J Am Heart Assoc. 2015; 4.

59 Louis P, Flint HJ. Diversity, metabolism and microbial ecology of butyrate-producing bacteria from the human large intestine. FEMS Microbiol Lett. 2009; 294: 1-8.

60 Maruhashi T, Soga J, Fujimura N, Idei N, Mikami S, Iwamoto Y, et al. Endothelial Dysfunction, Increased Arterial Stiffness, and Cardiovascular Risk Prediction in Patients With Coronary Artery Disease: FMD-J (Flow-Mediated Dilation Japan) Study A. J Am Heart Assoc. 2018; 7.

61 Brandes RP. Endothelial dysfunction and hypertension. Hypertension. 2014; 64: 924-928.

62 Zhao L, Zhang F, Ding X, Wu G, Lam YY, Wang X, et al. Gut bacteria selectively promoted by dietary fibers alleviate type 2 diabetes. Science. 2018; 359: 1151-1156.

63 Scher JU, Sczesnak A, Longman RS, Segata N, Ubeda C, Bielski C, et al. Expansion of intestinal Prevotella copri correlates with enhanced susceptibility to arthritis. Elife. 2013; 2: e01202.

64 Hofer U. Microbiome: anelloviridae go viral. Nat Rev Microbiol. 2014; 12: 4-5.

65 Brown JM, Hazen SL. Microbial modulation of cardiovascular disease. Nat Rev Microbiol. 2018; 16: 171-181.

66 Boden G. Obesity and free fatty acids. Endocrinol Metab Clin North Am. 2008; 37: 635-646, viii-ix.

67 Lin YK, Chen YC, Kao YH, Tsai CF, Yeh YH, Huang JL, et al. A monounsaturated fatty acid (oleic acid) modulates electrical activity in atrial myocytes with calcium and sodium dysregulation. Int J Cardiol. 2014; 176: 191-198.

68 O'Connell RP, Musa H, Gomez MS, Avula UM, Herron TJ, Kalifa J, et al. Free Fatty Acid Effects on the Atrial Myocardium: Membrane Ionic Currents Are

Remodeled by the Disruption of T-Tubular Architecture. PLoS One. 2015; 10: e0133052.

69 Kirchhof P, Benussi S, Kotecha D, Ahlsson A, Atar D, Casadei B, et al. 2016 ESC Guidelines for the management of atrial fibrillation developed in collaboration with EACTS. Europace. 2016; 18: 1609-1678.

70 Vizzardi E, Curnis A, Latini MG, Salghetti F, Rocco E, Lupi L, et al. Risk factors for atrial fibrillation recurrence: a literature review. J Cardiovasc Med (Hagerstown). 2014; 15: 235-253.

Figure 1. Elevated microbiota richness and altered community types in AF patients.

Click here to download Figure Figure 1.pdf

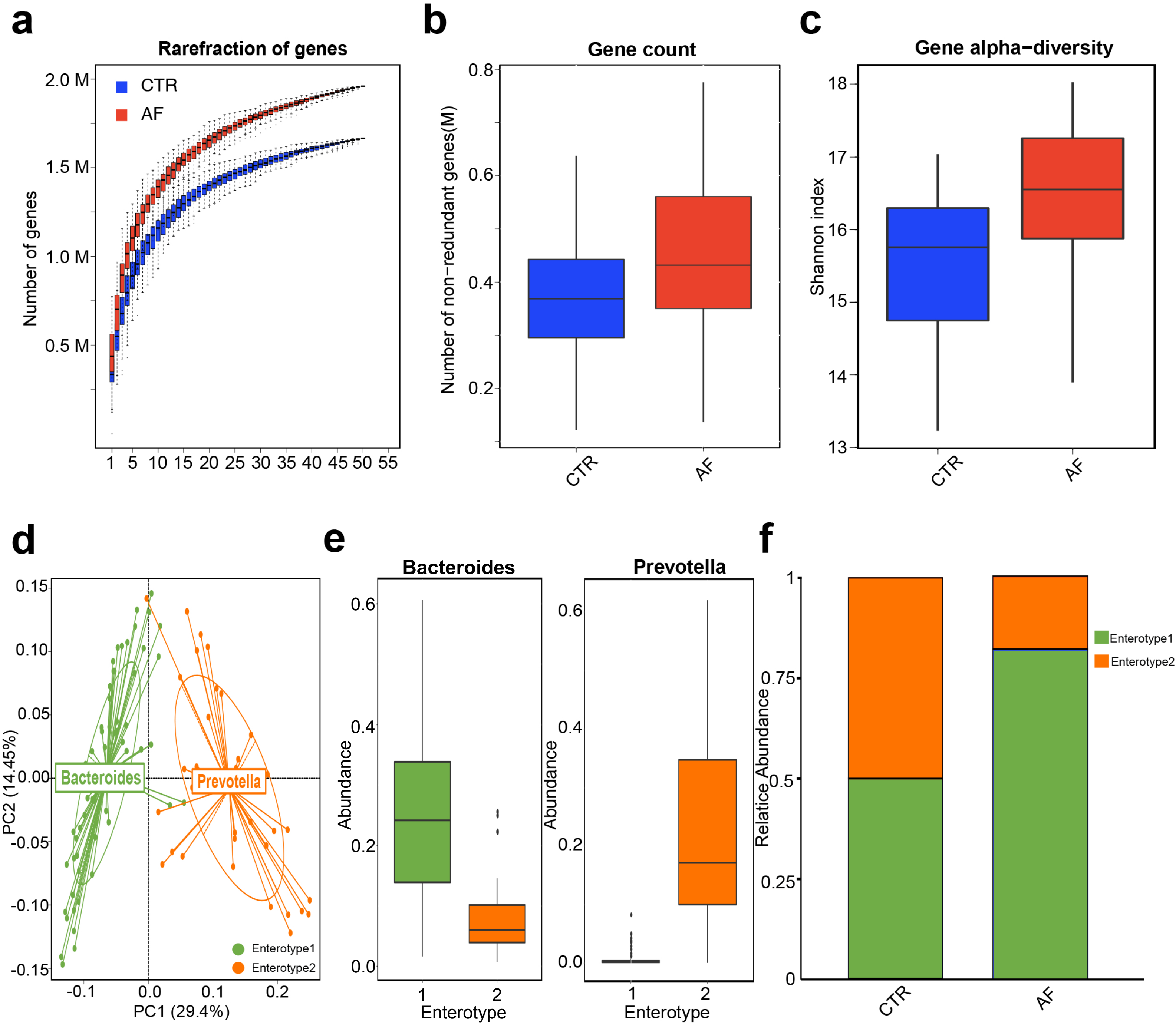

Figure 2. Genera strikingly different across groups.

Click here to download Figure Figure 2.pdf

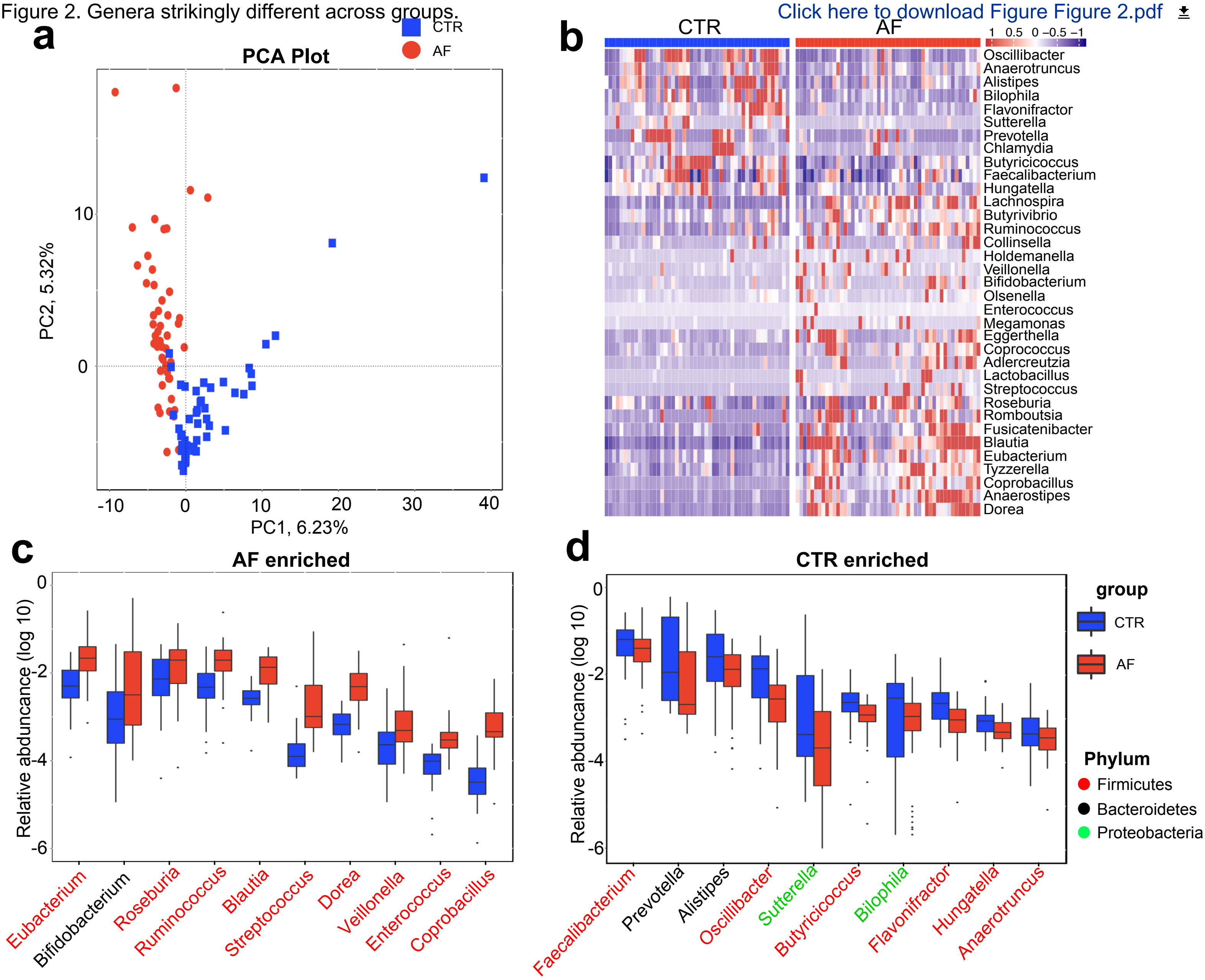

Figure 3. The network of CAGs enriched in AF compared with controls.

Click here to download Figure Figure 3.pdf

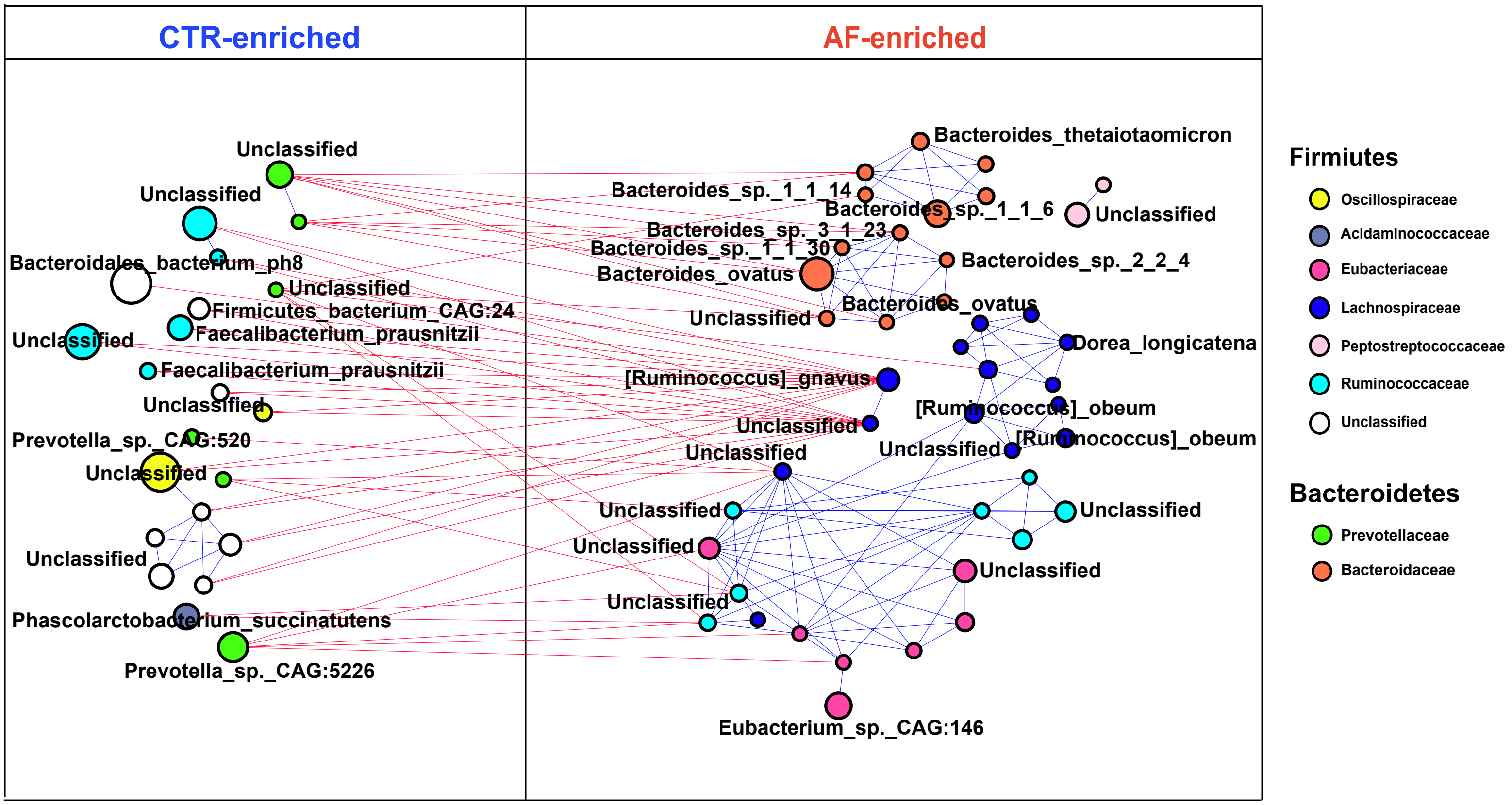

Figure 4. Gut CAGs classify AF from controls.

Click here to download Figure Figure 4.pdf

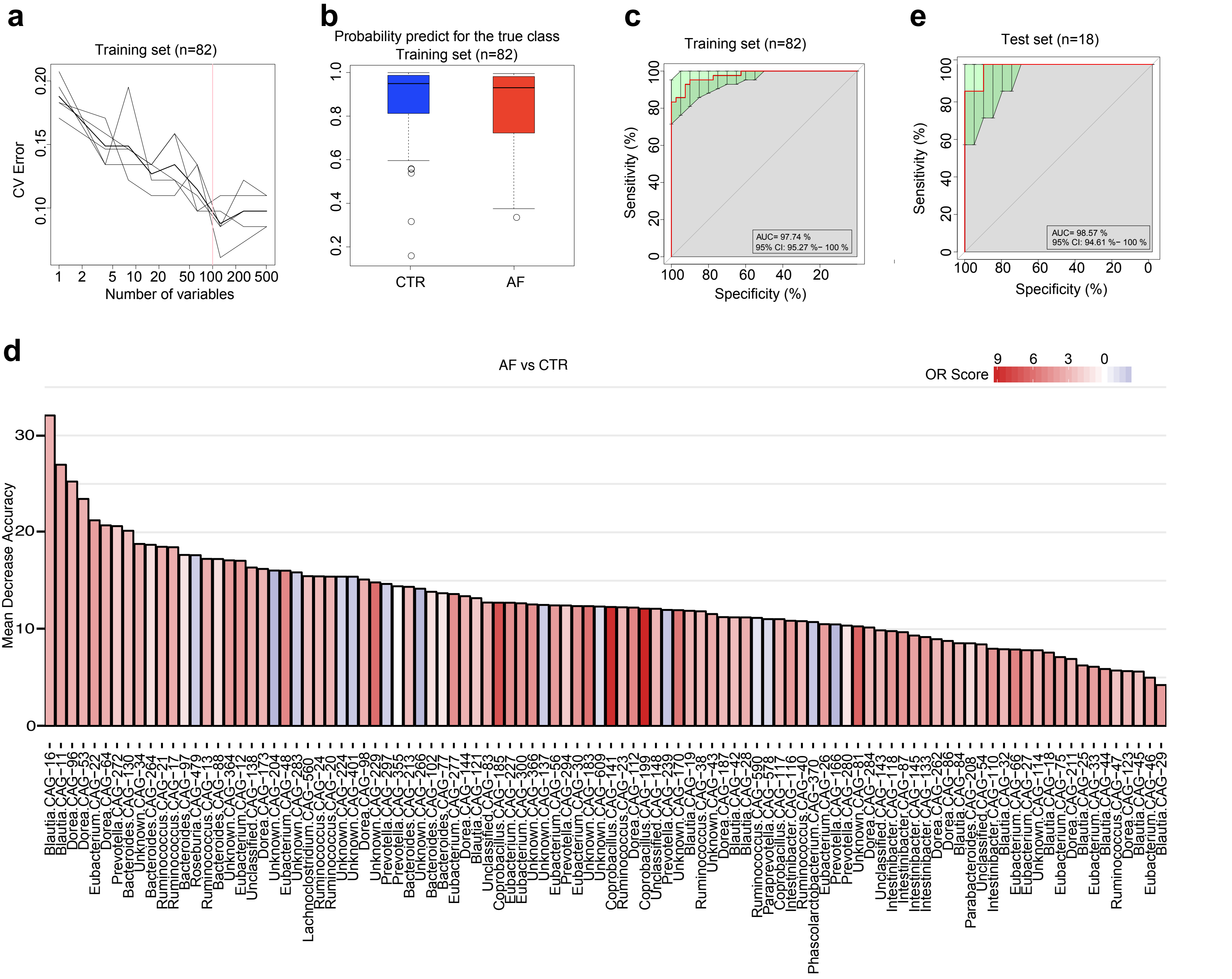

Figure 5. Microbial gene functions annotation in AF.

Click here to download Figure Figure 5.pdf

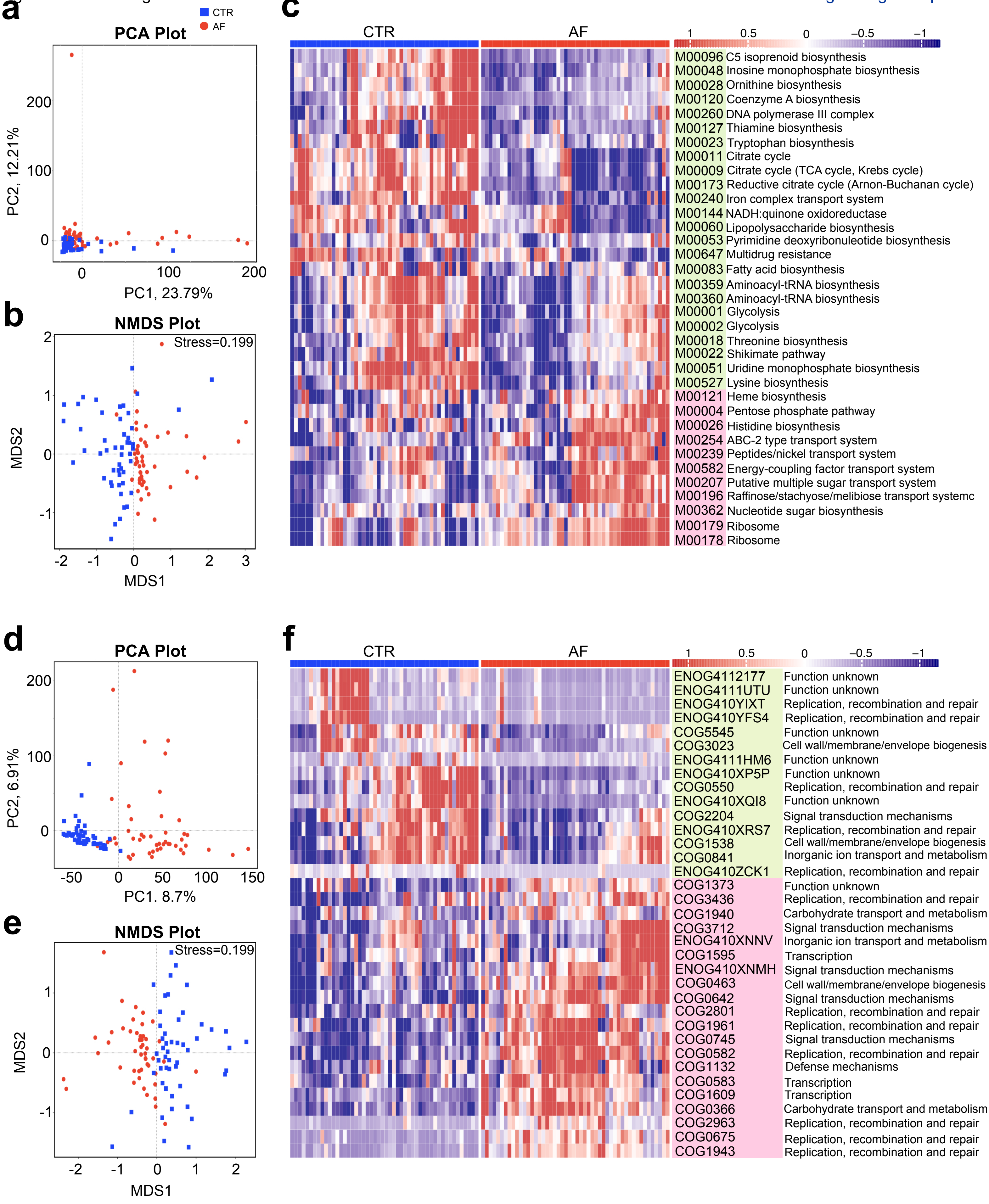

Figure 6. Distinguished metabolic patterns between AF and control

Click here to download Figure Figure 6.pdf

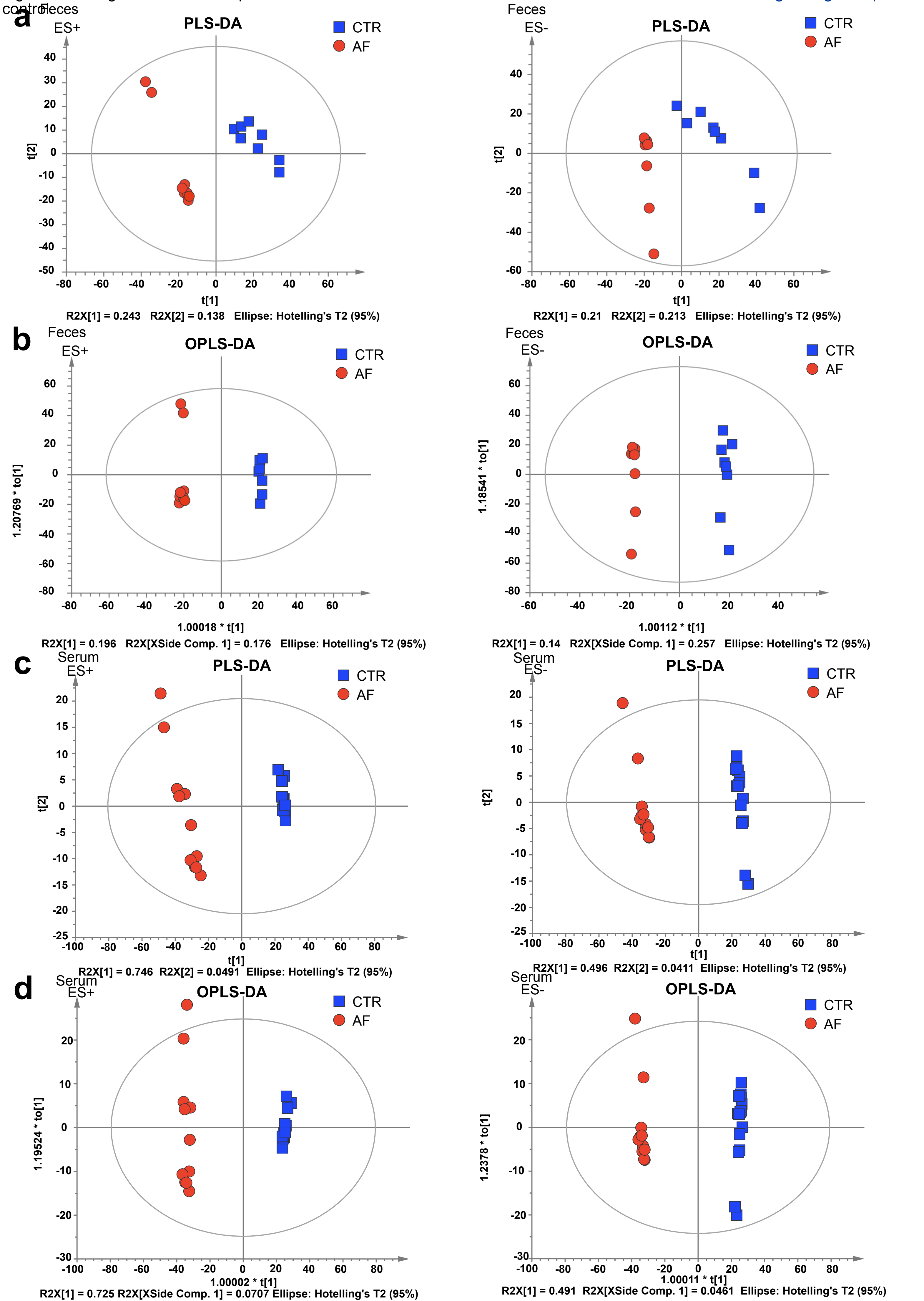

Figure 7. Aberrant metabolic patterns related to AF

Click here to download Figure Figure 7.pdf

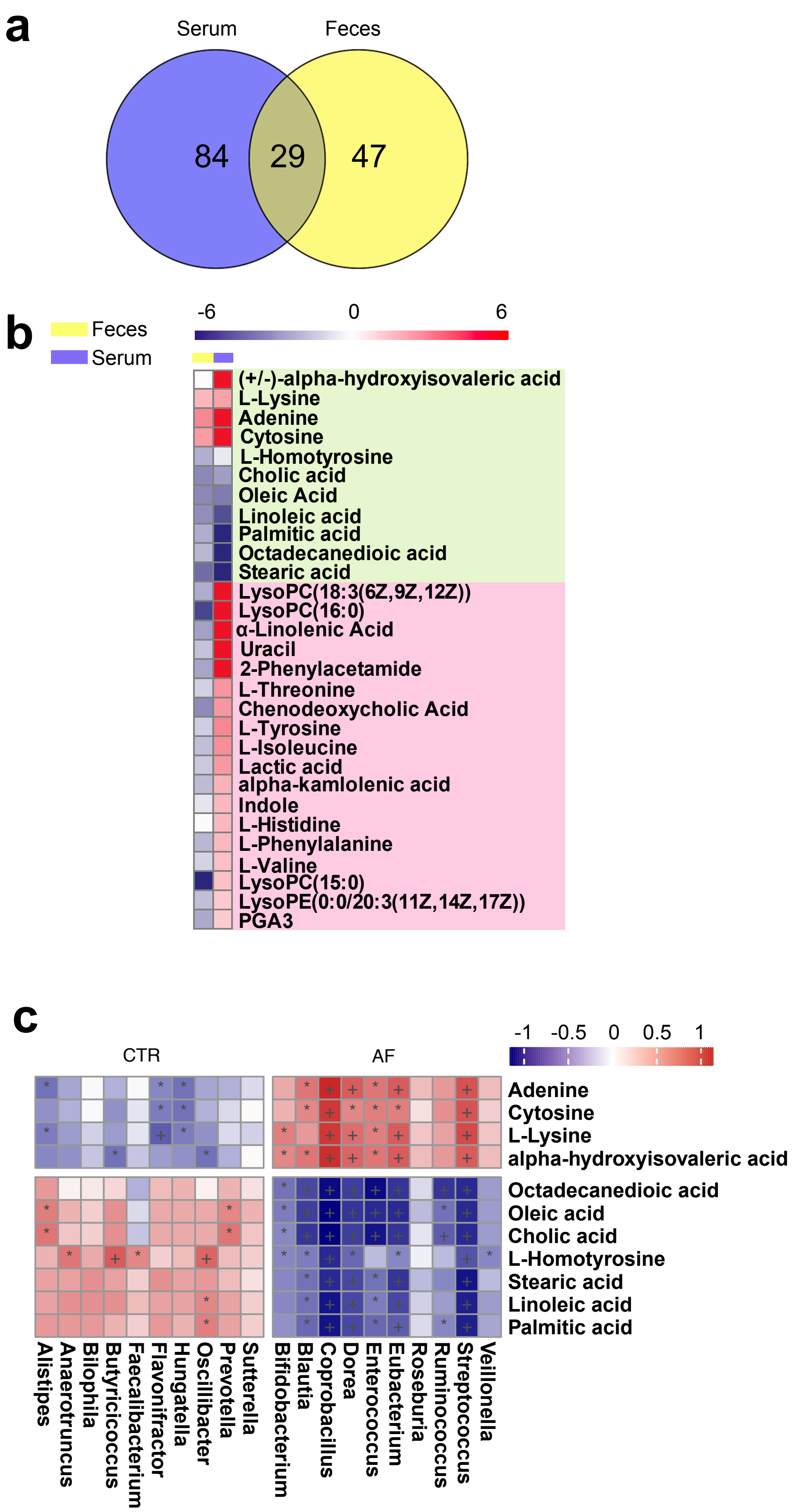

Supplementary Material Figure S1. Increased Firmicutes/Bacteroidetes ratio, Pielou evenness and Chao

Click here to access/download
Supplementary Material
Figure S1.pdf

Supplementary Material Figure S2. Another 12 genera significantly enriched in enterotype 1.

Click here to access/download
Supplementary Material
Figure S2.pdf

Supplementary Material Figure S3. Taxonomic annotation and abundance profiling.

Click here to access/download
Supplementary Material
Figure S3.pdf

Supplementary Material Figure S4. Species strikingly different across groups.

Click here to access/download
Supplementary Material
Figure S4.pdf

Supplementary Material Figure S5. Mediation effect of T2DM/TC during the process from disordered gut microbiota to AF.

Click here to access/download
Supplementary Material
Figure S5.pdf

Supplementary Material Figure S6. Insignificant influents of statins and metformin usage on GM.

Click here to access/download
Supplementary Material
Figure S6.pdf

Supplementary Material Figure S7. Gut CAGs (variables in 5, 10, 20, 50, 70) classify AF from controls.

Click here to access/download
Supplementary Material
Figure S7.pdf

Supplementary Material Figure S8. Metabolites differentially enriched in AF and controls in serum.

Click here to access/download
Supplementary Material
Figure S8.pdf

Supplementary Material Figure S9. Metabolites differentially enriched in AF and controls in feces.

Click here to access/download

Supplementary Material

Figure S9.pdf

Supplementary Material Table S1-S12, S15

Click here to access/download
Supplementary Material
renamed_f89dc.xlsx

Supplementary Material Table S13. Baseline characteristics of serum samples from metabolomic analyses.

Click here to access/download
Supplementary Material
Table S13.docx

Supplementary Material Table S14. Baseline characteristics of feces samples from metabolomic analyses.

Click here to access/download
Supplementary Material
Table S14.docx

cover letter Click here to download Personal Cover Cover letter from Kun Zuo GS.docx

Dear Editor,

We would like to submit our manuscript entitled "Disordered gut microbiota and alterations in metabolic patterns are associated with atrial fibrillation" to ***GigaScience*** for consideration as an Article. All of the authors have approved the enclosed manuscript, and no conflicts of interest exist. On behalf of all of the authors, I declare that the work described is original research that has not been previously published nor is under consideration for publication elsewhere, in whole or in part.

Atrial Fibrillation (AF) is one of the most common cardiac arrhythmias with worldwide prevalence, increasing disability and morbidity. It has become a major global public health concern. Although a correlation between gut microbiota (GM) and AF has recently been reported in a canine study, the signature change in gut microbes and their fermentation products in human populations with AF remains largely unknown. The direct evidence of altered gut bacteria in AF patients and gut dysbiosis contribution to aberrant metabolic patterns that accelerate AF progression still needs to be identified.

For the purpose above, we performed metagenomic sequencing and analyses of stool samples from patients with AF outlining the potential compositional and functional alterations of GM in 100 Chinese participants. Beyond exploring the relationship between disordered GM and altered metabolomic profiles in AF, we also aimed to construct a microbiota-dependent discrimination index for distinguishing AF, and thus provide a comprehensive understanding of GM dysbiosis in the progression of AF. Our findings describe the disordered patterns of GM and aberrant microbial-related metabolites in a cohort of AF patients for the first time. Specifically, our main novel findings are listed as follows:

(1) We reported for the first time the global alterations occurring in the intestinal microbiota of AF patients, including a dramatic elevation in microbial richness and diversity, a disorder in gut enterotype distribution and a specific perturbation of GM composition.

(2) We observed an imbalance of gut microbial function and changes in metabolic patterns in the fecal and serum samples from the AF group; we were able to identify a correlation between the GM and their endogenous metabolic products in AF patients.

(3) We captured the microbial features for AF patients, revealed their common microbial characteristics and highlighted the potential clinical value of GM in distinguishing AF by constructing a random forest disease classifier.

Recently, there has been extensive attention directed towards the gut microbiome in the development of AF but limited evidence has hence surfaced. We believe that our study directly demonstrates the crucial contribution of disordered GM to AF pathogenesis, and therefore is of substantial interest to the readers of ***GigaScience***.

We greatly appreciate your interest and encouragement concerning our manuscript. We look forward to receiving comments from you and the reviewers. If you have any questions, please do not hesitate to contact me at the address below.

Sincerely yours,

Xinchun Yang, MD, PhD

Heart Center, Beijing ChaoYang Hospital, Capital Medical University,

Beijing Key Laboratory of Hypertension,

8th Gongtinanlu Rd, Chaoyang District, Beijing, China, 100020

Tel: 86-10-85231937

Fax: 86-10-85231937

E-mail: yxc6229@163.com

Jing Li, MD, PhD

Heart Center, Beijing ChaoYang Hospital, Capital Medical University,

Beijing Key Laboratory of Hypertension,

8th Gongtinanlu Rd, Chaoyang District, Beijing, China, 100020

Tel: 86-10-85231937

Fax: 86-10-85231937

E-mail: lijing11999@126.com
