# Supplementary material for: Disordered gut microbiota and alterations in metabolic patterns are associated with atrial fibrillation
Source: Gigascience. 2019 May 30;8(6):giz058. doi: 10.1093/gigascience/giz058 (PMC6543127; doi:10.1093/gigascience/giz058)
Supplement: giz058_GIGA-D-18-00364_Revision_2 [file giz058_giga-d-18-00364_revision_2.pdf]

GigaScience

Disordered gut microbiota and alterations in metabolic patterns are associated with atrial fibrillation

--Manuscript Draft--

Manuscript Number:	GIGA-D-18-00364R2	
Full Title:	Disordered gut microbiota and alterations in metabolic patterns are associated with atrial fibrillation	
Article Type:	Research	
Funding Information:	National Natural Science Foundation of China (81670214)	Dr. Xinchun Yang
	National Natural Science Foundation of China (81500383)	Dr. Jing Li
	National Natural Science Foundation of China (81870308)	Dr. Jing Li
	National Natural Science Foundation of China (81770253)	Dr. Jiuchang Zhong
	National Natural Science Foundation of China (81370362)	Dr. Jiuchang Zhong
	Beijing Natural Science Foundation (7172080)	Dr. Xinchun Yang
	Beijing Municipal Administration of Hospitals’ Youth Programme (QML20170303)	Dr. Jing Li
	1351 personnel training plan (CYMY-2017-03)	Dr. Xinchun Yang

Abstract:

Background: With the establishment of the heart-gut axis concept, accumulating studies suggest that the gut microbiome plays an important role in the pathogenesis of cardiovascular diseases. Yet, little evidence has been reported in characterizing the gut microbiota shift in atrial fibrillation.
Methods: We include the result of the global alterations that occur in the intestinal microbiota in a cohort of 50 atrial fibrillation patients and 50 matched controls based on a strategy of metagenomic and metabolomic analyses.
Results: The alterations include a dramatic elevation in microbial diversity, and a specific perturbation of gut microbiota composition. Overgrowth of Ruminococcus, Streptococcus and Enterococcus, as well as reduction of Faecalibacterium, Alistipes, Oscillibacter, and Bilophila were detected in atrial fibrillation patients. A gut microbial function imbalance and correlated metabolic pattern changes were observed with atrial fibrillation in both fecal and serum samples. The differential gut microbiome signatures could be used to identify atrial fibrillation patients.
Conclusion: Our findings characterize the disordered gut microbiota and microbial metabolite profiles in atrial fibrillation. Intervention strategies targeting intestinal microbiome composition to counteract the progression of atrial fibrillation are highly suggested.

Corresponding Author:	Xinchun Yang<br><br>CHINA
Corresponding Author Secondary Information:	
Corresponding Author's Institution:	
Corresponding Author's Secondary Institution:	
First Author:	Kun Zuo

Powered by Editorial Manager® and ProduXion Manager® from Aries Systems Corporation

**First Author Secondary Information:**	
**Order of Authors:**	Kun Zuo
	Jing Li
	Kuibao Li
	Chaowei Hu
	Yuanfeng Gao
	Mulei Chen
	Roumu Hu
	Ye Liu
	Hongjie Chi
	Hongjiang Wang
	Yanwen Qin
	Xiaoyan Liu
	Shichao Li
	Jun Cai
	Jiuchang Zhong
	Xinchun Yang
**Order of Authors Secondary Information:**	
**Response to Reviewers:**	Dear editor,<br><br>Thank you very much for your consideration and encouragement on our manuscript entitled "Disordered gut microbiota and alterations in metabolic patterns are associated with atrial fibrillation" (GIGA-D-18-00364R1). We thank reviewers for their professional and positive comments to improve our manuscript. We have considered the comments from the editors and reviewers and revised the manuscript.<br>Furthermore, we have made the raw metabolomics MS data openly available under Metabolomics Workbench and add the accession details in the manuscript. The metabolomics data was available at the NIH Common Fund's Data Repository and Coordinating Center website with Metabolomics Workbench Study ID: ST001168 (for fecal metabolomic analyses) and ST001169 (for serum metabolomic analyses). And the data are directly accessible at<br>http://dev.metabolomicsworkbench.org:22222/data/DRCCMetadata.php?Mode=Study&StudyID=ST001168 (for fecal metabolomic analyses);<br>http://dev.metabolomicsworkbench.org:22222/data/DRCCMetadata.php?Mode=Study&StudyID=ST001169 (for serum metabolomic analyses) (page 36, #line 715 in the revision).<br><br>Point-by-point replies are listed as below.<br>A list of the changes in Tables<br>Table S15 was updated with report of ppm and change “0” to “/” in column of “score”.<br><br>Respond to Reviewer #1:<br>The authors have improved the manuscript sustainably. I have some small comment for authors to check and report in the manuscript.<br>Question: 1) When the authors identified metabolite names from exact mass using METLIN please specify the tolerant cut-off in ppm. The ppm different result obtain from METLIN query needs to be reported in supplementary the table S15.<br>Response: Thanks for this valuable suggestion. Indeed, ppm value is important during the process of metabolites identification, which refers to the error between molecular mass detected by the instrument and the molecular mass in the database. The lower ppm means the closer molecular mass detected by the instrument and the molecular mass in the database. The cut-off value of ppm in the current study is set less than 25. The ppm value in this study is mainly lower than 10, which has been supplemented in

	table S15 (page 34, #line 674 in the revision; page 34, #line 673 in the clean revision).<br><br>Question: 2) -In supplementary table S 15 why score of L-Threonine and α-Linolenic Acid are 0 ??<br>Response: Thanks for this suggestion. It would be reasonable to change “0” to “/” in column of “score” in table S15. Because the score of L-Threonine and α-Linolenic were failed to find in the database. The second-order mass spectrometry is even more difficult to find due to the low intensity of the parent ion in sample. Therefore, the exact molecular mass was used to identify the metabolites related to the featured peak in the Metlin database (http://metlin.scripps.edu).<br><br>We greatly appreciate your interest and encouragement concerning our manuscript. We look forward to receiving comments from you. If you have any questions, please don't hesitate to contact me at the address below.<br><br>Sincerely yours,<br><br>Xinchun Yang, MD, PhD<br>Heart Center, Beijing ChaoYang Hospital, Capital Medical University,<br>Beijing Key Laboratory of Hypertension,<br>8th Gongtinanlu Rd, Chaoyang District, Beijing, China, 100020<br>Tel: 86-10-85231937<br>Fax: 86-10-85231937<br>E-mail: yxc6229@163.com<br><br>Jiuchang Zhong, MD, PhD<br>Heart Center, Beijing ChaoYang Hospital, Capital Medical University,<br>Beijing Key Laboratory of Hypertension,<br>8th Gongtinanlu Rd, Chaoyang District, Beijing, China, 100020<br>Tel: 86-10-85231937<br>Fax: 86-10-85231937<br>E-mail: jiuchangzhong@aliyun.com
**Additional Information:**	
**Question**	**Response**
Are you submitting this manuscript to a special series or article collection?	No
**Experimental design and statistics**<br><br>Full details of the experimental design and statistical methods used should be given in the Methods section, as detailed in our Minimum Standards Reporting Checklist. Information essential to interpreting the data presented should be made available in the figure legends.<br><br>Have you included all the information requested in your manuscript?	Yes
**Resources**	Yes

A description of all resources used, including antibodies, cell lines, animals and software tools, with enough information to allow them to be uniquely identified, should be included in the Methods section. Authors are strongly encouraged to cite Research Resource Identifiers (RRIDs) for antibodies, model organisms and tools, where possible.<br><br>Have you included the information requested as detailed in our Minimum Standards Reporting Checklist?	
**Availability of data and materials**<br><br>All datasets and code on which the conclusions of the paper rely must be either included in your submission or deposited in publicly available repositories (where available and ethically appropriate), referencing such data using a unique identifier in the references and in the "Availability of Data and Materials" section of your manuscript.<br><br>Have you have met the above requirement as detailed in our Minimum Standards Reporting Checklist?	Yes

Powered by Editorial Manager® and ProduXion Manager® from Aries Systems Corporation

Manuscript with DOI
Click here to view linked References
Click here to access/download;Manuscript;revised clean version-2-2019-4-12 WITH DOI.docx

Disordered gut microbiota and alterations in metabolic patterns are associated with atrial fibrillation

Kun Zuo[1#], Jing Li[1#], Kuibao Li[1], Chaowei Hu[2], Yuanfeng Gao[1], Mulei Chen[1], Roumu Hu[1], Ye Liu[1], Hongjie Chi[1], Hongjiang Wang[1], Yanwen Qin[2], Xiaoyan Liu[3], Shichao Li[1], Jun Cai[4], Jiuchang Zhong[1*], Xinchun Yang[1*].

1 Heart Center & Beijing Key Laboratory of Hypertension, Beijing Chaoyang Hospital, Capital Medical University, Beijing 100020, China

2 The Key Laboratory of Upper Airway Dysfunction-related Cardiovascular Diseases, Beijing An Zhen Hospital, Capital Medical University, Beijing Institute of Heart, Lung and Blood Vessel Diseases, Beijing 100029, China

3 Medical Research Center, Beijing Chaoyang Hospital, Capital Medical University, Beijing 100020, China

4 Hypertension Center, Fuwai Hospital, State Key Laboratory of Cardiovascular Disease of China, National Center for Cardiovascular Diseases of China, Chinese Academy of Medical Sciences and Peking Union Medical College, Beijing 100037, China

Author email addresses

Kun Zuo, zuokun699@163.com

Jing Li, lijing11999@126.com

Kuibao Li, kuibaoli@126.com

Chaowei Hu, halohu123@sina.com

Yuanfeng Gao, gaoyuanwind1@163.com

Mulei Chen, cml68@sina.cn

Roumu Hu, roumuhu@126.com

Ye Liu, liuye8810@sina.com

Hongjie Chi, chihongjie@163.com

Hongjiang Wang, wanghongjiang@126.com

Yanwen Qin, qinyanwen@vip.126.com

Xiaoyan Liu, lxy-213@163.com

Shichao Li, lishichao3@sina.com

Jun Cai, caijun@fuwaihospital.org

Jiuchang Zhong, jiuchangzhong@aliyun.com

Xinchun Yang, yxc6229@163.com

#Equal contributors

*__Correspondence to:__

Xinchun Yang, MD, PhD

Heart Center, Beijing ChaoYang Hospital, Capital Medical University,

Beijing Key Laboratory of Hypertension,

8th Gongtinanlu Rd, Chaoyang District, Beijing, China, 100020

Tel: 86-10-85231937

Fax: 86-10-85231937

E-mail: yxc6229@163.com

Jiuchang Zhong, MD, PhD

Heart Center, Beijing ChaoYang Hospital, Capital Medical University,

Beijing Key Laboratory of Hypertension,

8th Gongtinanlu Rd, Chaoyang District, Beijing, China, 100020
Tel: 86-10-85231937
Fax: 86-10-85231937
E-mail: jiuchangzhong@aliyun.com

Abstract

Background: With the establishment of the heart-gut axis concept, accumulating studies suggest that the gut microbiome plays an important role in the pathogenesis of cardiovascular diseases. Yet, little evidence has been reported in characterizing the gut microbiota shift in atrial fibrillation.

Methods: We include the result of the global alterations that occur in the intestinal microbiota in a cohort of 50 atrial fibrillation patients and 50 matched controls based on a strategy of metagenomic and metabolomic analyses.

Results: The alterations include a dramatic elevation in microbial diversity, and a specific perturbation of gut microbiota composition. Overgrowth of *Ruminococcus*, *Streptococcus* and *Enterococcus*, as well as reduction of *Faecalibacterium*, *Alistipes*, *Oscillibacter*, and *Bilophila* were detected in atrial fibrillation patients. A gut microbial function imbalance and correlated metabolic pattern changes were observed with atrial fibrillation in both fecal and serum samples. The differential gut microbiome signatures could be used to identify atrial fibrillation patients.

Conclusion: Our findings characterize the disordered gut microbiota and microbial metabolite profiles in atrial fibrillation. Intervention strategies targeting intestinal microbiome composition to counteract the progression of atrial fibrillation are highly suggested.

Keywords: Atrial fibrillation, Gut microbiota, Metagenome, Metabolism

Background

Atrial Fibrillation (AF), an abnormal heart rhythm characterized by rapid and irregular beating of the atria, is the most common arrhythmia with heavy global burdens, intensifying disability and morbidity. In Europe and the US, one in four middle-aged adults will subject to AF [1, 2]. AF is prevalent in approximately 3% of adults at the age of 20 years or older [3], with greater prevalence in older persons and in patients with conditions such as hypertension (HTN), heart failure, obesity or type two diabetes mellitus (T2DM) [4]. AF is independently associated with a 2-fold increased risk of all-cause mortality in women and a 1.5-fold increase in men [5] and has become a significant contributor to cardiovascular events leading to cardiac death worldwide. Currently, ideal preventive and therapeutic strategies to counteract the progression of AF remain sparse. The heterogeneity of underlying atrial substrate, extent of atrial fibrosis, and the discrepancies between inter-individual electrophysiological characteristics contribute to unpredictable responses to drug or ablation therapy [6]. It is essential to embrace AF prevention as a priority, not only focusing on rate, rhythm controlling or stroke prevention but also considering AF as a concomitant factor of adverse atrial remodeling rather than a solitary disease. Therefore, efforts to identify the pathological mechanisms of AF are warranted. Various genetic mutations have been identified to be associated with AF [7] and environmental or unhealthy lifestyle factors

are also believed to contribute to the development of AF [8]. It was worth noting that AF risk factors or contributors, such as HTN, T2DM and obesity have been linked to dietary intake that possibly contributes to alterations in the composition of the gut microbiota [8-11].

Recently, more investigators have focused on the role of gut microbiome (GM), which has been identified as an essential factor affecting human health [9-14]. Dysbiotic GM has been reported in multiple diseases, such as T2DM [10], obesity [11], HTN [9], atherosclerotic cardiovascular disease [15], liver cirrhosis [12], colorectal adenoma-carcinoma [13], rheumatoid arthritis [14], irritable bowel syndrome [16], anxiety and depression [17], and shown to activate the immune system [18], eliciting chronic diseases. As the understanding of the relationship between intestinal microbiome and diseases has deepened, possible underlying mechanisms have been proposed. For example, emerging evidence suggest that through immune system and metabolic alterations, gut microbiota disequilibrium could induce obesity, HTN and T2DM, traditional cardiac risk factors that play essential role during atrial remodeling in the development of AF [8, 19]. However, data demonstrating a correlation between AF and the intestinal microbiome are still lacking. To our knowledge, studies of gut microbiota and AF have been few in number. Information regarding the impact of microbial metabolites is also incomplete. A gut microbial-dependent metabolite, trimethylamine-N-oxide (TMAO), which is positively correlated with cardiovascular disease in human, is proatherogenic and could increase the instability of atrial

electrophysiology [20]. However, it remains unclear whether circulating TMAO levels derived from the intrinsic microbiome can reach the ganglionated plexi and create local concentrations sufficient to result in comparable arrhythmogenic effects. In addition, recent studies have shown that gut-derived lipopolysaccharide (LPS) is predictive for major adverse cardiovascular events in AF patients [21]. Furthermore, microbiome-derived free fatty acids, such as palmitic (PA) and adrenic acid might have potential influences on arrhythmogenesis [22], [23].

These seminal studies provided the first clues indicating a possible interaction between gut microbiota and AF. They encouraged us to identify direct evidence of gut bacteria alterations in patients with AF and evaluate the possible contribution of gut dysbiosis to aberrant metabolic patterns that accelerate the progression of AF. We performed metagenomic sequencing analyses of stool samples from patients with AF to outline the potential compositional and functional alterations of GM. In addition, to expose the relationship between disordered GM and altered metabolomic profiles in AF, we aimed to construct a microbiota-dependent discrimination index for distinguishing AF, thus providing a comprehensive understanding of gut microbiota dysbiosis in the progression of AF. This work is fundamental for further studies to reveal the causal relationship and explore preventative measures for postponing AF progression.

Results

Baseline characteristics of the study cohort

We enrolled 100 Chinese participants consisting of 50 patients with nonvalvular AF and 50 individuals as matched controls (CTR). AF was diagnosed using an electrocardiogram and defined as the absence of P waves, replaced by disorganized electrical activity and irregular R–R intervals due to irregular conduction of impulses to the ventricles [24]. To adjust for the effect of HTN on gut microbiota composition, we selected 50 samples from our previous gut microbiota work matched for a history of HTN [9]. None of the subjects had heart failure, coronary heart disease, structural heart disease, inflammatory bowel diseases, irritable bowel syndrome, autoimmune diseases, liver diseases, renal diseases or cancer. Patients who had used antibiotics or probiotics in the last month were excluded. The clinical characteristics of all subjects are shown in Table 1. There was no significant difference between AF patients and controls in terms of body mass index, creatinine, total bilirubin or glutamic-pyruvic transaminase. Most of the patients were elderly, with 70% greater than 60 years old. For the control group, there were more males than females, with males accounting for 82%. Although the total cholesterol (TC) serum levels were much lower in patients with AF, these clinical indices were all within the normal range.

Elevated microbiota richness and altered community types in the gut of subjects with AF

Whole-metagenome shotgun sequencing of the 100 stool samples from our study cohort was performed. A total of 612.84 Gb high-quality sequencing reads were generated (6.13 ± 0.96 (s.d.) million reads per sample on average) (Additional files 1: Table S1). Rarefaction analyses, performed as we previous described [9], showed that the curves approached saturation in each group and with a significantly increased gene number in the microbiomes of patients with AF (Fig. 1a). We also compared the gene count, within-sample diversity (Shannon index) and 3 other ecological parameters, including Chao richness, Pieloueveness and Firmicutes/Bacteroidetes ratio (F/B ratio) between controls and AF patients. Consistently, gut microbial richness (gene count), diversity in AF group were much higher (P =0.007 for gene count, Fig. 1b; P =3.53e-05 for Shannon index, Fig. 1c; P=7.162e-05 for F/B ratio, Additional files 2: Fig. S1a; P=0.007633 for Chao richness, Fig. S1b; P=4.262e-06 for Pielou evenness, Fig. S1c). The elevated richness of genes or genera observed in our cohort may suggest the overgrowth of a variety of harmful bacteria in patients with AF.

To investigate the shift of gut microbiota community structure during AF state, microbial enterotype features were examined using the Partitioning Around Medoid clustering method. The 100 samples were divided into two clusters by Principal Coordinate Analysis (PCoA) based on the Jensen-Shannon divergence (Fig. 1d). Enterotype 1 was dominated by *Bacteroides* as the most enriched genus, and *Prevotella* was the core in enterotype 2 (P = 1.730774e-09 and P = 4.376078e-14, respectively; Wilcoxon rank sum test, Fig. 1e-f). Both enterotypes have been previously reported in

HTN, T2DM, colorectal cancer and irritable bowel syndrome [9, 10, 13, 16]. There were 12 other significantly increased genera in enterotype 1, including *Blautia*, *Coprobacillus*, *Dorea*, *Enterococcus, Streptococcus* and *Veillonella* (Additional files 3: Fig. S2). Interestingly, there was a dysbiosis of enterotype distribution by AF conditions. For the control group, the percentage of samples in both enterotypes was the same (50% in enterotype 1, 50% in enterotype 2), whereas a higher percentage of AF patients were found to be distributed in enterotype 1 (82%), and less in enterotype 2 (P =0.001, AF vs CTR; Fisher's exact test; Fig. 1g). Furthermore, similar difference in enterotype distribution at the species level was also found, although no significant different species were found between enterotypes (Additional files 4: Fig. S3). Therefore, a morbid state of AF is associated with imbalanced gut microbial communities, with a tendency towards the enterotype dominated by *Bacteroides* and away from the *Prevotella* prominent enterotype.

Taxonomic profile of AF-associated gut microbiota

In order to compare the taxonomic profile of gut microbiota in AF patients with those in healthy individuals, we accessed the GM abundances and phylogenetic profiles at the genus level. Genes were aligned to the NR database using DIAMOND61 (Version 0.7.9.58) and annotated to taxonomic groups (Additional files 5: Fig. S4). The relative abundance of gut microbes was calculated by summing the abundance of genes as listed in Additional files 6-7: Table S2-S3. The state of disease significantly separated the

subjects with AF or without AF in principal component analysis (PCA) analysis or in non-metric dimensional scaling (NMDS) analysis at the genus level (Additional files 5 : Fig. S4a-b). The 35 most abundant genera in AF patients and healthy controls are shown in Additional files 5: Fig. S4c.

Overall, 574 genera were dramatically different in control and AF subjects ($p< 0.05$, *p* values were tested using the Wilcoxon rank sum test and corrected for multiple testing with the Benjamin & Hochberg method [12]. Additional files 8: Table S4). And consistent results were also obtained when the PCA analysis was performed based on the genera or species differentially enriched across groups ($P < 0.05$, Anosim, genus: Fig.2a, species: Additional files 9: Fig. S5a). The top 10 different gut bacteria that dominated in AF or controls at the genus level are shown in Fig. 2c, d. In AF patients, the proportion of *Streptococcus*, *Enterococcus*, *Blautia*, *Dorea*, *Veillonella* and *Coprobacillus* were much higher than in controls (Fig. 2c), in agreement with our previous observations that they were more abundant in the AF-correlated enterotype (enterotype 1). In addition to *Eubacterium, Bifidobacterium and Roseburia*, *Ruminococcus* were also overexpressed in individuals with AF (Fig. 2c). *Ruminococcus* is known to possess a pro-inflammatory property, which was implicated in the development of inflammatory bowel disease [25-27]. Transplantation of *Ruminococcus* into germ free mice has been reported to enhance the levels of interferon-γ, interleukin-17 and interleukin-22 [26]. *Streptococcus*, recognized as a morbific oral bacteria, has also been demonstrated to be elevated in HTN [9], congestive heart failure (CHF) [28]

and atherosclerotic cardiovascular disease (ACVD) [15, 29]. Furthermore, *Veillonella*, a Gram-negative anaerobic coccus, was suggested to be inversely correlated with cardiovascular protective metabolites such as niacin, cinnamic acid and orotic acid [30]. In addition, *Enterococcus* is known to produce cytolysin, a toxin that causes rupture of a variety of target membranes, including bacterial cells, erythrocytes and other mammalian cells [31].

Of the top 10 different species in AF group shown in Fig. S5c, *Escherichia coli*, a potentially pathogenic bacteria, was the most abundant and may be correlated with the progression of AF. *Eubacterium rectale* is a main representative of *Firmicutes* and a kind of conditioned pathogen, which can ferment the metabolic products of glucose (such as formic acid, acetic acid and butyric acid) as well as proteins, thereby inhibiting the proliferation of other beneficial bacteria in the intestines and decreasing catabolic enzymes of glycan [32]. Furthermore, species enriched in AF group, including *Bifidobacterium longum*, *Collinsella aerofaciens*, were more abundant in metastatic melanoma patients [33]. Meanwhile, *Faecalibacterium prausnitzii* [34], the butyrate-producing bacterial species was found decreased in AF group. These results showed the imbalanced structure of intestinal floras, reduced probiotics and increased quantity of harmful bacteria in patients with AF. It is speculated that these clusters of conditioned pathogens accumulated in the gut might influence AF susceptibility.

Moreover, *Faecalibacterium*, *Prevotella*, *Alistipes*, *Oscillibacter* and *Sutterella*

were dramatically decreased in the AF patients compared with controls and a similar shift was found for *Butyricicoccus, Flavonifractor* and *Bilophila* (Fig. 2c). In addition, we also identified a dramatic decline of species such as *Faecalibacterium prausnitzii*, *Oscillibacter sp.*, and also *Firmicutes bacterium* in the AF patients (Fig. S5c). *Faecalibacterium prausnitzii* is a butyrate-producing commensal bacterium with anti-inflammatory properties and its deficiency may aggravate chronic inflammation, leading to ulcerative colitis, Crohn's disease, obesity, asthma and major depressive disorder [35-38]. *Alistipes* is a common member of the human intestinal microbiota, capable of producing short chain fatty acids from amino acids, such as succinic and acetic acids [39]. The enrichment of *Oscillibacter sp.* and *Alistipes* were previously reported to be essential for maintaining balanced gut microbes protecting from HTN [9], CHF [28] and ACVD [15]. In addition, *Bilophila* is found in normal flora in human feces [40] and *Flavonifractor* was enriched in the feces of non-obese subjects [41].

Considering the difference of baseline characteristics, including gender, age, T2DM diagnosis and TC levels between the two groups, we questioned whether or not the alterations of GM observed in AF patients were mediated by these clinical factors [10, 42], [43]. PCA plot was performed to assess the contribution of these factors, and the results showed that it failed to distinguish AF patients into separated group based on these factors, indicating the negligible impact of gender, age, T2DM or TC on our results ($p>0.05$, Anosim, Additional files 10, Fig. S6).

Additionally, medication is a key factor that can alter gut microbiome as shown in previous study [43, 44]. Therefore, the effects of statins and DMBG usage were further analyzed by PCA plots to assess the possible influence of drug consumption on GM in AF patients. As indicated above, there were 4 AF patients taking statins and 6 taking DMBG. The PCA at the genus level failed to separate the AF patients into different clusters based on the usage of statins or DMBG ($P > 0.05$, Anosim, Additional files 10: Fig. S6e). These findings based on the taxonomic profile of gut microbiota supported our hypothesis that there is serious dysbiosis of gut bacteria under AF state, which may play a crucial role in the pathology of atrial remodeling and the formation of an arrhythmogenic substrate.

AF state is identifiable by the gut co-abundance group

At the gene level, there were 121,145 genes differentially enriched in AF patients versus the controls (Additional files 11: Table S5). These genes were further clustered into co-abundance groups (CAGs) as we described previously [9] which generated 15,289 distinct CAGs (Additional files 12-15: Table S6-S9). The confident of taxonomic annotation confidence of individual CAG and distribution of CAG size (number of genes) was shown in Fig. S7 (Additional files 16). 477 CAGs were assigned to known bacterial genera based on the tracer genes, with at least 80% of the genes mapped to the reference genome at an identity higher than 85%. The CAGs were then compared with the controls yielding 240 CAGs specifically enriched in AF (Additional

files 13: Table S7). A cluster of CAGs containing *Prevotella*, along with anti-inflammatory CAGs such as *Faecalibacterium*, were more abundant in the healthy controls (Additional files 17: Fig. S8). In contrast, the AF-enriched CAGs formed a cluster originated from proinflammatory *Ruminococcus*, *Dorea*, *Eubacterium*, and *Bacteroides*, some microbes enriched in CVD [9, 15, 28].

Based on the clusters of microbial CAG gene markers specific to AF, we aimed to further delineate the features of AF-associated GM and investigate the clinical values of intestinal microbiome for distinguishing AF. Therefore, we performed a random forest disease classifier using the relative abundance of CAGs abundances as variables. With 5, 10, 20, 50, 70, 100 CAG marker variables, the classification error remained low and relatively stable (Additional files 18: Fig. S9, Fig. 3a). According to the box-and-whisker plot for the probability of AF in the cross-validation training set showed that either control or AF group showed a high probability for predicting the true class in training set (n=82) (Fig. 3b). As shown in Fig. 3c, the area under receiver operating curve (AUC) was 97.74% (95% confidence interval (CI) of 95.27 %-100 %) in the training set (n=82), suggesting that subjects suffering from AF could be classified from the controls effectively. Consistently, the AUC for identifying AF from the controls was 98.57 (95% CI, 94.61%–100%) in the testing set (n=18). The CAGs that originated from *Blautia*, *Dorea*, *Eubacterium*, *Prevotella*, *Bacteroides*, *Ruminococcus* and *Lachnospiraceae* contributed the most to discriminating AF from controls (Fig. 3d). These CAGs were significantly correlated with each other. The abundance of bacteria

enriched in controls were inversely correlated with AF group, and cluster together into a complicated network (Additional files 17: Fig. S8). So far, we have constructed a microbiota-dependent discrimination model for AF detection, and thus the values of dysbiotic GM under AF condition should be further emphasized and uncovered.

Aberrant microbial functions in AF populations

The Kyoto Encyclopedia of Genes and Genomes (KEGG) and evolutionary genealogy of genes: Non-supervised Orthologous Groups (EggNOG) databases were utilized in the present study to access the gut microbial gene functions as described previously [45, 46] (Additional files 19-21: Table S10-S12). AF and control groups could be separated clearly from each other by both PCA and NMDS, suggesting significant difference of microbial functions between AF patients and controls ($P<0.001$, Anosim, Fig. 4a, b, d, e). There were thirty-five KEGG modules differentially enriched among the two groups (adjusted P value <0.05, Wilcoxon rank sum test, Fig. 4c), of which, twenty-four modules that decreased in the AF group were implicated in the biosynthesis of fatty acid and aminoacyl-tRNA. Furthermore, genes for iron complex transport system, nucleotide sugar biosynthesis, citrate cycle and glycolysis were also reduced in AF patients. These metabolic functions produce metabolites necessary for maintaining human health and some have been indicated to be deficient in patients with HTN [9], CHF [28] or liver cirrhosis [12]. Eleven KEGG modules such as histidine biosynthesis, putative multiple sugar transport system, heme biosynthesis (glutamate to

protoheme/siroheme) and pentose phosphate pathway were found to be significantly elevated in the AF group. They were also increased in patients with colorectal adenoma-carcinoma [13], rheumatoid arthritis, T2DM, obesity, ACVD and cirrhosis [15]. Moreover, some EggNOG orthologs enriched in the control group participate in maintaining the normal human operations, such as DNA replication, recombination and repair and cell wall/ membrane/ envelope biogenesis. Other identified EggNOG orthologs that are enhanced in AF patients, function in signal transduction mechanisms such as carbohydrate transport and metabolism. Furthermore, we performed correlation analysis between CAGs and KEGG modules and eggNOGs (Additional files 22: Fig. S10). AF deficient CAGs positively correlated with some basic functions necessary for life-sustaining activities such as such as aminoacyl-tRNA biosynthesis and citrate cycle. Considering these findings, the abnormal microbial functions that result from disordered GM composition in AF populations may directly lead to imbalanced in metabolic profiles, resulting in disease development.

Alterations in gut and serum metabolomics in AF

Mammalian metabolism is thought to be greatly influenced by an interaction with the intestinal microflora community. To explore how the host metabolic pattern alterations were impacted by the gut microbiota dysbiosis in AF patients, serum and fecal samples were collected and analyzed by high-throughput liquid chromatography-mass spectrometry (LC/MS) in both positive ion mode (ES+) and negative ion mode (ES−).

A subset of 65 subjects (36 controls and 29 AFs) from the present study were enrolled in the serum metabolic study and 59 (17 controls, and 42 AFs) were enrolled in the feces study (Additional files 23-24, table S13-14). For serum, 2548 features at (ESI+) ion mode and 1733 features at (ESI-) ion mode were detected. And for feces, 2547 features at (ESI+) ion mode and 1894 features at (ESI-) ion mode were tested in this experiment. The partial least-squares discriminant analysis (PLS-DA) and the orthogonal partial least-squares discriminant analysis (OPLS-DA) were plotted to reveal the global metabolic changes between AF and controls. For the fecal samples, a clear separation between AF patients and healthy controls were obtained under both ES+ and ES- modes (Fig. 5 a, b). The serum data recapitulated the distinction, successfully classifying the AF and control groups with PLS-DA and OPLS-DA methods (Fig. 5 c, d).

Significant differentially enriched metabolites were identified based on the variable importance in the projection (VIP) threshold >1 and the p value < 0.05 and were further matched in the Metlin database. Overall, 96 serum metabolites, 46 elevated and 50 decreased, were detected in AF patients as compared to controls (Additional files 25: Fig. S11). For the stool samples, 63 metabolites, 15 increased and 48 down-regulated, differentiated AF patients from healthy controls (Additional files 26: Fig. S12).

Notably, 27 metabolites were altered in both serum and stool samples of AF patients (Fig. 6a, b), 16 of which showed the same variation trend and were the focus

of further investigation (Fig. 6b, Additional files 27: Table S15). These compositional changes identified AF-enriched compounds, such as Chenodeoxycholic acid and LysoPC (15:0). There were 14 metabolites with significantly decreased abundance in AF including cholic acid, oleic acid (OLA), linoleic acid (LA), and α-Linolenic Acid (ALA) (Fig. 6b). Chenodeoxycholic acid was able to activate the NLRP3 inflammasome in macrophages, which could primarily induce IL-1β and aggravates inflammatory process and affected the epithelial integrity by inducing the production of pro-inflammatory cytokines [47]. Cholic acid may influence the cardiac electrophysiology, inhibiting the activity of cardiac myocytes, causing calcium overload and leading to sudden fetal death hence might influence the cardiac electrophysiology [48]. Furthermore, cholic acid was reported could strongly reduce endoplasmic reticulum (ER) stress by inhibiting ERK signaling and ER stress-related transcription factor ATF4 [49]. A 20-year cohort study following more than 74,000 participants revealed that OLA consumption significantly relieved the risk for developing cardiovascular disease (CVD) [50]. OLA prevents coronary heart disease by suppressing oxidative stress, mitigating cardiomyocyte cell damage [51]. Previous observational studies have reported that LA, the predominant n-6 PUFA from vegetable oils and nuts, could reduce major risk factors of ACVD [52]. Increased LA intake is believed to reduce LDL cholesterol, promote insulin sensitivity and attenuate the risk of HTN [53]. These metabolic variations might aggravate or even promote the

arrhythmogenic substrate aggravation in the left atrium during the pathological processes of AF.

Furthermore, some metabolites showed increased tendency in serum but decreased in feces. These pathogenic substances might originate from a pathway other than gut microbes. For example, higher levels of circulating palmitic acid was associated with a higher risk of AF [22]. Circulating succinate, a metabolite produced by both microbiota and the host, was increased in HTN, ischemic heart disease, and type 2 diabetes [54]. Adrenic acid is an inflammation enhancer in non-alcoholic fatty liver disease [23].

To explore the association between aberrant metabolites and disordered gut microflora, we carried out a correlation analysis between the top 10 genera (Fig. 6c) and species (Fig. 6d) enriched in AF or control groups and the 16 representative metabolites in serum or feces with similar variation tendencies. Consistently, LA and ALA, previous described as cardiovascular protectors, were negatively associated with generas such as *Flavonifractor, Hungatella* and species like *Prevotella. copri*. ALA and LA were reported to prevent as well as terminate the lysophosphatidylcholine or acylcarnitine-induced arrhythmias [55]. The close relationship between microbes and metabolites indicate the specific metabolites might be produced at least indirectly by corresponding gut microbe, which remains further investigation.

Based on the significant correlation between the distinguished metabolic features in AF and the disordered gut floras, it was indicated that the gut microbiota dysbiosis

induced disordered microbial functions, causing the deficiency of multiple cardiovascular-protective metabolites and thus increased susceptibility to AF.

Discussion

In the present study we obtained seminal evidence delineating the features of the AF-associated gut dysbiosis through the integration of metagenomic and metabolomic analyses. The AF individuals exhibited significantly elevated richness and increased diversity of gut microbiota and thus the overgrowth of bacteria may be key to the development and establishment of AF. The GM shift from an enterotype represented by *Prevotella* to *Bacteroides* further characterized an imbalanced intestinal microbial environment specific to AF. Gut bacteria such as *Faecalibacterium*, *Alistipes*, *Oscillibacter*, *Bilophila* and *Flavonifractor* declined substantially in the intestinal tract from AF patients. Inversely, *Ruminococcus*, *Streptococcus* and *Enterococcus* were typically enriched in the AF-associated gut metagenomic composition. Metabolic profiles of both fecal and serum samples analyzed from AF patients demonstrated significant alterations, which were correlated with gut microbiota dysbiosis. More importantly, a discriminant model based on bacterial signature profiles has been established and may have the potential to be used as biomarkers for AF in the future. It is therefore hypothesized that an increase of a specific group of gut flora may induce disordered metabolic activity of GM, triggering the accumulation of bacterial metabolites in the circulation. This accumulation, where they could negatively affect

human health perturbing the progression of AF and may even play an important role in the establishment of AF. Intervention strategies targeting gut microbiota to improve the progression of AF are strongly encouraged.

To our knowledge, the richness and diversity of GM has been evaluated in multiple diseases, particularly in CVD, and variable findings were reported recently. In atherosclerotic disease, it was suggested that GM diversity is inversely associated with arterial stiffness in women [56], whereas a higher microbial richness and diversity in the systemic microbiome of ST-segment correlated to elevated myocardial infarction events [57]. The increased diversity of GM was also observed in stroke and transient ischemic attack patients and this dysbiosis was correlated with the severity of the disease [58]. Thus, the evaluated richness and diversity of GM could reflect the imbalanced gut milieu, characterized by overgrowth of a variety of harmful bacteria and fewer commensal or beneficial genera. This is consistent with the present study.

A cluster of bacteria significantly aggregated in the gut from AF patients, including *Ruminococcus*, *Streptococcus* and *Enterococcus*. The accrual of these microorganisms in the intestine may inhibit the growth of some bacteria enriched in healthy populations. For example, the decline of *Faecalibacterium*, *Alistipes*, *Oscillibacter*, *Bilophila* and *Flavonifractor* often occurred in conjunction with changes *Streptococcus* abundance [9, 15, 28]. It is worth noting that AF patients shared the enrichment of numerous microbial flora, such as *Streptococcus*, *Dorea*, *Enterococcus*

and *Coprobacillus*, demonstrated in HTN [9], CHF [28] and ACVD [15]. Additionally, patients with cardiovascular diseases often have decreased levels of *Faecalibacterium* and *Oscillibacter*, which are butyrate-producing species identified as important anti-inflammatory commensal bacterium [36, 59]. *Alistipes*, *Bilophila* and *Butyricicoccus* also exhibited the same decreasing trending AF and other CVDs, like HTN [9], CHF [28] and ACVD [15]. This group of bacterial strains is consistently altered in multiple cardiovascular diseases and is therefore considered a guild emerging during the progression of disease. The chronic cardiovascular diseases mentioned above might be a consequence of the imbalanced gut microbial composition associated with the establishment of this guild. Although the underlying mechanism remains largely unknown, several CVDs share some common pathophysiologic pathways, such as endothelial dysfunction [60]. Reestablishing the functionally active ecological populations as the primary ecosystem service providers is crucial to a healthier gut microbiota. Restoring the deficient gut microbe might alleviate or attenuate the disease phenotypes or progression. Targeted promotion of the gut ecosystem by individualized intertion may present a novel ecological approach for manipulating the gut microbiota to manage CVD and potentially other dysbiosis-related diseases [61].

Notably, GM of AF exhibited some unique features not displayed in other related diseases. For example, *prevotella*, whose function is to encode superoxide reductase, phosphoadenosine phosphosulphate reductase and favor the development of inflammation [62], showed a declined trend in AF, but overgrowth in HTN [9]. In

addition, some flora decreased in HTN [9] exhibited a tendency to be increased in AF, CHF [28] and ACVD [15], such as *Ruminococcus, Enterococcus, Veillonella, Coprococcus* etc. These seemingly paradoxical phenomena may partly be explained by the complex and various factors involved in the pathophysiological process. To a certain extent, the generality and specificity of cardiovascular diseases could be analyzed from the point of view of gut flora.

Metabolites derived by the gut microbiota, such as TMAO, have been confirmed to act on downstream cellular targets to improve or contribute to the pathogenesis of structural, metabolic and functional cardiovascular remodeling [63]. Here, our present study revealed decreased levels of LA and ALA in AF patients, which was consistent with the decreased function of GM in fatty acid biosynthesis. Notably, ALA/LA exerted protective effects through inhibition of reactive oxygen species generation, down regulation of the activation of the p38 mitogen-activated protein kinases (MAPKs) pathway and the expression of transforming growth factor β1 (TGF-β1), which played the regulatory role of atrial fibrosis and contributed to the progression of AF [53]. Taken together, these findings highlight the potential and diverse physiological effects of GM-related metabolites during the progression of AF. Further studies are required to make clear the biological mechanism underlying these differential effects.

Promisingly, the microbiota-dependent discrimination model we built could distinguish AF from controls nicely based on the GM feature. Traditionally, AF can be

further distinguished as paroxysmal (PAF) and persistent (PeAF) AF based on the presentation, duration and spontaneous termination of AF episodes. The episodic pattern of PAF is self-terminating, in most cases within 48 hours, while peAF is characterized as lasting longer than 7 days, including episodes that are terminated by cardioversion, with drugs or by direct current cardioversion after 7 days or more [64]. Among our present AF cohort, there were 30 PAF patients and 20 peAF patients. The types of AF may be partially determined by the varying extent of personalized electrical and structural remodeling in atrial arrhythmogenic substrate. Additionally, they have different prognoses and responses to rhythm-controlling therapy and distinction between the types helps the physician and patient to make individualized therapeutic decisions [65]. Therefore, the classification of AF type based on the characteristics of gut microbiota might have more significant clinical value, which will be explored in our future work.

Consideration of possible confounders and limitations are of relevance to our study and help to inform the design of future studies. Some of the AF patients recruited in our cohort were also diagnosed with HTN or T2DM. Isolated AF, driven by genetic factors, represents a minority of AF cases and the pathogenesis of AF may be an end stage of multiple metabolic and cardiovascular diseases [7, 8]. To reflect the real signature of clinical practice we did not exclude patients with comorbidities even though HTN and T2DM have been widely known to be connected with GM dysfunction. To evaluate the disordered patterns of GM resulting solely from AF, the HTN history in each group

was matched individually to remove the HTN contribution. Separately, there were 12 AF patients with T2DM which was not adjusted between groups. We performed PCA plot to assess the contribution of different baseline characteristics and found that PCA failed to distinguished AF patients into separated group based on these factors, indicating the negligible impact of gender, age, TC or T2DM on our data. Therefore, the majority effect of GM dysbiosis observed in the AF-associated cohort was not mediated by HTN or T2DM. Secondly, although we excluded subjects who used antibiotics or probiotics and confirmed the possible influences of drug consumption (DMBG and statins) on gut microbiota, exercise and dietary information were not collected and corrected in this study. Thirdly, the conclusions drawn from our data were associations rather than causal relationships. Further studies such as gut microbiota transplantation and electrophysiological modulation testing AF inducibility are still needed. The present results provided preliminary clues and evidence for future investigations regarding the potential mechanisms between gut microbes and AF.

Conclusions

The present study provides the first comprehensive description of the disordered patterns of gut microbiota and aberrant microbial-related metabolites in a cohort of AF patients. These novel findings are fundamental for further studies exploring the causal relationship between AF and GM, but they are just the beginning. An extensive amount

of research is still needed to explore the clinical values of intervention strategies based on gut microbiota to improve AF conditions.

Methods

Study cohort

50 patients with nonvalvular AF were consecutively enrolled from Beijing Chaoyang Hospital and 50 individuals as matched controls were enrolled from Kailuan cohort who received biennial medical examination in Kailuan General Hospital [66]. Individuals with a history of heart failure, coronary heart disease, structural heart disease, comorbidities (inflammatory bowel diseases, irritable bowel syndrome, autoimmune diseases, liver diseases, renal diseases or cancer) or use of antibiotics or probiotics in the last 1 month were excluded. Demographic and clinical characteristics were obtained by completing face-to-face surveys and checking hospital or medical examination records. 50 samples from our previous work [9] regarding gut microbiota were selected by matching for the history of hypertension and the metagenomic sequencing data of 50 control stool samples from our previous study were used as controls in the present study. Among the 50 AF patients included, fecal samples were available from each subject and used for metagenomic analyses. Metabolomic analyses were performed using serum samples from 8 AF patients and 12 controls and stool samples from 8 AF patients and 8 controls. The study conforms well to the principles from the Declaration of Helsinki. The research protocol was approved by the ethics

committee of Beijing Chaoyang Hospital and Kailuan General Hospital. All of the participants signed informed consents.

Stool sample collection and DNA extraction

Fresh stool samples were collected from each participant, immediately frozen at −20 °C, transported on ice to the laboratory and then stored at −80 °C. Bacterial DNA was extracted using TIANGEN kit from Novogene Bioinformatics Technology Co., Ltd.

Metagenomic sequencing, gene catalogue construction

Paired-end metagenomic sequencing was sequenced on the Illumina platform (insert size 300 bp, read length 150 bp) at the Novogene Bioinformatics Technology Co., Ltd. After quality control, the reads aligned to the human genome (alignment with SOAP2, Version 2.21, parameters: -s 135, -l 30, -v 7,-m 200,-x 400, RRID: SCR_005503) were removed and the remaining high-quality reads were used for further analysis. The assembly of reads was executed using SOAP denovo (Version 2.04, parameters: -d 1 -M 3 -R -u -F. RRID: SCR_010752). For each sample, we used a series of k-mer values (from 49 to 87) and chose the optimal one with the longest N50 value for the remaining scaffolds [12]. The clean data was mapped against scaffolds using SOAP2 (Version 2.21, parameters: -m 200 -x 400 -s 119, RRID: SCR_005503). Unused reads from each sample were assembled using the same parameters.

Gene prediction from the assembled contigs was performed using Meta GeneMark (prokaryotic GeneMark. hmm version 2.10). A non-redundant gene catalogue was constructed with CD-HIT (version 4.5.8, parameters: -G 0 -aS 0.9 -g 1 -d 0 -c 0.95. RRID: SCR_007105) using a sequence identity cut-off of 0.95, with a minimum coverage cut-off of 0.9 for the shorter sequences. Reads were realigned to the gene catalogue with SOAP2 using parameters to determine the abundance of genes: -m 200 -x 400 -s 119. Only genes with ≥2 mapped reads were decided included. The gene abundance was calculated by counting the number of reads and normalizing by gene length.

Analyses of genera richness and enterotypes

Rarefaction analysis was carried out to evaluate gene richness. Using R (Version 2.15.3, vegan package), the cohort was randomly sampled 100 times with replacement and the total number of identified genes from these samples was assessed.

Based on the genera profiles, we calculated the within-sample (α) diversity using the Shannon index to estimate the genera richness of the sample. A high α diversity denotes a high richness of genera within the sample.

By using the PAM method based on relative abundance of genera, we analyzed the community types of each sample. As previously described [67], we estimated the optimal number of clusters using the CH index. Genera with an average relative abundance ≥10−4 and present in at least six samples would be used in the analysis. The

genera in enterotype 1 were clustered according to the Spearman's correlation between genera abundances and their co-occurrence network was visualized using Cytoscape (Version 3.2.1; RRID：SCR_003032).

Taxonomic assignment, annotation and abundance profiling

Genes were aligned to the integrated NR database to assess the taxonomic assignment by using DIAMOND (Version 0.7.9.58, default parameter except that -k 50 -sensitive -e 0.00001; RRID: SCR_016071). To distinguish taxonomic groups, the significant matches for each gene, defined by e-values ≤10 × e-value of the top hit, were determined and the retained matches were used as previously described [68]. The taxonomical level of each gene was determined using the lowest common ancestor-based algorithm implemented with MEGAN (RRID: SCR_011942). The abundance of a taxonomic group was calculated by summing the abundance of genes annotated to a feature.

Co-abundance gene groups (CAGs) and CAGs network of marker CAGs

As previously described [69, 70], we compared the abundance of each gene across groups to identify the marker genes associated with AF. Based on their abundance variation across groups these marker genes were clustered into groups [34]. Co-abundance gene groups (CAGs) were defined as clusters with more than 50 genes [9], [12], [70]. CAG abundance profiles were calculated based on the average gene depth signal and weighted by gene length. Taxonomic assignment of the CAGs was

performed based on the taxonomy of tracer genes, as previously described [9, 10]. All genes from one CAG were aligned to the reference microbial genomes at the nucleotide level (by BLASTN) and the NCBI-nr database at the protein level (by BLASTP). The alignment hits were filtered by both thee-value (< 1×10-5at the nucleotide level and < 1×10-5at the protein level) and the alignment coverage (>70% of a query sequence). From the alignments with the reference microbial genomes, we obtained a list of well-mapped bacterial genomes for each CAG and ordered these bacterial genomes according to the proportion of genes that could be mapped onto the bacterial genome, as well as the average identity of the alignments. The species assignment required 90% of the genes in a CAG to match with the species' genome with 95% identity and 70% overlap of query. The CAG assignment to a genus required 80% of its genes to align to the genome with 85% identity in both DNA and protein sequences.

The enriched CAGs were identified and clustered according to Spearman's correlation and the co-occurrence network was visualized by Cytoscape (Version 3.2.1; RRID：SCR_003032). Based on the abundance in the set of compared samples, an odds ratio (OR) score [69] was calculated for each CAG and for the comparative analysis between control and AF samples; the AF-associated CAGs were identified as AF-enriched (OR >2) or AF-depleted (OR <0.5).

Functional annotation

Using DIAMOND (Version 0.7.9.58, default parameter except that -k 50 –sensitive -e 0.00001), all genes in catalogue were aligned to the KEGG database (Release 73.1, with animal and plant genes removed) and to the eggNOG database (v4.5 via eggNOG-mapper with HMM search mode). Each protein was assigned to the KEGG and eggnog orthologs using the highest scoring annotated hits containing at least one HSP scoring over 60 bits. By summing the abundance of genes annotated to the same feature, the abundance of KEGG orthologue/module was calculated.

Metabolomic analysis based on LC/MS

50 mg fecal samples were pipetted into centrifuge tubes (1.5 mL) in preparation for extraction. The protein was precipitated with 800 μL of methanol and 10 μL of internal standard (2.9 mg/mL, DL-o-Chlorophenylalanine) was added. The samples were ground at 65 KHz for 90 s and centrifuged at 12000 rpm for 15 min at 4 °C. 200 μL of the supernatant was transferred into a vial for further analysis. The serum samples were thawed at room temperature and 100 μL was pipetted into centrifuge tubes (1.5 mL) in preparation for extraction. The protein was precipitated with 300 μL of methanol, and 10 μL of internal standard (2.9 mg/mL, DL-o-Chlorophenylalanine) was added. The samples were vortexed for 30 s and centrifuged at 12000 rpm for 15 minat 4 °C. 200 μL of the supernatant was transferred to a vial for further analysis. The fecal and serum metabolic profiles were performed on a LC/MS platform (Thermo, Ultimate 3000LC, Orbitrap Elite) using a Hypergod C18 (100 × 4.6 mm 3 μm) column. The

chromatographic separation conditions were as follows: the column temperature, 40 °C; flow rate, 0.3 mL/min; mobile phase A, water +0.1% formic acid; mobile phase B, acetonitrile +0.1% formic acid; injection volume, 4 ml; automatic injector temperature, 4 °C.

For both fecal and serum samples the following conditions were used for the positive ion mode (ES+): heater temp, 300 °C; sheath gas flow rate, 45arb; aux gas flow rate, 15arb; sweep gas flow rate, 1arb; spray voltage, 3.0KV; capillary temp, 350 °C; S-lens RF level, 30%. The following conditions were used for negative ion mode (ES−): Heater temp, 300 °C; sheath gas flow rate, 45arb; aux gas flow rate, 15arb; sweep gas flow rate, 1arb; spray voltage, 3.2KV; capillary temp, 350 °C; S-lens RF level, 60%.

All metabolomic data were prepared for feature extraction and preprocessed with Compound Discoverer 2.0 software (Thermo). Data were normalized at the start. Considering the remarkable differences existed among various metabolites, some signals of metabolites with too high or low concentration might be covered up and failed to be identified as biomarkers. So, normalization, aiming to adjust the weight of different variables to decrease the gap of different signals, should be performed to make the dimension (for example mean and standard deviation) of all variables in a similar level and make the data more comparable. The calculation process was to normalize the peak area of each sample to 1000000 and divide the peak area of each ion by the total peak area of the sample and multiplied by 1000000. Data were then edited into a

two-dimensional data matrix by excel 2010 software, using Retention time (RT), Compound Molecular Weight (compMW), Observations (samples) and peak areas. Using SIMCA-P software (Umetrics AB, Umea, Sweden), a multivariate Analysis (MVA) was performed. Compounds were significantly distinguished between groups, identified by a variable influence on projection (VIP) > 1and p value < 0.05 based on the peak areas. The exact molecular mass, ppm (<25) and ms/ms value of these compounds was used to identify the metabolites related to the featured peak in the Metlin database (http://metlin.scripps.edu). Furthermore, we will compare the mass compactum. The score value indicated the matching rate was calculated by Compound Discoverer 2.0 software (Thermo) with max of 100. For metabolites detected in both ES+ and ES−, the data in the mode with the lower p value was retained for further analysis.

Statistical analysis

Quantitative demographic and clinical characteristic data with normal distributions were presented as mean ± standard deviation and the t-test was used for between group comparisons. Quantitative data with non-normal distributions were presented as median (first quartile, third quartile) and the Wilcoxon rank sum test was performed for between group comparisons. Qualitative data were presented as a percentage and the Chi-square test was used for between group comparisons. All statistical tests were 2-sided and $p < 0.05$ was regarded as significant. Statistical analyses were performed with

the SPSS version 22.0 (IBM Corp., Armonk, New York)

The Shannon index at the genera level was calculated with QIIME (Version 1.7.0. RRID: SCR_008249). PCA was performed using the Facto MineR package in R software (Version 2.15.3) while PCoA was performed by using ade4 package, cluster packages, fpc packages, and cluster Sim package in R software (Version 2.15.3). PLS-SEM analysis was conducted using the Smart-PLS 3 software. PLS-DA was carried out using the SIMCA-P software to cluster sample plots across groups.

Differential abundance of genes, genera and KO modules was tested based on the Wilcoxon rank sum test and P values were corrected for multiple testing with the Benjamin & Hochberg method. Genera with an average relative abundance ≥10−4 and presence in at least six subjects were included in the analyses.

Based on the profiles of CAGs, the samples were randomly divided into training and test sets. A random forest classifier was trained on 80% of the data and tested on the remaining 20% of our data using the random forest package in R. We performed a 10-fold cross-validation within the training set to evaluate the performance of the predictive model and obtain more precise curves. The cross-validation error curves (average of 10 test sets each) from five trials of the 10-fold cross-validation were averaged. Variable importance was calculated for the random forest models using the full set of features determined by mean decrease in accuracy. At the lowest cross-validation error, the number of variables was 1000. Therefore, the predictive model was

constructed using the 1000 most important variables, and the performance was assessed using ROC analysis. The 95% CIs for the ROC curves were calculated using the pROC R package. The performance of the smaller models was measured as the AUC when applied to the test set.

Data Availability

The data supporting the results of this article has been deposited in the EMBL European Nucleotide Archive (ENA) under the BioProject accession code PRJEB28384. The metabolomics data is available at the NIH Common Fund's Data Repository and Coordinating Center website with Metabolomics Workbench Study ID: ST001168 (for fecal metabolomic analyses) and ST001169 (for serum metabolomic analyses). Other data further supporting this work is available in the *GigaScience* repository, GigaDB [71].

Abbreviations: AF, atrial fibrillation; BMI, body mass index; HTN, hypertension; DM, diabetes mellitus; CHD, coronary heart disease; TC, total cholesterol; TG, triglyceride; LDL, low density lipoprotein; FBG, fasting blood glucose; UA, uric acid; TBil, total bilirubin; ALT, glutamic-pyruvic transaminase; ACEI, angiotensin converting enzyme inhibitors; ARB, angiotensin receptor blockers; DMBG, dimethyl biguanide; OAC, oral anticoagulation therapy. IQR, interquartile range; Data are presented as mean± SD, or median (IQR), as appropriate.

Ethics approval and consent to participate

The research protocol was approved by the ethics committee of Beijing Chaoyang Hospital and Kailuan General Hospital. All of the participants signed informed consents.

Competing interests: The authors that they have no competing interests.

Funding

This work was supported by the National Natural Science Foundation of China (81500383, 81670214, 81870308, 81770253, 81370362), the Beijing Natural Science Foundation (7172080), the Beijing Municipal Administration of Hospitals' Youth Programme (QML20170303), and the 1315 personnel training plan (CYMY-2017-03).

Author contributions

XCY, JL and KZ conceived the study, directed the project, designed the experiments, interpreted the results, and wrote the manuscript. YFG, MLC, RMH, YL, HJC, and HJW recruited, diagnosed, and collected the clinical details from the subjects. SCL and XYL collected the blood and feces samples from the subjects. KZ, JL, JC and KBL analyzed the data. XCY, JL, YQW, HCW, JC and JCZ revised the manuscript. All authors read and approved the final manuscript.

Acknowledgements

Not applicable.

Table1. Baseline clinical characteristics of the study cohort.

	AF Group	Control Group	P value
**Number**	50	50	/
**Age, years**	66 (57, 71.25)	55 (50.5, 57.5)	<0.001
**Male/ Female**	32/18	41/9	0.043
**BMI**	26.46 (23.79, 28.64)	24.77 (22.79, 27.62)	0.112
**HTN**	27	27	/
**DM**	12	0	/
**TC**	4.13±1.05	4.82±0.96	0.001
**TG**	1.29 (1.02, 1.88)	1.06 (0.77, 1.80)	0.084
**LDL**	2.45 (1.58, 2.93)	2.3 (1.96, 2.86)	0.872
**FBG**	4.95 (4.50, 5.83)	5.12 (4.56, 5.55)	0.883
**Creatinine**	68.5 (60.48, 79.35)	70 (60, 89.5)	0.533
**UA**	321.5 (278, 389.75)	333 (264.5, 384)	0.927
**TBil**	14 (10.08, 19.5)	14.7 (11.59, 19.75)	0.431
**ALT**	19 (13.75, 28.5)	19 (12, 25)	0.185
**ACEI**	7	0	/
**ARB**	4	0	/
**β receptor blockers**	8	0	/
**statins**	4	0	/
**aspirin**	2	0	/
**amiodarone**	10	0	/
**DMBG**	6	0	/
**OAC**	13	0	0

Additional files

Additional files 1: Table S1, Data production of 100 samples in control and AF.

Additional files 2: Fig. S1, Increased Firmicutes/Bacteroidetes ratio, Pielou evenness and Chao richness in AF.

Additional files 3: Fig. S2, Another 12 genera significantly enriched in enterotype 1.

Additional files 4: Fig. S3, Enterotype analysis at the species level.

Additional files 5: Fig. S4, Taxonomic annotation and abundance profiling.

Additional files 6: Table S2, Relative abundance profile at the phylum level.

Additional files 7: Table S3, Relative abundance profile at the genus level.

Additional files 8: Table S4, Detailed information of differential genera.

Additional files 9: Fig. S5, Species strikingly different across groups.

Additional files 10: Fig. S6, Influents of baseline characteristics, including age, gender, T2DM, TC and medication on GM.

Additional files 11: Table S5, Detailed information for 121145 gene markers.

Additional files 12: Table S6, Reference genomes for CAG's taxonomy assignment.

Additional files 13: Table S7, Detailed information of enriched CAGs in different groups.

Additional files 14: Table S8, Detailed information of 477 CAGs.

Additional files 15: Table S9, Spearman’s correlation between enriched CAGs.

Additional files 16: Fig. S7, Size distribution and taxonomic assignment of CAGs.

Additional files 17: Fig. S8, The network of CAGs enriched in AF compared with controls.

Additional files 18: Fig. S9, Gut CAGs (variables in 5, 10, 20, 50, 70) classify AF from controls.

Additional files 19: Table S10, Detailed information of differential KEGG modules.

Additional files 20: Table S11, Detailed information of differential KEGG orthologues.

Additional files 21: Table S12, Detailed information of differential eggNOG family.

Additional files 22: Fig. S10, Correlation between CAGs and altered function module.

Additional files 23: Table S13, Clinical characteristics of participants in serum metabolism.

Additional files 24: Table S14, Clinical characteristics of participants in fecal metabolism.

Additional files 25: Fig. S11, Metabolites differentially enriched in AF and controls in serum.

Additional files 26: Fig. S12, Metabolites differentially enriched in AF and controls in feces.

Additional files 27: Table S15, Detailed information of 16 metabolites differently enriched across groups.

Additional files 28: The computational code of step by step for bioinformatic analysis.

Figure Legends

Figure 1. Elevated microbiota richness and altered community types in AF patients.

a. Rarefaction curves for gene number which were calculated after 50 random sampling with replacement in control (n = 50) and AF (n = 50). X-axis is the number of genes and Y-axis means number of genes. The blue box represents CTR and the red box denotes AF. The rarefaction curve is near smooth when the sequencing data are great enough with few new genes undetected and the present sample size has met the need of this study.

b, c. Gene count (b) and a-diversity (Shannon index) (c) based on the genera profile in the AF and CTR cohorts. Boxes represent the inter quartile ranges, lines inside the boxes denote medians and circles are outliers. Gut microbial richness (gene count), diversity in AF group were much higher (p=0.007, CTR vs AF; for gene count. p=3.53e-05, CTR vs AF; for α diversity; Kruskal-Wallis test.)

d. 100 samples (AFs in shape of circle and controls in block) are clustered into enterotype 1 (green) and enterotype 2 (orange) by principal component analysis (PCA) of Jensen-Shannon divergence values at the genus level. The major contributor in the two enterotypes is *Bacteroides* and *Prevotella*, respectively.

e, f. Relative abundances of the top genera in each enterotype, *Bacteroides* in enterotype 1 (e), *Prevotella* in enterotype 2 (f). Boxes represent the inter quartile ranges, lines

inside the boxes denote medians and circles are outliers. p = 1.730774e-09 and p = 4.376078e-14, respectively; Wilcoxon rank sum test.

g. The percentage of control and AF samples distributed in enterotype1 and enterotype 2. A dysbiosis of enterotype distribution by AF conditions was revealed. 50% CTRs in enterotype1, 50% CTRs in enterotype2. 82% AFs in enterotype1, 18% AFs in enterotype2. p = 0.001, CTR vs AF; Fisher's exact test.

Figure S1. Increased Firmicutes/Bacteroidetes ratio, Pielou evenness and Chao richness in AF.

a. Firmicutes/Bacteroidetes ratio based on the phylum profile in the AF and CTR cohorts. The blue box represents CTR and the red box denotes AF. Boxes represent the inter quartile ranges, lines inside the boxes denote medians and circles are outliers. Firmicutes/Bacteroidetes was higher in AFs (p=7.162e-05, CTR vs AF; Kruskal-Wallis test.)

b, c. Chao richness (b) and Pielou evenness (c) based on the genera profile in the AF and CTR cohorts. The blue box represents CTR and the red box denotes AF. Boxes represent the inter quartile ranges, lines inside the boxes denote medians and circles are outliers. Consistently, GM richness and evenness in AF group were much higher (p=0.007633, CTR vs AF; for Chao richness. p=4.262e-06, CTR vs AF; for Pielou evenness; Kruskal-Wallis test.)

Figure S2. Another 12 genera significantly enriched in enterotype 1.

The relative abundances of another 12 genera enriched enterotype 1. X-axis is the top 12 genera enriched in enterotype 1 and Y-axis denotes relative abundance of them. The green box represents enterotype 1 and the orange box denotes enterotype 2. Boxes represent the inter quartile ranges, lines inside the boxes denote medians and circles are outliers and q (adjust *p*) value was shown in the top of box; Wilcoxon rank sum test.

Figure S3. Enterotype analysis at the species level.

a**.** 100 samples (AFs in shape of circle and controls in block) are clustered into enterotype 1 (blue) and enterotype 2 (brown) by principal component analysis (PCA) of Jensen-Shannon divergence values at the species level.

b. The percentage of control and AF samples distributed in enterotype 1 and enterotype 2 at the species level. A dysbiosis of enterotype distribution by AF conditions was revealed consistently. 57.14% CTRs in enterotype 1, 42.86% CTRs in enterotype 2. 80.43% AFs in enterotype 1, 19.57% AFs in enterotype 2. $p = 0.01662$, CTR vs AF; Fisher's exact test.

Figure S4. Taxonomic annotation and abundance profiling at the genus level.

a, b. PCA (a) and non-metric dimensional scaling (NMDS) (b) plot based on abundances of the microbes showed the structures of gut microbiota in AF was

discriminative from controls. The blue block represents CTR and the red circle denotes AF.

c. Heatmap of top 35 genera enriched across controls and AF patients. The relative abundance profiles were transformed into Z scores by subtracting the average abundance and dividing the standard deviation of all samples. Z score is negative (shown in blue) when the row abundance is lower than the mean, and red when the row abundance is higher than the mean.

Figure 2. Genera strikingly different across groups.

a. PCA based on abundances of the microbes showed the structures of gut microbiota in AF were significantly different from controls at the genus level. The blue block represents CTR and the red circle denotes AF.

b. Relative abundance of the top 35 most different genera across groups at the criteria of q value <0.05 and q value was presented in the square brackets; Wilcoxon rank sum test. The abundance profiles are transformed into Z scores by subtracting the average abundance and dividing the standard deviation of all samples. Z score is negative (shown in blue) when the row abundance is lower than the mean, and red when the row abundance is higher than the mean.

c. The box plot shows the relative abundance of top 10 genera enriched in controls and AFs. The blue box represents CTR and the red box denotes AF. Genera are colored

according to the phylum, boxes represent the inter quartile ranges, lines inside the boxes denote medians and circles are outliers and q (adjust P) value is shown in the top of box; Wilcoxon rank sum test.

Figure S5. Species strikingly different across groups.

a. PCA based on abundances of the microbes showed the structures of gut microbiota in AF were significantly different from controls at the species level. The blue block represents CTR and the red circle denotes AF.

b. Relative abundance of the top 35 most different species across groups at the criteria of *q* value <0.05 and q value was presented in the square brackets; Wilcoxon rank sum test. The abundance profiles are transformed into Z scores by subtracting the average abundance and dividing the standard deviation of all samples. Z score is negative (shown in blue) when the row abundance is lower than the mean, and red when the row abundance is higher than the mean.

c. The box plot showed the relative abundance of top 10 species enriched in controls and AF patients. The blue box represents CTR and the red box denotes AF. Species are colored according to the phylum, boxes represent the inter quartile ranges, lines inside the boxes denote medians and circles are outliers and q (adjust *p*) value is shown in the top of box; Wilcoxon rank sum test.

Figure S6. Influents of baseline characteristics, including age, gender, T2DM, TC and medication on GM.

a. PCA plot based on age and abundances of the microbes at the genus level. 100 samples were divided into three grades according to age, <55 (color in yellow), 55-65 (color in light pink), and >65 (color in violet). The block represents CTR and the circle denotes AF.

b. PCA plot based on gender and abundances of the microbes at the genus level. 100 samples were divided into 2 groups according to gender, female (color in dark pink), and male (color in dark blue). The block represents CTR and the circle denotes AF.

c. PCA plot based on T2DM and abundances of the microbes at the genus level. 100 samples were divided into 2 groups according to T2DM history, without T2DM (color in grey), with T2DM (color in dark purple). The block represents CTR and the circle denotes AF.

d. PCA plot based on TC and abundances of the microbes at the genus level. 100 samples were divided into 2 groups according to their TC level, without TC<5.18 (color in green), TC≥5.18 (color in pink). The block represents CTR and the circle denotes AF.

e. PCA plot based on medication and abundances of the microbes at the genus level. 50 AF samples were divided into 3 groups according to their medication, circle colored in

light red denotes subjects receiving DMBG therapy, blue block means subjects with statin therapy, and triangle colored in green denotes subjects without DMBG or statin therapy.

Figure S7. Size distribution and taxonomic assignment of CAGs.

a. The 121,145 genes significantly different across control and AF group were clustered into linked gene groups, and the distribution of gene number within these clusters were shown in the histogram. Clusters with a gene number higher than 50 were defined as CAG.

b. Characterization of taxonomic assignment for CAGs based on the genes. The size of points denoted the gene number within the CAG, and the color of points indicated different phylum. The X-axis (coverage) represented the percentage of genes in the CAGs annotated to known bacterial phylum, and the Y-axis was the identity of genes to align with a genome in both DNA and protein sequences according to BLAST.

Figure S8. The network of CAGs enriched in AF compared with controls.

CAGs are colored according to the taxonomic assignment as labeled, and the node size is scaled with the number of genes within the CAG. Edges between nodes denote Spearman correlation positive (blue) or negative (red). Positive intra-group correlation and negative inter-group correlation was shown.

Figure 3. Gut CAGs classify AF from controls.

a. The random forest disease classifier. The model was trained using relative abundance of the CAGs in the controls and AF samples as variables. In training set (n=82), distribution of 5 trials of 10-fold cross-validation (CV) error in random forest classification of AF as the number of CAGs increased. The red line marked the number of CAGs in the optimal set with the lowest cross-validation error.

b. Box-and-whisker plot for the probability of AF in the cross-validation training set according to the model in a. Either control or AF group showed a high probability for predicting the true class in training set (n=82).

c. Receiver operating curve (ROC) for the training set (n=82). The area under receiver operating curve (AUC) is 97.74% and the green area indicated 95% CI: 95.27%-100%.

d. The top 30 different CAGs distinguish AF from control based on the random forest model using explanatory variables of CAGs.

e. ROC for the test set (n=18). The AUC is 98.57% and the green area indicated 95% CI: 94.61%-100%.

Figure S9. Gut CAGs (variables in 5, 10, 20, 50, 70) classify AF from controls.

(a-d, variable=5); e-h, variable=10; i-l, variable=20; m-p, variable=50; q-t, variable=70.)

a, e, i, m, q. Distribution of 5 trials of 10-fold cross-validation error in random forest classification of AF as the number of CAGs increases. With 5 (a), 10 (e), 20 (i), 50(m), 70 (q) CAG marker variables, the classification error remained low and relatively stable.

The red line marked the number of CAGs in the optimal set with the lowest cross-validation error.

b, f, j, n, r. With 5 (b), 10 (f), 20 (j), 50(n), 70 (r) CAG marker variables, box-and-whisker plot for the probability of AF in the cross-validation training set. Either control or AF group showed a high probability for predicting the true class in training set (n=82).

c. With 5 CAG marker variables, receiver operating curve (ROC) for the training set (n=82). The area under receiver operating curve (AUC) is 95.36% and the green area indicated 95% CI: 91.54%-99.18%.

d. With 5 CAG marker variables, ROC for the test set (n=18). The AUC is 94.29% and the green area indicated 95% CI: 83.93%-100%.

g. ROC for the training set (n=82) with 10 CAG marker variables. The area under receiver operating curve (AUC) is 95.48% and the green area indicated 95% CI: 91.67%-99.28%.

h. ROC for the test set (n=18) with 10 CAG marker variables. The AUC is 97.14% and the green area indicated 95% CI: 90.48%-100%.

k. ROC for the training set (n=82) with 20 CAG marker variables. The area under receiver operating curve (AUC) is 96.31% and the green area indicated 95% CI: 92.98%-99.63%.

l. ROC for the test set (n=18) with 20 CAG marker variables. The AUC is 97.14% and the green area indicated 95% CI: 90.48%-100%.

o. ROC for the training set (n=82) with 50 CAG marker variables. The area under receiver operating curve (AUC) is 97.02% and the green area indicated 95% CI: 94.16%-99.89%.

p. ROC for the test set (n=18) with 50 CAG marker variables. The AUC is 98.57% and the green area indicated 95% CI: 94.61%-100%.

s. ROC for the training set (n=82) with 70 CAG marker variables. The area under receiver operating curve (AUC) is 97.56% and the green area indicated 95% CI: 95.02%-100%.

t. ROC for the test set (n=18) with 70 CAG marker variables. The AUC is 98.57% and the green area indicated 95% CI: 94.61%-100%.

Figure 4. Microbial gene functions annotation in AF.

a, b. PCA (a) and NMDS (b) based on the relative abundance of KEGG orthology groups in 100 samples showed significant difference between AF and CTR. The blue block represents CTR and the red circle denotes AF.

c. The average abundance of KEGG modules differentially enriched in control and AF gut microbiome. The relative abundance profiles were transformed into Z scores by

subtracting the average abundance and dividing the standard deviation of all samples. Z score is negative (shown in blue) when the row abundance is lower than the mean, and red when the row abundance is higher than the mean. Overall, 24 modules enriched in control, and 11 modules overrepresented in AF are shown in green and pink, respectively. The physiological effect of KEGG modules and q value are demonstrated on the right; Wilcoxon rank sum test.

d, e. PCA (d) and NMDS (e) based on the relative abundance of eggNOG orthologues in 100 samples showed significant difference between AF and CTR either. The blue block represents CTR and the red circle denotes AF.

f. The average abundance of eggnog orthologues differentially enriched in control and AF. The relative abundance profiles were transformed into Z scores by subtracting the average abundance and dividing the standard deviation of all samples. Z score is negative (shown in blue) when the row abundance is lower than the mean, and red when the row abundance is higher than the mean. Overall, 15 eggNOGs enriched in control, and 20 eggNOGs overrepresented in AF are shown in green and pink, respectively. The potential function of eggNOGs and q value are demonstrated on the right; Wilcoxon rank sum test.

Figure S10. Correlation between CAGs and altered function module.

Spearman's correlation analysis between top 10 significant different CAGs and the top 10 altered KEGG module (a) or eggNOG orthologues (b) in AF; Red, negative correlation; blue, positive correlation, *$p < 0.05$, +$p < 0.01$. The enriched type of each CAGs and functional module was colored according to its direction of enrichment. Green, enriched in controls; pink, enriched in AF patients.

Figure 5. Distinguished metabolic patterns between AF and control.

a. Partial least squares-discriminant analysis (PLS-DA) score plots based on the metabolic profiles in feces samples from control and AF group in ES+ and ES−. The blue block represents CTR and the red circle denotes AF. A clear separation between AF patients and healthy controls were obtained under both ES+ and ES- modes.

b. Score scatter plots of orthogonal PLS-DA (OPLS-SA) comparing the feces metabolic differences identify the separation between AF and control in ES+ and ES−. The blue block represents CTR and the red circle denotes AF.

c. PLS-DA score plots based on the metabolic profiles in serum samples from control and AF group in ES+ and ES−, which successfully classifying the AF and control group. The blue block represents CTR and the red circle denotes AF.

d. Score scatter plots of OPLS-DA comparing the serum metabolic differences identify the separation between AF and control in ES+ and ES−. The blue block represents CTR and the red circle denotes AF.

Figure S11. Metabolites differentially enriched in AF and controls in serum.

The average serum abundance of 97 endogenous compounds differentially enriched in control and AF at VIP >1.0 and P value (t test) <0.05. The relative abundance profiles were transformed into Z scores by subtracting the average abundance and dividing the standard deviation of all samples. Z score is negative (shown in blue) when the row abundance is lower than the mean, and red when the row abundance is higher than the mean. *P* value are demonstrated on the right; t- test.

Figure S12. Metabolites differentially enriched in AF and controls in feces.

The average fecal abundance of 64 endogenous compounds differentially enriched in control and AF at VIP >1.0 and P value (t test) <0.05. The relative abundance profiles were transformed into Z scores by subtracting the average abundance and dividing the standard deviation of all samples. Z score is negative (shown in blue) when the row abundance is lower than the mean, and red when the row abundance is higher than the mean. *P* value are demonstrated on the right; t- test.

Figure 6. Aberrant metabolic patterns related to AF

a. Venn diagrams demonstrated the number of altered metabolites shared between serum (purple) and feces (yellow). The overlap showed that there were 27 endogenous compounds concurrently identified in both feces and serum.

b. The heatmap of fold change (AF/CTR) of 27 compounds which altered in both serum and stool samples of AF patients. The fold change was transformed into t-scores, and t-score is negative (shown in blue) when the compound showed a decline tendency in AF group. Compounds which increased or decreased simultaneously (n=16) or unsynchronized (n=11) in feces and serum were shown in green and pink, respectively.

c, d. The relationship between 16 endogenous metabolites and the top 10 altered genera (c) and species (d) in AF. The 16 metabolites increased or decreased simultaneously in feces and serum were shown in light red and light blue, respectively. Considering the circulating metabolites played the role during the process of GM mediated responses, the serum data of metabonomic was used in spearman's correlation analysis. Red, negative correlation; blue, positive correlation, *$p < 0.05$, +$p < 0.01$. The enriched type of each genera and metabolic patterns was colored according to its direction of enrichment. Blue, enriched in controls; red, enriched in AF patients.

References

1. Lloyd-Jones DM, Wang TJ, Leip EP, Larson MG, Levy D, Vasan RS, et al. Lifetime risk for development of atrial fibrillation: the Framingham Heart Study. Circulation. 2004;110 9:1042-6. doi:10.1161/01.CIR.0000140263.20897.42.
2. Heeringa J, van der Kuip DA, Hofman A, Kors JA, van Herpen G, Stricker BH, et al. Prevalence, incidence and lifetime risk of atrial fibrillation: the Rotterdam study. Eur Heart J. 2006;27 8:949-53. doi:10.1093/eurheartj/ehi825.
3. Haim M, Hoshen M, Reges O, Rabi Y, Balicer R and Leibowitz M. Prospective national study of the prevalence, incidence, management and outcome of a large

contemporary cohort of patients with incident non-valvular atrial fibrillation. J Am Heart Assoc. 2015;4 1:e001486. doi:10.1161/JAHA.114.001486.

4. Oldgren J, Healey JS, Ezekowitz M, Commerford P, Avezum A, Pais P, et al. Variations in cause and management of atrial fibrillation in a prospective registry of 15,400 emergency department patients in 46 countries: the RE-LY Atrial Fibrillation Registry. Circulation. 2014;129 15:1568-76. doi:10.1161/CIRCULATIONAHA.113.005451.

5. Andersson T, Magnuson A, Bryngelsson IL, Frobert O, Henriksson KM, Edvardsson N, et al. All-cause mortality in 272,186 patients hospitalized with incident atrial fibrillation 1995-2008: a Swedish nationwide long-term case-control study. Eur Heart J. 2013;34 14:1061-7. doi:10.1093/eurheartj/ehs469.

6. Marrouche NF, Wilber D, Hindricks G, Jais P, Akoum N, Marchlinski F, et al. Association of atrial tissue fibrosis identified by delayed enhancement MRI and atrial fibrillation catheter ablation: the DECAAF study. JAMA. 2014;311 5:498-506. doi:10.1001/jama.2014.3.

7. Fox CS, Parise H, D'Agostino RB, Sr., Lloyd-Jones DM, Vasan RS, Wang TJ, et al. Parental atrial fibrillation as a risk factor for atrial fibrillation in offspring. JAMA. 2004;291 23:2851-5. doi:10.1001/jama.291.23.2851.

8. Du X, Dong J and Ma C. Is Atrial Fibrillation a Preventable Disease? J Am Coll Cardiol. 2017;69 15:1968-82. doi:10.1016/j.jacc.2017.02.020.

9. Li J, Zhao F, Wang Y, Chen J, Tao J, Tian G, et al. Gut microbiota dysbiosis contributes to the development of hypertension. Microbiome. 2017;5 1:14. doi:10.1186/s40168-016-0222-x.

10. Qin J, Li Y, Cai Z, Li S, Zhu J, Zhang F, et al. A metagenome-wide association study of gut microbiota in type 2 diabetes. Nature. 2012;490 7418:55-60. doi:10.1038/nature11450.

11. Chang CJ, Lin CS, Lu CC, Martel J, Ko YF, Ojcius DM, et al. Ganoderma lucidum reduces obesity in mice by modulating the composition of the gut microbiota. Nat Commun. 2015;6:7489. doi:10.1038/ncomms8489.

12. Qin N, Yang F, Li A, Prifti E, Chen Y, Shao L, et al. Alterations of the human gut microbiome in liver cirrhosis. Nature. 2014;513 7516:59-64. doi:10.1038/nature13568.

13. Feng Q, Liang S, Jia H, Stadlmayr A, Tang L, Lan Z, et al. Gut microbiome development along the colorectal adenoma-carcinoma sequence. Nat Commun. 2015;6:6528. doi:10.1038/ncomms7528.

14. Zhang X, Zhang D, Jia H, Feng Q, Wang D, Liang D, et al. The oral and gut microbiomes are perturbed in rheumatoid arthritis and partly normalized after treatment. Nat Med. 2015;21 8:895-905. doi:10.1038/nm.3914.

15. Jie Z, Xia H, Zhong SL, Feng Q, Li S, Liang S, et al. The gut microbiome in atherosclerotic cardiovascular disease. Nat Commun. 2017;8 1:845. doi:10.1038/s41467-017-00900-1.

16. Su T, Liu R, Lee A, Long Y, Du L, Lai S, et al. Altered Intestinal Microbiota with Increased Abundance of Prevotella Is Associated with High Risk of Diarrhea-Predominant Irritable Bowel Syndrome. Gastroenterol Res Pract. 2018;2018:6961783. doi:10.1155/2018/6961783.

17. Foster JA and McVey Neufeld KA. Gut-brain axis: how the microbiome influences anxiety and depression. Trends Neurosci. 2013;36 5:305-12. doi:10.1016/j.tins.2013.01.005.

18. Schirmer M, Smeekens SP, Vlamakis H, Jaeger M, Oosting M, Franzosa EA, et al. Linking the Human Gut Microbiome to Inflammatory Cytokine Production Capacity. Cell. 2016;167 7:1897. doi:10.1016/j.cell.2016.11.046.

19. Dzeshka MS, Shahid F, Shantsila A and Lip GYH. Hypertension and Atrial Fibrillation: An Intimate Association of Epidemiology, Pathophysiology, and Outcomes. Am J Hypertens. 2017;30 8:733-55. doi:10.1093/ajh/hpx013.

20. Yu L, Meng G, Huang B, Zhou X, Stavrakis S, Wang M, et al. A potential relationship between gut microbes and atrial fibrillation: Trimethylamine N-oxide, a gut microbe-derived metabolite, facilitates the progression of atrial fibrillation. Int J Cardiol. 2018;255:92-8. doi:10.1016/j.ijcard.2017.11.071.
21. Pastori D, Carnevale R, Nocella C, Novo M, Santulli M, Cammisotto V, et al. Gut-Derived Serum Lipopolysaccharide is Associated With Enhanced Risk of Major Adverse Cardiovascular Events in Atrial Fibrillation: Effect of Adherence to Mediterranean Diet. J Am Heart Assoc. 2017;6 6 doi:10.1161/JAHA.117.005784.
22. Fretts AM, Mozaffarian D, Siscovick DS, Djousse L, Heckbert SR, King IB, et al. Plasma phospholipid saturated fatty acids and incident atrial fibrillation: the Cardiovascular Health Study. J Am Heart Assoc. 2014;3 3:e000889. doi:10.1161/JAHA.114.000889.
23. Horas HNS, Nishiumi S, Kawano Y, Kobayashi T, Yoshida M and Azuma T. Adrenic acid as an inflammation enhancer in non-alcoholic fatty liver disease. Arch Biochem Biophys. 2017;623-624:64-75. doi:10.1016/j.abb.2017.04.009.
24. Kirchhof P, Benussi S, Kotecha D, Ahlsson A, Atar D, Casadei B, et al. 2016 ESC Guidelines for the management of atrial fibrillation developed in collaboration with EACTS. Eur Heart J. 2016;37 38:2893-962. doi:10.1093/eurheartj/ehw210.
25. Joossens M, Huys G, Cnockaert M, De Preter V, Verbeke K, Rutgeerts P, et al. Dysbiosis of the faecal microbiota in patients with Crohn's disease and their unaffected relatives. Gut. 2011;60 5:631-7. doi:10.1136/gut.2010.223263.
26. Hoffmann TW, Pham HP, Bridonneau C, Aubry C, Lamas B, Martin-Gallausiaux C, et al. Microorganisms linked to inflammatory bowel disease-associated dysbiosis differentially impact host physiology in gnotobiotic mice. ISME J. 2016;10 2:460-77. doi:10.1038/ismej.2015.127.

27. Machiels K, Sabino J, Vandermosten L, Joossens M, Arijs I, de Bruyn M, et al. Specific members of the predominant gut microbiota predict pouchitis following colectomy and IPAA in UC. Gut. 2017;66 1:79-88. doi:10.1136/gutjnl-2015-309398.

28. Cui X, Ye L, Li J, Jin L, Wang W, Li S, et al. Metagenomic and metabolomic analyses unveil dysbiosis of gut microbiota in chronic heart failure patients. Sci Rep. 2018;8 1:635. doi:10.1038/s41598-017-18756-2.

29. Hurst JR, Kasper KJ, Sule AN and McCormick JK. Streptococcal pharyngitis and rheumatic heart disease: the superantigen hypothesis revisited. Infect Genet Evol. 2018;61:160-75. doi:10.1016/j.meegid.2018.03.006.

30. Brook I. Veillonella infections in children. J Clin Microbiol. 1996;34 5:1283-5.

31. Zheng JX, Wu Y, Lin ZW, Pu ZY, Yao WM, Chen Z, et al. Characteristics of and Virulence Factors Associated with Biofilm Formation in Clinical Enterococcus faecalis Isolates in China. Front Microbiol. 2017;8:2338. doi:10.3389/fmicb.2017.02338.

32. Riviere A, Gagnon M, Weckx S, Roy D and De Vuyst L. Mutual Cross-Feeding Interactions between Bifidobacterium longum subsp. longum NCC2705 and Eubacterium rectale ATCC 33656 Explain the Bifidogenic and Butyrogenic Effects of Arabinoxylan Oligosaccharides. Appl Environ Microbiol. 2015;81 22:7767-81. doi:10.1128/AEM.02089-15.

33. Matson V, Fessler J, Bao R, Chongsuwat T, Zha Y, Alegre ML, et al. The commensal microbiome is associated with anti-PD-1 efficacy in metastatic melanoma patients. Science. 2018;359 6371:104-8. doi:10.1126/science.aao3290.

34. Takahashi K, Nishida A, Fujimoto T, Fujii M, Shioya M, Imaeda H, et al. Reduced Abundance of Butyrate-Producing Bacteria Species in the Fecal Microbial Community in Crohn's Disease. Digestion. 2016;93 1:59-65. doi:10.1159/000441768.

35. Miquel S, Martin R, Rossi O, Bermudez-Humaran LG, Chatel JM, Sokol H, et al. Faecalibacterium prausnitzii and human intestinal health. Curr Opin Microbiol. 2013;16 3:255-61. doi:10.1016/j.mib.2013.06.003.

36. Sokol H, Pigneur B, Watterlot L, Lakhdari O, Bermudez-Humaran LG, Gratadoux JJ, et al. Faecalibacterium prausnitzii is an anti-inflammatory commensal bacterium identified by gut microbiota analysis of Crohn disease patients. Proc Natl Acad Sci U S A. 2008;105 43:16731-6. doi:10.1073/pnas.0804812105.

37. Newton RJ, McLellan SL, Dila DK, Vineis JH, Morrison HG, Eren AM, et al. Sewage reflects the microbiomes of human populations. MBio. 2015;6 2:e02574. doi:10.1128/mBio.02574-14.

38. Jiang H, Ling Z, Zhang Y, Mao H, Ma Z, Yin Y, et al. Altered fecal microbiota composition in patients with major depressive disorder. Brain Behav Immun. 2015;48:186-94. doi:10.1016/j.bbi.2015.03.016.

39. Nagai F, Morotomi M, Watanabe Y, Sakon H and Tanaka R. Alistipes indistinctus sp. nov. and Odoribacter laneus sp. nov., common members of the human intestinal microbiota isolated from faeces. Int J Syst Evol Microbiol. 2010;60 Pt 6:1296-302. doi:10.1099/ijs.0.014571-0.

40. Finegold S, Summanen P, Hunt Gerardo S and Baron E. Clinical importance of Bilophila wadsworthia. Eur J Clin Microbiol Infect Dis. 1992;11 11:1058-63.

41. Kasai C, Sugimoto K, Moritani I, Tanaka J, Oya Y, Inoue H, et al. Comparison of the gut microbiota composition between obese and non-obese individuals in a Japanese population, as analyzed by terminal restriction fragment length polymorphism and next-generation sequencing. BMC Gastroenterol. 2015;15:100. doi:10.1186/s12876-015-0330-2.

42. Fu J, Bonder MJ, Cenit MC, Tigchelaar EF, Maatman A, Dekens JA, et al. The Gut Microbiome Contributes to a Substantial Proportion of the Variation in

Blood Lipids. Circ Res. 2015;117 9:817-24. doi:10.1161/CIRCRESAHA.115.306807.

43. Wu H, Esteve E, Tremaroli V, Khan MT, Caesar R, Manneras-Holm L, et al. Metformin alters the gut microbiome of individuals with treatment-naive type 2 diabetes, contributing to the therapeutic effects of the drug. Nat Med. 2017;23 7:850-8. doi:10.1038/nm.4345.

44. Zhernakova A, Kurilshikov A, Bonder MJ, Tigchelaar EF, Schirmer M, Vatanen T, et al. Population-based metagenomics analysis reveals markers for gut microbiome composition and diversity. Science. 2016;352 6285:565-9. doi:10.1126/science.aad3369.

45. Kanehisa M, Araki M, Goto S, Hattori M, Hirakawa M, Itoh M, et al. KEGG for linking genomes to life and the environment. Nucleic Acids Res. 2008;36 Database issue:D480-4. doi:10.1093/nar/gkm882.

46. Huerta-Cepas J, Szklarczyk D, Forslund K, Cook H, Heller D, Walter MC, et al. eggNOG 4.5: a hierarchical orthology framework with improved functional annotations for eukaryotic, prokaryotic and viral sequences. Nucleic Acids Res. 2016;44 D1:D286-93. doi:10.1093/nar/gkv1248.

47. Wang J, Dong R and Zheng S. Roles of the inflammasome in the gutliver axis (Review). Mol Med Rep. 2018; doi:10.3892/mmr.2018.9679.

48. Gao H, Chen LJ, Luo QQ, Liu XX, Hu Y, Yu LL, et al. Effect of cholic acid on fetal cardiac myocytes in intrahepatic choliestasis of pregnancy. J Huazhong Univ Sci Technolog Med Sci. 2014;34 5:736-9. doi:10.1007/s11596-014-1344-7.

49. Han JY, Jeong HI, Park CW, Yoon J, Ko J, Nam SJ, et al. Cholic Acid Attenuates ER Stress-Induced Cell Death in Coxsackievirus-B3 Infection. J Microbiol Biotechnol. 2018;28 1:109-14. doi:10.4014/jmb.1708.08009.

50. Fung TT, Rexrode KM, Mantzoros CS, Manson JE, Willett WC and Hu FB. Mediterranean diet and incidence of and mortality from coronary heart disease

and stroke in women. Circulation. 2009;119 8:1093-100. doi:10.1161/CIRCULATIONAHA.108.816736.

51. Al-Shudiefat AA, Sharma AK, Bagchi AK, Dhingra S and Singal PK. Oleic acid mitigates TNF-alpha-induced oxidative stress in rat cardiomyocytes. Mol Cell Biochem. 2013;372 1-2:75-82. doi:10.1007/s11010-012-1447-z.

52. Farvid MS, Ding M, Pan A, Sun Q, Chiuve SE, Steffen LM, et al. Dietary linoleic acid and risk of coronary heart disease: a systematic review and meta-analysis of prospective cohort studies. Circulation. 2014;130 18:1568-78. doi:10.1161/CIRCULATIONAHA.114.010236.

53. Jiang M, Zhang H, Zhai L, Ye B, Cheng Y and Zhai C. ALA/LA ameliorates glucose toxicity on HK-2 cells by attenuating oxidative stress and apoptosis through the ROS/p38/TGF-beta1 pathway. Lipids Health Dis. 2017;16 1:216. doi:10.1186/s12944-017-0611-6.

54. Serena C, Ceperuelo-Mallafre V, Keiran N, Queipo-Ortuno MI, Bernal R, Gomez-Huelgas R, et al. Elevated circulating levels of succinate in human obesity are linked to specific gut microbiota. ISME J. 2018;12 7:1642-57. doi:10.1038/s41396-018-0068-2.

55. Kang JX and Leaf A. Protective effects of free polyunsaturated fatty acids on arrhythmias induced by lysophosphatidylcholine or palmitoylcarnitine in neonatal rat cardiac myocytes. Eur J Pharmacol. 1996;297 1-2:97-106.

56. Menni C, Lin C, Cecelja M, Mangino M, Matey-Hernandez ML, Keehn L, et al. Gut microbial diversity is associated with lower arterial stiffness in women. Eur Heart J. 2018;39 25:2390-7. doi:10.1093/eurheartj/ehy226.

57. Zhou X, Li J, Guo J, Geng B, Ji W, Zhao Q, et al. Gut-dependent microbial translocation induces inflammation and cardiovascular events after ST-elevation myocardial infarction. Microbiome. 2018;6 1:66. doi:10.1186/s40168-018-0441-4.

58. Yin J, Liao SX, He Y, Wang S, Xia GH, Liu FT, et al. Dysbiosis of Gut Microbiota With Reduced Trimethylamine-N-Oxide Level in Patients With Large-Artery Atherosclerotic Stroke or Transient Ischemic Attack. J Am Heart Assoc. 2015;4 11 doi:10.1161/JAHA.115.002699.

59. Louis P and Flint HJ. Diversity, metabolism and microbial ecology of butyrate-producing bacteria from the human large intestine. FEMS Microbiol Lett. 2009;294 1:1-8. doi:10.1111/j.1574-6968.2009.01514.x.

60. Maruhashi T, Soga J, Fujimura N, Idei N, Mikami S, Iwamoto Y, et al. Endothelial Dysfunction, Increased Arterial Stiffness, and Cardiovascular Risk Prediction in Patients With Coronary Artery Disease: FMD-J (Flow-Mediated Dilation Japan) Study A. J Am Heart Assoc. 2018;7 14 doi:10.1161/JAHA.118.008588.

61. Zhao L, Zhang F, Ding X, Wu G, Lam YY, Wang X, et al. Gut bacteria selectively promoted by dietary fibers alleviate type 2 diabetes. Science. 2018;359 6380:1151-6. doi:10.1126/science.aao5774.

62. Scher JU, Sczesnak A, Longman RS, Segata N, Ubeda C, Bielski C, et al. Expansion of intestinal Prevotella copri correlates with enhanced susceptibility to arthritis. Elife. 2013;2:e01202. doi:10.7554/eLife.01202.

63. Brown JM and Hazen SL. Microbial modulation of cardiovascular disease. Nat Rev Microbiol. 2018;16 3:171-81. doi:10.1038/nrmicro.2017.149.

64. Kirchhof P, Benussi S, Kotecha D, Ahlsson A, Atar D, Casadei B, et al. 2016 ESC Guidelines for the management of atrial fibrillation developed in collaboration with EACTS. Europace. 2016;18 11:1609-78. doi:10.1093/europace/euw295.

65. Vizzardi E, Curnis A, Latini MG, Salghetti F, Rocco E, Lupi L, et al. Risk factors for atrial fibrillation recurrence: a literature review. J Cardiovasc Med (Hagerstown). 2014;15 3:235-53. doi:10.2459/JCM.0b013e328358554b.

66. Wu S, Huang Z, Yang X, Zhou Y, Wang A, Chen L, et al. Prevalence of ideal cardiovascular health and its relationship with the 4-year cardiovascular events in a northern Chinese industrial city. Circ Cardiovasc Qual Outcomes. 2012;5 4:487-93. doi:10.1161/CIRCOUTCOMES.111.963694.

67. Arumugam M, Raes J, Pelletier E, Le Paslier D, Yamada T, Mende DR, et al. Enterotypes of the human gut microbiome. Nature. 2011;473 7346:174-80. doi:10.1038/nature09944.

68. Qin J, Li R, Raes J, Arumugam M, Burgdorf KS, Manichanh C, et al. A human gut microbial gene catalogue established by metagenomic sequencing. Nature. 2010;464 7285:59-65. doi:10.1038/nature08821.

69. Greenblum S, Turnbaugh PJ and Borenstein E. Metagenomic systems biology of the human gut microbiome reveals topological shifts associated with obesity and inflammatory bowel disease. Proc Natl Acad Sci U S A. 2012;109 2:594-9. doi:10.1073/pnas.1116053109.

70. Nielsen HB, Almeida M, Juncker AS, Rasmussen S, Li J, Sunagawa S, et al. Identification and assembly of genomes and genetic elements in complex metagenomic samples without using reference genomes. Nat Biotechnol. 2014;32 8:822-8. doi:10.1038/nbt.2939.

71. Zuo K; Li J; Li K; Hu C; Gao Y; Chen M; Hu R; Liu Y; Chi H; Wang H; Qin Y; Liu X; Li S; Cai J; Zhong J; Yang X: Supporting data for "Disordered gut microbiota and alterations in metabolic patterns are associated with atrial fibrillation" *GigaScience* Database. 2019. http://dx.doi.org/10.5524/100597

Figure 1-r1. Elevated microbiota richness and altered community types in AF patients

Click here to access/download;Figure;Figure 1 -r1.pdf

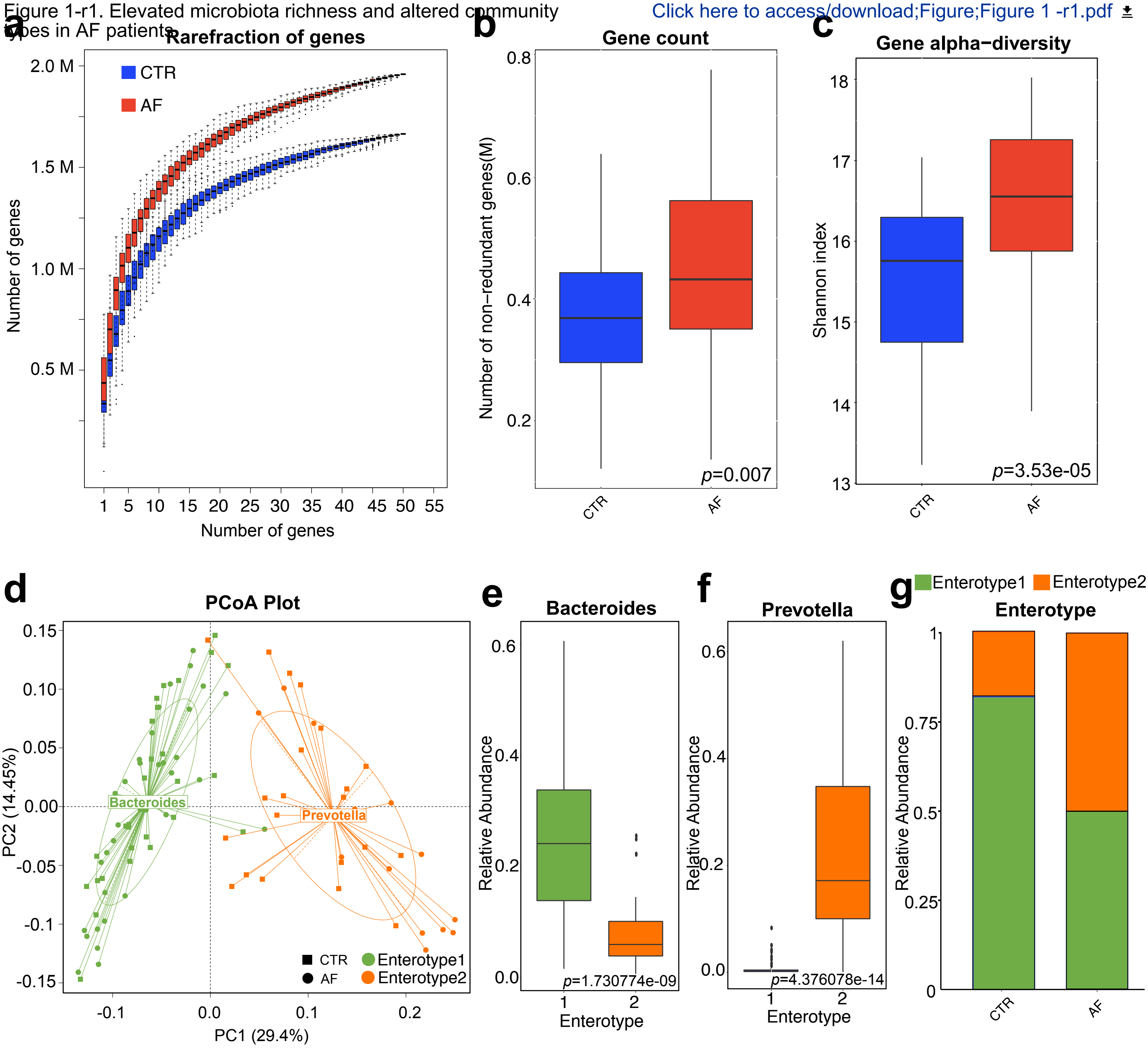

Figure 2-r1. Genera strikingly different across groups.

Click here to access/download;Figure;Figure 2-r1.pdf

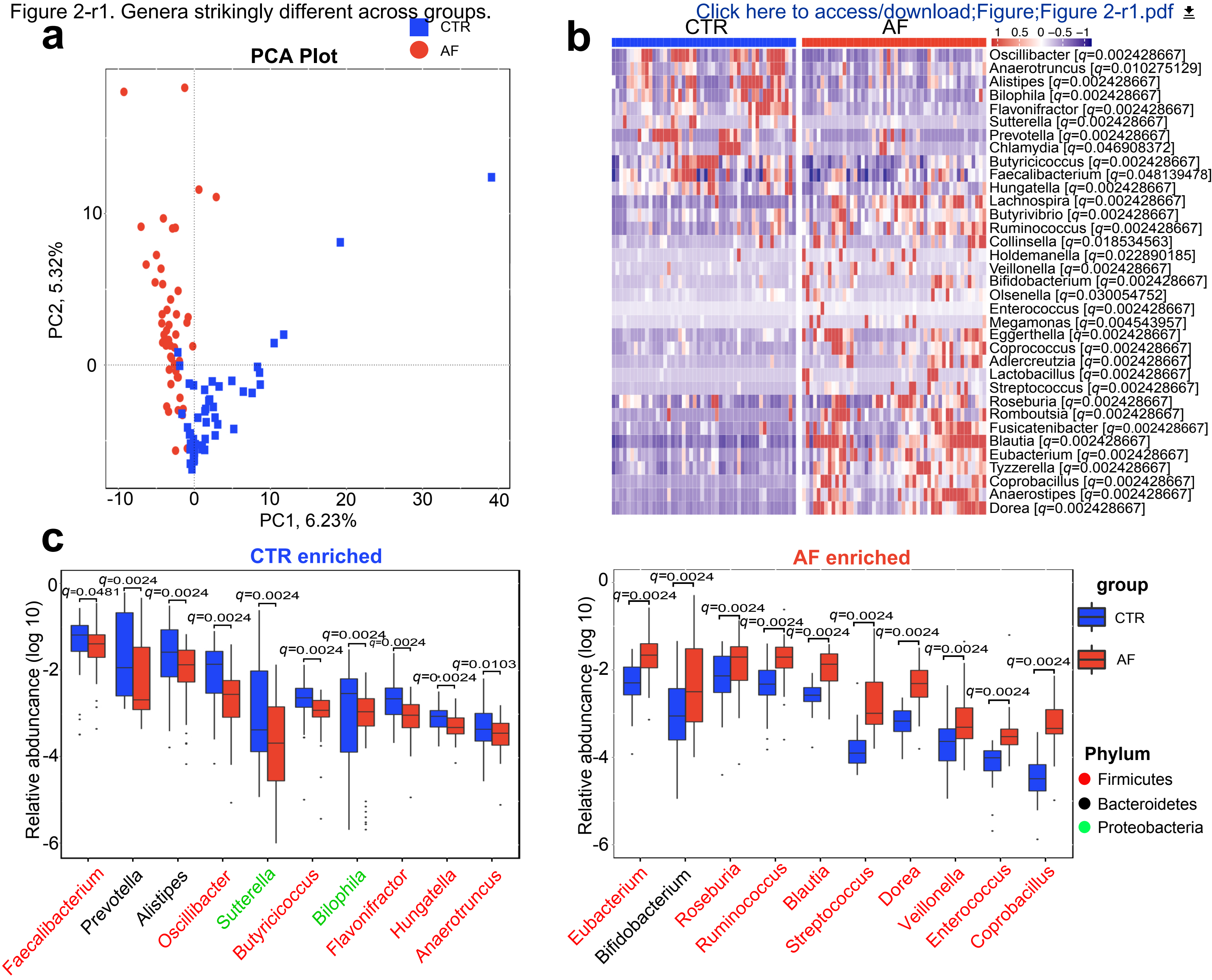

Figure 3-r1. Gut CAGs classify AF from controls.

Click here to access/download;Figure;Figure 3-r1.pdf

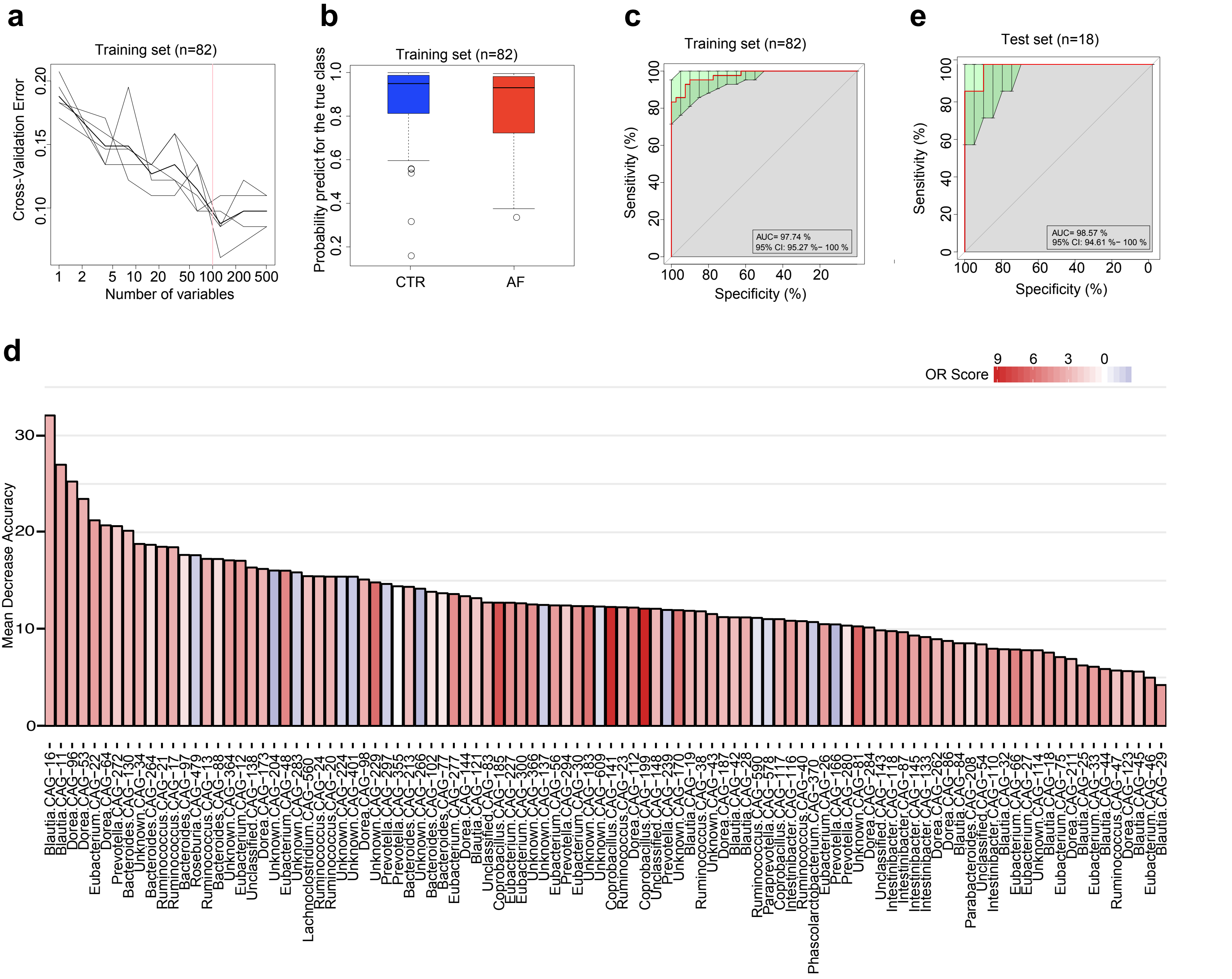

Figure 4-r1. Microbial gene functions annotation in AF.

Click here to access/download;Figure;Figure 4-r1.pdf

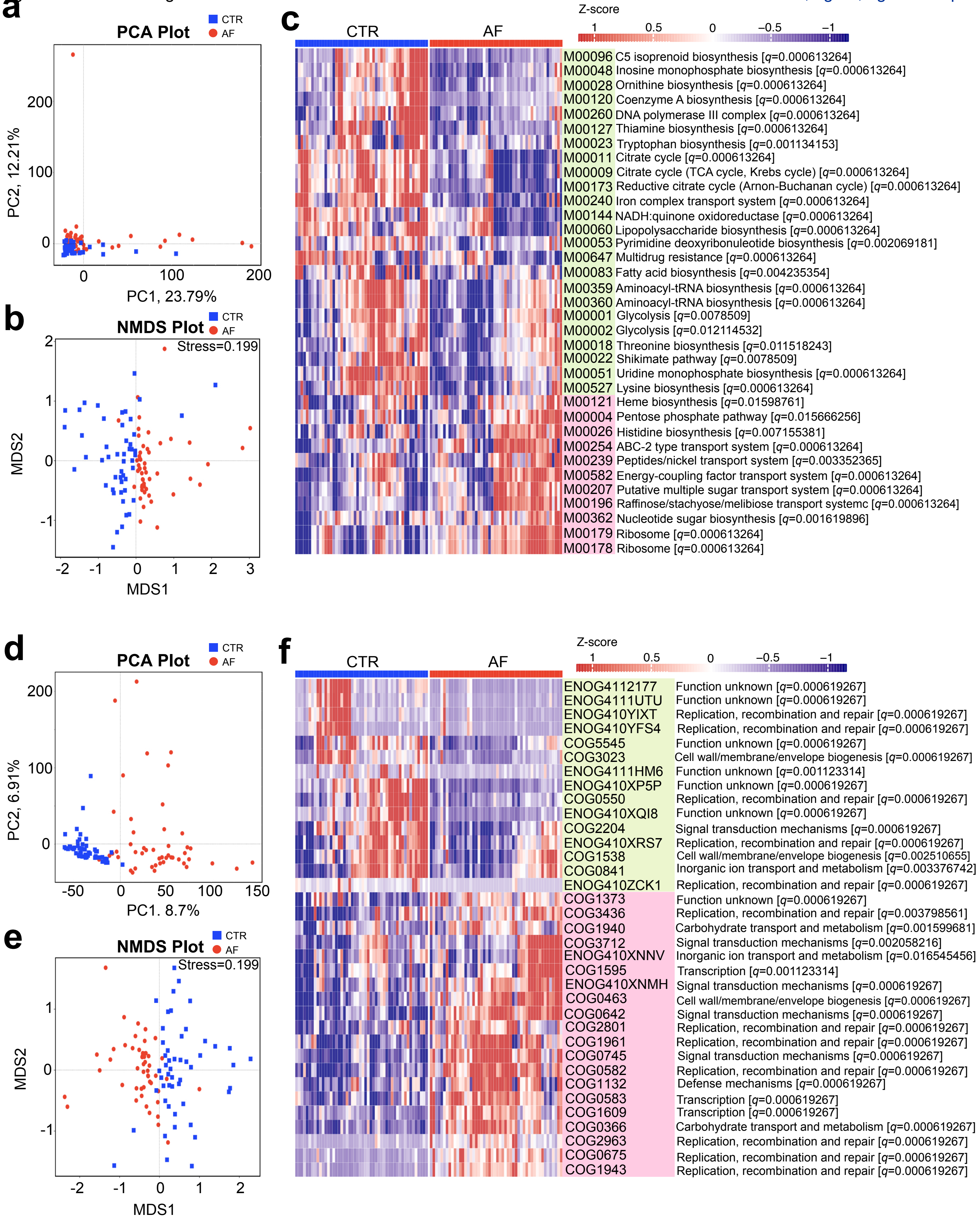

Figure 5-r1. Distinguished metabolic patterns between AF and control.

Click here to access/download;Figure;Figure 5-r1.pdf

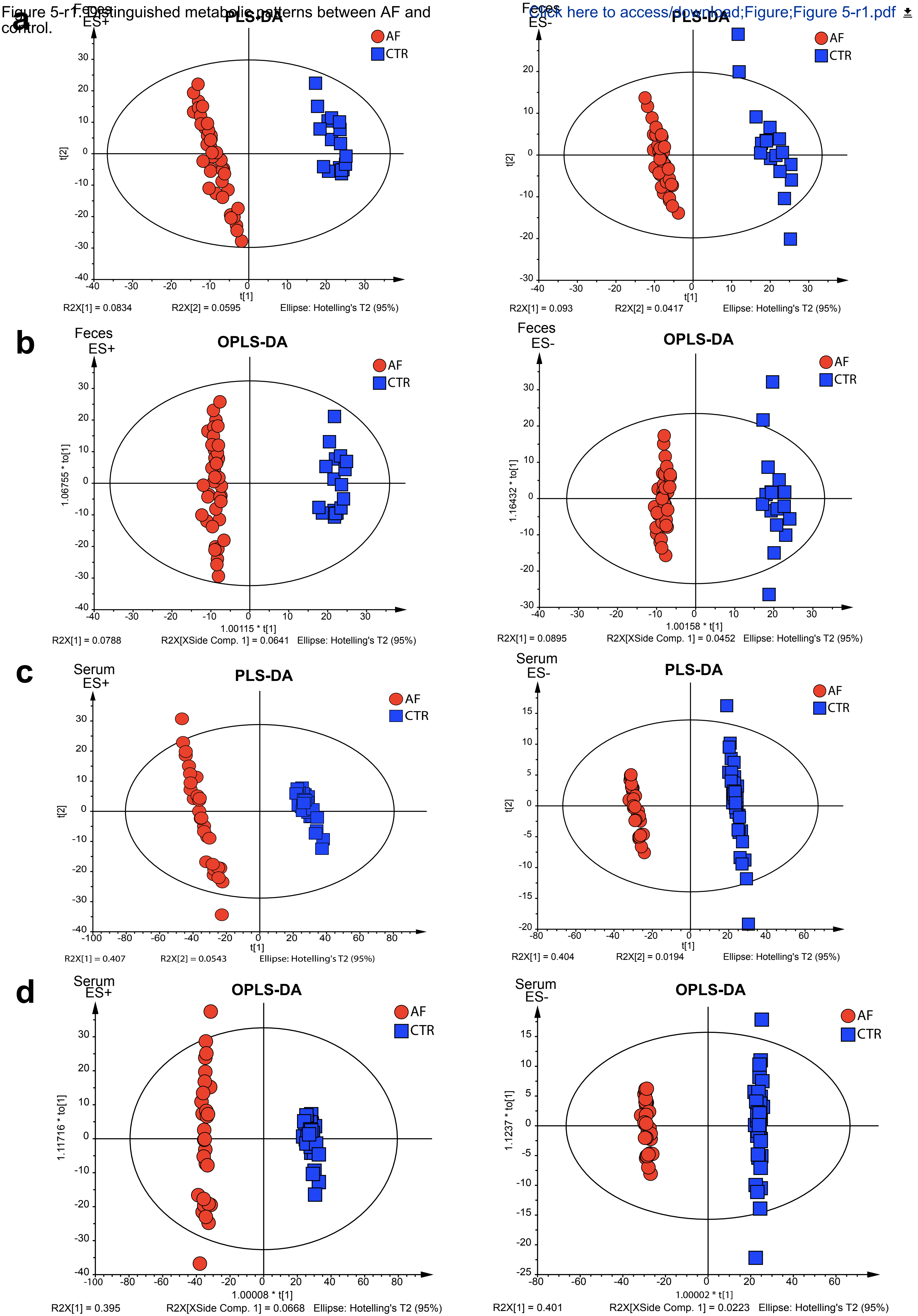

Figure 6-r1. Aberrant metabolic patterns related to AF

Click here to access/download;Figure;Figure 6-r1.pdf

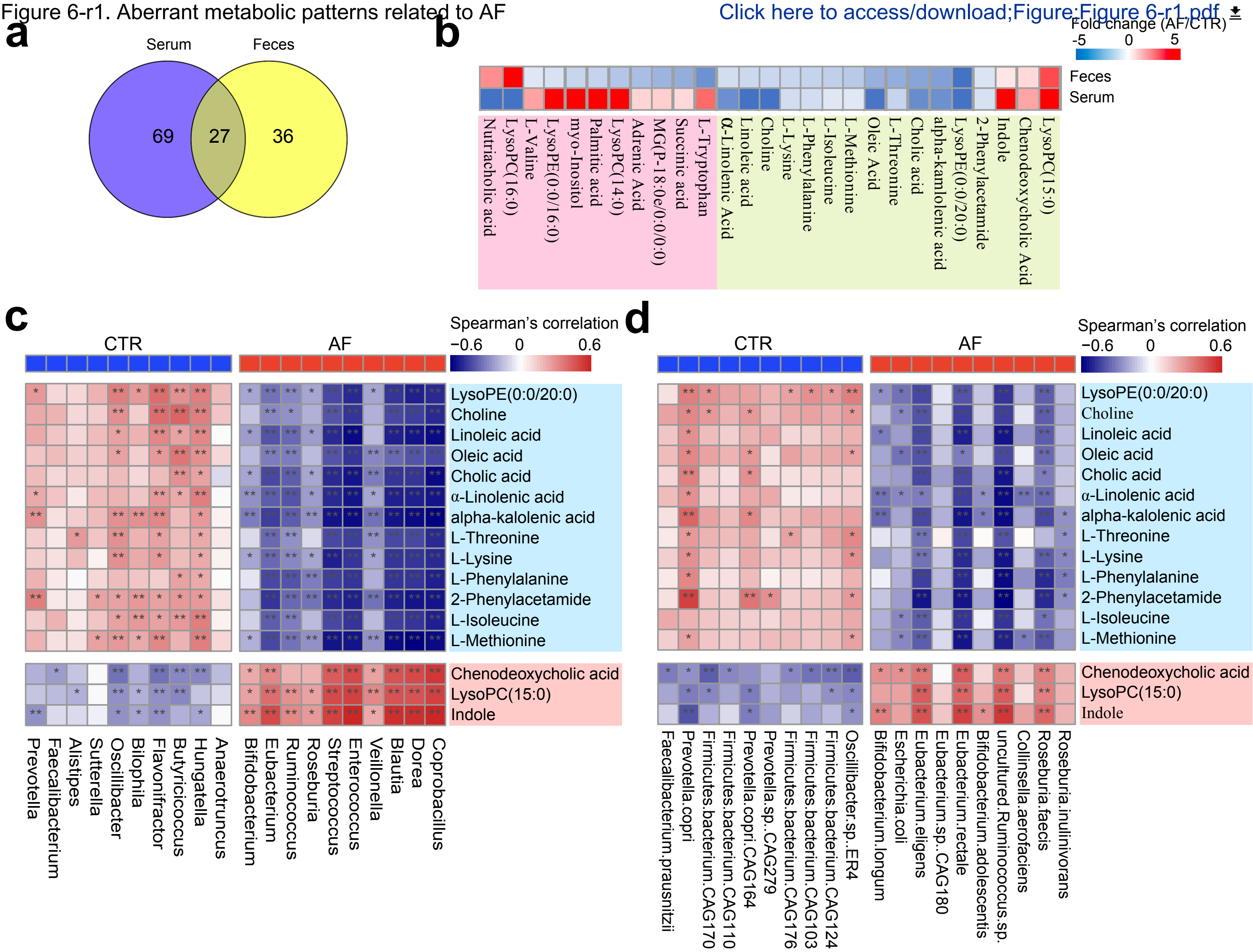

Figure S1-r1. Increased Firmicutes/Bacteroidetes ratio, Pielou evenness and Chao richness in AF.

Click here to access/download
Supplementary Material
Figure S1-r1.pdf

Figure S2-r1. Another 12 genera significantly enriched in enterotype 1.

Click here to access/download
Supplementary Material
Figure S2-r1.pdf

Figure S3-r1. Enterotype analysis at the species level.

Click here to access/download
Supplementary Material
Figure S3-r1.pdf

Figure S4-r1. Taxonomic annotation and abundance profiling at the genus level.

Click here to access/download
Supplementary Material
Figure S4-o.pdf

Figure S5-r1. Species strikingly different across groups.

Click here to access/download
Supplementary Material
Figure S5-r1.pdf

Figure S6-r1. Influents of baseline characteristics, including age, gender, T2DM, TC and medication on GM.

Click here to access/download
Supplementary Material
Figure S6-r1.pdf

Figure S7-r1. Size distribution and taxonomic assignment of CAGs.

Click here to access/download
Supplementary Material
Figure S7-r1.pdf

Figure S8-r1. The network of CAGs enriched in AF compared with controls.

Click here to access/download
Supplementary Material
Figure S8-r1.pdf

Figure S9-r1. Gut CAGs (variables in 5, 10, 20, 50, 70) classify AF from controls.

Click here to access/download
Supplementary Material
Figure S9-o.pdf

Figure S10-r1. Correlation between CAGs and altered function module.

Click here to access/download
Supplementary Material
Figure S10-r1.pdf

Figure S11-r1. Metabolites differentially enriched in AF and controls in serum.

Click here to access/download
Supplementary Material
Figure S11-r1.pdf

Figure S12-r1. Metabolites differentially enriched in AF and controls in feces.

Click here to access/download
Supplementary Material
Figure S12-r1.pdf

Supplementary Material-Supplementary tables-table S1-12, revised S15.xlsx

Click here to access/download
Supplementary Material
renamed_6fe6c.xlsx

Table S13-r1. Baseline characteristics of serum samples from metabolomic analyses.

Click here to access/download
Supplementary Material
renamed_b2fb8.docx

Table S14-r1. Baseline characteristics of feces samples from metabolomic analyses.

Click here to access/download
Supplementary Material
renamed_d9d24.docx

Click here to access/download
Supplementary Material
Additional files 28 computational code.docx

Click here to access/download
Supplementary Material
GIGA Response to Reviewers-2019-4-12.docx

cover letter

Click here to access/download;Personal Cover;Cover letter from Kun Zuo GS.docx

Dear Editor,

We would like to submit our manuscript entitled "Disordered gut microbiota and alterations in metabolic patterns are associated with atrial fibrillation" to ***GigaScience*** for consideration as an Article. All of the authors have approved the enclosed manuscript, and no conflicts of interest exist. On behalf of all of the authors, I declare that the work described is original research that has not been previously published nor is under consideration for publication elsewhere, in whole or in part.

Atrial Fibrillation (AF) is one of the most common cardiac arrhythmias with worldwide prevalence, increasing disability and morbidity. It has become a major global public health concern. Although a correlation between gut microbiota (GM) and AF has recently been reported in a canine study, the signature change in gut microbes and their fermentation products in human populations with AF remains largely unknown. The direct evidence of altered gut bacteria in AF patients and gut dysbiosis contribution to aberrant metabolic patterns that accelerate AF progression still needs to be identified.

For the purpose above, we performed metagenomic sequencing and analyses of stool samples from patients with AF outlining the potential compositional and functional alterations of GM in 100 Chinese participants. Beyond exploring the relationship between disordered GM and altered metabolomic profiles in AF, we also aimed to construct a microbiota-dependent discrimination index for distinguishing AF, and thus provide a comprehensive understanding of GM dysbiosis in the progression of AF. Our findings describe the disordered patterns of GM and aberrant microbial-related metabolites in a cohort of AF patients for the first time. Specifically, our main novel findings are listed as follows:

(1) We reported for the first time the global alterations occurring in the intestinal microbiota of AF patients, including a dramatic elevation in microbial richness and diversity, a disorder in gut enterotype distribution and a specific perturbation of GM composition.

(2) We observed an imbalance of gut microbial function and changes in metabolic patterns in the fecal and serum samples from the AF group; we were able to identify a correlation between the GM and their endogenous metabolic products in AF patients.
(3) We captured the microbial features for AF patients, revealed their common microbial characteristics and highlighted the potential clinical value of GM in distinguishing AF by constructing a random forest disease classifier.

Recently, there has been extensive attention directed towards the gut microbiome in the development of AF but limited evidence has hence surfaced. We believe that our study directly demonstrates the crucial contribution of disordered GM to AF pathogenesis, and therefore is of substantial interest to the readers of ***GigaScience***.

We greatly appreciate your interest and encouragement concerning our manuscript. We look forward to receiving comments from you and the reviewers. If you have any questions, please do not hesitate to contact me at the address below.

Sincerely yours,

Xinchun Yang, MD, PhD
Heart Center, Beijing ChaoYang Hospital, Capital Medical University,
Beijing Key Laboratory of Hypertension,
8th Gongtinanlu Rd, Chaoyang District, Beijing, China, 100020
Tel: 86-10-85231937
Fax: 86-10-85231937
E-mail: yxc6229@163.com
Jing Li, MD, PhD
Heart Center, Beijing ChaoYang Hospital, Capital Medical University,
Beijing Key Laboratory of Hypertension,
8th Gongtinanlu Rd, Chaoyang District, Beijing, China, 100020
Tel: 86-10-85231937
Fax: 86-10-85231937

E-mail: lijing11999@126.com
