# Supplementary material for: Disordered gut microbiota and alterations in metabolic patterns are associated with atrial fibrillation
Source: Gigascience. 2019 May 30;8(6):giz058. doi: 10.1093/gigascience/giz058 (PMC6543127; doi:10.1093/gigascience/giz058)
Supplement: giz058_Supplement_Files [file giz058_supplement_files.zip › Additional files 28 computational code.docx]

**The computational code of step by step for bioinformatic analysis.**

**###### Metagenomic sequencing, gene catalogue construction ######**

**#### begin ####**

**### step1: Quality control(Shell) ###**

java -jar trimmomatic-0.36.jar PE -phred33 -threads 8 sampleA.raw_1.fq.gz sampleA.raw_2.fq.gz sampleA.pe.clean_1.fq.gz sampleA.se.clean_1.fq.gz sampleA.pe.clean_2.fq.gz sampleA.se.clean_2.fq.gz LEADING:3 SLIDINGWINDOW:4:15 MINLEN:36

# For other samples, the script is same with sampleA

**### step2: Aligned the reads to the human genome(Shell) ###**

soap -s 135 -l 30 -v 7 -m 200 -x 400 -D ucsc.hg19.fasta.index -a sampleA.pe.clean_1.fq.gz -b sampleA.pe.clean_2.fq.gz -o sampleA.hg19.pe.soap -2 sampleA.hg19.se.soap -u sampleA.unmapping.fa

# For other samples, the script is same with sampleA

**### step3: Assembly of clean reads(Shell) ###**

SOAPdenovo-63mer all -d 1 -M 3 -R -u –F -s sampleA.soapdenovo.config -K 49 -o SampleA

# sampleA.soapdenovo.config:

max_rd_len=150

[LIB]

avg_ins=300

reverse_seq=0

asm_flags=3

rank=1

f=sampleA.unmapping.fa

# We used a series of k-mer values (from 49 to 87) and chose the optimal one with the longest N50 value for the remaining scaffolds(perl).

use strict;

use warnings;

die"usage:perl $0 <in:input seqs> <out:result>\n" unless @ARGV;

my $result_seqs=pop@ARGV;

my @n50;

foreach my $path (@ARGV){

my $n50=&N_50($path);

$n50 ||= 0;

push @n50,$n50;

}

my ($max_n50,$max_num);

for(my $j=0;$j<@ARGV;$j++){

if(! $max_n50 || $max_n50 < $n50[$j]){

$max_n50=$n50[$j];

$max_num=$j;

}

}

print “MAX N 50:$ARGV[$max_num]\n”;

sub N_50{

my ($path,)=@_;

my ($len,$total)=(0,0);

my @x;

open(FA,$path)||die;

while(<FA>){

if(/^[\>\@]/){

if($len>0){$total+=$len;push @x,$len;}

$len=0;

}else{

s/\s//g;$len+=length($_);

}

}

if($len>0){$total+=$len;push @x,$len;}

@x=sort{$b<=>$a} @x;

my ($count,$half)=(0,0);

for (my $j=0;$j<@x;$j++){

$count+=$x[$j];

if (($count>=$total/2)&&($half==0)){

#print "N50: $x[$j]\n";

$half=$x[$j];

close FA;

return $half;

}

}

}

# For other samples, the script is same with sampleA

**### step4: Assembly of unused reads(Shell) ###**

soap -s 119 -m 200 -x 400 -D sampleA.scaffold.fa -a sampleA. clean_1.fq.gz -b sampleA. clean_2.fq.gz -o sampleA.clean.pe.soap -2 sampleA.clean.se.soap -u sampleA.unmapping.fa

# Assembly of unmapping reads by SOAP de novo, the script is same with sampleA

**### step5: Gene prediction from the assembled contigs(Shell) ###**

gmhmmp -a -d -f G -m MetaGeneMark_v1.mod SampleA.scaffold.fa -A sampleA_protein.fasta -D sampleA_nucleotide.fasta

# For other samples, the script is same with sampleA

**### step6: Construction of a non-redundant gene catalogue(Shell) ###**

cat sample[ABCDE…]_protein.fasta >total.samples_protein.fasta

cat sample[ABCDE…]_ nucleotide.fasta > total.samples_nucl.fasta

cd-hit -G 0 -aS 0.9 -g 1 -d 0 -c 0.95 -i total.samples_protein.fasta -o NonRundant.gene.prot.fasta

**### step7: Aligned the reads to the gene catalogue (Shell) ###**

soap -m 200 -x 400 -s 119 -a sampleA.clean.fq1.gz -b sampleA.clean.fq2.gz -D NonRundant.gene.nucl.fasta.index -o sampleA_PE.soap -2 sampleA_SE.soap

# From sampleA_PE.soap and sampleA_SE.soap, The gene abundance was calculated by counting the number of reads and normalizing by gene length(perl).

use warinings;

use strict;

die"usage:perl $0 <in:pe.soap> <in:se.soap> <in:len> <output>\n" unless @ARGV ==4;

my ($pe_file,$se_file,$len,$out)=@ARGV;

my %gene2len;

open IN,$len;

while(<IN>){

chomp;

my($id,$length)=(split/\s+/)[0,1];

$gene2len{$id}=$length;

}

close IN;

my %gene;

$pe_file =~ /.gz$/ ? open PE, "gzip -dc $pe_file |" : open PE, $pe_file ;

while (<PE>) {

chomp;

my @tmp = split();

if ( $tmp[3] == 1 ) {

$gene{$tmp[7]} += 0.5 if exists $gene{$tmp[7]};

}

}

close PE;

$se_file =~ /.gz$/ ? open SE, "gzip -dc $se_file |" : open SE, $se_file ;

my ( $prid, $pgid );

while (<SE>) {

chomp;

my ( $rid, $gid ) = (split)[ 0, 7 ];

if ( $prid && $pgid ) {

if ( $prid eq $rid && $pgid eq $gid ) {

$gene{$gid}++;

( $prid, $pgid ) = ();

}else {

$gene{$pgid}++;

( $prid, $pgid ) = ( $rid, $gid );

}

}else {

( $prid, $pgid ) = ( $rid, $gid );

}

}

close SE;

my $total;

foreach my $gene (keys %gene){$total += $gene{$gene}/$gene2len{$gene};}

open OUT,">$out" || die $!;

print OUT "Gene_ID\tGene_Reads\n";

foreach my $key (sort {$gene{$b} <=> $gene{$a}} keys %gene ) {

print OUT "$key\t”.($gene{$key}/$gene2len{$gene})/$total.”\n";

}

close OUT;

**#### end ####**

**###### Analysis of genera richness and enterotypes ######**

**#### begin ####**

**## step1: Alpha diversity(Shell) ##**

biom convert -i Unigenes.readsNum.even.xls -o Unigenes.readsNum.even.biom --table-type "OTU table" --to-json

python alpha_diversity.py -i Unigenes.readsNum.even.biom -m observed_species,shannon,chao1 -o Unigenes.alpha_index.txt

**## step2: Analysis of enterotypes(Shell) ##**

As mentioned by the website(http://enterotype.embl.de/enterotypes.html), It explained how enterotypes are calculated.

**## step3: calculate the Spearman’s correlation between genera(R) ##**

vare <- t(read.table("taxonomy.table.txt",sep="\t",header=T,row.names=1))

corr <- cor(vare,vare,method="spearman")

**#### end ####**

**###### Taxonomic** **assignment, annotation and abundance profiling ######**

**#### begin ####**

# Alignment by diamond

diamond blastp -d Micro_NR.fa -k 50 -sensitive -e 0.00001 -q NonRundant.gene.prot.fasta -a NonRundant.gene.prot.fasta

diamond view -a NonRundant.gene.prot.fasta.daa -o NonRundant.gene.prot.fasta.m8

# To distinguish taxonomic groups, the significant matches for each gene, defined by e-values ≤10 × e-value of the top hit(perl)

use strict;

use warnings;

my %result;

while(<>){

chomp;

my($id,$evalue)=(split/\s+/)[0,10];

if($result{$id}){

print “$_\n” if $evalue <= $result{$id}{cutoff} ;

}else{

$result{$id}{cutoff}=$evalue*10;

print “$_\n”;

}

}

# Using MEGAN to determine the taxonomical level of each gene by the lowest common ancestor-based algorithm

**#### end ####**

**###### Functional annotation ######**

**#### begin ####**

**## step1: For KEGG annotation(Shell) ##**

diamond blastp -d KEGG.database.fa -k 50 -sensitive -e 0.00001 -q NonRundant.gene.prot.fasta -a NonRundant.gene.prot.fasta

diamond view -a NonRundant.gene.prot.fasta.daa -o NonRundant.gene.prot.fasta.m8

# for eggnog annotation, the script is same with KEGG

# Each protein was assigned to the KEGG and eggNOG orthologs using the highest scoring annotated hits containing at least one HSP scoring over 60 bits(perl)

use strict;

use warnings;

die"usage:perl $0 <infile> <outfile>\n" unless @ARGV == 2;

my($in,$out)=@ARGV;

open IN,$in;

open OUT,">$out";

my ($line,%check,$check_id);

while (<IN>) {

chomp;

my($id,$gap,$score)=(split/\s+/)[0,5,11];

next if $gap || $score < 60;

if (!$check{$id}) {

$line = $_;

$check{$id}=$score;

print OUT "$line\n" if $check_id && $id ne $check_id;

}else {

if ($score > $check{$id}) {

$line = $_;

$check{$id} = $score;

}

}

$check_id = $id;

}

close IN;

print OUT "$line\n";

close OUT;

`tac $out|sed '2d'|tac > mid;mv mid $out;`;

**#### end ####**

**###### Statistical analysis ######**

**#### begin ####**

**## step1. PCA analysis(R) ##**

library(FactoMineR)

data <- read.table("taxonomy.table.txt", head=T, row.names=1,sep="\t")

data = data[,-dim(data)[2]] #del taxonomy

data = data[-dim(data)[1],] #del others

groups = read.table("group.list", head=F,colClasses=c("character","character"))

colnames(groups) <- c("sample","group")

pca = PCA(data, scale.unit=T, graph=F)

write.csv(pca$ind$coord,file="pca.csv")

**## step2: PCoA analysis(R) ##**

library(“ade4”)

data <- read.table("unweighted.unifrac.distance.matrix")

d <- as.dist(data)

pco <- dudi.pco(d scannf=F, nf=2)

pc1 <- round((pco$eig/sum(pco$eig))*100,2)[1]

pc2 <- round((pco$eig/sum(pco$eig))*100,2)[2] write.csv(pco$li,file="PCoA.csv")

**## step3: Differential analysis(R) ##**

data <- read.table("taxonomy.table.txt",header=T,row.names=1)

data <- data[-dim(data)[1],] #delete others

data <- data[,-dim(data)[2]] #delete detail taxonomy

taxonomy <- rownames(data)

p.value <- matrix(,dim(data)[1],1)

for(i in 1:dim(data)[1]){

p.value[i,1] <- wilcox.test(as.numeric(data[i,1:5]),as.numeric(data[i,6:10]))$p.value #1:5 are group1, 6:10 are group2.

}

data <- cbind(data,p.value) #result for pvalues

sig.pvalue <- data[which(data$p.value < 0.05),]

q.value <- p.adjust(sig.pvalue$p.value,method="fdr", n = length(sig.pvalue$p.value))

new.data <- cbind(sig.pvalue,q.value) #result for qvalues

**## step4: Random forest(R) ##**

library(randomForest)

library("pROC")

trainset <- t(read.table("input.trainset.table.xls",header=F,row.names=1))

testset <- t(read.table("input.testset.table.xls",header=F,row.names=1))

fits <- randomForest(group ~ . , data = trainset, subset = train)

predicts <- predict(fits, testset,probability=T)

pred.prob = attr(predicts, "probabilities")

pred.to.roc = pred.prob[, 2]

pred.rocr = prediction(pred.to.roc, testset$CAG)

perf.rocr = performance(pred.rocr, measure = "auc", x.measure = "cutoff")

perf.tpr.rocr = performance(pred.rocr, "tpr","fpr")

plot(perf.tpr.rocr, colorize=T,main=paste("AUC:",(perf.rocr@y.values)))

**#### end ####**
