# Supplementary material for: Disordered gut microbiota and alterations in metabolic patterns are associated with atrial fibrillation
Source: Gigascience. 2019 May 30;8(6):giz058. doi: 10.1093/gigascience/giz058 (PMC6543127; doi:10.1093/gigascience/giz058)
Supplement: giz058_Supplement_Files [file giz058_supplement_files.zip › Figure S11-r1.pdf]

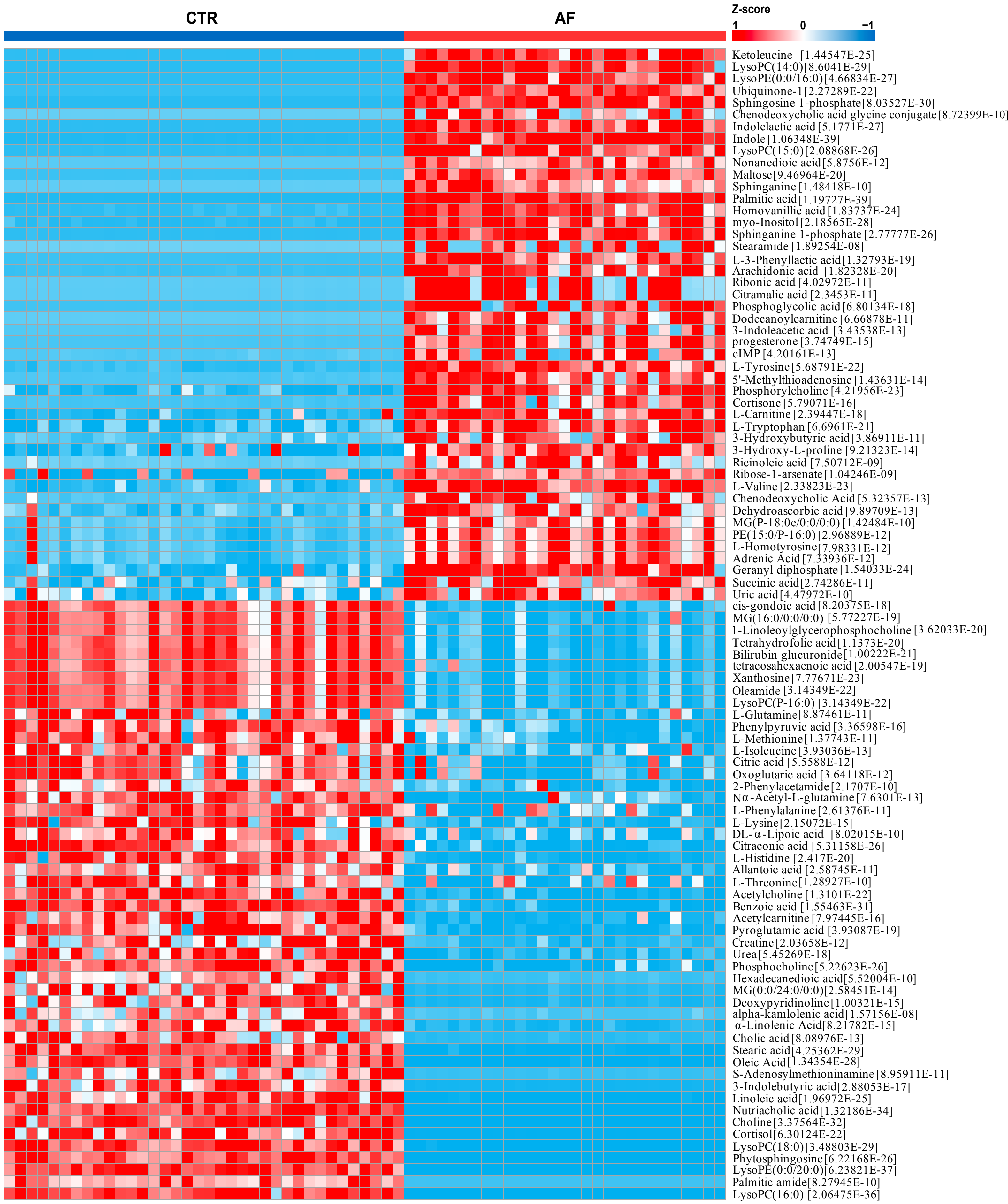

CTR
AF
Z-score
1
0
−1
Ketoleucine [1.44547E-25]
LysoPC(14:0)[8.6041E-29]
LysoPE(0:0/16:0)[4.66834E-27]
Ubiquinone-1[2.27289E-22]
Sphingosine 1-phosphate[8.03527E-30]
Chenodeoxycholic acid glycine conjugate[8.72399E-10]
Indolelactic acid[5.1771E-27]
Indole[1.06348E-39]
LysoPC(15:0)[2.08868E-26]
Nonanedioic acid[5.8756E-12]
Maltose[9.46964E-20]
Sphinganine[1.48418E-10]
Palmitic acid[1.19727E-39]
Homovanillic acid[1.83737E-24]
myo-Inositol[2.18565E-28]
Sphinganine 1-phosphate[2.77777E-26]
Stearamide[1.89254E-08]
L-3-Phenyllactic acid[1.32793E-19]
Arachidonic acid [1.82328E-20]
Ribonic acid[4.02972E-11]
Citramalic acid[2.3453E-11]
Phosphoglycolic acid[6.80134E-18]
Dodecanoylcarnitine[6.66878E-11]
3-Indoleacetic acid [3.43538E-13]
progesterone[3.74749E-15]
cIMP[4.20161E-13]
L-Tyrosine[5.68791E-22]
5'-Methylthioadenosine[1.43631E-14]
Phosphorylcholine[4.21956E-23]
Cortisone[5.79071E-16]
L-Carnitine[2.39447E-18]
L-Tryptophan[6.6961E-21]
3-Hydroxybutyric acid[3.86911E-11]
3-Hydroxy-L-proline[9.21323E-14]
Ricinoleic acid[7.50712E-09]
Ribose-1-arsenate[1.04246E-09]
L-Valine[2.33823E-23]
Chenodeoxycholic Acid[5.32357E-13]
Dehydroascorbic acid[9.89709E-13]
MG(P-18:0e/0:0/0:0)[1.42484E-10]
PE(15:0/P-16:0)[2.96889E-12]
L-Homotyrosine[7.98331E-12]
Adrenic Acid[7.33936E-12]
Geranyl diphosphate[1.54033E-24]
Succinic acid[2.74286E-11]
Uric acid[4.47972E-10]
cis-gondoic acid[8.20375E-18]
MG(16:0/0:0/0:0) [5.77227E-19]
1-Linoleoylglycerophosphocholine[3.62033E-20]
Tetrahydrofolic acid[1.1373E-20]
Bilirubin glucuronide[1.00222E-21]
tetracosahexaenoic acid[2.00547E-19]
Xanthosine[7.77671E-23]
Oleamide[3.14349E-22]
LysoPC(P-16:0) [3.14349E-22]
L-Glutamine[8.87461E-11]
Phenylpyruvic acid[3.36598E-16]
L-Methionine[1.37743E-11]
L-Isoleucine[3.93036E-13]
Citric acid[5.5588E-12]
Oxoglutaric acid[3.64118E-12]
2-Phenylacetamide[2.1707E-10]
Nα-Acetyl-L-glutamine[7.6301E-13]
L-Phenylalanine[2.61376E-11]
L-Lysine[2.15072E-15]
DL-α-Lipoic acid [8.02015E-10]
Citraconic acid[5.31158E-26]
L-Histidine[2.417E-20]
Allantoic acid[2.58745E-11]
L-Threonine[1.28927E-10]
Acetylcholine[1.3101E-22]
Benzoic acid[1.55463E-31]
Acetylcarnitine[7.97445E-16]
Pyroglutamic acid[3.93087E-19]
Creatine[2.03658E-12]
Urea[5.45269E-18]
Phosphocholine[5.22623E-26]
Hexadecanedioic acid[5.52004E-10]
MG(0:0/24:0/0:0)[2.58451E-14]
Deoxypyridinoline[1.00321E-15]
alpha-kamlolenic acid[1.57156E-08]
α-Linolenic Acid[8.21782E-15]
Cholic acid[8.08976E-13]
Stearic acid[4.25362E-29]
Oleic Acid[1.34354E-28]
S-Adenosylmethioninamine[8.95911E-11]
3-Indolebutyric acid[2.88053E-17]
Linoleic acid[1.96972E-25]
Nutriacholic acid[1.32186E-34]
Choline[3.37564E-32]
Cortisol[6.30124E-22]
LysoPC(18:0)[3.48803E-29]
Phytosphingosine[6.22168E-26]
LysoPE(0:0/20:0)[6.23821E-37]
Palmitic amide[8.27945E-10]
LysoPC(16:0) [2.06475E-36]
