# Supplementary material for: Disordered gut microbiota and alterations in metabolic patterns are associated with atrial fibrillation
Source: Gigascience. 2019 May 30;8(6):giz058. doi: 10.1093/gigascience/giz058 (PMC6543127; doi:10.1093/gigascience/giz058)
Supplement: giz058_Supplement_Files [file giz058_supplement_files.zip › Table_S13.docx]

|  | **AF Group** | **Control Group** | **P value** |
| --- | --- | --- | --- |
| **Number** | 29 | 36 | / |
| **Age, years** | 68 (61.5, 74.5) | 54 (49.25, 57.75) | <0.001 |
| **Male/ Female** | 19/10 | 28/8 | 0.276 |
| **BMI** | 26.35 (24.05, 29.65) | 24.79 (22.87, 27.53) | 0.143 |
| **HTN** | 19 | 18 | 0.213 |
| **DM** | 9 | 0 | <0.001 |
| **TC** | 4.29 (3.37, 4.71) | 5.02 (4.48, 5.59) | <0.001 |
| **TG** | 1.76 (1.13, 2.04) | 1.15 (0.90, 1.85) | 0.123 |
| **LDL** | 2.50 (1.55, 2.85) | 2.3 (2.01, 2.81) | 0.882 |
| **FBG** | 4.71(4.44, 5.90) | 5.2 (4.79, 5.56) | 0.610 |
| **Creatinine** | 69.5 (59.6, 82.25) | 68 (59.25, 73) | 0.460 |
| **UA** | 321 (295.5, 394.5) | 333 (240, 406) | 0.686 |
| **TBil** | 14.8 (11.9, 21.45) | 15 (11.9, 21.9) | 0.989 |
| **ALT** | 19 (13, 30.5) | 19 (12, 25.75) | 0.315 |

Table S13. Baseline characteristics of serum samples from metabolomic analyses.

Abbreviations: AF, atrial fibrillation; BMI, body mass index; HTN, hypertension; DM, diabetes mellitus; CHD, coronary heart disease; TC, total cholesterol; TG, triglyceride; LDL, low density lipoprotein; FBG, fasting blood glucose; UA, uric acid; TBil, total bilirubin; ALT, glutamic-pyruvic transaminase. IQR, interquartile range; Data are presented as mean± SD, or median (IQR), as appropriate.
