# Supplementary material for: Disordered gut microbiota and alterations in metabolic patterns are associated with atrial fibrillation
Source: Gigascience. 2019 May 30;8(6):giz058. doi: 10.1093/gigascience/giz058 (PMC6543127; doi:10.1093/gigascience/giz058)
Supplement: giz058_Supplement_Files [file giz058_supplement_files.zip › Table_S14.docx]

|  | **AF Group** | **Control Group** | **P value** |
| --- | --- | --- | --- |
| **Number** | 42 | 17 | / |
| **Age, years** | 67.50 (56.75, 72.25) | 52 (50.5, 58.5) | <0.001 |
| **Male/ Female** | 28/14 | 14/3 | 0.232 |
| **BMI** | 26.09 (23.37, 27.85) | 24.77 (22.92, 27.66) | 0.536 |
| **HTN** | 25 | 9 | 0.646 |
| **DM** | 12 | 0 | 0.014 |
| **TC** | 4.12 (3.40, 4.65) | 4.68 (4.31, 5.39) | 0.007 |
| **TG** | 1.29 (1.02, 1.92) | 1.05 (0.65, 1.80) | 0.103 |
| **LDL** | 2.40 (1.50, 2.83) | 2.2 (2.02, 2.58) | 0.663 |
| **FBG** | 4.97 (4.50, 5.83) | 4.96 (4.46, 5.44) | 0.734 |
| **Creatinine** | 69.85 (60.35, 84.88) | 63 (59, 70) | 0.052 |
| **UA** | 321.5 (277.75, 389.75) | 355 (318.75, 400.75) | 0.178 |
| **TBil** | 14.35 (11.00, 19.55) | 17.6 (13.8, 22.3) | 0.121 |
| **ALT** | 19.5 (15.50, 28.50) | 19.5 (13.5, 24.5) | 0.291 |

Table S14. Baseline characteristics of feces samples from metabolomic analyses.

Abbreviations: AF, atrial fibrillation; BMI, body mass index; HTN, hypertension; DM, diabetes mellitus; CHD, coronary heart disease; TC, total cholesterol; TG, triglyceride; LDL, low density lipoprotein; FBG, fasting blood glucose; UA, uric acid; TBil, total bilirubin; ALT, glutamic-pyruvic transaminase. IQR, interquartile range; Data are presented as mean± SD, or median (IQR), as appropriate.
