# Supplementary figures and images for: Disordered gut microbiota and alterations in metabolic patterns are associated with atrial fibrillation
Source: Gigascience. 2019 May 30;8(6):giz058. doi: 10.1093/gigascience/giz058 (PMC6543127; doi:10.1093/gigascience/giz058)

**a**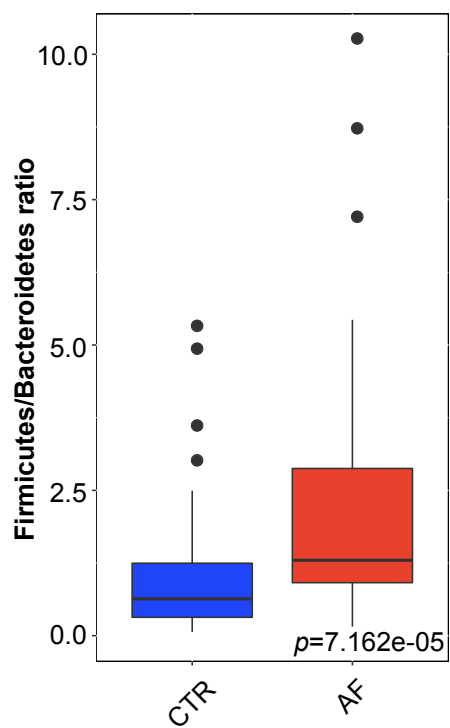**b**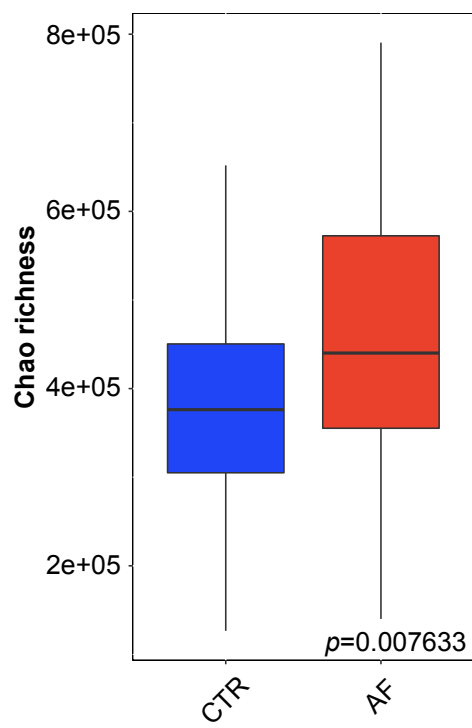**c**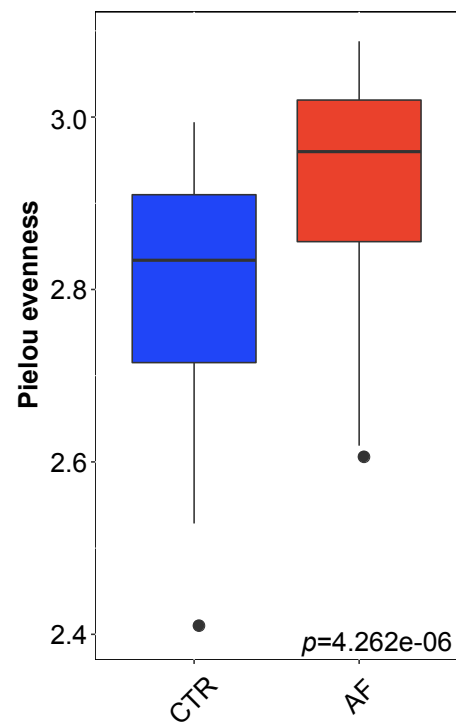

Supplement: giz058_Supplement_Files [file giz058_supplement_files.zip › Figure S1-r1.pdf]

a

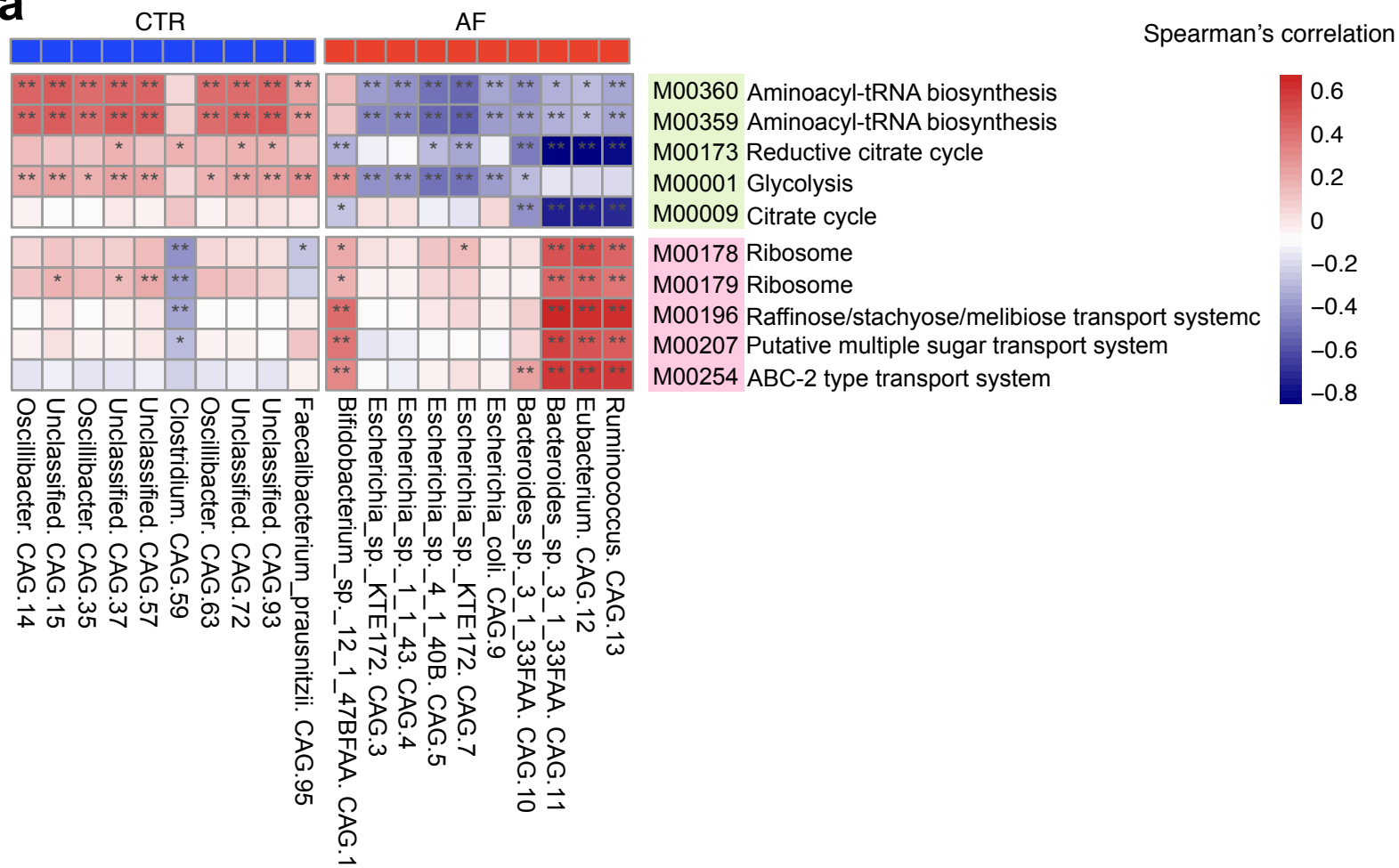

b

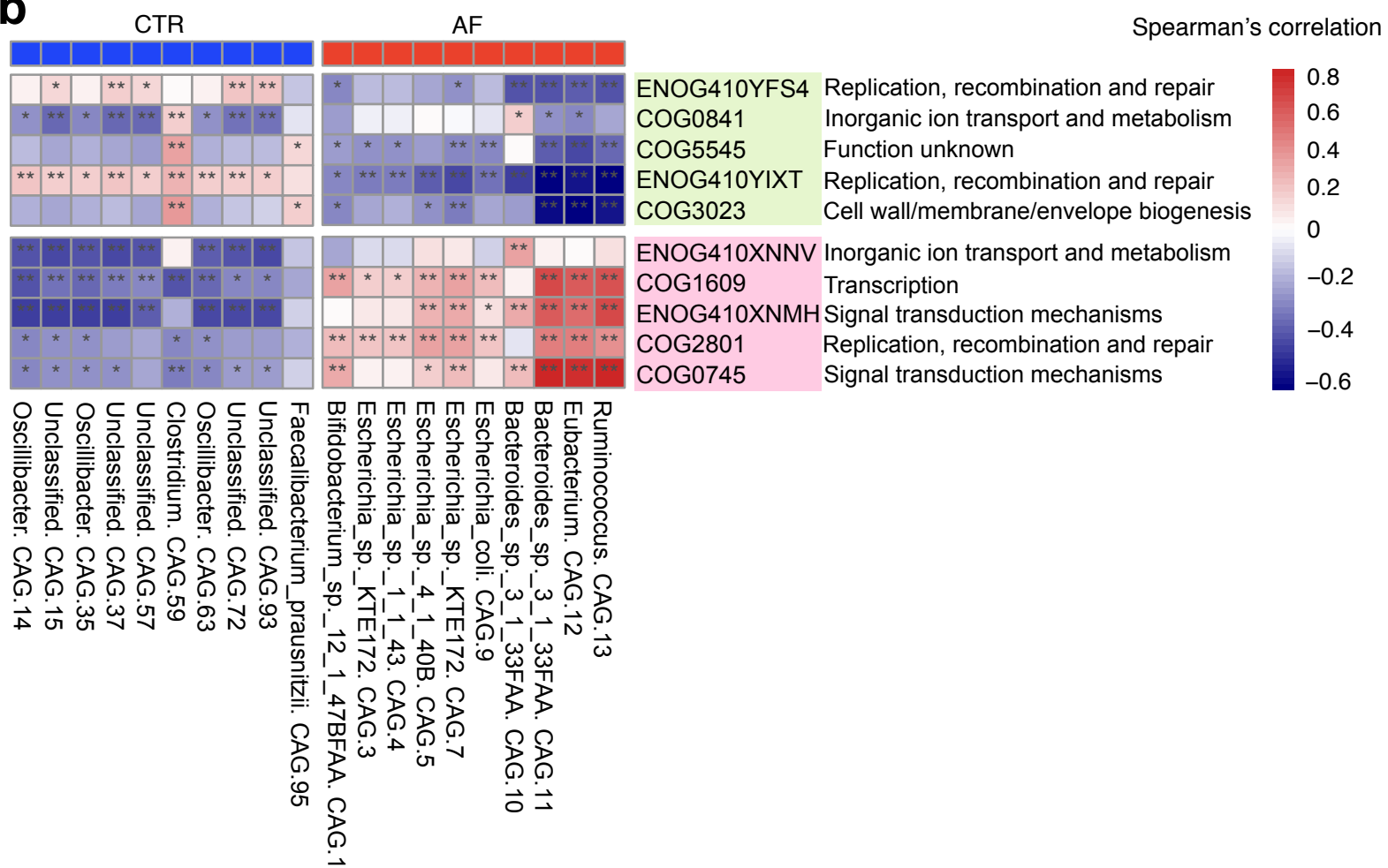

Supplement: giz058_Supplement_Files [file giz058_supplement_files.zip › Figure S10-r1.pdf]

CTR

AF

Z-score

1

0

-1

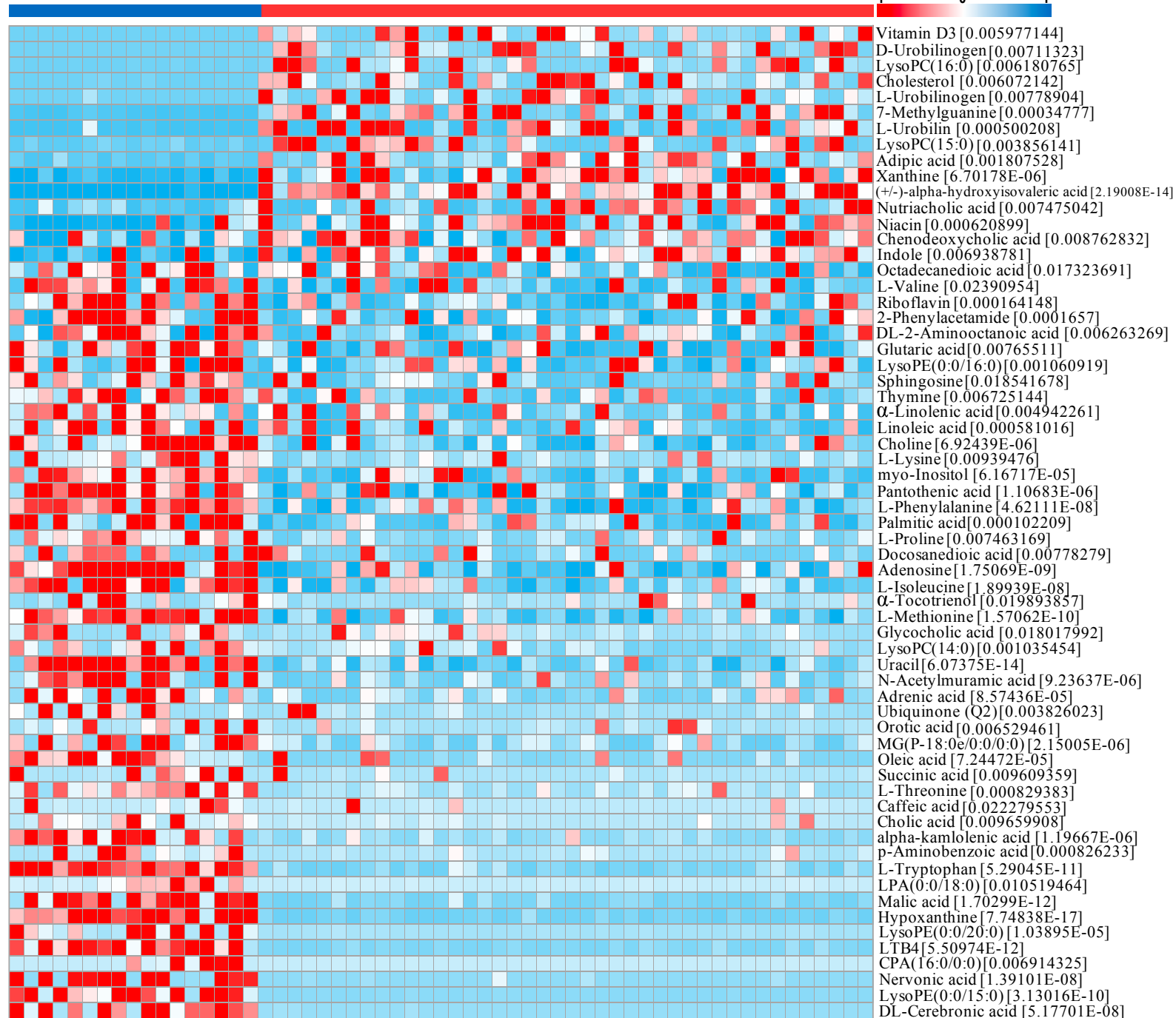

Supplement: giz058_Supplement_Files [file giz058_supplement_files.zip › Figure S12-r1.pdf]

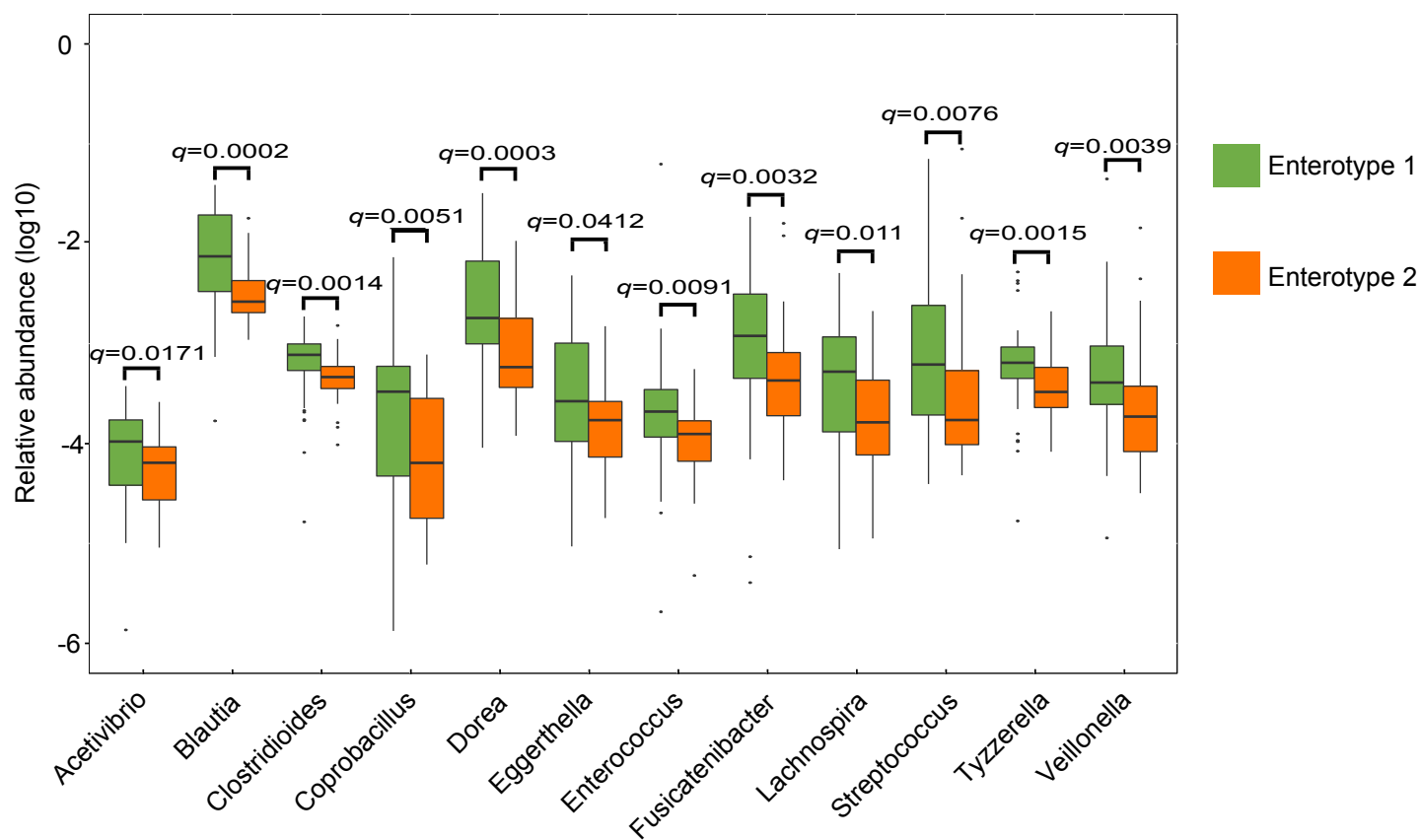

Supplement: giz058_Supplement_Files [file giz058_supplement_files.zip › Figure S2-r1.pdf]

**a**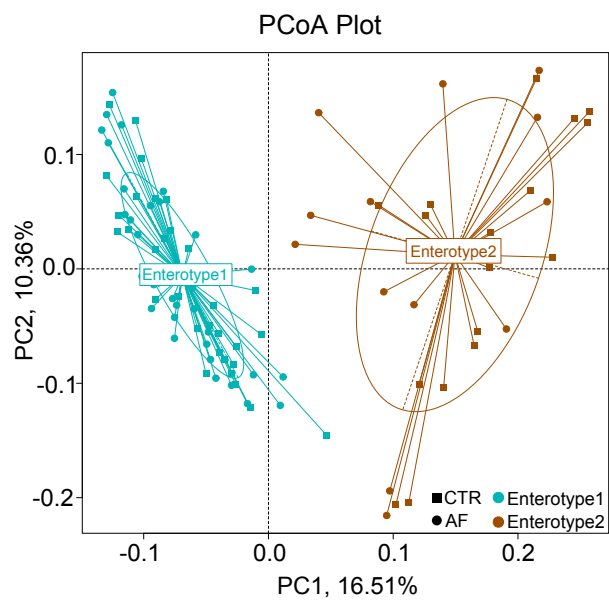**b**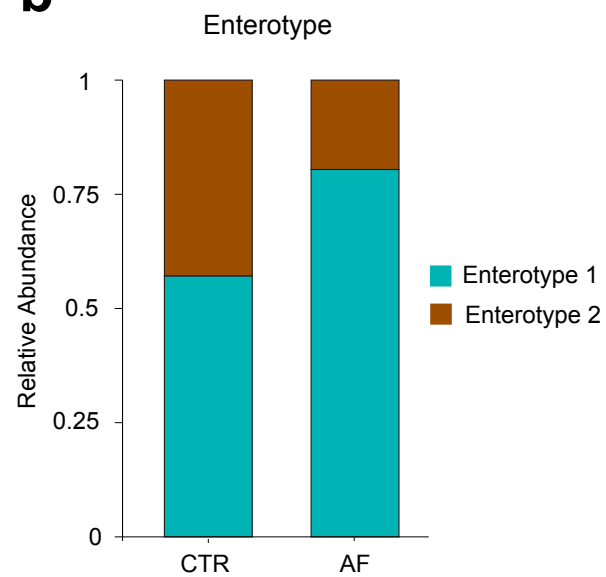

Supplement: giz058_Supplement_Files [file giz058_supplement_files.zip › Figure S3-r1.pdf]

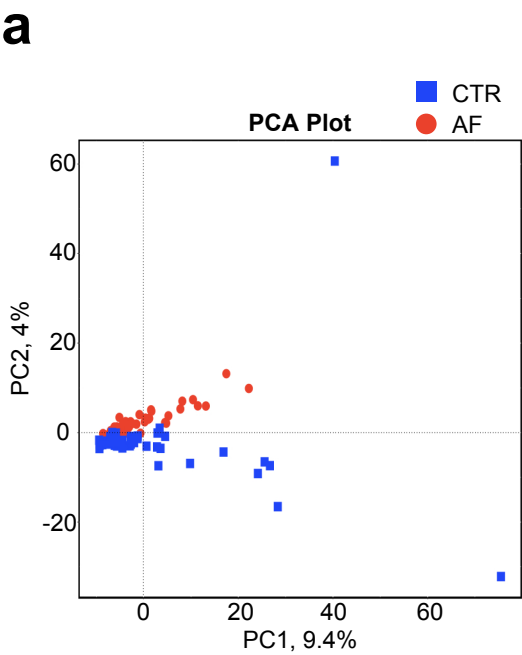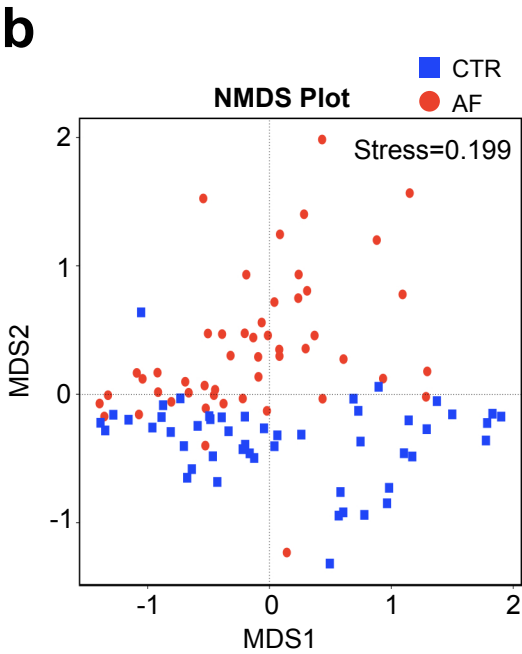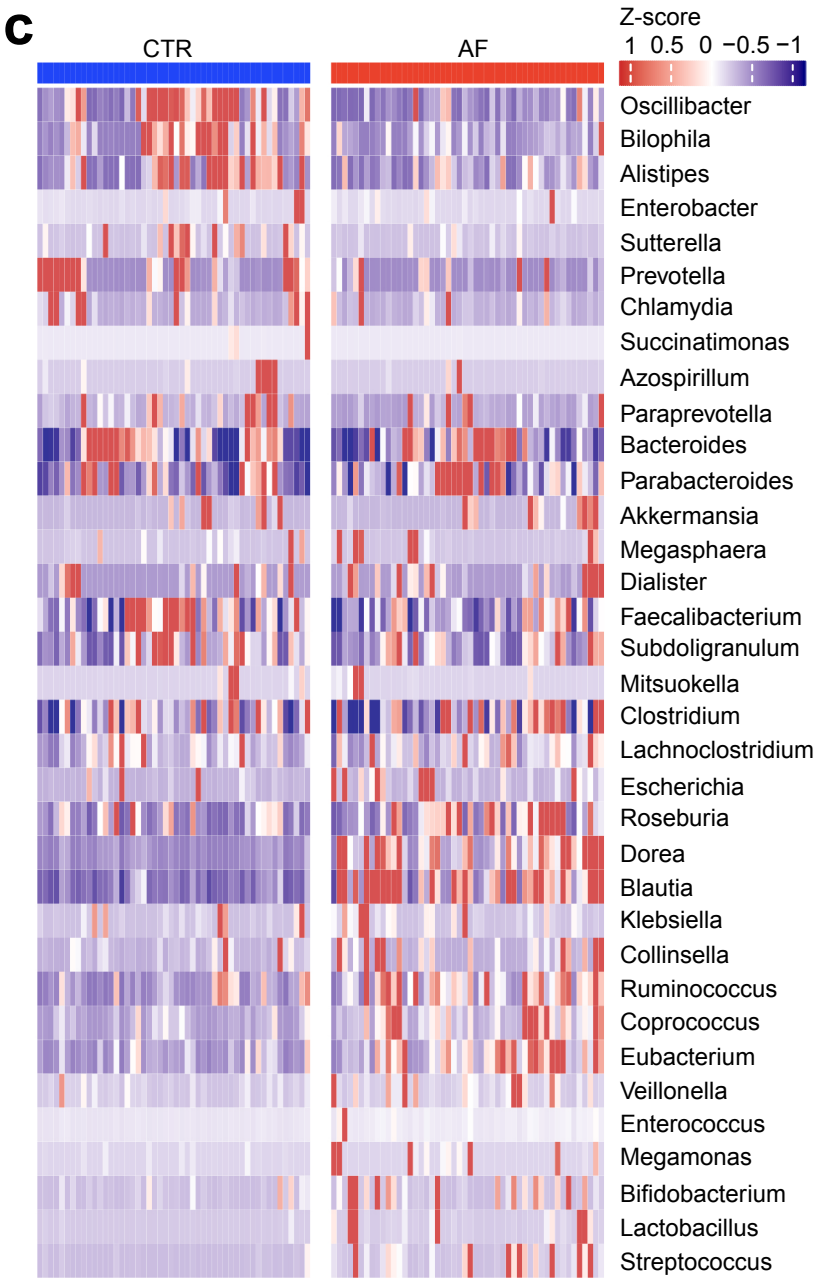

Supplement: giz058_Supplement_Files [file giz058_supplement_files.zip › Figure S4-o.pdf]

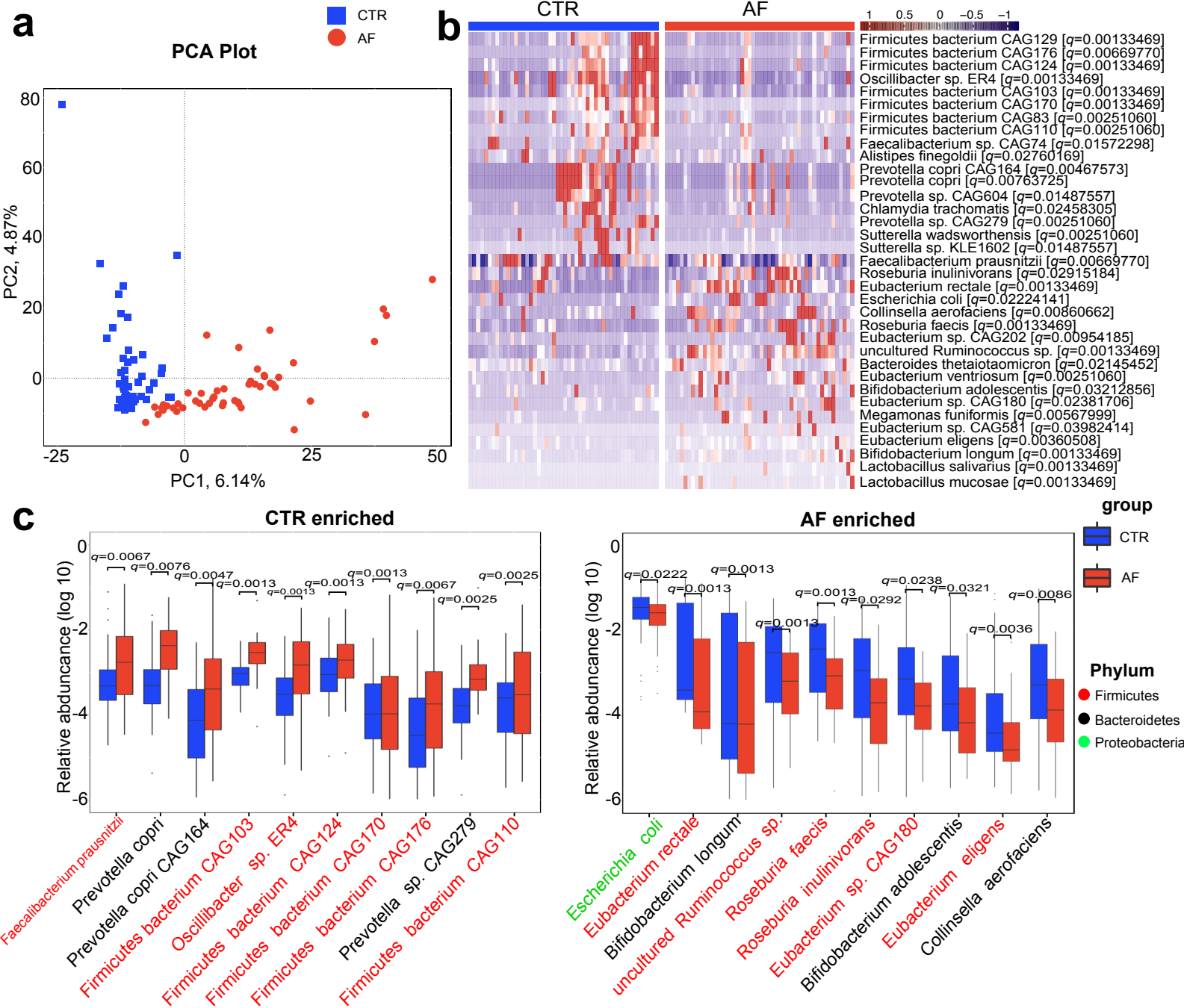

Supplement: giz058_Supplement_Files [file giz058_supplement_files.zip › Figure S5-r1.pdf]

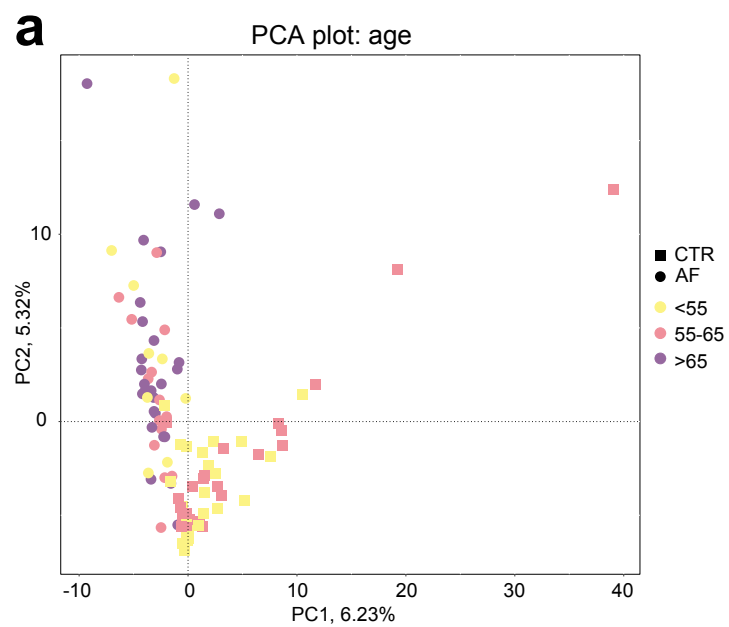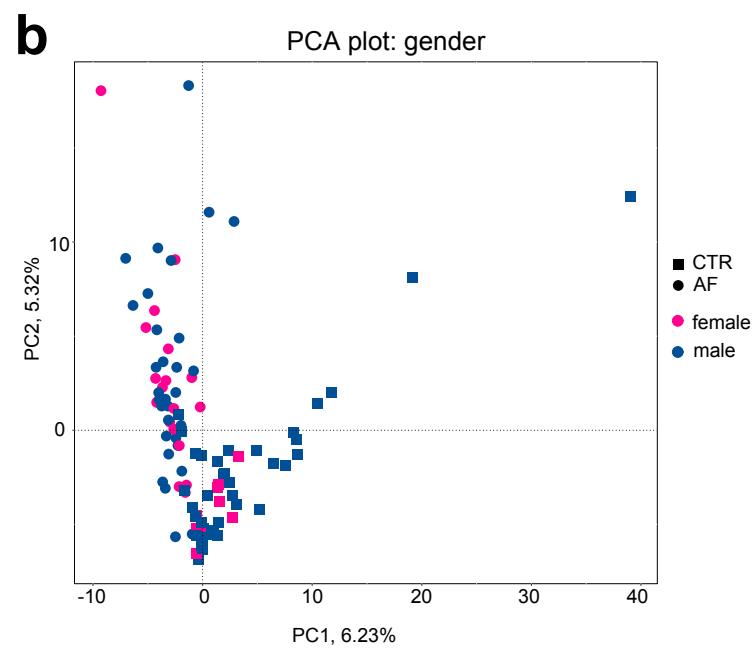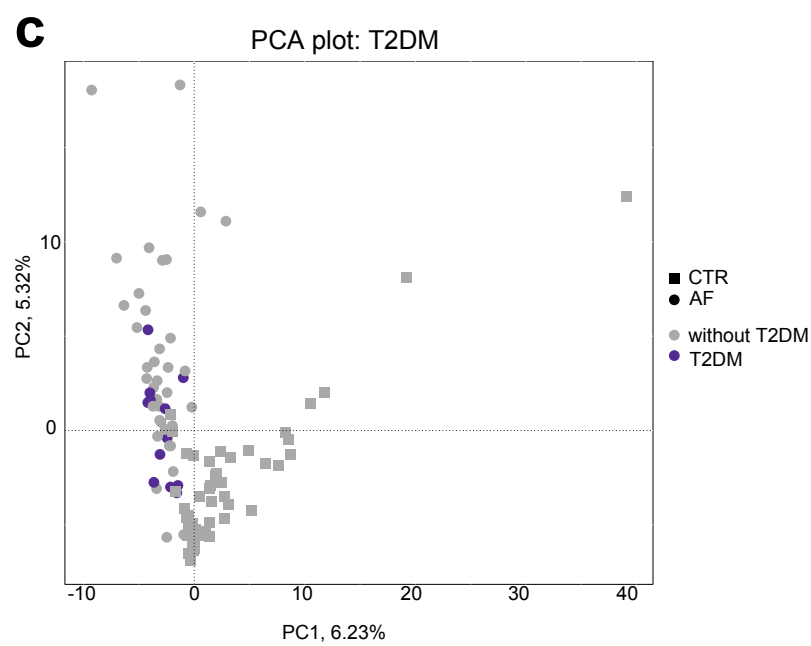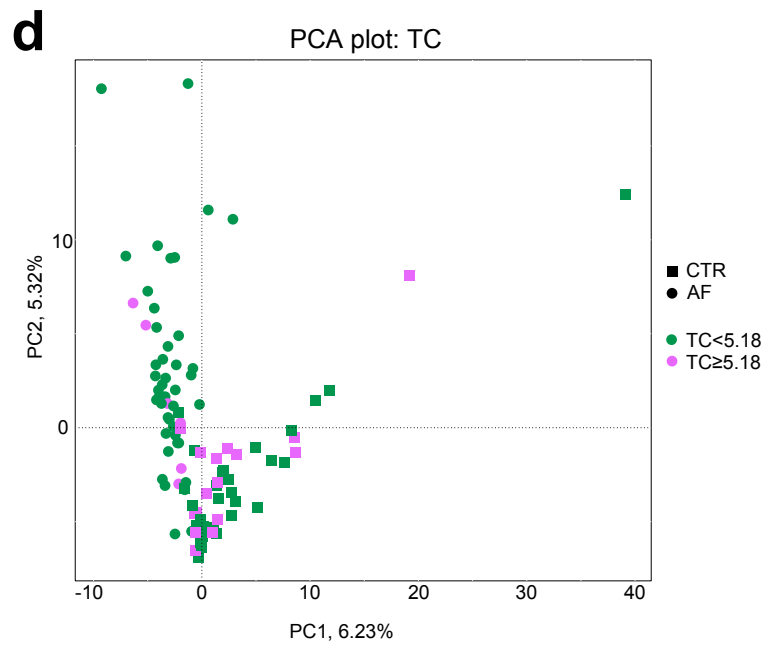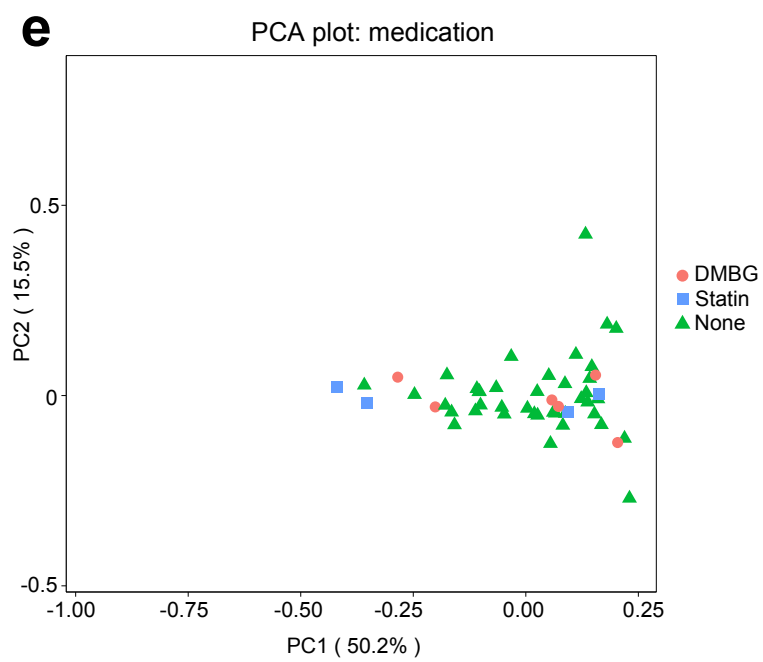

Supplement: giz058_Supplement_Files [file giz058_supplement_files.zip › Figure S6-r1.pdf]

**a**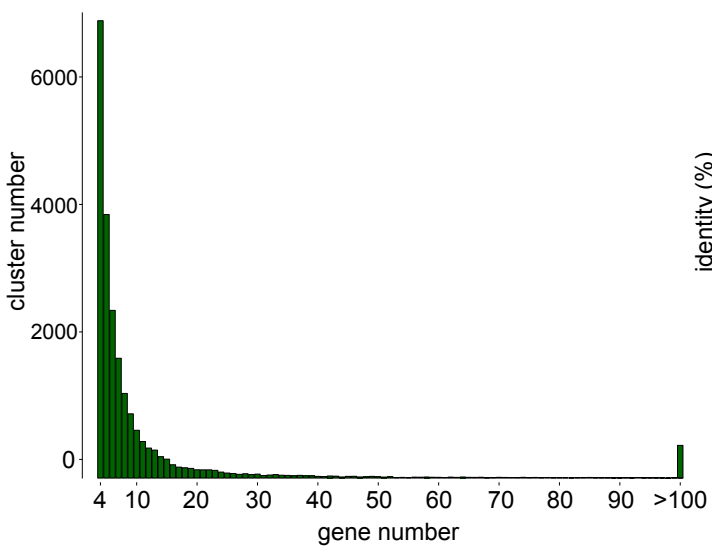**b**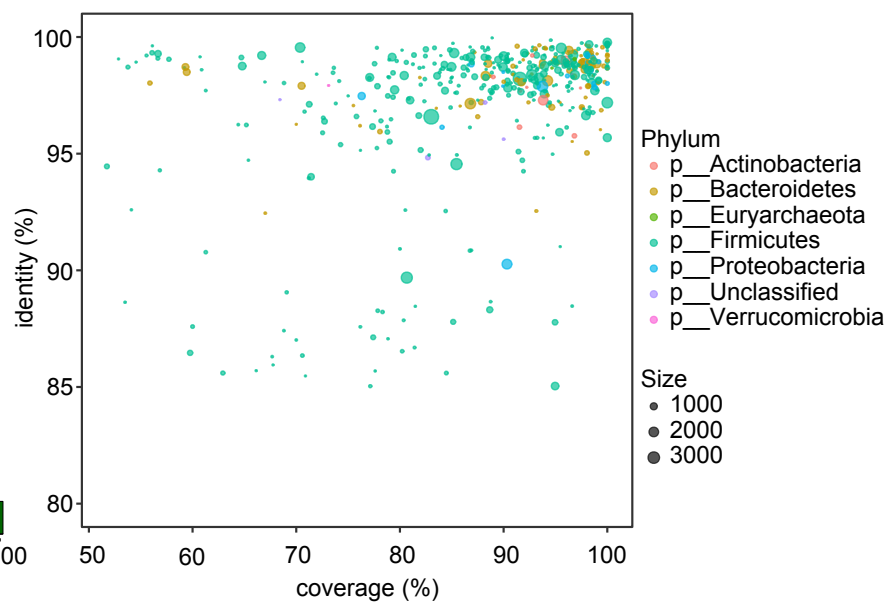

Supplement: giz058_Supplement_Files [file giz058_supplement_files.zip › Figure S7-r1.pdf]

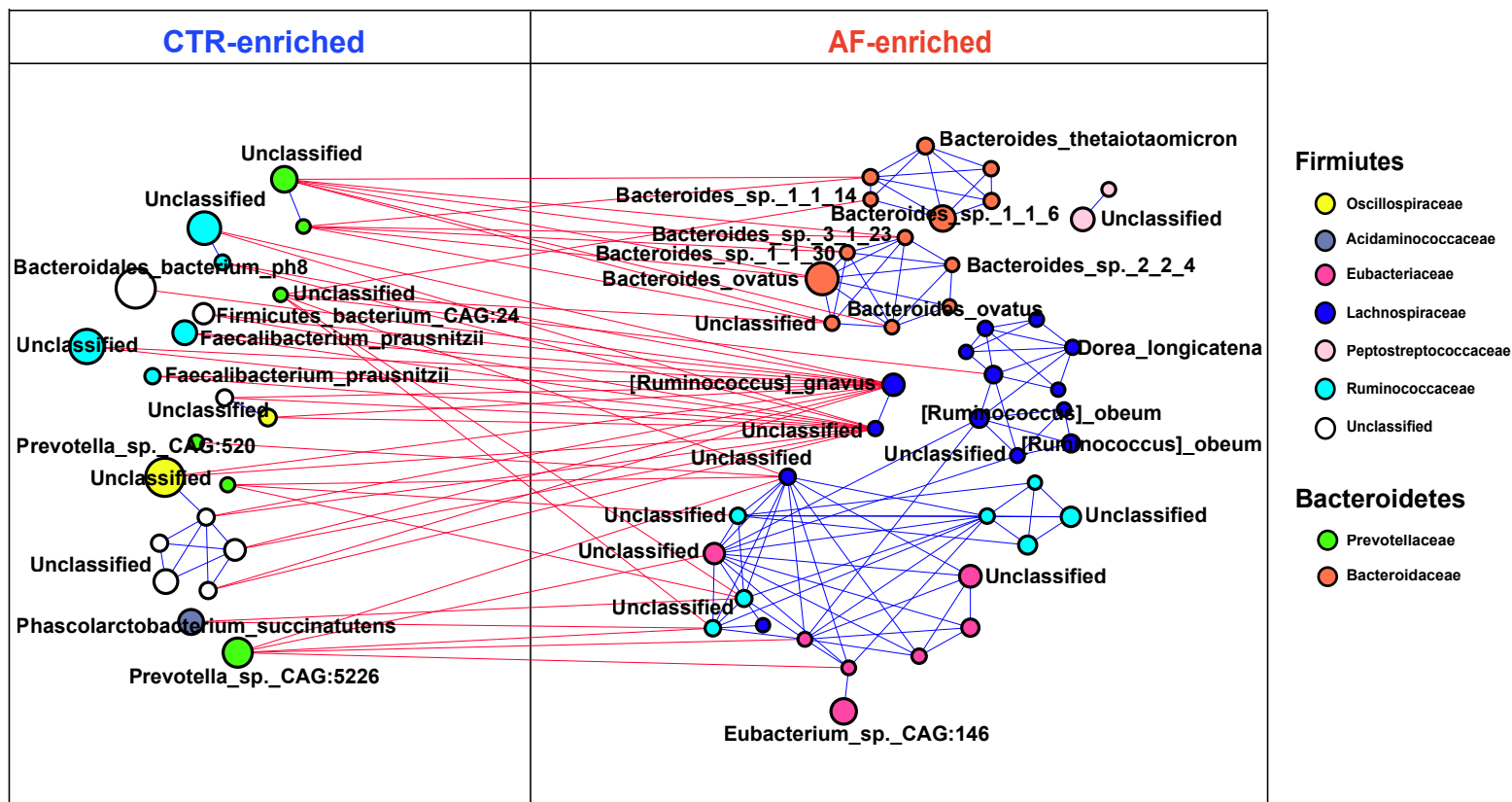

Supplement: giz058_Supplement_Files [file giz058_supplement_files.zip › Figure S8-r1.pdf]

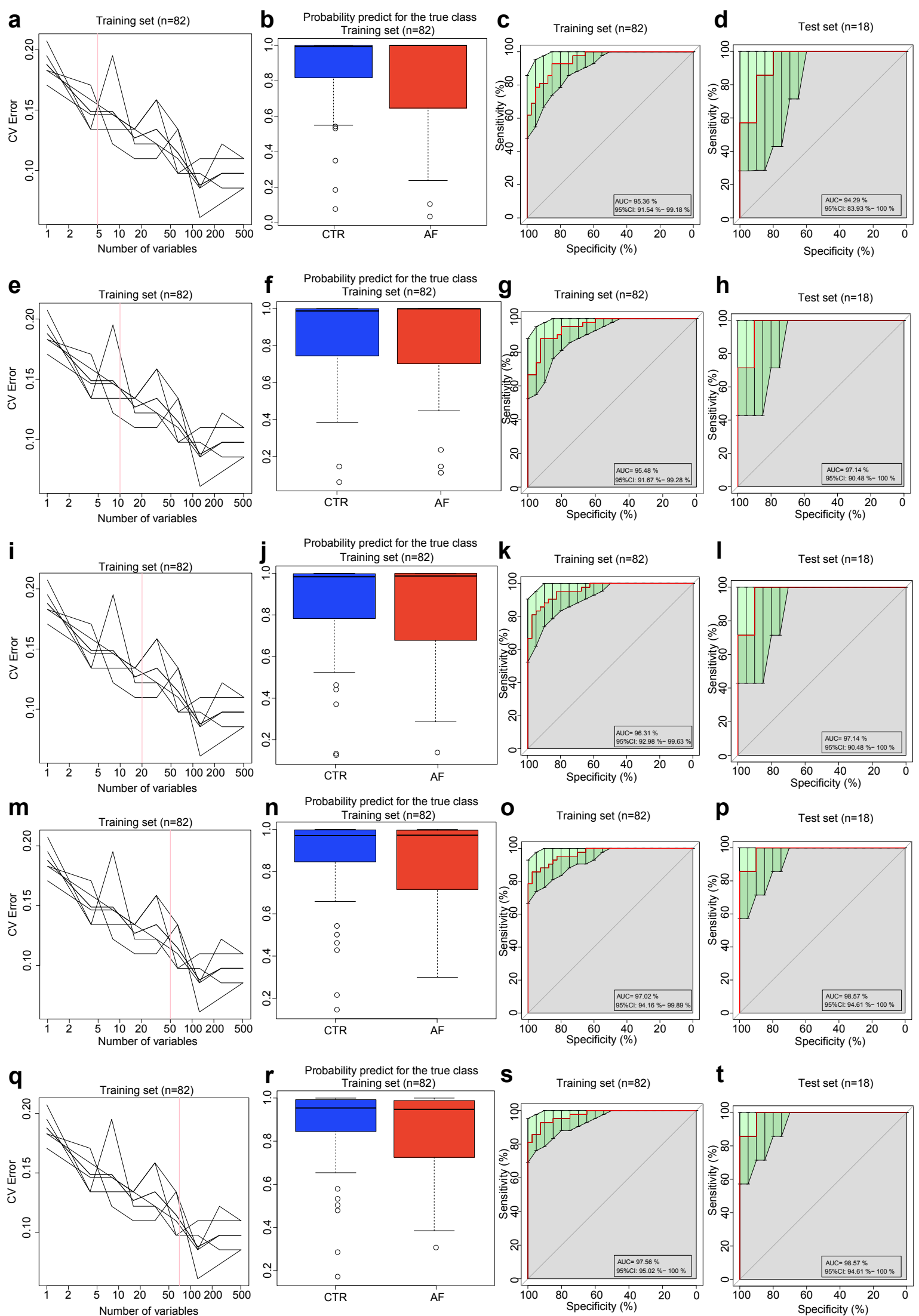

Supplement: giz058_Supplement_Files [file giz058_supplement_files.zip › Figure S9-o.pdf]
