# Supplementary material for: Disordered gut microbiota and alterations in metabolic patterns are associated with atrial fibrillation
Source: Gigascience. 2019 May 30;8(6):giz058. doi: 10.1093/gigascience/giz058 (PMC6543127; doi:10.1093/gigascience/giz058)
Supplement: giz058_Response_to_Reviewer_Comments_Original_Submission [file giz058_response_to_reviewer_comments_original_submission.pdf]

Author's Response To Reviewer Comments

Close

Dear editor,

Thank you very much for your consideration and encouragement on our manuscript entitled "Disordered gut microbiota and alterations in metabolic patterns are associated with atrial fibrillation" (GIGA-D-18-00364). We thank reviewers for their professional and positive comments to improve our manuscript. We have considered all the comments from the reviewers and revised the manuscript. In addition, we have done several experiments as suggested by the reviewers which strengthen our conclusions. Furthermore, we re-performed the metabolomics analysis with larger sample size, and the raw metabolomics MS data has been uploaded to Metabolomics Workbench. However, the state is still at “response under review” (DataTrack ID: 1593). We will continue tracking the data uploading process and make the metabolomics data openly as soon as possible. The RRID number has been included in the manuscript. And the computational code of step by step for bioinformatic analysis was provided as additional files 28.

Point-by-point replies are listed as below.
A list of the changes in Figures
1. Figure 1b, c, e, f was updated with report of p value.
Figure 1d was updated with different shape of dot among AF and controls.
2. Figure S1 was updated with report of p value in boxplot.
3. Figure S2 was updated with report of p value in boxplot.
4. A new figure S3 was supplemented to show the enterotype analysis in species level.
5. Figure S3 in previous version was changed to figureS4.
6. Figure 2 b, c, d was updated with report of q value in heatmap and box plot. Furthermore, we combined figure 2c and figure 2d in a new figure 2c.
7. Figure S4 in previous version was changed to figureS5 and updated with report of q value in heatmap and box plot. And figure S5c and figure S5d were combined to figure S4c.
8. Figure S5 in the previous version was deleted, and figure S6 in the previous version was moved to figureS6e. New figure S6 a-d were added.
9. A new figure S7 was added.
9. Figure 3 in the previous version was changed to figure S8.
10. Figure 4 in the previous version was changed to figure 3 and label of Y-axis in figure 3a, b was updated with clear description.
11. Figure S7 in the previous version was changed to figure S9.
12. Figure 5 in the previous version was changed to figure 4, and figure 4c, f was updated with display of q value in heatmap
13. A new figure S10 was added to show the correlation between significant different CAGs and KEGG module and eggNOG in heatmap.
14. Figure 6 in the previous version was changed to figure 5 and was fully updated, because the metabolism analysis was re-performed in a larger sample size.
15. Figure S8 in the previous version was changed to figure S10 and fully updated as the metabolism analysis was re-performed in a larger sample size.
16. Figure S9 in the previous version was changed to figure S11 and fully updated as the metabolism analysis was re-performed in a larger sample size.
17. Figure 7 in the previous version was changed to figure 6 and was fully updated for the new metabolism analysis. Figure 6d was added, where this correlation analysis was performed in species level.
18. Figure legends has been updated according to the change of figures.

A list of the changes in Tables
1. Table 1 was updated with newly description about medication.
2. Table S13-S15 was updated because of the re-performed metabonomics with larger sample size.

Respond to Reviewer #1:

Zuo et al present a manuscript titled "Disordered gut microbiota and alterations in metabolic patterns are associated with atrial fibrillation". The manuscript describes the study of 100 subjects (50 AF vs 50 control) using shot gun meta genomic and metabolomic analysis of fecal and serum to study the impact of microbiome on the Atrial Fibrillation disease. The study generated a lot of data. However, I found many points that need to be improved and clarified as below.

Question: 1) The authors stated that "To adjust for the effect of HTN on gut microbiota composition, we selected 50 samples from our previous gut microbiota work matched for a history." There is no reference or details to following this statement.

Response: Reference about "our previous gut microbiota work" has been added (Li, J., et al. Microbiome. 2017; 5: 14.), which described the dysbiotic gut microbiota in hypertension patients. And 50 individuals from this previous work were selected as controls and their metagenomic data were utilized in the current study (page 8, #line 215 in the revision; page 8, #line 154 in the clean revision).

Question: 2) Medications is a key factor that can alter gut microbiome as shown in previous study (e.g. https://www.nature.com/articles/nm.4345). There is no report of control medication? I recommend the author report medication in a systematic way in a table because it is very important information.

Response: Following the reviewer's suggestion, we have updated table 1 with the information of medication and added this part (Wu, H., et al. Nat Med. 2017; 23: 850-858.) in the revised version (page 14, #line 389 in the revision; page 14, #line 269 in the clean revision). There were 4 AF patients taking statins and 6 taking DMBG. As shown in Fig. S6e, the effects of statins and DMBG usage were analyzed by PCA plots to assess the possible influence of drug consumption on GM in AF patients.

Question: 3) I recommend the author make a better figure 2d to visualize the higher percentage AF patients in enterotype 1 by changing the dot types in different shape. This will make the reader to catch the claim up from the figure.

Response: We have revised figure 1d with AF patients in shape of circle and controls in block.

Question: 4) The authors reported the statistical different of genera between AF and control in FigS3 and S4. I recommend the author to put statistical values details in the figures.

Response: We have added the details of statistical values (q value) in heatmap and boxplot, including figure 1, 2, 4, and figure S1-2, S5, S11-12 in the revised version. We could not add q value in figure S3c in previous version (figure S4 in the revised version), because this heatmap showed the top 35 most abundant genera in AF and control group without comparison between two groups.

Question: 5) The author reported the small influence of T2DM and TC on the gut microbiota change using PLS-SEM as shown in figure S5. However, the most different phenotype is age as show in the table 1. I strongly recommend the authors perform the analysis to see the effect of age, gender and HTN confounders. This is very important issue for the conclusion of the paper.

Response: Thanks for this suggestion. We did not exclude AF patients with HTN or T2DM as mentioned in the limitation part, while HTN, T2DM, cholesterol and aging were all strong risk factor in the development of AF. And these risk factors involved in the process of oxidative stress, endothelial dysfunction and atrial remodeling, while formed the risky mutual relationships.
As described in table 1, HTN has been matched individually yet, and there were 27 participants diagnosed with HTN in both AF group and control group, so the influence of HTN factor to our result was eliminated.
The aim of building PLS-SEM model was to test if there was a mediation effect (indirect effect) of confounders during the GM shifts observed in AF. However, GM cannot influence age or gender during AF development. So, it seemed that PLS-SEM model was not appropriate to analyze the confounding effect of age and gender in AF.
Considering the difference of gender, age, TC and T2DM, we performed PCA plot to assess the contribution of different baseline characteristics and found that PCA failed to distinguished AF patients into separated group based on these factors, indicating the negligible impact of gender, age, TC or T2DM

on our data (p>0.05, Anosim, Additional files 10, figure S6) (page 13, #line 361 in the revision; page 13, #line 261 in the clean revision).

Question: 6) Figure 3 is meaning less I suggest to put in supplementary. Taxonomic annotation of CAG is also unclear for me in M&M. The author need to provide a better description to assign taxonomic of CAG. What is "the taxonomy of tracer genes", no reference about this ?? Author need to provide the confident of taxonomic annotation confidence of individual CAG. Moreover, distribution of CAG size (number of genes) should we plot to see. I think 50 gene cut-off for CAG as mentioned in M&M is proper. It is too small !

Response: Figure 3 has been moved to supplementary data in figure S8. Detailed description about the process of taxonomic annotation of CAG has been supplemented in methods (page 31, #line 838 in the revision; page 31, #line 609 in the clean revision) and described as followed: "All genes from one CAG were aligned to the reference microbial genomes at the nucleotide level (by BLASTN) and the NCBI-nr database at the protein level (by BLASTP). The alignment hits were filtered by both thee-value (< 1×10-5at the nucleotide level and < 1×10-5at the protein level) and the alignment coverage (>70% of a query sequence). From the alignments with the reference microbial genomes, we obtained a list of well-mapped bacterial genomes for each CAG and ordered these bacterial genomes according to the proportion of genes that could be mapped onto the bacterial genome, as well as the average identity of the alignments."
And the detailed information of CAG has been shown in supplementary tables, including table S6 (Reference genomes for CAG's taxonomy assignment), table S7 (Detailed information of enriched CAGs in different groups), table S8 (Detailed information of 477 CAGs), and table S9 (Spearman's correlation between enriched CAGs).
Tracer genes were all the genes clustered in a CAG and reference about tracer genes was added (Qin J, et al. Nature. 2012; 490:55–60.) (page 31, #line 838 in the revision; page 31, #line 609 in the clean revision).
The confident of taxonomic annotation confidence of individual CAG and distribution of CAG size (number of genes) was added as figure S7. And 477 CAGs were assigned to known bacterial genera based on the tracer genes, with at least 80% of the genes mapped to the reference genome at an identity higher than 85% (page 14, #line 402 in the revision; page 14, #line 282 in the clean revision).
About "50 gene cut-off for CAG", we have included 2 references (Li, J., et al. Microbiome. 2017; 5: 14. Qin, N., et al. Nature. 2014; 513: 59-64. Nielsen, H. B., et al. Nat Biotechnol. 2014; 32: 822-828.), where CAGs were defined as clusters with more than 50 genes (page 30, #line 835 in the revision; page 30, #line 606 in the clean revision).

Question: 7) What is OR score ??? no full term mentioned in the manuscript.

Response: We used an odds ratio (OR) test to measure the fold change in the abundance of CAGs and reference about OR score was added (Greenblum, S., et al. Proc Natl AcadSci USA. 2012; 109: 594-599.) (page 31, #line 852 in the revision; page 31, #line 623 in the clean revision).

Question: 8) Figure 4 legend need to be improved, not all components, and details were explained clearly.

Response: Figure 4 in the previous version was changed to figure 3 and legend of figure 3 (page 48, #line 1249 in the revision; page 48, #line 916 in the clean revision) has been revised as following.
a. The random forest disease classifier. The model was trained using relative abundance of the CAGs in the controls and AF samples as variables. In training set (n=82), distribution of 5 trials of 10-fold cross-validation (CV) error in random forest classification of AF as the number of CAGs increased. The red line marked the number of CAGs in the optimal set with the lowest cross-validation error.
b. Box-and-whisker plot for the probability of AF in the cross-validation training set according to the model in a. Either control or AF group showed a high probability for predicting the true class in training set (n=82).
c. Receiver operating curve (ROC) for the training set (n=82). The area under receiver operating curve (AUC) is 97.74% and the green area indicated 95% CI: 95.27%-100%.
d. The top 30 different CAGs distinguish AF from control based on the random forest model using explanatory variables of CAGs.
e. ROC for the test set (n=18). The AUC is 98.57% and the green area indicated 95% CI: 94.61%-

100%.

Question: 9) The result of CAG and KEGG and eggNOG function should be coordinated. The author reported them separately. For example, the gene that significant different is and KEGG and eggnog analysis is associate with the high score CAG?

Response: Thanks for this suggestion. We have performed a correlation analyses between CAGs and KEGG modules, or CAGs and eggNOGs, which was shown as a heatmap in figure S10. We found CTR enriched CAGs positively correlated with some basic functions necessary for life-sustaining activities such as aminoacyl-tRNA biosynthesis and citrate cycle. (page 17, #line 477 in the revision; page 17, #line 336 in the clean revision).

Question: 10) Statistical values should report in the Figure 5

Response: Figure 5 in the previous version was changed to figure 4 and q value was added in heatmap.

Question: 11) The author then measures metabolites in fecal, serum using LC-MS in positive and negative mode. It is not clear in M&M how the author handle and analyze data such as normalization method, how many features is detected and use in the analysis.

Response: Considering the remarkable differences existed among various metabolites, some signals of metabolites with too high or low concentration might be covered up and failed to be identified as biomarkers. So, normalization, aiming to adjust the weight of different variables to decrease the gap of different signals, should be performed to make the dimension (for example mean and standard deviation) of all variables in a similar level and make the data more comparable. The calculation process was to normalize the peak area of each sample to 1000000 and divide the peak area of each ion by the total peak area of the sample and multiplied by 1000000 (page 33, #line 889 in the revision; page 33, #line 660 in the clean revision).
And for features, in serum, 2548 features at (ESI+) ion mode and 1733 features at (ESI-) ion mode were detected. And for feces, 2547 features at (ESI+) ion mode and 1894 features at (ESI-) ion mode were tested in this experiment (page 18, #line 494 in the revision; page 18, #line 351 in the clean revision).

Question: 12) How to identify compound name-confident score need to be report. It is very critical information for conclusion. Most of the time people interpret wrong so confident values of compound in dentification (name) is needed.

Response: The exact molecular mass and ms/ms value of these compounds was used to identify the metabolites related to the featured peak in the Metlin database (http://metlin.scripps.edu). Furthermore, the mass compactum of each metabolites would be compared either. And the confident score indicated the matching rate was calculated by Compound Discoverer 2.0 software (Thermo) with max of 100 and has been shown in supplementary table S15 (page 34, #line 927 in the revision; page 34, #line 673 in the clean revision).

Question: 13) The overlap compound in figure 7 is based on names or chromatogram and mass spectra. It is very important to check that they have similar MS pattern because, as I mentioned before on the issue of compound identification is not well report.

Response: Thanks for this valuable suggestion. The overlap compound in figure 7 (figure 6 in revised version) was based on molecular mass and ms/ms value. As mentioned in previous question, the exact molecular mass and ms/ms value of these compounds was used to identify the metabolites related to the featured peak in the Metlin database (http://metlin.scripps.edu) and the mass compactum was compared.

Question: 14) For reproducibility of research especially data analysis, step by step for bioinformatic analysis as computational note book need to be provide for transparency as example in this paper

https://www.ncbi.nlm.nih.gov/pmc/articles/PMC4940747/. I am very serious about this.

Response: The computational code of step by step for bioinformatic analysis was provided as additional files 28.

Question: 15) I cannot get any supplementary tables at all.

Response: All supplementary tables numbered from table S1 to table S15 has been uploaded yet and could be seen in “Manuscript Draft” which was automatically produced by GigaScience.

Question: 16) No information on data deposition for shotgun sequencing and metabolome. It is very essential.

Response: Table S1 showed data production of 100 samples in control and AF and table S15 showed the detailed information of 27 metabolites differently enriched across groups. And the data set supporting the results of this article has been deposited in the EMBL European Nucleotide Archive (ENA) under BioProject accession code PRJEB28384 [http://www.ebi.ac.uk/ena/data/view/PRJEB28384]. And we re-performed the metabolomics analysis with larger sample size, and the raw metabolomics MS data has been uploaded to Metabolomics Workbench. However, the state is still at “response under review” (DataTrack ID: 1593). We will continue tracking the data uploading process and make the metabolomics data openly as soon as possible.

Question: 17) In general, all figure legends need to be improved

Response: All figure legends has been revised with detailed descriptions and important statistical values (page 41, #line 1069 in the revision; page 41, #line 785 in the clean revision).

Question: 18) It would be fantastic if the study has dietary information of the subject.

Response: Thanks for this valuable suggestion. As mentioned in the limitation part (page 26, #line 745 in the revision; page 26, #line 520 in the clean revision), dietary habit was associated with altered gut microbial composition. However, the detail of dietary was very complicated. The proportion of different kind of food, fibers, proteins, carbohydrates and fat were highly variable and daily intake in a same person was also indeterminate, which might be influenced by numerous uncontrolled factors. So, we are so sorry about failing to gather and quantify the dietary information of the subject.

Respond to Reviewer #2:
The manuscript, entitled "Disordered gut microbiota and alterations in metabolic patterns are associated with atrial fibrillation", by K Zuo etc. compared gut microbiome, fecal and serum metabolomics profile between patients with atrial fibrillation (AF) and healthy controls and found a lot of microbiota community shifts and metabolite discrimination, characterized by elevated richness and increased diversity of microbes and decreased fatty acids, and concluded that gut microbiota dysbiosis in AF patients and the subsequent metabolomics profile changes might be the driver to AF pathogenesis. This manuscript was well organized and read smoothly, the findings are very interesting. However, several concerns still need be addressed.

Question: 1) Authors investigated gut microbiota shifts based on metagenomic sequencing, which should allow authors to compare microbes at the strain level. However, it seems that authors mostly used genus level except species level used in Figure S4 for comparison.

Response: Thanks so much for this valuable suggestion. Besides analysis of species strikingly different across groups in figure S4, we further analyzed the enterotype in species level, which was shown in figure S3. Similar difference in enterotype distribution at the species level was also found, although no significant different species were found between enterotypes. 57.14% CTRs in enterotype 1, 42.86% CTRs in enterotype 2. 80.43% AFs in enterotype 1, 19.57% AFs in enterotype 2. p = 0.01662, CTR vs AF; Fisher’s exact test (page 10, #line 263 in the revision; page 10, #line 194 in the clean revision).

In addition, we performed the correlation analyses between different metabolites and species in figure 6d (page 20, #line 575 in the revision; page 20, #line 398 in the clean revision). For example, LA and ALA, previous described as cardiovascular protectors, were negatively associated with species like Prevotella. copri. The close relationship between microbes and metabolites indicate the specific metabolites might be produced at least indirectly by corresponding gut microbe, which remains further investigation.

Question: 2) Authors stated that the elevated richness and increased diversity of microbes is due to overgrowth of pathogenic bacterium, but only E. coli was mentioned. More analysis by making full use of the metagenomic data will be a plus.

Response: Thanks for this suggestion. At the genus level, elevated proportion of Streptococcus, Enterococcus, Veillonella, and Ruminococcus were described and the potential pathogenic effect was discussed as below (page 11, #line 304 in the revision; page 11, #line 222 in the clean revision). "Ruminococcus is known to possess a pro-inflammatory property, which was is implicated in the development of inflammatory bowel disease. Transplantation of Ruminococcus into germfree mice enhanced the levels of interforon-γ, interleukin-17 and interleukin-22. Streptococcus, recognized as a morbific oral bacteria, has also been demonstrated to be elevated in HTN, congestive heart failure (CHF) and atherosclerotic cardiovascular disease (ACVD). Furthermore, Veillonella, a Gram-negative anaerobic coccus, was suggested to be inversely correlated with cardiovascular protective metabolites such as niacin, cinnamic acid and orotic acid. In addition, Enterococcus is known to produce cytolysin, a toxin that causes rupture of a variety of target membranes, including bacterial cells, erythrocytes and other mammalian cells."
While at the species level, we supplemented discussions about several species differently enriched among controls and AFs (page 12, #line 331 in the revision; page 12, #line 236 in the clean revision). Eubacterium rectale was reported increased in the intestines of patients with hypertension. Eubacterium rectale is a main representative of Firmicutes and a kind of conditioned pathogen, which can ferment the metabolic products of glucose (such as formic acid, acetic acid and butyric acid) as well as proteins, thereby inhibiting the proliferation of other beneficial bacteria in the intestines and decreasing catabolic enzymes of glycan (Riviere, A., et al. Appl Environ Microbiol. 2015; 81: 7767-7781.). Furthermore, species enriched in AF group including Bifidobacterium longum, Collinsella aerofaciens, were more abundant in responders in metastatic melanoma patients (Matson, V., et al. Science. 2018; 359: 104-108.). Meanwhile, control group enriched species such as Faecalibacterium prausnitzii (Takahashi, K., et al. Digestion. 2016; 93: 59-65.), the butyrate-producing bacterial species was found decreased in AF group. The results showed the imbalanced structure of intestinal floras, decreased number of probiotics and increased quantity of harmful bacteria in patients with AF.

Question: 3) In the demographic Table, the two groups show some discrimination between age and gender, authors should test the age and gender effect on the gut microbiota shift.

Response: Thanks for this valuable suggestion. Considering the difference of gender, age, we performed PCA plot to assess the contribution of these factors. The results showed that PCA failed to distinguish AF patients into separated group based on either gender or age. The distribution of CTR and AF individuals separated obviously, while individuals in different gender and age mixed together. Therefore, the impact of gender, age on our result seemed negligible ($p>0.05$, Anosim, Additional files 10, figure S6) (page 13, #line 361 in the revision; page 13, #line 261 in the clean revision).

Question: 4) There are 50 patients in each group, however, authors only present metabolomics profile from 8 AF patients and 12 controls. Is it possible for authors to provide all the patients' metabolic profile?

Response: Thanks so much for this valuable suggestion. For multiple reasons, we were not able to get enough sample for metabonomics analysis from all the patients. And finally, sixty-five (n=65) individuals from our study cohort, with 36 controls and 29 AFs, were subjected to metabonomics analysis on serum samples. Fifty-nine (n=59) individuals were subjected to metabonomics analysis on feces samples. This cohort was composed of 17 controls, and 42 patients of AF. And the results referring to metabonomics has been updated (page 18, #line 492 in the revision; page 18, #line 349 in the clean revision).

Question: 5) In Page 31, the metabolomics analysis method can be shortened since both fecal samples and serum samples used the same LC condition.

Response: We have shortened the metabolomics analysis method, and revised this part in (page 33, #line 882 in the revision; page 33, #line 652 in the clean revision)

Question: 6) In page 32, #line 638, authors said that "Data were then normalized", authors should provide how to normalize and normalize to what.

Response: Considering the remarkable differences existed among various metabolites, some signals of metabolites with too high or low concentration might be covered up and failed to be identified as biomarkers. So, normalization, aiming to adjust the weight of different variables to decrease the gap of different signals, should be performed to make the dimension (for example mean and standard deviation) of all variables in a similar level and make the data more comparable. The calculation process was to normalize the peak area of each sample to 1000000 and divide the peak area of each ion by the total peak area of the sample and multiplied by 1000000 (page 33, #line 889 in the revision; page 33, #line 659 in the clean revision).

Respond to Reviewer #3:
Zuo et al reported a study indicating disordered gut microbiota and metabolic changes in patients with atrial fibrillation. The manuscript is well written and the results are well described. However, I have a couple comments below to improve the quality of manuscript.

Question: 1) In this case-control study, authors used cases and controls from different hospitals of two different cities. Table 1 also indicated the differences in age, gender and total cholesterol between case and control groups. Thus, authors should justify more on potential selection bias and possible confounding effects.

Response: Thanks for this valuable suggestion. It was true that AFs were recruited from Beijing and controls from Kailuan. The two cities were both located in northern China, and shared a similar life style. However, not all the subjects recruited from Beijing hospital were permanent residents of Beijing, so it is in Kailuan hospital. Some of the subjects were nearby residents who came to Beijing or Kailuan for medical treatment. Therefore, the result from the current study represented the characteristics of the population in northern China, but not one or two cities.
Furthermore, as described in table 1, HTN has been matched yet, and there were 27 participants diagnosed with HTN in both AF and control group.
Considering the difference of gender, age, TC and T2DM, we performed PCA plot to assess the contribution of different baseline characteristics. The results showed that PCA failed to distinguish AF patients into separated group based on these factors, and the distribution of CTR and AF individuals separated obviously, while the distribution of individuals in different baseline characteristics mixed together. Therefore, the impact of gender, age, TC or T2DM on our data seemed negligible ($p>0.05$, Anosim, Additional files 12, figure S6) (page 13, #line 361 in the revision; page 13, #line 261 in the clean revision).
We did not exclude AF patients with HTN or T2DM as mentioned in limitation, while both HTN, T2DM, cholesterol and aging were strong risk factor in the development of AF. And these risk factors involved in the process of oxidative stress, endothelial dysfunction and atrial remodeling, while formed the risky mutual relationships. So, in the current 100 participants, the phenomenon of elder AF patients was explicable either.

Question: 2) Although this is a pioneer study under this topic, authors should elaborate more on sample size justification.

Response: 50 controls and 50 AFs were recruited in the present study. To assess the sequencing depth, we performed rarefaction curve for gene number after 50 random sampling with replacement. As shown in figure 1d, rarefaction curve was nearly smooth, with few new genes undetected as the increase of sample size at the end stage of the curve. So, the sequencing data has been great enough and the present sample size has met the need of this study. Furthermore, the current study is still at the discovery stage, and the sample size will be expanded in validation stage to illustrate the result in

further studies.

We greatly appreciate your interest and encouragement concerning our manuscript. Believing that all the concerns raised by the reviewers have been addressed. We look forward to receiving comments from you. If you have any questions, please don't hesitate to contact me at the address below.

Sincerely yours,

Xinchun Yang, MD, PhD
Heart Center, Beijing ChaoYang Hospital, Capital Medical University,
Beijing Key Laboratory of Hypertension,
8th Gongtinanlu Rd, Chaoyang District, Beijing, China, 100020
Tel: 86-10-85231937
Fax: 86-10-85231937
E-mail: yxc6229@163.com

Jiuchang Zhong, MD, PhD
Heart Center, Beijing ChaoYang Hospital, Capital Medical University,
Beijing Key Laboratory of Hypertension,
8th Gongtinanlu Rd, Chaoyang District, Beijing, China, 100020
Tel: 86-10-85231937
Fax: 86-10-85231937
E-mail: jiuchangzhong@aliyun.com

Close
