# Supplementary material for: Disordered gut microbiota and alterations in metabolic patterns are associated with atrial fibrillation
Source: Gigascience. 2019 May 30;8(6):giz058. doi: 10.1093/gigascience/giz058 (PMC6543127; doi:10.1093/gigascience/giz058)
Supplement: giz058_Response_to_Reviewer_Comments_Revision_1 [file giz058_response_to_reviewer_comments_revision_1.pdf]

Author's Response To Reviewer Comments

Close

Dear editor,

Thank you very much for your consideration and encouragement on our manuscript entitled "Disordered gut microbiota and alterations in metabolic patterns are associated with atrial fibrillation" (GIGA-D-18-00364R1). We thank reviewers for their professional and positive comments to improve our manuscript. We have considered the comments from the editors and reviewers and revised the manuscript. Furthermore, we have made the raw metabolomics MS data openly available under Metabolomics Workbench and add the accession details in the manuscript. The metabolomics data was available at the NIH Common Fund's Data Repository and Coordinating Center website with Metabolomics Workbench Study ID: ST001168 (for fecal metabolomic analyses) and ST001169 (for serum metabolomic analyses). And the data are directly accessible at
http://dev.metabolomicsworkbench.org:22222/data/DRCCMetadata.php?Mode=Study&StudyID=ST001168 (for fecal metabolomic analyses);
http://dev.metabolomicsworkbench.org:22222/data/DRCCMetadata.php?Mode=Study&StudyID=ST001169 (for serum metabolomic analyses) (page 36, #line 715 in the revision).

Point-by-point replies are listed as below.
A list of the changes in Tables
Table S15 was updated with report of ppm and change “0” to “/” in column of “score”.

Respond to Reviewer #1:
The authors have improved the manuscript sustainably. I have some small comment for authors to check and report in the manuscript.
Question: 1) When the authors identified metabolite names from exact mass using METLIN please specify the tolerant cut-off in ppm. The ppm different result obtain from METLIN query needs to be reported in supplementary the table S15.
Response: Thanks for this valuable suggestion. Indeed, ppm value is important during the process of metabolites identification, which refers to the error between molecular mass detected by the instrument and the molecular mass in the database. The lower ppm means the closer molecular mass detected by the instrument and the molecular mass in the database. The cut-off value of ppm in the current study is set less than 25. The ppm value in this study is mainly lower than 10, which has been supplemented in table S15 (page 34, #line 674 in the revision; page 34, #line 673 in the clean revision).

Question: 2) -In supplementary table S 15 why score of L-Threonine and α-Linolenic Acid are 0 ??
Response: Thanks for this suggestion. It would be reasonable to change “0” to “/” in column of “score” in table S15. Because the score of L-Threonine and α-Linolenic were failed to find in the database. The second-order mass spectrometry is even more difficult to find due to the low intensity of the parent ion in sample. Therefore, the exact molecular mass was used to identify the metabolites related to the featured peak in the Metlin database (http://metlin.scripps.edu).

We greatly appreciate your interest and encouragement concerning our manuscript. We look forward to receiving comments from you. If you have any questions, please don't hesitate to contact me at the address below.

Sincerely yours,

Xinchun Yang, MD, PhD
Heart Center, Beijing ChaoYang Hospital, Capital Medical University,
Beijing Key Laboratory of Hypertension,
8th Gongtinanlu Rd, Chaoyang District, Beijing, China, 100020
Tel: 86-10-85231937

Fax: 86-10-85231937
E-mail: yxc6229@163.com

Jiuchang Zhong, MD, PhD
Heart Center, Beijing ChaoYang Hospital, Capital Medical University,
Beijing Key Laboratory of Hypertension,
8th Gongtinanlu Rd, Chaoyang District, Beijing, China, 100020
Tel: 86-10-85231937
Fax: 86-10-85231937
E-mail: jiuchangzhong@aliyun.com

Close
