# Supplementary material for: Disordered gut microbiota and alterations in metabolic patterns are associated with atrial fibrillation
Source: Gigascience. 2019 May 30;8(6):giz058. doi: 10.1093/gigascience/giz058 (PMC6543127; doi:10.1093/gigascience/giz058)
Supplement: giz058_Reviewer_1_Report_Original_Submission -- Intawat Nookaew — 11/20/2018 Reviewed [file giz058_reviewer_1_report_original_submission.pdf]

Reviewer Report

Title: Disordered gut microbiota and alterations in metabolic patterns are associated with atrial fibrillation

Version: Original Submission **Date:** 11/20/2018

Reviewer name: Intawat Nookaew

Reviewer Comments to Author:

Zuo et al present a manuscript titled "Disordered gut microbiota and alterations in metabolic patterns are associated with atrial fibrillation" .The manuscript describes the study of 100 subjects (50 AF vs 50 control) using shot gun meta genomic and metabolomic analysis of fecal and serum to study the impact of microbiome on the Atrial Fibrillation disease. The study generated a lot of data. However, I found many points that need to be improved and clarified as below
- The authors stated that "To adjust for the effect of HTN on gut microbiota composition, we selected 50 samples from our previous gut microbiota work matched for a history." There is no reference or details to following this statement.
- Medications is a key factor that can alter gut microbiome as shown in previous study (e.g. https://www.nature.com/articles/nm.4345). There is no report of control medication? I recommend the author report medication in a systematic way in a table because it is very important information.
- I recommend the author make a better figure 2d to visualize the higher percentage AF patients in enterotype 1 by changing the dot types in different shape. This will make the reader to catch the claim up from the figure.
- The authors reported the statistical different of genera between AF and control in FigS3 and S4. I recommend the author to put statistical values details in the figures.
- The author reported the small influence of T2DM and TC on the gut microbiota change using PLS-SEM as shown in figure S5. However, the most different phenotype is age as show in the table 1. I strongly recommend the authors perform the analysis to see the effect of age, gender and HTN confounders. This is very important issue for the conclusion of the paper.
- Figure 3 is meaning less I suggest to put in supplementary. Taxonomic annotation of CAG is also unclear for me in M&M. The author need to provide a better description to assign taxonomic of CAG. What is "the taxonomy of tracer genes" , no reference about this ?? Author need to provide the confident of taxonomic annotation confidence of individual CAG. Moreover, distribution of CAG size (number of genes) should we plot to see. I think 50 gene cut-off for CAG as mentioned in M&M is proper. It is too small !.
- What is OR score ??? no full term mentioned in the manuscript.
- Figure 4 legend need to be improved, not all components, and details were explained clearly
- The result of CAG and KEGG and eggNOG function should be coordinated. The author reported them separately. For example, the gene that significant different is and KEGG and eggnog analysis is associate with the high score CAG ??
- Statistical values should report in the Figure 5

- The author then measures metabolites in fecal, serum using LC-MS in positive and negative mode. It is not clear in M&M how the author handle and analyze data such as normalization method, how many features is detected and use in the analysis.
- How to identify compound name - confident score need to be report. It is very critical information for conclusion. Most of the time people interpret wrong so confident values of compound in dentification (name) is needed.
- The overlap compound in figure 7 is based on names or chromatogram and mass spectra. It is very important to check that they have similar MS pattern because, as I mentioned before on the issue of compound identification is not well report.
- For reproducibility of research especially data analysis, step by step for bioinformatic analysis as computational note book need to be provide for transparency as example in this paper https://www.ncbi.nlm.nih.gov/pmc/articles/PMC4940747/. I am very serious about this.
- I cannot get any supplementary tables at all.
- No information on data deposition for shot gun sequencing and metabolome. It is very essential.
- In general, all figure legends need to be improved
- It would be fantastic if the study have dietary information of the subject.

Methods

Are the methods appropriate to the aims of the study, are they well described, and are necessary controls included? Choose an item.

Conclusions

Are the conclusions adequately supported by the data shown? Choose an item.

Reporting Standards

Does the manuscript adhere to the journal's guidelines on minimum standards of reporting? Choose an item.

Choose an item.

Statistics

Are you able to assess all statistics in the manuscript, including the appropriateness of statistical tests used? Choose an item.

Quality of Written English

Please indicate the quality of language in the manuscript: Choose an item.

Declaration of Competing Interests

Please complete a declaration of competing interests, considering the following questions:

- Have you in the past five years received reimbursements, fees, funding, or salary from an organisation that may in any way gain or lose financially from the publication of this manuscript, either now or in the future?
- Do you hold any stocks or shares in an organisation that may in any way gain or lose financially from the publication of this manuscript, either now or in the future?
- Do you hold or are you currently applying for any patents relating to the content of the manuscript?
- Have you received reimbursements, fees, funding, or salary from an organization that holds or has applied for patents relating to the content of the manuscript?
- Do you have any other financial competing interests?
- Do you have any non-financial competing interests in relation to this paper?

If you can answer no to all of the above, write 'I declare that I have no competing interests' below. If your reply is yes to any, please give details below.

'I declare that I have no competing interests'

I agree to the open peer review policy of the journal. I understand that my name will be included on my report to the authors and, if the manuscript is accepted for publication, my named report including any attachments I upload will be posted on the website along with the authors' responses. I agree for my report to be made available under an Open Access Creative Commons CC-BY license (http://creativecommons.org/licenses/by/4.0/). I understand that any comments which I do not wish to be included in my named report can be included as confidential comments to the editors, which will not be published.

I agree to the open peer review policy of the journal

To further support our reviewers, we have joined with Publons, where you can gain additional credit to further highlight your hard work (see: https://publons.com/journal/530/gigascience). On publication of this paper, your review will be automatically added to Publons, you can then choose whether or not to claim your Publons credit. I understand this statement.

Yes Choose an item.
