# Supplementary material for: Disordered gut microbiota and alterations in metabolic patterns are associated with atrial fibrillation
Source: Gigascience. 2019 May 30;8(6):giz058. doi: 10.1093/gigascience/giz058 (PMC6543127; doi:10.1093/gigascience/giz058)
Supplement: giz058_Reviewer_2_Report_Original_Submission -- Zeneng Wang — 11/25/2018 Reviewed [file giz058_reviewer_2_report_original_submission.pdf]

Reviewer Report

Title: Disordered gut microbiota and alterations in metabolic patterns are associated with atrial fibrillation

Version: Original Submission **Date:** 11/25/2018

Reviewer name: Zeneng Wang

Reviewer Comments to Author:

The manuscript, entitled "Disordered gut microbiota and alterations in metabolic patterns are associated with atrial fibrillation", by K Zuo etc compared gut microbiome, fecal and serum metabolomics profile between patients with atrial fibrillation (AF) and healthy controls and found a lot of microbiota community shifts and metabolite discrimination, characterized by elevated richness and increased diversity of microbes and decreased fatty acids, and concluded that gut microbiota dysbiosis in AF patients and the subsequent metabolomics profile changes might be the driver to AF pathogenesis. This manuscript was well organized and read smoothly, the findings are very interesting. However, several concerns still need be addressed.
Authors investigated gut microbiota shifts based on metagneomic sequencing, which should allow authors to compare microbes at the strain level. However, it seems that authors mostly used genus level except species level used in Figure S4 for comparison. Authors stated that the elevated richness and increased diversity of microbes is due to overgrowth of pathogenic bacterium, but only E. coli was mentioned. More analysis by making full use of the metagenomic data will be a plus.
In the demographic Table, the two groups show some discrimination between age and gender, authors should test the age and gender effect on the gut microbiota shift.
There are 50 patients in each group, however, authors only present metabolomics profile from 8 AF patients and 12 controls. Is it possible for authors to provide all the patients' metabolic profile? In Page 31, the metabolomics analysis method can be shortened since both fecal samples and serum samples used the same LC condition. In page 32, #line 638, authors said that "Data were then normalized", authors should provide how to normalize and normalize to what.

Methods

Are the methods appropriate to the aims of the study, are they well described, and are necessary controls included? Choose an item.

Conclusions

Are the conclusions adequately supported by the data shown? Choose an item.

Reporting Standards

Does the manuscript adhere to the journal's guidelines on [minimum standards of reporting?] Choose an item.

Choose an item.

Statistics

Are you able to assess all statistics in the manuscript, including the appropriateness of statistical tests used? Choose an item.

Quality of Written English

Please indicate the quality of language in the manuscript: Choose an item.

Declaration of Competing Interests

Please complete a declaration of competing interests, considering the following questions:

- Have you in the past five years received reimbursements, fees, funding, or salary from an organisation that may in any way gain or lose financially from the publication of this manuscript, either now or in the future?
- Do you hold any stocks or shares in an organisation that may in any way gain or lose financially from the publication of this manuscript, either now or in the future?
- Do you hold or are you currently applying for any patents relating to the content of the manuscript?
- Have you received reimbursements, fees, funding, or salary from an organization that holds or has applied for patents relating to the content of the manuscript?
- Do you have any other financial competing interests?
- Do you have any non-financial competing interests in relation to this paper?

If you can answer no to all of the above, write 'I declare that I have no competing interests' below. If your reply is yes to any, please give details below.

'I declare that I have no competing interests'

I agree to the open peer review policy of the journal. I understand that my name will be included on my report to the authors and, if the manuscript is accepted for publication, my named report including any attachments I upload will be posted on the website along with the authors' responses. I agree for my report to be made available under an Open Access Creative Commons CC-BY license (http://creativecommons.org/licenses/by/4.0/). I understand that any comments which I do not wish to be included in my named report can be included as confidential comments to the editors, which will not be published.

I agree to the open peer review policy of the journal

To further support our reviewers, we have joined with Publons, where you can gain additional credit to further highlight your hard work (see: https://publons.com/journal/530/gigascience). On publication of this paper, your review will be automatically added to Publons, you can then choose whether or not to claim your Publons credit. I understand this statement.

Yes Choose an item.
